# Exploring Therapeutic Targets for Preventing Cardiac Arrest by Modulating Dyslipidemia and 25-Hydroxyvitamin D Metabolism: A Mendelian Randomization Study

**DOI:** 10.1155/humu/5536318

**Published:** 2025-06-19

**Authors:** Xinya Jia, Keke Du, Yuanting Zhu, Liuyang Xie, Tangjuan Zhang, Liu Yang, Yuepeng Hu, Chao Lan, Qiang Zhang

**Affiliations:** ^1^Department of Emergency Medicine, The First Affiliated Hospital of Zhengzhou University, Zhengzhou, China; ^2^Henan Engineering Research Center for Cardiopulmonary and Cerebral Resuscitation, The First Affiliated Hospital of Zhengzhou University, Zhengzhou, China; ^3^Zhengzhou Key Laboratory for Sudden Death Prevention and Precision Resuscitation of Cardiac Arrest, The First Affiliated Hospital of Zhengzhou University, Zhengzhou, China; ^4^Department of Cardiology, The Seventh Affiliated Hospital of Sun Yat-sen University, Shenzhen, China

**Keywords:** cardiac arrest, dyslipidemia, Mendelian randomization

## Abstract

Cardiac arrest (CA) prevention continues to be a substantial hurdle for global public health. Although dyslipidemia and 25-hydroxyvitamin D (25(OH)D) insufficiency are recognized contributing factors for cardiovascular disease (CVD), their causal relationship with CA risk is still uncertain. Here, we explored these correlations and pinpointed possible therapeutic targets for CA prevention though Mendelian randomization (MR). Both two-sample and multivariable MR analysis methods were conducted to assess how serum lipid traits and 25(OH)D influence the susceptibility to develop CA. Nine thousand nine hundred eighty-eight participants in total from the National Health and Nutrition Examination Survey (NHANES) engaged in validating the relationship between the concentrations of 25(OH)D and cardiovascular mortality in individuals with dyslipidemia. The integration of MR with expression quantitative trait locus (eQTL) analysis enabled the identification of druggable targets, and molecular docking was used to screen small molecules, which were subsequently validated in animal models. The MR results revealed that both elevated levels of low-density lipoprotein cholesterol (LDL-C) and apolipoprotein B (ApoB), as well as triglycerides (TGs), significantly contributed to an increased CA risk (*p* < 0.05). Conversely, higher amounts of apolipoprotein A1 (ApoA1), high-density lipoprotein cholesterol (HDL-C), and 25(OH)D were causally contributing to a decreased risk of CA (*p* < 0.05). A bidirectional causal relationship was observed among LDL-C, TG, ApoB, and 25(OH)D levels. Mediation MR suggests that dyslipidemia and low 25(OH)D status could potentially elevate the CA risk through pathways involving myocardial infarction, diabetes, and hypertension. NHANES data confirmed that higher 25(OH)D were tied to decreased risks of all-cause and CVD death among those with dyslipidemia (*p* < 0.01). Notably, chromobox 6 (CBX6), negatively associated with CA risk (OR = 0.87, 95% CI: 0.78–0.99, *p* = 0.029), was determined to be a target of both sanguinarine and lycorine, which improved lipid profiles and 25(OH)D in mice. In conclusion, dyslipidemia and low 25(OH)D status are causally related to CA risk, they appear to interact, and their coexistence may confer a higher risk of CVD mortality. Compounds targeting specific genes can both improve dyslipidemia and elevate 25(OH)D levels, thereby exhibiting potential therapeutic effects for preventing CA. Overall, this study enhances our understanding of the underlying mechanisms linking dyslipidemia, 25(OH)D deficiency, and CA and offers new perspectives for prevention.

## 1. Introduction

Cardiac arrest (CA), which presents a significant challenge to global healthcare systems, is an unexpected event that is often lethal. Despite significant advances in prehospital, emergency, and critical care medicine, CA continues to have a high mortality rate. Recent studies have reported that more than 356,000 out-of-hospital cardiac arrest (OHCA) cases occur annually across the United States, accompanied by a mortality rate of around 90%, while European data indicate an estimated 89 OHCA instances among 100,000 people annually, and the survival rate, on average, stands at just 8% [1]. Targeted temperature management is considered an effective strategy for reducing post-CA injuries, although its clinical benefits remain controversial because of the high incidence of complications [2]. The clinical management of CA is challenging and characterized by the lack of effective interventions and targeted pharmacotherapies. Therefore, identifying risk factors for CA and implementing appropriate interventions in at-risk populations are effective strategies to alleviate the associated disease burden.

Although CA is most commonly observed in individuals with pre-existing structural heart disease, it can also affect healthy individuals with few or no known cardiovascular risk factors. As CA is a sudden and unpredictable emergency, additional research is of critical importance to pinpoint the potential associated detriments. Previous studies have highlighted several independent CA determinants such as diabetes, asthma, and hypertension, and most of them are irreversible. Therefore, effective preventive strategies implemented prior to disease onset are crucial. Early intervention is paramount to mitigate the risk of CA and improve patient outcomes [3]. However, several modifiable risk factors for future cardiovascular events are well established, including metabolic syndrome and lifestyle factors, such as dyslipidemia, 25(OH)D, and body mass index (BMI), highlighting the significant role of these factors in preventing disease onset [4].

Dyslipidemia and inadequate 25(OH)D amounts are common throughout the world and significantly associated with metabolic disorders including obesity, diabetes, and atherosclerosis [5, 6]. Increasing evidence suggests that both conditions contribute substantially to the onset of cardiovascular disease (CVD), while CVD is known to act as a major contributing condition for the occurrence of CA. Nevertheless, the connection among 25(OH)D status, dyslipidemia, and CA remains unclear. Although certain observational investigations have suggested a strong link among dyslipidemia, 25(OH)D, CVD risk, and its related mortality [7, 8], these studies often face limitations due to methodological challenges and the possibility of uncontrolled confounding factors. Consequently, the causal relationship between dyslipidemia, 25(OH)D, and CA risk remains undetermined. Moreover, reduced 25(OH)D is often detected together with augmented low-density lipoprotein cholesterol (LDL-C) and triglyceride (TG) levels [9]; however, whether there is a causal relationship between them still requires further exploration.

Due to the lack of effective treatments and the urgent need for preventive strategies, recognizing and comprehending the risk factors that can be modified is of utmost clinical importance. Mendelian randomization (MR), applying genetic polymorphisms as instrumental variables, provides a robust approach to infer causality and minimize confounding. Therefore, in this study, we used both two-sample and multivariable Mendelian randomization (MVMR) analyses to investigate the potential causal effects of lipid traits and 25(OH)D levels on CA risk, and data from the National Health and Nutrition Examination Survey (NHANES) further confirm the results. We also employed expression quantitative trait locus (eQTL) data and molecular protein docking techniques, aiming to find potential therapeutic targets for CA prevention by modulating dyslipidemia and 25(OH)D metabolism, thereby contributing to a deeper understanding of CA prevention.

## 2. Materials and Methods

### 2.1. Study Design

**Figure 1:**
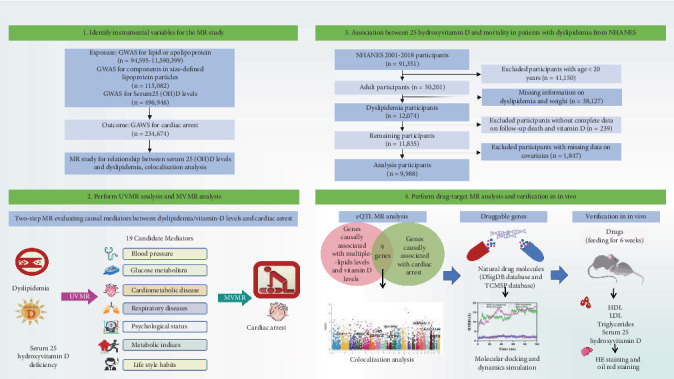
Overview of the study design. This study consisted of four stages of analyses. In Stage 1, we assessed the causal associations of various serum lipid and APO traits, as well as 25(OH)D levels on cardiac arrest, and we also conducted an MR study for the relationship between 25(OH)D levels and lipid traits. In Stage 2, 19 candidate mediators in the pathway from exposure to outcome were selected, and a two-step Mendelian randomization (MR) analysis was conducted to evaluate the mediating effects of each selected mediator on the causal association between serum lipid traits or 25(OH)D levels and cardiac arrest risk. In Stage 3, we investigated the association between 25(OH)D levels and mortality in patients with dyslipidemia using data from the NHANES database. In Stage 4, through molecular docking and molecular dynamics simulation, the selected natural drugs were subsequently validated in in vivo experiments. 25(OH)D, 25-hydroxyvitamin D.

Figure 1 presents a summary of the study, which was structured into four distinct phases. Initially, a univariate Mendelian randomization (UVMR) examination was executed to determine the total effect of lipid traits, apolipoprotein (Apo) traits, and 25(OH)D on the probability of CA. A bidirectional MR examination was then utilized to evaluate whether traits linked to serum lipid and Apo levels exerted a causal influence on 25(OH)D, or conversely. Colocalization analysis further supported the presence of a causal relationship among these factors. Following that, 19 mediators are being considered as candidates that may be involved in the occurrence of CA in the causal pathway. A two-stage MR examination was used to evaluate the intermediary influences of each selected moderator in the causal connection between serum lipid traits or 25(OH)D and CA risk. Thirdly, we explored the correlation between the amounts of 25(OH)D and mortality in patients with dyslipidemia using data from the NHANES. Finally, drug-target MR analysis was conducted to identify potential therapeutic targets. Subsequently, the selected natural drugs were validated using in vivo experiments. The methods of the MR analysis were carried out following the guidelines of the STROBE-MR checklist [10], and additional information is detailed in Supporting Information 4: Table S1.

### 2.2. Genome-Wide Association Study (GWAS) Data Source

The analysis employed GWAS summary statistics, which were sourced from publicly available datasets provided by relevant authoritative consortiums. Summary statistics for high-density lipoprotein cholesterol (HDL-C), LDL-C, ApoA1, ApoB, serum 25(OH)D levels, and size-dependent lipid profiles in lipoprotein particles came from the UK Biobank. TG-related summary data were derived from European cohorts, and CA-related datasets were obtained from the FinnGen Biobank (Supporting Information 4: Table S2). The samples in the exposure and outcome datasets did not overlap, maintaining analytical independence. The eQTL-related data in this article were sourced from the eQTL GenConsortium. A total of 16,987 genes and 31,684 trait-associated single nucleotide polymorphisms (SNPs) from individuals of European descent were included in the eQTLGen dataset. The following quality control conditions were applied to ensure that SNPs functioned as instrumental variables. In the course of our analysis, all SNPs included in the preliminary analysis exhibited a *p* value of at least 5 × 10^−8^, indicating suggestive significance. The clumping process (*r*^2^ < 0.001 within a 10,000 kb window) was performed to assess linkage disequilibrium (LD) between the SNPs.

### 2.3. Genetic Variant Selection

The designated standards were applied to identify appropriate genetic instruments that optimally fulfilled the three foundational requirements for MR analysis (Supporting Information 1: Figure S1). In particular, SNPs linked to serum lipid traits were selected to serve as genetic variables for instrumental analysis with significance (*p* < 5 × 10^−8^). The same selection criteria were applied to investigate SNPs associated with concentrations of 25(OH)D. SNPs strongly correlated (*p* < 5 × 10^−5^) with potential confounders of CA were excluded using data from the IEU Open GWAS database (https://gwas.mrcieu.ac.uk/). Current evidence suggests that ischemic cardiomyopathy, hypertrophic cardiomyopathy, aortic aneurysm, obesity, depressive disorders, and lifestyle-related factors are all potential contributors to CA [11–13]. Therefore, we included them as confounding factors in this study. The identified confounders and their associated SNPs are presented in Supporting Information 4: Table S4.

### 2.4. MR Analysis

For UVMR, we performed the analysis by matching the identified instrumental variables against the outcome summary data, ensuring the effect direction aligned with the effect alleles. The method of random effects inverse variance weighted (IVW) was utilized to aggregate effect estimates of SNPs on the exposure and outcome variables, thereby evaluating the overall causal effect. To address the horizontal pleiotropy, weighted median and weighted mode tests were performed. Cochran's *Q* statistic and the MR-Egger test were also applied to assess heterogeneity and pleiotropy [14, 15]. A causal relationship was considered statistically significant only when both requirements listed below were fulfilled: the *p* value of IVW was less than the predetermined significance threshold and the estimated effect directions were consistent across all employed methods. For MVMR analysis, a multivariate IVW was applied as the principal method of analysis. Complementary methods including MVMR-median and MVMR-Egger were also implemented. The the assessment of horizontal pleiotropy took into account the intercept from the MVMR-Egger regression and the corresponding significance level (*p* value).

### 2.5. Selection of Mediators and Two-Step MR

Potential mediators within the causal pathway connecting dyslipidemia, 25(OH)D, and CA were selected according to the criteria below: (1) presence of causal relationship involving dyslipidemia, 25(OH)D, and the associated mediators; (2) a causal association between all mediators and CA; (3) no evidence of inverse causality among the mediators, lipid traits, and concentrations of 25(OH)D; (4) the consistency in the direction of each mediator' s influence and the overall effect of dyslipidemia and 25(OH)D on CA. Mediation analysis was implemented through a two-phase method. Firstly, the genetic causal impacts predicted dyslipidemia and 25(OH)D on the mediators were assessed using UVMR. Subsequently, MVMR was applied to estimate the distinct influences (*β*2) of mediators on CA while accounting for dyslipidemia and 25(OH)D. For every mediator, the mediating effect (*β*1 × *β*2) was obtained, and the mediation proportion was determined through dividing this effect by the total effect (*β*3). All statistical procedures were performed using the TwoSampleMR package and MR package in the R software (Version 4.3.1).

### 2.6. Data Sources for Potential Mediators

Disrupted lipid profiles and 25(OH)D deficiency have detrimental effects on various physiological processes, including blood pressure and glucose regulation, cardiovascular and respiratory health, and psychological disorders, along with lifestyle-related outcomes [16–21]. Consequently, these factors were recognized as candidate intermediates in the causal pathway from dyslipidemia or 25(OH)D deficiency to CA; the specific potential mediators are as follows:

1. Three traits related to blood pressure were considered [16]. Data regarding hypertension (*n* = 462,933), systolic blood pressure (SBP) (*n* = 97,656), diastolic blood pressure (DBP) (*n* = 436,424) were obtained from within-family consortium and MRC-IEU consortium.
2. Two insulin and glycemic-related traits were considered [17]. Type 2 diabetes mellitus data (*n* = 84,780) were sourced from the DIAGRAM consortium, along with HbA1c (*n* = 45,734) from the MAGIC consortium.
3. Three traits related to cardiometabolic diseases were included. Myocardial infarction (MI, *n* = 187,840) data were obtained from the FinnGen Biobank, angina pectoris (AP, *n* = 470,931) data were obtained from the UK Biobank consortium, and atrial fibrillation (AF, *n* = 463,010) data were obtained from the MRC-IEU consortium.
4. Two respiratory disease-related traits were included [18, 19]. Data on asthma (*n* = 26,475) were obtained from the GABRIEL consortium, and that on chronic obstructive airway disease (*n* = 462,933) from the MRC-IEU consortium.
5. Five traits concerning physical morphology and metabolic indices were included. Waist circumference (WC, *n* = 336,639) and hip circumference (HC, *n* = 336,601) data were obtained from the Neale Lab consortium. Data on weight (*n* = 454,893), basal metabolic rate (BMR, *n* = 454,874), and BMI (*n* = 454,893) came from the MRC-IEU consortium.
6. Two lifestyle habits were considered [20]. Alcohol consumption (*n* = 335,394) and smoking data (*n* = 607,291) were obtained from the GSCAN consortium.
7. Two psychological states were included [21]. Anxiety (*n* = 463,010) and depression data (*n* = 462,933) were obtained from the MRC-IEU Biobank.

### 2.7. Study Population in NHANES

All NHANES data applied are openly available at the link https://www.cdc.gov/nchs/n-hanes/, and it was accessed on December 8, 2024. This study covered nine 2-year cycles starting from 2001 and ending in 2018, and the sample consisted of 50,201 individuals aged 20 years and older. The following exclusion criteria were applied: participants without dyslipidemia or missing data on WTSAF2YR (*n* = 38,127); participants with missing data on levels of 25(OH)D, all-cause mortality, and mortality of CVD (*n* = 239); and those with incomplete covariate data (*n* = 1850). The final analysis comprised 9988 participants.

### 2.8. Assessment of Dyslipidemia and 25(OH)D in NHANES

Participants were considered to have dyslipidemia if they satisfied any of the following conditions: total cholesterol (TC) exceeding 6.21 mmol/L, TG over 2.26 mmol/L, LDL-C above 4.14 mmol/L, HDL-C below 1.03 mmol/L in males or below 1.29 mmol/L in females, and intake of lipid-regulating drugs or a diagnosis of dyslipidemia by a physician [22]. 25(OH)D levels were grouped into four predefined categories for analytical purposes according to the current standards [23, 24]: < 25.00 nmol/L (severe deficiency), 25.00–49.99 nmol/L (deficiency), 50.00–74.99 nmol/L (insufficiency), and ≥ 75.00 nmol/L (sufficiency).

### 2.9. Colocalization Analysis

Colocalization analysis is conducted to determine whether both of the associated traits are driven by a common causative element, and the findings help clarify potential causal links between genes or diseases within defined genomic regions. For genes significant in both cohorts, colocalization analysis regarding the risk of lipids and Apos was carried out with the R package coloc [25]. Loci featuring posterior probability of Hypothesis 4 (PP.H4) exceeding 0.8 were considered colocalized, and genes that were selected could be identified as candidate targets for drug development [26].

### 2.10. Phenome-Wide Association Analysis

To evaluate the possible horizontal pleiotropy between the genes of interest and negative effects, a phenome-wide association study (PheWAS) using the AstraZeneca PheWAS Portal (https://azphewas.com/) was conducted. Details of the construction process are available in the original study [27]. To minimize false positives, multiple adjustments were made with a threshold of 2e − 9.

### 2.11. Enrichment Analysis and Protein–Protein Interaction (PPI) Network Construction

Enrichment analyses for Gene Ontology (GO) and Kyoto Encyclopedia of Genes and Genomes (KEGG) were executed through the utilization of ClusterProfiler and Pathview R packages. GO consists of three fundamental categories: biological process (BP), molecular function (MF), and cellular component (CC). PPI networks can enhance our understanding of intracellular interaction mechanisms among proteins; after being constructed with STRING (https://cn.string-db.org/), they were analyzed using GeneMANIA (https://genemania.org/) [28].

### 2.12. Prediction of Natural Medicines

TCMBank (https://TCMBank.CN/) and the Drug Signatures Database were utilized to assess molecules of drug and identify natural medicine candidates [29]. Assessing interactions between proteins and drugs is essential to determine the therapeutic potential of target genes. TCMBank, a database offering standardized information on traditional Chinese medicines, contains 9192 herbs, 61,966 ingredients, and 15,179 target records, providing significant support for modern drug discovery [30]. DSigDB is an extensive data source that connects drugs and chemicals with their candidate genes. Genes were then uploaded into DSigDB to predict potential natural medicines and assess their pharmacodynamic effect.

### 2.13. Molecular Docking

To clarify the impact of potential therapeutic agents on druggable genes and their suitability for therapeutic applications, molecular docking was performed at the atomic level. Ligand files in the SDF (structure data file) format were retrieved from the PubChem database (https://pubchem.ncbi.nlm.nih.gov/). Molecular structure models of proteins were obtained from the UniProt database (https://www.uniprot.org/), and the docking was carried out with AutoDock Vina.

### 2.14. Molecular Dynamics Simulation

To assess structural stability, the complex was simulated for 100 ns using the molecular dynamics package GROMACS-2023. CHARMM36 and GAFF2 force fields were, respectively, employed to define the protein and ligand topologies. Electrostatic forces were calculated via the particle mesh Ewald (PME) method, while the Verlet algorithm was applied for short-range interactions. The process achieved equilibration for 100,000 steps in both the constant-temperature and constant-volume (NVT) and constant-pressure (NPT) ensembles. We set the coupling constant at 0.1 ps with a 100-ps total simulation time. Van der Waals and Coulomb interactions were computed with a 1.0 nm cutoff. Then, the system was simulated for 100 ns at 300 K [31].

### 2.15. Animal Experiments

C57BL/6J mice (20–25 g) were purchased from Zhejiang ZiYuan Experimental Animal Technology Co. Ltd. and maintained under specific pathogen-free (SPF) conditions with constant temperature. Approval for the experimental procedures was issued by the First Affiliated Hospital of Zhengzhou University's Ethics Committee (approval number: ZZU-LAC20241227 [16]). A high-fat diet (HFD) containing 60% fat, 20.6% carbohydrates, and 19.4% protein was used. Mice were assigned to four groups: a blank control group (Ctrl group, gavaged with saline), a HFD-fed group (HFD group, gavaged with saline), a HFD plus sanguinarine treatment group (HFD diet, gavaged with sanguinarine 5 mg/kg/day), and a HFD plus lycorine treatment group (HFD diet, gavaged with lycorine 15 mg/kg/day). The drug dosages used here were based on those used in previous studies, which demonstrated efficacy in achieving desired outcomes [32, 33]. The animals were euthanized after 6 weeks of feeding, and samples were isolated for analysis.

### 2.16. Determination of Serum Lipids and 25(OH)D Levels

Serum levels of TG, 25(OH)D, LDL-C, and HDL-C were assessed using commercially sourced assay kits: TG kit (Keweide, KWD-E9494M-B), 25(OH)D kit (Keweide, KWD-E11141M-B), LDL-C kit (Keweide, KWD-E10977M-B), and HDL-C kit (Keweide, KWD-E11502M-B). We collected blood samples from the retro-orbital sinuses of the mice, the collected samples were subsequently centrifuged at 3500 rpm (10 min), and the supernatant was obtained and then subjected to further processing for analysis.

### 2.17. Histological Analysis of Liver and Adipose

Upon euthanasia, livers of the mice were promptly collected and placed in 4% paraformaldehyde at 4°C to be fixed. Following that, paraffin wax embedding was performed on the tissues, which were then sectioned to a thickness of 5 *μ*m. Placing the sections of tissue onto slides, we stained them with hematoxylin and eosin (H&E) to perform a standard histological assessment. Sections were then treated with oil red O (HX-R, 14288-70-1) to visualize lipid accumulation using hematoxylin counterstaining. The adipose tissue sections were also stained with H&E.

### 2.18. Western Blot Analysis

Tissues obtained were homogenized in RIPA lysis buffer (Beyotime, P0013B), to which a 1% protease–phosphatase inhibitor cocktail (Beyotime, 78442) was added. The supernatant was obtained following centrifugation at 14,000 × g for a duration of 25 min, and the concentration of protein was measured. Primary antibodies, anti-chromobox 6 (CBX6) (1:1000, Affinity, AF0411), and anti-*β*-actin (1:1000, Abcam, ab8227) were incubated overnight, followed by 1-h incubation with secondary antibodies. Immunoblots were visualized using the BeyoECL Star chemiluminescence reagent kit and quantified using ImageJ software.

### 2.19. Statistical Analysis

The MR analysis was implemented with the use of R (Version 4.4.1). To take into consideration the complex multistage sampling design of NHANES and focus on patients with dyslipidemia, weighted analysis methods were employed. Weighted mean ± standard errors (SEs) were implemented for dealing with continuous variables, whereas unweighted frequencies were reported for categorical variables. Rao–Scott chi-square test or Kruskal–Wallis test was employed to contrast mortality outcomes and 25(OH)D subgroups. Weighted Cox regression analysis assessed the association between 25(OH)D and mortality of both all-cause and cardiovascular-cause, adjusting for various factors in three models: unadjusted, adjusted for sex and age, and adjusted additionally for demographic, lifestyle, and health factors. Kaplan–Meier curves and log-rank tests evaluated survival over time and compared the survival curves. Restricted cubic splines (RCSs) were applied to study the dose–response link between 25(OH)D and mortality, and the population of the study was classified into several groups in accordance with 25(OH)D inflection points for further analysis. Subgroup analysis was performed to explore differences across age, sex, hypertension, diabetes, and CVD subgroups. Sensitivity analysis, excluding participants with < 1 year of follow-up, was implemented to check the results' stability and reliability. When performing the in vivo experiments, we employed GraphPad Prism 8 software for analyzing the data, and one-way analysis of variance (ANOVA) was utilized to examine the differences between groups.

## 3. Result

### 3.1. Univariable MR Analyses of the Causal Association Between Lipid Traits, 25(OH)D, and CA

**Figure 2:**
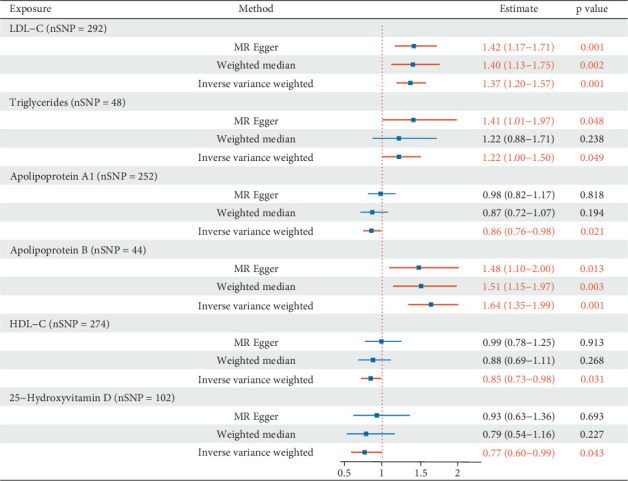
Results of Mendelian analysis presented by the forest map. Summary of the univariable MR analysis results for the association between lipid-related traits or vitamin D and the risk of cardiac arrest using the IVW, MR-Egger, and weighted median methods. nSNP, number of SNPs; CI, confidence interval; HDL-C, high-density lipoprotein cholesterol; LDL-C, low-density lipoprotein cholesterol; OR, odds ratio; SNP, single-nucleotide polymorphisms; TG, triglycerides.

Instrumental variables of lipid-related traits (HDL-C, LDL-C, TG, ApoA1, and ApoB) and 25(OH)D, as detailed in Supporting Information 4: Table S3, were selected after excluding SNPs associated with potential confounders (Supporting Information 4: Table S4). The final analyses included 292 SNPs for LDL-C, 48 for TG, 252 for ApoA1, 44 for ApoB, 274 for HDL-C, and 102 for 25(OH)D. Univariate forward MR analysis revealed potential associations between genetically proxied lipid traits, 25(OH)D, and CA. The analysis revealed correlations between increased LDL-C, TG, ApoB, and CA risk. For a one-standard deviation (SD) elevation in LDL-C, the odds ratio (OR) for CA risk was 1.40 (95% confidence interval (CI): 1.01–1.45; *p* < 0.001). For a one-SD increase in TG, the OR was 1.22 (95% CI: 1.00–1.50; *p* = 0.049). For a one-SD increase in ApoB, the OR was 1.64 (95% CI: 1.35–1.99; *p* < 0.001). Elevated ApoA1 and HDL-C levels correlated with decreased CA risk, with ORs (95% CI) of 0.86 (0.76–0.98; *p* = 0.021) and 0.85 (0.76–0.98; *p* = 0.031). An underlying connection was identified between increased 25(OH)D and a decreased risk of CA (OR = 0.77 (95% CI: 0.60–0.99]; *p* = 0.043) (Figure 2). These findings are consistent with the causal associations indicated by the weighted median and MR-Egger methodologies. The weighted median and MR-Egger methods further validated the causal inference. Heterogeneity was estimated using Cochran's *Q* value (*p* < 0.05).

When the *p* value was < 0.05, the primary analysis used the IVW method with a multiplicative random-effects model; otherwise, a fixed-effects IVW model was applied. Although signs of heterogeneity were observed (e.g., LDL-C and ApoB), there had no horizontal pleiotropy in most analyses, and the outcomes showed a consistent trend throughout the sensitivity analyses (Supporting Information 4: Table S5). A number of studies have indicated that the risk of CA may be influenced by sex [34], with sex-related hormones, such as testosterone levels, estradiol levels, and sex hormone-binding globulin (SHBG), contributing to this association [35, 36]. Given that the data in this study were summarized and do not allow for sex-stratified analyses, we further excluded SNPs associated with these hormone levels from the exposure variables and conducted MR analyses, and SNPs associated with sex hormone traits excluded from the exposures are detailed in Supporting Information 4: Table S6. Notably, the relationships of lipid traits and levels of 25(OH)D with CA continued to show significance after adjusting for potential confounding from sex hormone–related SNPs (*p* < 0.05; Supporting Information 2: Figure S2).

### 3.2. Univariable Bidirectional MR Analyses for the Causal Association Between Lipid Traits and Serum 25(OH)D

**Figure 3:**
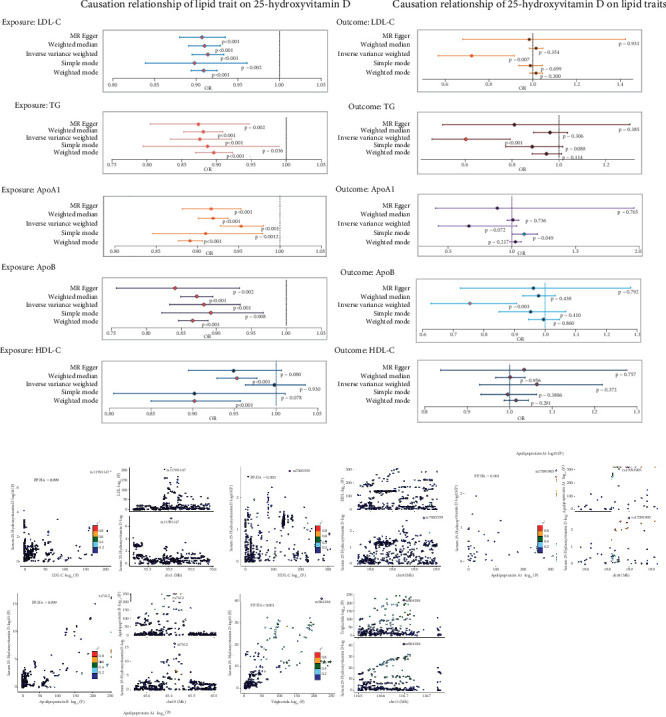
Causal estimates of bidirectional MR between lipid characteristics and vitamin D and colocalization analysis. (a) Causation relationship of lipid traits on 25-hydroxyvitamin D (left) and causation relationship of 25-hydroxyvitamin D on lipid traits (right). (b) Regional plot of colocalization evidence of lipid-related traits and vitamin D.

While lipid profiles are strongly linked to 25(OH)D, the causal relationship remains unclear [37, 38]. A bidirectional MR analysis was executed by us to determine whether there was a deterministic relationship between lipid traits and 25(OH)D. Univariate forward MR analyses showed that four lipid traits were associated with 25(OH)D. IVW multivariable regression analysis revealed that higher LDL-C (OR: 0.91, *p* < 0.001), TG (OR: 0.88, *p* < 0.001), ApoA1 (OR: 0.95, *p* = 0.012), and ApoB showed a relationship with a decreased likelihood of low serum 25(OH)D, with an OR of 0.499 (*p* = 0.001). This relationship received additional support from the MR-Egger, weighted median MR, and simple mode approaches, as their respective *p* values were less than 0.05. No causal relationship was detected between HDL-C and 25(OH)D levels (Figure 3). Sensitivity analysis showed that, with the exception of ApoA1, there was no indication of horizontal pleiotropy. A thorough assessment of horizontal pleiotropy and heterogeneity is provided in Supporting Information 4: Table S5. Reverse MR analyses demonstrated meaningful causal influence of 25(OH)D on three lipid-related traits: LDL-C (OR: 0.72, *p* = 0.01, IVW), TG (OR: 0.60, *p* < 0.001, IVW), and ApoB (OR: 0.75, *p* = 0.042, IVW). Simple mode analysis suggested a positive correlation between higher 25(OH)D and ApoA1 levels, although this association was not significant in the IVW analysis. Horizontal pleiotropy is described in Supporting Information 4: Tables S7–S8 based on the MR-Egger intercept test, and 25(OH)D showed no causal impact on HDL-C (OR: 1.06, *p* = 0.37, IVW). To further explore the relationship and potential mechanisms between lipid traits and levels of 25(OH)D, we executed Bayesian colocalization analysis to identify the common genetic loci, which presented a high enough posterior probability (PP4 > 0.8), indicating that ApoB and 25(OH)D share a single common variant (Supporting Information 4: Table S9). Consistent findings were also observed for the LDL-C and 25(OH)D levels (Figure 3).

### 3.3. Causal Impact of Genetically Predicted Size-Specific Lipoprotein Particles on CA Risk

**Figure 4:**
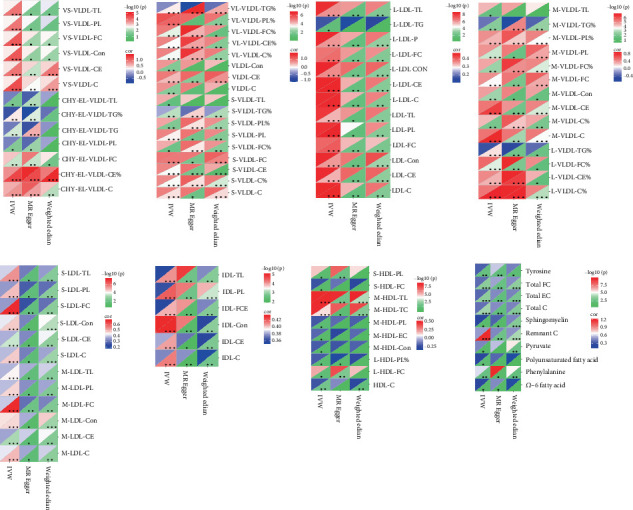
Associations of various lipoprotein particle fractions with risk of CA in the form of heatmaps. ⁣^∗^*p* < 0.05; ⁣^∗∗^*p* < 0.01; ⁣^∗∗∗^*p* < 0.001. C, cholesterol; CE, cholesterol ester; CON, concentration; HDL, high-density lipoprotein; IDL, intermediate density lipoprotein; L, large; LDL, low-density lipoprotein; M, medium; S, small; SVS, small vessel stroke; TG, triglycerides; TL, total lipids; VLDL, very low-density lipoprotein; X, extra; XL, extra large; XS, extra small.

To further explore the identified correlations, we applied genetic instruments to measure the association of cholesterol (CE), TG, and lipid profiles across size-categorized lipoprotein particles, with CA risk. Individuals genetically predisposed to elevated cholesterol levels in both small and large very low-density lipoprotein (VLDL) subfractions are more likely to experience CA, as illustrated in Figure 4. Similarly, a genetic predisposition to higher TG levels in intermediate density lipoprotein (IDL) and medium/large low-density lipoprotein corpuscles is related to an elevated risk of experiencing CA. Finally, lower cholesterol and cholesterol ester levels in medium and large HDL corpuscles determined by genetics were connected to an elevated risk of experiencing CA. In addition to lipoprotein particle characteristics, we also investigated the genetic causal effects of traits linked to lipid metabolism on outcomes and discovered that lower genetically predicted levels of phenylalanine, polyunsaturated fatty acid, pyruvate, remnant C, omega-6 fatty acid, sphingomyelin, total C, total EC, total FC, and tyrosine, as well as higher lactate contribute to an increased CA risk. In summary, through UVMR analyses, it was revealed that the risk of CA was causally influenced by size-defined lipoprotein fractions as a result of genetic factors (Supporting Information 4: Table S10).

### 3.4. UVMR and MVMR Analyses for the Causal Impact of Lipid-Related Traits or 25(OH)D Levels on Potential Mediators

**Figure 5:**
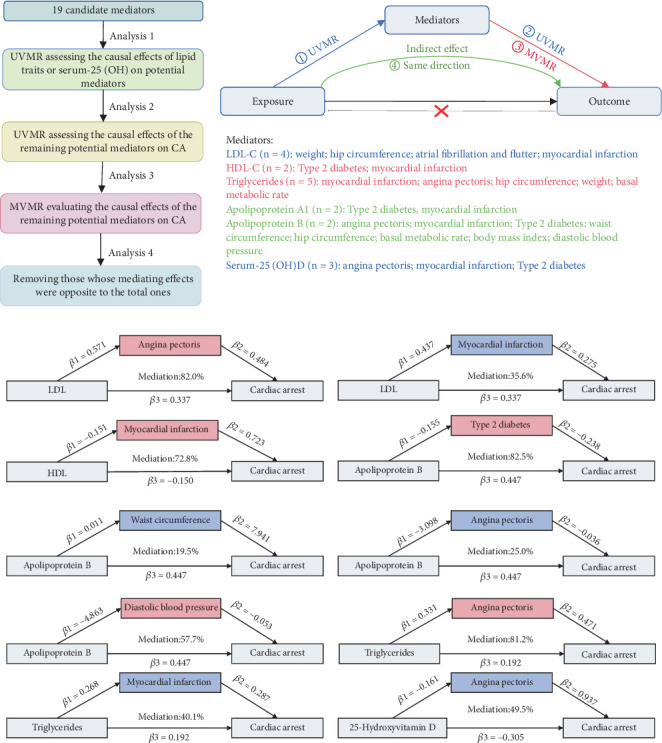
Screening process for mediators in the causal correlation and mediating effect of selected traits on the associations of lipid or vitamin D with risk of CA. (a) Four analytical criteria were performed to select mediators in the causal pathway from lipid traits or serum 25(OH) on potential mediators to CA: (1) causal association of lipid traits or um-25(OH) on potential mediators, (2) mediator's causal effect on the outcome via UVMR, (3) direct causal effect of mediators on the occurrence of CA from MVMR, and (4) removing those whose mediating effects were opposite to the total ones. (b) Mediating effect of selected traits on the associations of lipid or vitamin D with the risk of CA, MR estimates for the mediating effect of each mediator, and the proportion mediated by each mediator. “Total effect (*β*3)” indicates the effect of exposures on the risk of CA, “direct effect (*β*1)” indicates the effect of exposures on each mediator, “direct effect (*β*2)” indicates the effect of each mediator on CA, and “mediation effect” indicates the effect of exposure on the risk of CA via each mediator. Total effect (*β*3) and direct effect (*β*1) were derived by the IVW method in UVMR; direct effect B (*β*2) was from the IVW method in MVMR.

Based on MR analysis, 19 potential mediators were identified, and their mediation proportions, along with 95% CIs, were calculated. A flowchart illustrating the analytical workflow is shown in Figure 5a. We first conducted UVMR analyses for these mediators, with the results summarized in Supporting Information 4: Table S11. Any mediators displaying nonsignificant associations (*p* > 0.05) or signs of pleiotropy were excluded from the study. The remaining candidates were then subjected to MVMR. Variables with *p* values < 0.05 in the MVMR analysis were identified as mediators and included in the subsequent analysis (Supporting Information 4: Table S12). Finally, six factors were identified as the mediators which are linked to CA risk (Supporting Information 4: Table S13). Two mediators were identified in the correlation between LDL-C and CA risk: AP (mediation proportion: 82.1%) and MI (35.6%). The association between ApoB and CA risk involved four mediators: Type 2 diabetes (82.7%), WC (19.9%), AP (25.2%), and DBP (57.9%). Regarding the association between TG and CA risk, AP (81.4%) and MI (40.2%) were identified as mediators. For 25(OH)D status and CA risk, AP was identified as a mediator modulating the risk of CA, with a mediation proportion of 49.3% (Figure 5b).

### 3.5. 25(OH)D and Mortality in Dyslipidemia (NHANES)

**Figure 6:**
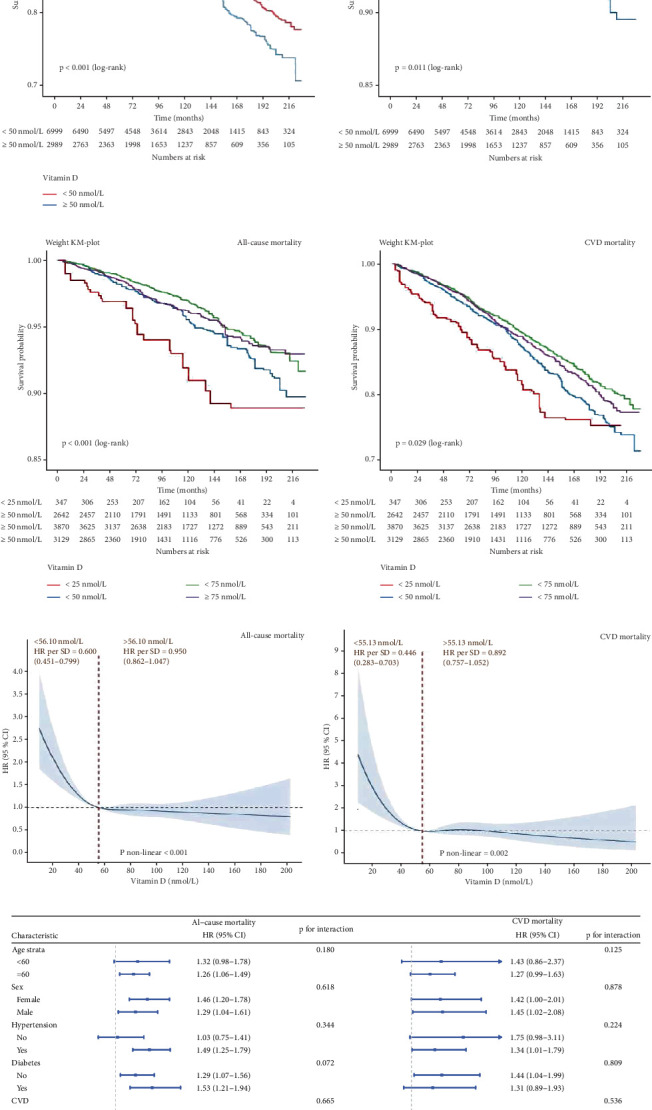
Relationship between serum 25(OH)D levels and mortality outcomes in patients with dyslipidemia from NHANES. (a, b) The Kaplan–Meier survival curve and data on survival outcomes for all-cause mortality and CVD mortality by serum vitamin D levels in patients with dyslipidemia. (c) Restricted cubic splines (RCSs) for nonlinear relationship analysis between 25(OH)D concentration and both all-cause mortality and cardiovascular disease (CVD) mortality in patients with dyslipidemia. (d) Subgroup and sensitivity analyses demonstrated that the survival advantage of high 25(OH)D concentration relative to low 25(OH)D concentration in dyslipidemia patients was consistent across subgroup analyses stratified by age, sex, history of hypertension, diabetes, and cardiovascular disease.

We additionally investigated the relationship among dyslipidemia, 25(OH)D status, and mortality outcomes using data from the NHANES database. Among the 9988 patients with dyslipidemia, 25(OH)D deficiency (< 50.00 nmol/L) and insufficiency (< 75.00 nmol/L) were prevalent in 23.83% and 63.71% of the participants, respectively. The baseline features of the study cohort and the grouping information are described in detail in Supporting Information 4: Tables S14–S15. When 25(OH)D was insufficient or in a deficient state, the mortality from all causes and cardiovascular events increased (Supporting Information 4: Table S9). The survival curve analysis further demonstrated this relationship. According to the log-rank test, individuals with heightened 25(OH)D exhibited better survival outcomes, with *p* values < 0.05 (Figure 6a,b). During the total follow-up duration of 88,501 person-years, among 1554 deaths, 540 were CVD-related. Following multivariable adjustment including age and sex (Model 3), the multivariate-adjusted hazard ratios (HRs) and 95% CIs for 25(OH)D categories (< 25.00, 25.00–49.99, 50.00–74.99, and ≥ 75.00 nmol/L) were as follows: all-cause mortality: 1.000 (reference), 0.596 (0.408–0.870), 0.280 (0.185–0.425), and 0.453 (0.303–0.676), respectively; cardiovascular mortality: 1.000 (reference), 0.481 (0.265–0.872), 0.195 (0.108–0.352), and 0.298 (0.167–0.530), respectively (Supporting Information 4: Table S16).

Using RCS for nonlinear relationship analysis, an L-shaped nonlinear association between 25(OH)D concentration and all-cause and CVD mortality was found (Figures 6a, 6b, and 6c). When 25(OH)D concentration was < 56.10 or 55.13 nmol/L, an increase of one SD in 25(OH)D was connected to the HRs of all-cause and CVD mortality of 0.600 (95% CI: 0.451–0.799) and 0.446 (95% CI: 0.283–0.703), respectively. Once the 25(OH)D concentration surpassed 56.10 or 55.13 nmol/L, no significant link was found between 25(OH)D and CVD or all-cause mortality. Subgroup and sensitivity analyses demonstrated that the survival advantage of high 25(OH)D (≥ 50 nmol/L) relative to low 25(OH)D (< 50 nmol/L) for individuals diagnosed with dyslipidemia was consistent across subgroup analyses stratified by age, sex, diabetes, history of hypertension, and CVD (Figure 6d). No notable significant interaction existed between 25(OH)D and the stratified factors. Additionally, the study indicates a more pronounced negative correlation between 25(OH)D levels and all-cause mortality among older adults (≥ 60 years), women, and individuals with hypertensive history and diabetes, although the interaction tests were not significant. The sensitivity analysis results also further confirmed the robustness of our study (Supporting Information 4: Table S17).

### 3.6. Causal Associations: Genes, Lipids, 25(OH)D, and CA Risk

**Figure 7:**
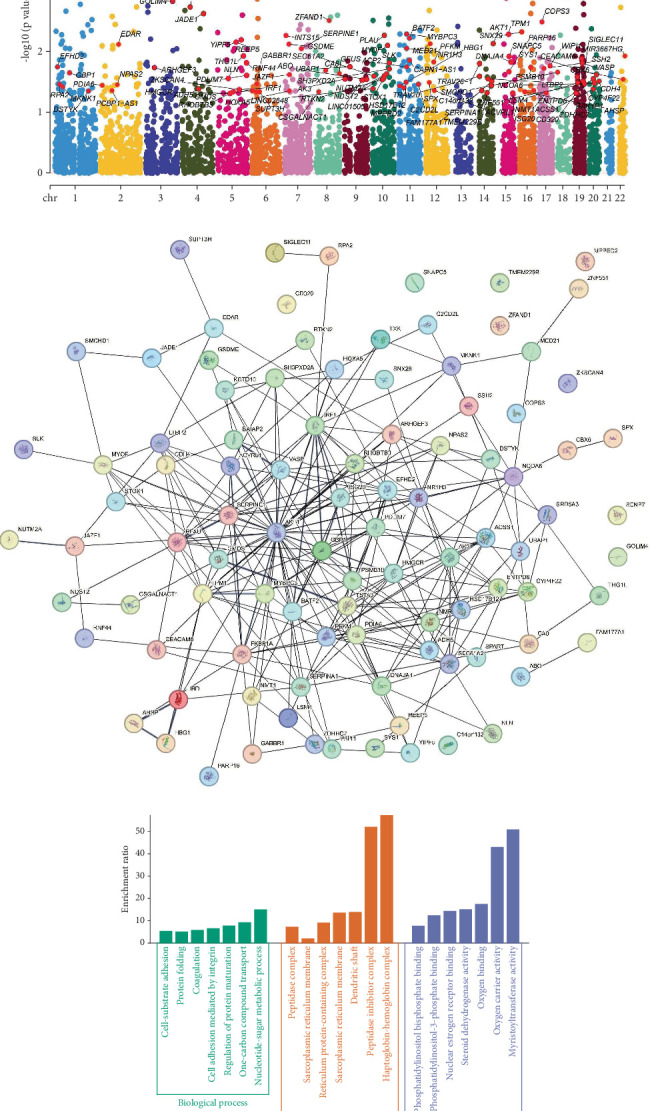
Genes causally significantly associated with lipid traits, 25(OH)D levels, and CA risk. (a) Venn diagram displayed the intersection of identified genes both associated with lipid traits and CA risk after the MR analysis of eQTL data. (b) Manhattan plot for correlation of interacting genes with CA in MR analysis. (c) The protein–protein interaction (PPI) network demonstrated the interactions among the intersection genes. (d) GO enrichment of the intersection genes. (e) KEGG enrichment to explore the functional roles and pathways of these genes.

In the discovery phase, we identified 275 genes that were markedly related to CA risk and 675 genes that were causally closely correlated with LDL-C (Figure 7). Considering the intersection of these gene sets, we found 35 genes associated with both CA risk and LDL-C. We performed similar intersection analyses for CA risk with HDL-C (38 genes), HDL-C (38 genes), ApoA1 (26 genes), TG (21 genes), ApoB (17 genes), and 25(OH)D (23 genes) levels. Ultimately, 67 genes linked to CA risk were obtained, and the results of the MR analysis are visualized in a Manhattan plot, which is presented in Figure 7. The obtained results were uploaded to the STRING website in order to make a PPI network that illustrated the interactions between these genes (Figure 7). GO enrichment analysis (focusing on gene-term interactions) and KEGG enrichment analysis (emphasizing gene-pathway relationships) were performed for the chosen genes. These genes were primarily involved in BPs such as “nucleotide-sugar metabolic process,” “protein folding,” and “regulation of protein maturation.” They were also associated with CCs like the “haptoglobin–hemoglobin complex” and “peptidase inhibitor complex.” Furthermore, their primary MFs included “myristoyltransferase activity” and “oxygen carrier activity,” among others (Figure 7). Those genes were enriched in the “fructose and mannose metabolism” and “biosynthesis of nucleotide sugars” pathways, among others (Figure 7). Previous studies also indicated that these pathways are involved in regulating lipid traits, CVD, diabetes, and other metabolic diseases [39, 40].

### 3.7. Candidate Drug Prediction

**Figure 8:**
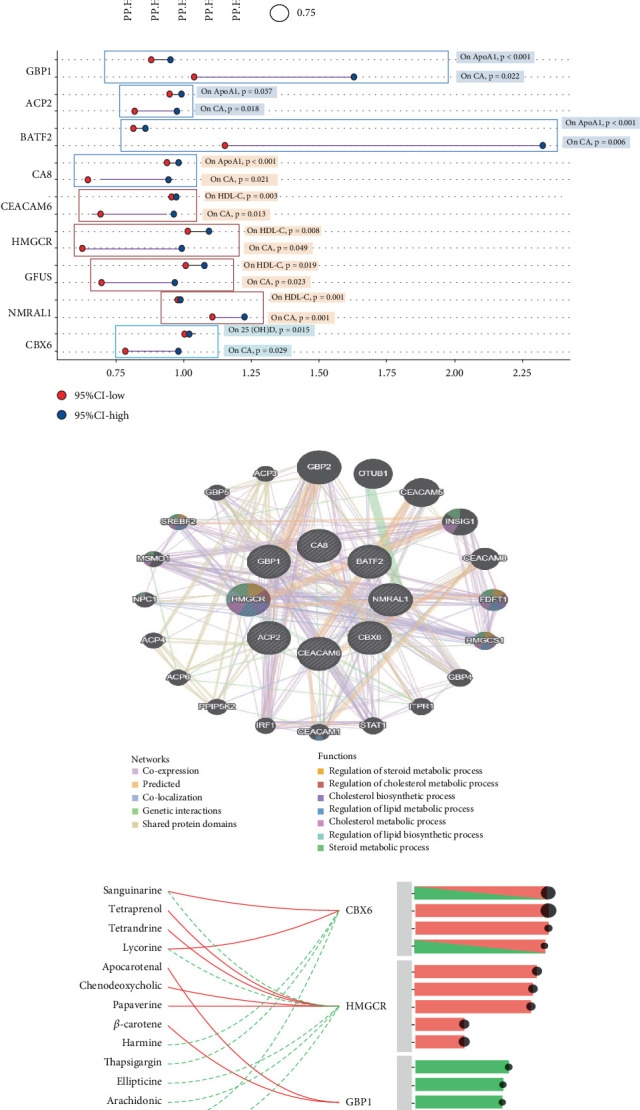
Druggable gene identification and natural molecular drug screening. (a) Colocalization analysis demonstrated compelling proof of colocalization. (b) Genetic variant causal effects on associated traits and CA risk. (c) PPI network built with GeneMANIA. Each circle is colored to indicate the functional pathway in which each gene is involved. (d) Screening of 14 natural drugs from TCMBank and DSigDB.

To address the potential for false-positive MR results due to LD, a colocalization analysis of the 67 genes identified in the discovery phase was performed. Prior research has indicated that proteins identified through both MR and colocalization-related analyses demonstrate enhanced capability for drug target validation and approval. The analysis exhibited substantial conclusive evidence of colocalization (PP.H4 > 0.8) among five proteins: basic leucine zipper transcription factor, ATF-like 2 (BATF2), carbonic anhydrase 8 (CA8), lysosomal acid phosphatase 2 (ACP2), and guanylate binding protein 1 (GBP1), related to ApoA1; four other proteins, including CEA cell adhesion molecule 6 (CEACAM6), HMG-CoA reductase (HMGCR), GDP-L-fucose synthase (GFUS), and CBX6, related to HDL-C; and one protein, NMRAL1, with respect to serum 25(OH)D. These proteins are potential candidate drug target genes (Figure 8a and Supporting Information 4: Table S18). To additionally clarify the likely pleiotropy existing in the genes and the potential negative consequences, a PheWAS analysis for nine genes was implemented (Supporting Information 3: Figure S3).  Figure 8b illustrates the estimated causal influences that genetic variants exert on the associated characteristics, showing a strong inverse correlation between GBP1, BATF2, and ApoA1, while both GBP1 and BATF2 were positively correlated with the risk of CA. HMGCR exhibited a positive connection with HDL-C level and a powerful negative link with CA risk. NmrA-like family domain-containing protein (NMRAL1) expression negatively correlated with 25(OH)D while positively correlated with CA risk (Supporting Information 4: Table S19). We further used GeneMANIA to construct a PPI network that included these nine drug targets and 20 additional genes that potentially interact, resulting in 868 interaction links (Figure 8c). Functional assessment of the network brings to light the significances of these drug targets and related genes, indicating their involvement in “regulation of lipid biosynthetic process,” “regulation of lipid metabolic process,” and “steroid metabolic process,” which is consistent with our prior hypothesis (Figure 8c). The TCMBank and DSigDB databases were used to predict potentially effective natural intervention drugs. Finally, 14 natural drugs were screened from both TCMBank and DSigDB, based on adjusted *p* values < 0.05, and eight natural drugs (sanguinarine, tetraprenol, tetrandrine, lycorine, chenodeoxycholic, papaverine, harmine, and thapsigargin) were ultimately identified as potentially interacting with three proteins: CBX6, HMGCR, and GBP1 (Figure 8d, Supporting Information 4: Table S20).

### 3.8. Molecular Docking and Dynamics Simulation

**Figure 9:**
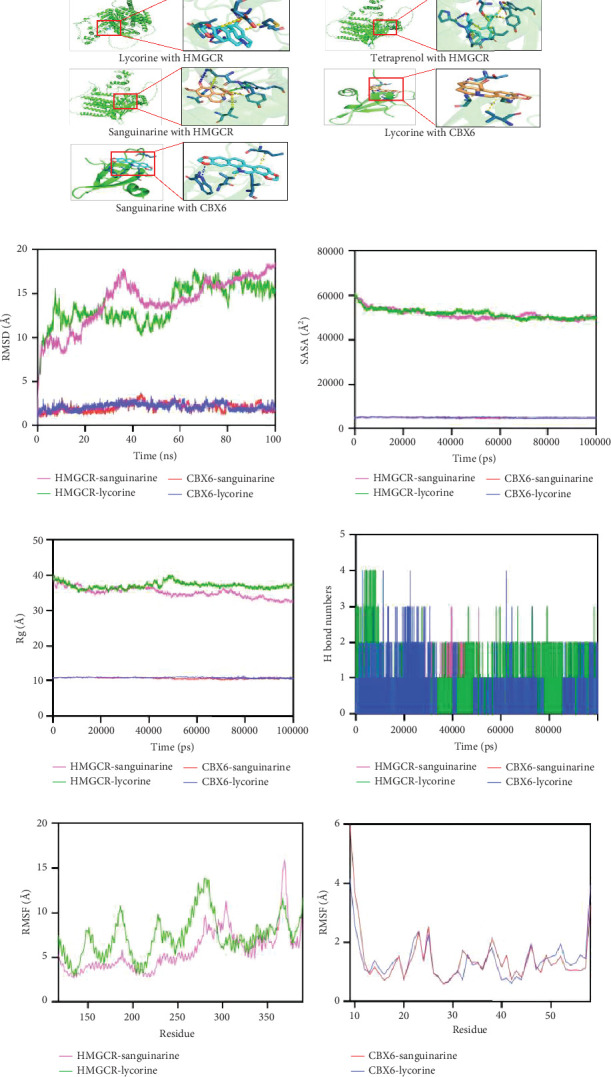
Molecular docking and molecular dynamics simulation. (a) Docking results of available proteins small molecules. (b) RMSD for simulation equilibration assessment. (c, d) The radius of gyration (Rg) and solvent accessible surface area (SASA) of the composite system of CBX6-sanguinarine and CBX6-lycorine fluctuate stably during motion. (e) Number of hydrogen bonds formed between small molecules and the target protein in dynamics. (f, g) Root mean square fluctuation (RMSF) showed the flexibility of amino acid residues in a protein.

Valid docking results were obtained for two proteins and their interacting drugs with the application of AutoDock Vina (Figure 9a). The analysis revealed that each drug candidate formed stable complexes with its target protein, which were characterized by prominent hydrogen bonding and strong electrostatic interactions. The drug candidates effectively occupied the binding pockets of their respective targets. Sanguinarine and lycorine both demonstrated strong binding to HMGCR, with binding energies of −7.4 and −7.2 kcal/mol, suggesting highly stable interactions. Furthermore, interactions between these two drugs and CBX1 were also observed at lower binding energies (sanguinarine and CBX6, −6.3 kcal/mol; lycorine and CBX6, −5.7 kcal/mol) (Supporting Information 4: Table S21). Therefore, these two drugs should be the primary focus of future studies. The root mean square deviation (RMSD), which reflects the atomic positional deviations from their initial states, was used to assess simulation equilibration. Lower RMSD values indicate greater conformational stability. As shown in Figure 9b, the equilibrium was attained in the HMGCR-sanguinarine and HMGCR-lycorine complexes after 95 and 60 ns, respectively. Equilibration of the CBX6-sanguinarine and CBX6-lycorine complexes was observed at approximately 10 ns, followed by stable RMSD oscillations around 2.8 Å. The solvent accessible surface area (SASA) and radius of gyration (Rg) of the composite system of CBX6-sanguinarine and CBX6-lycorine fluctuated stably during motion. Notably, sanguinarine and lycorine small molecules demonstrated enhanced stability upon binding to the CBX6 target protein (Figure 9c,d).  Figure 9e illustrates the fluctuations in hydrogen bond formation between the small molecules and target protein over the dynamic process. Hydrogen bond formation in the CBX6-sanguinarine and CBX6-lycorine systems varied from 0 to 4 throughout the simulation, indicating favorable hydrogen bonding interactions. Protein residue flexibility can be assessed through analysis of root mean square fluctuation (RMSF). The RMSF values of the CBX6-sanguinarine and CBX6-lycorine complex systems were relatively low, suggesting lower flexibility and higher stability. In summary, the CBX6-sanguinarine and CBX6-lycorine complex systems exhibited stable binding and favorable hydrogen bonding interactions (Figure 9f,g). These findings indicate that sanguinarine and lycorine small molecules effectively bind to the CBX6 target protein.

### 3.9. Sanguinarine and Lycorine Ameliorate Diet-Induced Hyperlipidemia, Hepatic Steatosis, and 25(OH)D Deficiency in Mice

**Figure 10:**
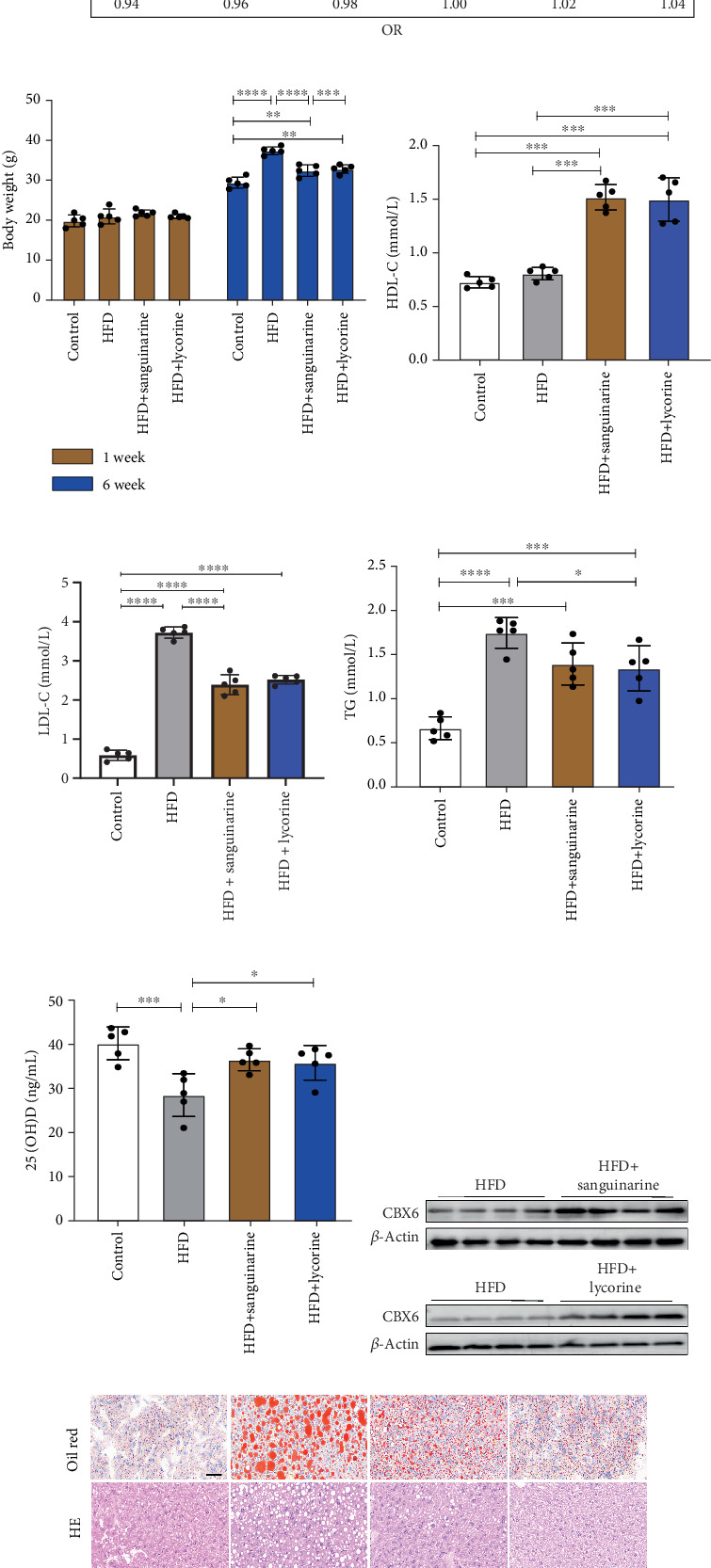
Sanguinarine and lycorine alleviate mouse hyperlipidemia and vitamin D deficiency. (a) MR analysis show the causation relationship of gene CBX6 on lipid and vitamin D. (b) Weight changes of mice before and after feeding with different diet combinations. (c–f) The effect of lycorine on serum HDL-C, LDL-C, TG, and vitamin D levels. (g) Western blot analysis demonstrated the protein expression levels of CBX6 in the liver tissues of mice in different groups. (h) Oil red staining and HE staining in liver of different groups, bar = 20* μ*m. Error bars are represented as mean ± SEM. ⁣^∗^*p* < 0.05, ⁣^∗∗^*p* < 0.01, and ⁣^∗∗∗^*p* < 0.001.

In subsequent animal experiments, we analyzed the effects of sanguinarine and lycorine on lipid metabolism and 25(OH)D levels in mice. We first investigated the impacts of CBX6 on HDL-C, LDL-C, TG, and 25(OH)D using MR analysis. The results showed that CBX6 promoted HDL-C expression and reduced TG levels. CBX6 showed no significant impact on 25(OH)D or LDL-C levels (Figure 10). After feeding the mice different diets for 6 weeks, we assessed the weight changes that occurred in the mice of different groups; the weight in the HFD group of mice increased significantly, whereas that of mice treated with sanguinarine and lycorine showed a lower increase, suggesting that sanguinarine and lycorine have an inhibitory effect on obesity (Figure 10). Subsequently, HDL-C, TG, LDL-C, and 25(OH)D were measured and found that both sanguinarine and lycorine alleviated and improved blood lipid and 25(OH)D metabolism, including increasing HDL-C and 25(OH)D while reducing TG and LDL-C (Figures 10, 10, 10, and 10). Through western blot analysis, we demonstrated that sanguinarine and lycorine promoted the expression of CBX6 within the liver tissue and reduced lipid accumulation (Figure 10). This showed that sanguinarine and lycorine regulate the expression of CBX6 in vivo, thereby improving the metabolism of lipids and 25(OH)D. Although analysis of MR did not reveal any effects, in vivo experiments showed that sanguinarine and lycorine regulated 25(OH)D and LDL-C. This also suggests a complex regulatory mechanism between 25(OH)D and lipids in vivo, and that the two influence each other.

## 4. Discussion

Through a large-scale and comprehensive MR analysis, this study confirmed a causal link between dyslipidemia, 25(OH)D, and CA risk. We also found that lipid traits and 25(OH)D levels have a mutual influence and that there is a bidirectional causal relationship between LDL-C, TG, ApoB, and 25(OH)D. Our results strongly indicated that they are jointly involved in the occurrence and progression of CA. Subsequent analysis identified key mediators such as diabetes and AP and estimated the mediating effects and their relative contributions in the pathway leading to the occurrence of CA. The NHANES data confirmed that low levels of 25(OH)D lead to higher all-cause and cardiovascular mortality rates in individuals with dyslipidemia, further demonstrating the reliability of the MR analysis. Finally, we focused on these risk factors to identify the corresponding drug targets that can prevent CA. We successfully identified natural drug molecules that target the crucial gene CBX6 and subsequently performed in vivo validation, demonstrating their potential to enhance lipid profiles and 25(OH)D, thereby contributing to CA prevention.

Dyslipidemia and 25(OH)D deficiency are major global health concerns with increasing prevalence. These conditions are associated with various comorbidities, including diabetes as well as other chronic diseases. While MR studies showed a causal link between 25(OH)D and dyslipidemia in these diseases, their relationship with CA remains unclear [41–44]. Observational studies have revealed a potential connection between lipid-related traits and CA. For example, investigations have revealed a connection between the fatty liver index and sudden CA in young adults [45] and a correlation between LDL-C and sudden CA in individuals with diabetes mellitus [6]. Studies have also indicated that lipid profiles are associated with neurological outcomes in patients with CA [46]. Additionally, studies have shown that 25(OH)D is closely intertwined with CA risk and the probability of succumbing to cardiovascular mortality [47]. The association that exists between 25(OH)D deficiency and neurological outcomes after cardiopulmonary resuscitation was previously reported [48]. However, our analysis focused on exploring the links among ApoA1, ApoB, 25(OH)D, and CA risk. Our findings indicate that elevated levels of HDL-C, ApoA1, and 25(OH)D are closely related to lower CA risk, and increased LDL-C, ApoB, and TG enhance CA occurrence. These findings improved understanding of CA risk and potential approaches for preventing CA. Additionally, our MR analysis revealed an inverse causal link between genetically based 25(OH)D status and TG and LDL-C, corroborating previous research [49]. Furthermore, colocalization analysis revealed shared genetic susceptibility factors among LDL-C, ApoB, and 25(OH)D, strongly indicating that they jointly impact the occurrence and subsequent clinical progress of CA.

In this study, we included a comprehensive list of candidate mediators (*n* = 19), including metabolic elements and lifestyle. Unusually, AP and MI were considered significant (with a mediating effect ranging from 25.2% to 82.1%) because almost all exposure factors can influence CA risk by affecting these two conditions. Reducing the occurrence rate of coronary heart disease is an effective strategy for preventing CA, which is consistent with current research reports [50]. Notably, we further detected the correlation between 25(OH)D and mortality in patients with dyslipidemia using the NHANES database (2001–2018), presenting additional corroboration of our conclusions. Various studies have demonstrated a significant correlation between serum 25(OH)D and total mortality among populations with different features. However, studies focusing on hyperlipidemic populations remain limited. In contrast to existing research, our analysis, which included a larger population sample with a wider age range from the NHANES data (2001–2018), provided sufficient statistical strength to reveal the role of vitamin D and dyslipidemia in reducing the mortality rates. This robust sample size enhanced the representativeness and reliability of our findings.

To find strategies for preventing CA, we initially identified the genes associated with lipid metabolic disorders and 25(OH)D metabolism, explored the relationship existing among these genes and CA, and identified nine genes that contribute substantially to regulating lipid and sterol biosynthesis. Herbal medicine is crucial in the prevention and therapy of long-term diseases such as obesity and diabetes. Natural remedies, derived from a wide array of plants, offer a viable option for therapeutic interventions. By integrating a natural Chinese medicine database with in vivo experimental validation, this study revealed a potential mechanism that accounts for the beneficial effects of specific herbal compounds. Molecular docking analysis revealed that sanguinarine and lycorine exhibited the highest binding affinities for CBX6, a key regulator of lipid metabolism. This finding, coupled with their ability to effectively reduce LDL-C and TG levels while concurrently elevating HDL-C and 25(OH)D, suggests their possible value as therapeutic tools for preventing CA. Several studies have demonstrated that sanguinarine modulates lipid metabolism and induces antiplatelet aggregation [51, 52]. Lycorine plays a regulatory role in lipid metabolism and helps mitigate structural cardiac changes and inflammation caused by angiotensin II [53, 54]. Our analysis reveals that CBX6 is a novel therapeutic target for preventing CA, further highlighting the potential of herbal medicines in addressing multiple aspects of cardiovascular health.

The analyses presented herein have several advantages. First, we use MR to investigate the relationship among lipid traits, 25(OH)D, and CA risk. Second, we employed NHANES data to further elucidate the relationship of 25(OH)D with all-cause and cardiovascular mortality within the dyslipidemic population. Furthermore, we identified two natural drugs and verified through in vivo experiments that they significantly improve lipid profiles and enhance 25(OH)D levels, potentially serving as therapeutic strategies for preventing CA. Although the reported findings are notable, this study has certain limitations. First, although MR analyses suggested that key genes may play beneficial roles in preventing CA and improving lipid profiles and vitamin D levels, our animal experiments only confirmed the effects of the candidate drugs on lipid metabolism and vitamin D status. Whether these drugs can actually reduce the risk of CA was not assessed in the animal models. Second, as our MR analyses were conducted exclusively in those with European heritage, the extent to which these findings hold true for other populations remains uncertain and requires further investigation.

## Data Availability

Data is available on request from the authors.

## Supporting Information

Supporting Information 1 Figure S1: Three significant assumptions for Mendelian randomization.

Supporting Information 2 Figure S2: Forest plot of Mendelian randomization results after excluding SNPs associated with testosterone, SHBG, and estradiol levels.

Supporting Information 3 Figure S3: Results of PheWAS analysis for nine genes.

Supporting Information 4 Table S1: STROBE-MR checklist of recommended items to address in reports of Mendelian randomization studies. Table S2: Detailed information on used GWAS studies. Table S3: Genetic variants used as instrumental variables in univariable MR. Table S4: Confounding factors and the SNPs associated with lipid traits and 25(OH)D. Table S5: Cochran *Q* test result and MR-Egger intercept in univariable MR. Table S6: Sex-related SNPs among lipid traits and 25(OH)D. Table S7: Cochran *Q* test result and MR-Egger intercept in univariable MR outcome: serum 25(OH)D. Table S8: Cochran *Q* test result and MR-Egger intercept in univariable MR exposure: serum 25(OH)D. Table S9: Results of Bayesian tests for colocalization. Table S10: The results of MR-radial method of size-defined lipoprotein particle fractions on CA risk. Table S11: Analyses for the causal impact of lipids and 25(OH)D on mediators. Table S12: MVMR analyses for the causal impact of potential mediators on CA. Table S13: Results of Mendelian mediation analysis. Table S14: Baseline characteristics of the study population. Table S15: Baseline characteristics of the study population. Table S16: HRs (95% CIs) for all-cause mortality and CVD mortality according to vitamin D concentrations. Table S17: Sensitivity analysis of the association of the vitamin D concentrations with all-cause mortality and CVD mortality. Table S18: colocalization results of eQTLs for nine genes with apolipoprotein A1, HDL, and serum 25-hydroxyvitamin D associated SNPs. Table S19: The impact of druggable genes on lipid traits, vitamin D, and the risk of cardiac arrest. Table S20: Candidate drug predicted using DSigDB and TCMSP. Table S21: Docking results of available proteins with small molecules.

## References

[1] Vazquez A. R., Sudhir A. (2023). Cardiac Arrest as a Public Health Issue. *Emergency Medicine Clinics of North America*.

[2] Chiu P. Y., Chung C. C., Tu Y. K., Tseng C. H., Kuan Y. C. (2023). Therapeutic Hypothermia in Patients After Cardiac Arrest: A Systematic Review and Meta-Analysis of Randomized Controlled Trials. *The American Journal of Emergency Medicine*.

[3] Kim Y. G., Roh S. Y., Han K. D. (2022). Hypertension and Diabetes Including Their Earlier Stage Are Associated With Increased Risk of Sudden Cardiac Arrest. *Scientific Reports*.

[4] Kim Y. G., Han K., Jeong J. H. (2022). Metabolic Syndrome, Gamma-Glutamyl Transferase, and Risk of Sudden Cardiac Death. *Journal of Clinical Medicine*.

[5] Oliveira M. A., Faerstein E., Koury J. C. (2021). Vitamin D Is Directly Associated With Favorable Glycemic, Lipid, and Inflammatory Profiles in Individuals With at Least One Component of Metabolic Syndrome Irrespective of Total Adiposity: Pró-Saúde Study, Brazil. *Nutrition Research*.

[6] Melguizo-Rodríguez L., Costela-Ruiz V. J., García-Recio E., De Luna-Bertos E., Ruiz C., Illescas-Montes R. (2021). Role of Vitamin D in the Metabolic Syndrome. *Nutrients*.

[7] Lee M. J., Jung H., Shin S. D. (2024). Vitamin D Deficiency as a Risk Factor for Sudden Cardiac Arrest: A Multicenter Case-Control Study. *Nutrition, Metabolism, and Cardiovascular Diseases*.

[8] Kim Y. G., Jeong J. H., Han K. D. (2023). Association Between Low-Density Lipoprotein Cholesterol and Sudden Cardiac Arrest in People With Diabetes Mellitus. *Cardiovascular Diabetology*.

[9] Jiang X., Peng M., Chen S., Wu S., Zhang W. (2019). Vitamin D Deficiency Is Associated With Dyslipidemia: A Cross-Sectional Study in 3788 Subjects. *Current Medical Research and Opinion*.

[10] Skrivankova V. W., Richmond R. C., Woolf B. A. R. (2021). Strengthening the Reporting of Observational Studies in Epidemiology Using Mendelian Randomization: The Strobe-Mr Statement. *JAMA*.

[11] Zimmerman D. S., Tan H. L. (2021). Epidemiology and Risk Factors of Sudden Cardiac Arrest. *Current Opinion in Critical Care*.

[12] Kim Y. G., Jeong J. H., Roh S. Y. (2023). Obesity Is Indirectly Associated With Sudden Cardiac Arrest Through Various Risk Factors. *Journal of Clinical Medicine*.

[13] Dimsdale J. E. (2023). Sudden Cardiac Death and Schizophrenia. *JACC: Clinical Electrophysiology*.

[14] Burgess S., Davey Smith G., Davies N. M. (2019). Guidelines for Performing Mendelian Randomization Investigations: Update for Summer 2023. *Wellcome Open Research*.

[15] Mazidi M., Katsiki N., Shekoohi N., Banach M. (2020). Monounsaturated Fatty Acid Levels May Not Affect Cardiovascular Events: Results From a Mendelian Randomization Analysis. *Frontiers in Nutrition*.

[16] Borghi C., Rodriguez-Artalejo F., De Backer G. (2016). The Association Between Blood Pressure and Lipid Levels in Europe: European Study on Cardiovascular Risk Prevention and Management in Usual Daily Practice. *Journal of Hypertension*.

[17] Taylor R., Barnes A. C., Hollingsworth K. G. (2023). Aetiology of Type 2 Diabetes in People With A 'Normal' Body Mass Index: Testing the Personal Fat Threshold Hypothesis. *Clinical Science (London, England)*.

[18] Monga N., Sethi G. S., Kondepudi K. K., Naura A. S. (2020). Lipid Mediators and Asthma: Scope of Therapeutics. *Biochemical Pharmacology*.

[19] Burkart K. M., Manichaikul A., Wilk J. B. (2014). Apom and High-Density Lipoprotein Cholesterol Are Associated With Lung Function and Per Cent Emphysema. *The European Respiratory Journal*.

[20] Zhang Y., Lin S., Li J., Song X., Chen G., Pei L. (2022). Interaction of Passive Smoking and Diet Habits on Vitamin D Deficiency Among Women of Reproductive Age in Rural Central China. *Nutrients*.

[21] Erensoy H. (2019). The Association Between Anxiety and Depression With 25(Oh)D and Thyroid Stimulating Hormone Levels. *Neurosciences (Riyadh)*.

[22] Pohl S. B., Engelbertz C., Reinecke H. (2024). Unused Potential of Lipid-Lowering Therapy in Very High-Risk Patients With Atherosclerotic Cardiovascular Disease. A Retrospective Data Analysis. *Nutrition, Metabolism, and Cardiovascular Diseases*.

[23] Holick M. F., Binkley N. C., Bischoff-Ferrari H. A. (2011). Evaluation, Treatment, and Prevention of Vitamin D Deficiency: An Endocrine Society Clinical Practice Guideline. *The Journal of Clinical Endocrinology and Metabolism*.

[24] Ross A. C., Manson J. E., Abrams S. A. (2011). The 2011 Report on Dietary Reference Intakes for Calcium and Vitamin D From the Institute of Medicine: What Clinicians Need to Know. *Journal of Clinical Endocrinology and Metabolism*.

[25] Giambartolomei C., Vukcevic D., Schadt E. E. (2014). Bayesian Test for Colocalisation Between Pairs of Genetic Association Studies Using Summary Statistics. *PLoS Genetics*.

[26] Feng K., Yang J., Liu K. (2024). Shared Genetic Associations and Etiology Between Obstructive Sleep Apnea and Cvds: A Genome-Wide Cross-Trait Analysis and Bidirectional Mendelian Randomization Analysis. *European Journal of Preventive Cardiology*.

[27] Wang Q., Dhindsa R. S., Carss K. (2021). Rare Variant Contribution to Human Disease in 281, 104 Uk Biobank Exomes. *Nature*.

[28] Warde-Farley D., Donaldson S. L., Comes O. (2010). The Genemania Prediction Server: Biological Network Integration for Gene Prioritization and Predicting Gene Function. *Nucleic Acids Research*.

[29] Yoo M., Shin J., Kim J. (2015). Dsigdb: Drug Signatures Database for Gene Set Analysis. *Bioinformatics*.

[30] Lv Q., Chen G., He H. (2023). Tcmbank-the Largest Tcm Database Provides Deep Learning-Based Chinese-Western Medicine Exclusion Prediction. *Signal Transduction and Targeted Therapy*.

[31] Tuo Y., Lu X., Tao F. (2024). The Potential Mechanisms of Catechins in Tea for Anti-Hypertension: An Integration of Network Pharmacology, Molecular Docking, and Molecular Dynamics Simulation. *Food*.

[32] Zhao N., Shen M., Zhao R. (2024). Sanguinarine Alleviates Ulcerative Colitis in Mice by Regulating the Nrf2/Nf-*Κ*b Pathway. *Nan Fang Yi Ke Da Xue Xue Bao*.

[33] Zheng Z. G., Zhu S. T., Cheng H. M. (2021). Discovery of a Potent Scap Degrader That Ameliorates Hfd-Induced Obesity, Hyperlipidemia and Insulin Resistance via an Autophagy-Independent Lysosomal Pathway. *Autophagy*.

[34] Bolijn R., Cham Sieben A. E., Kunst M., Blom H. L., Tan H. L., van Valkengoed I. G. M. (2021). Sex Differences in Incidence of Out-of-Hospital Cardiac Arrest Across Ethnic and Socioeconomic Groups: A Population-Based Cohort Study in the Netherlands. *International Journal of Cardiology*.

[35] Akdis D., Saguner A. M., Shah K. (2017). Sex Hormones Affect Outcome in Arrhythmogenic Right Ventricular Cardiomyopathy/Dysplasia: From a Stem Cell Derived Cardiomyocyte-Based Model to Clinical Biomarkers of Disease Outcome. *European Heart Journal*.

[36] Aribas E., Kavousi M., Laven J. S. E., Ikram M. A., Roeters van Lennep J. E. (2021). Aging, Cardiovascular Risk, and Shbg Levels in Men and Women From the General Population. *The Journal of Clinical Endocrinology and Metabolism*.

[37] Xu S. M., Lu K., Yang X. F. (2023). Association of 25-Hydroxyvitamin D Levels With Lipid Profiles in Osteoporosis Patients: A Retrospective Cross-Sectional Study. *Journal of Orthopaedic Surgery and Research*.

[38] Morvaridzadeh M., Agah S., Alibakhshi P. (2021). Effects of Calcium and Vitamin D Co-Supplementation on the Lipid Profile: A Systematic Review and Meta-Analysis. *Clinical Therapeutics*.

[39] Alam Y. H., Kim R., Jang C. (2022). Metabolism and Health Impacts of Dietary Sugars. *Journal of Lipid and Atherosclerosis*.

[40] Lockridge A., Hanover J. A. (2022). A Nexus of Lipid and O-Glcnac Metabolism in Physiology and Disease. *Frontiers in Endocrinology*.

[41] Manousaki D., Harroud A., Mitchell R. E. (2021). Vitamin D Levels and Risk of Type 1 Diabetes: A Mendelian Randomization Study. *PLoS Medicine*.

[42] Richardson T. G., Sanderson E., Palmer T. M. (2020). Evaluating the Relationship Between Circulating Lipoprotein Lipids and Apolipoproteins With Risk of Coronary Heart Disease: A Multivariable Mendelian Randomisation Analysis. *PLoS Medicine*.

[43] Lin Y. C., Tu H. P., Wang T. N. (2024). Blood Lipid Profile, Hba1c, Fasting Glucose, and Diabetes: A Cohort Study and a Two-Sample Mendelian Randomization Analysis. *Journal of Endocrinological Investigation*.

[44] Zhang Z., Qiu S., Wang Z., Hu Y. (2024). Vitamin D Levels and Five Cardiovascular Diseases: A Mendelian Randomization Study. *Heliyon*.

[45] Jeong J. H., Kim Y. G., Han K. D. (2024). Association of Fatty Liver Index With Sudden Cardiac Arrest in Young Adults. *Metabolism*.

[46] Lee H. Y., Lee D. H., Lee B. K. (2019). The Association Between Lipid Profiles and the Neurologic Outcome in Patients With Out-of-Hospital Cardiac Arrest. *Resuscitation*.

[47] Kong S. Y., Jung E., Hwang S. S. (2023). Circulating Vitamin D Level and Risk of Sudden Cardiac Death and Cardiovascular Mortality: A Dose-Response Meta-Analysis of Prospective Studies. *Journal of Korean Medical Science*.

[48] Chae B., Shin Y. S., Kim S. M. (2022). Association Between Vitamin D Deficiency and Neurologic Outcomes in Patients After Cardiopulmonary Resuscitation. *Shock*.

[49] Mai X. M., Videm V., Sheehan N. A., Chen Y., Langhammer A., Sun Y. Q. (2019). Potential Causal Associations of Serum 25-Hydroxyvitamin D With Lipids: A Mendelian Randomization Approach of the Hunt Study. *European Journal of Epidemiology*.

[50] Jabbari R., Risgaard B., Holst A. G. (2013). Cardiac Symptoms Before Sudden Cardiac Death Caused by Coronary Artery Disease: A Nationwide Study Among Young Danish People. *Heart*.

[51] Su Y., Huang P., Wu Z., Dai W., Zhang Y., Zeng J. (2024). Effect of Dietary Supplementation With Sanguinarine on Meat Quality and Lipid Metabolism of Broilers. *Poultry Science*.

[52] Jeng J. H., Wu H. L., Lin B. R. (2007). Antiplatelet Effect of Sanguinarine Is Correlated to Calcium Mobilization, Thromboxane and Camp Production. *Atherosclerosis*.

[53] Tuo P., Zhao R., Li N. (2024). Lycorine Inhibits Ang II-Induced Heart Remodeling and Inflammation by Suppressing the Pi3k-Akt/Nf-*Κ*b Pathway. *Phytomedicine*.

[54] Liang X., Fu W., Peng Y. (2023). Lycorine Induces Apoptosis of Acute Myeloid Leukemia Cells and Inhibits Triglyceride Production via Binding and Targeting Fabp5. *Annals of Hematology*.

