# Supplementary material for: Exploring Therapeutic Targets for Preventing Cardiac Arrest by Modulating Dyslipidemia and 25-Hydroxyvitamin D Metabolism: A Mendelian Randomization Study
Source: Hum Mutat. 2025 Jun 19;2025:5536318. doi: 10.1155/humu/5536318 (PMC12202069; doi:10.1155/humu/5536318)
Supplement: Supporting Information 1 — Figure S1: Three significant assumptions for Mendelian randomization. [file 5536318.f1.pdf]

Figure S1. Three significant assumptions for Mendelian randomization

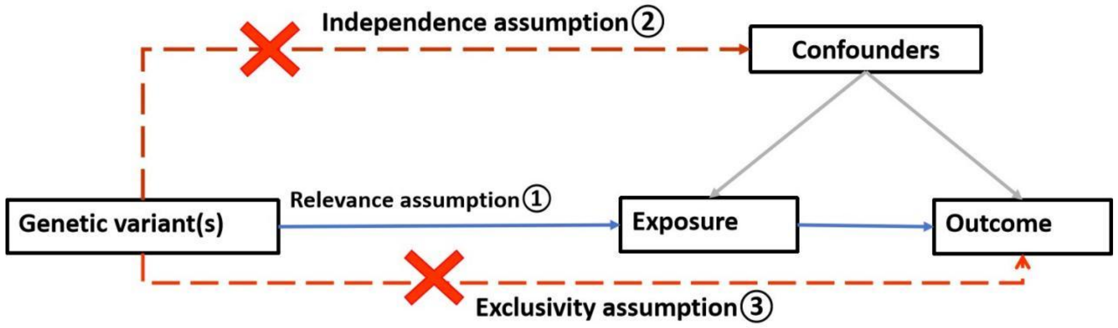

Assumption 1: Genetic instruments should be robustly associated with the exposure. Assumption 2:Genetic instruments should not be associated with any confounders. Assumption 3: Geneticinstruments influence risk of the outcome through the exposure, rather than through other pathways
