# Supplementary material for: Exploring Therapeutic Targets for Preventing Cardiac Arrest by Modulating Dyslipidemia and 25-Hydroxyvitamin D Metabolism: A Mendelian Randomization Study
Source: Hum Mutat. 2025 Jun 19;2025:5536318. doi: 10.1155/humu/5536318 (PMC12202069; doi:10.1155/humu/5536318)
Supplement: Supporting Information 2 — Figure S2: Forest plot of Mendelian randomization results after excluding SNPs associated with testosterone, SHBG, and estradiol levels. [file 5536318.f2.pdf]

Figure S2. Forest Plot of Mendelian Randomization Results After Excluding SNPs Associated With Testosterone, SHBG, and Estradiol Levels

Exposure	Method	Estimate	Pval
LDL−C			
	MR Egger	1.62(1.20−2.18)	0.002
	Weighted median	1.62(1.25−2.11)	0.001
	Inverse variance weighted	1.5(1.24−1.80)	0.001
Triglycerides			
	MR Egger	1.36(0.92−2.00)	0.128
	Weighted median	1.23(0.87−1.73)	0.241
	Inverse variance weighted	1.33(1.05−1.67)	0.016
Apolipoprotein A1			
	MR Egger	1.01(0.84−1.22)	0.919
	Weighted median	0.92(0.75−1.14)	0.446
	Inverse variance weighted	0.87(0.76−0.99)	0.049
Apolipoprotein B			
	MR Egger	1.80(0.91−3.57)	0.104
	Weighted median	1.85(1.26−2.72)	0.002
	Inverse variance weighted	1.98(1.48−2.63)	0.001
HDL−C			
	MR Egger	0.99(0.77−1.26)	0.909
	Weighted median	0.86(0.66−1.12)	0.268
	Inverse variance weighted	0.85(0.72−0.99)	0.0491
25−Hydroxyvitamin D			
	MR Egger	1.01(0.68−1.49)	0.983
	Weighted median	0.79(0.54−1.17)	0.245
	Inverse variance weighted	0.74(0.55−0.96)	0.023

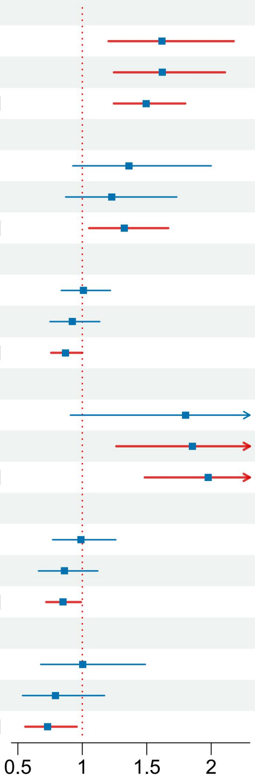

Abbreviations: SHBG, sex hormone-binding globulin； LDL-C,Low-Density Lipoprotein Cholesterol；HDL-C,.High-Density Lipoprotein Cholesterol.
