# Supplementary material for: Exploring Therapeutic Targets for Preventing Cardiac Arrest by Modulating Dyslipidemia and 25-Hydroxyvitamin D Metabolism: A Mendelian Randomization Study
Source: Hum Mutat. 2025 Jun 19;2025:5536318. doi: 10.1155/humu/5536318 (PMC12202069; doi:10.1155/humu/5536318)
Supplement: Supporting Information 3 — Figure S3: Results of PheWAS analysis for nine genes. [file 5536318.f3.pdf]

Figure S3 Results of PheWAS analysis for 9 genes(a-i)The bottom dashed line represents the Suggestive line and the top dashed line is the Significant line. Traits that exceeded the significant line were considered to be significantly associated with a gene.

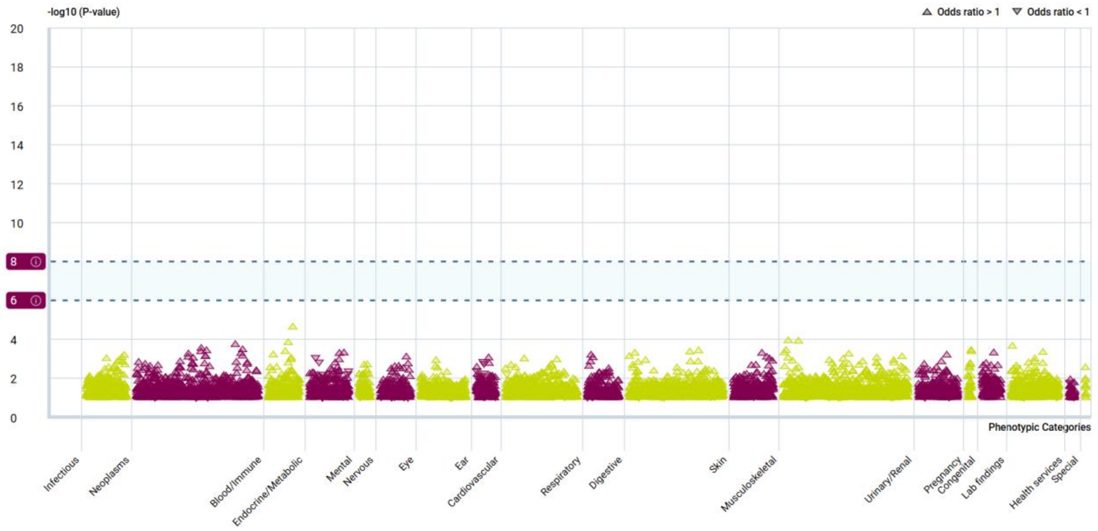

a. Binary traits PheWAS association with NMRAL1

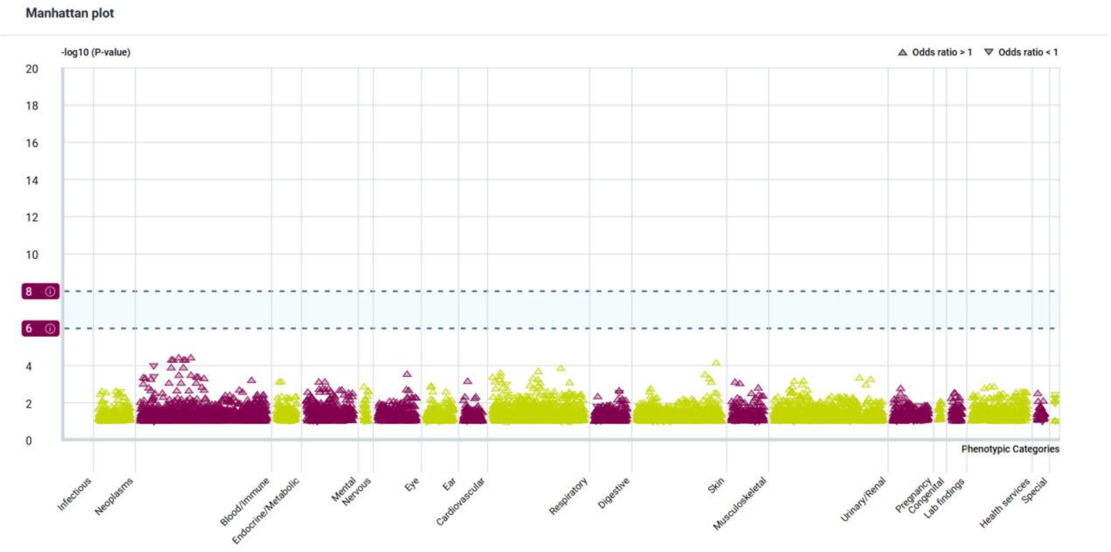

b. Binary traits PheWAS association with CEACAM6

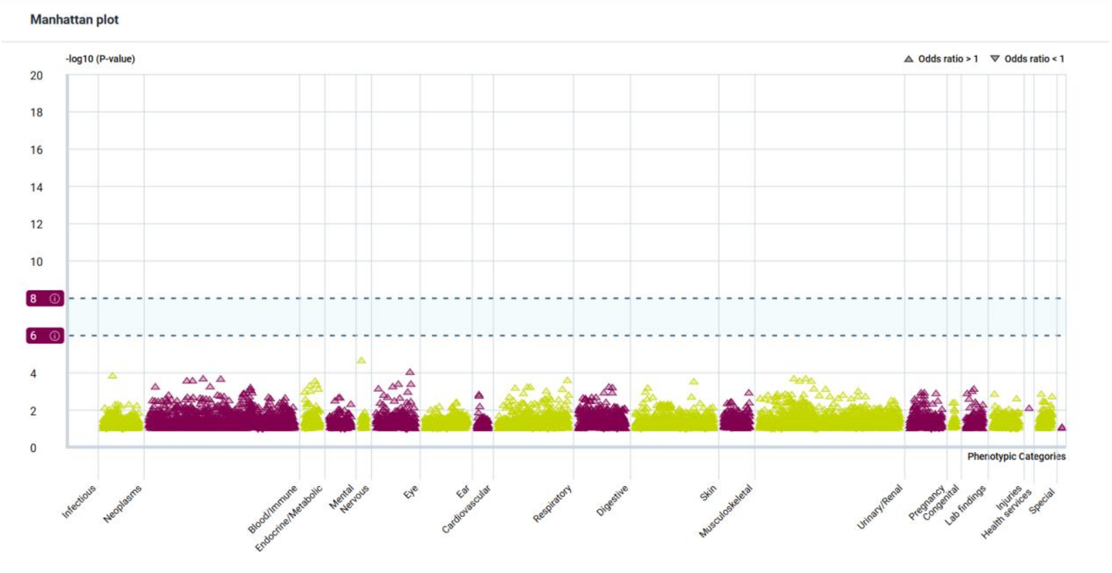

c. Binary traits PheWAS association with CA8

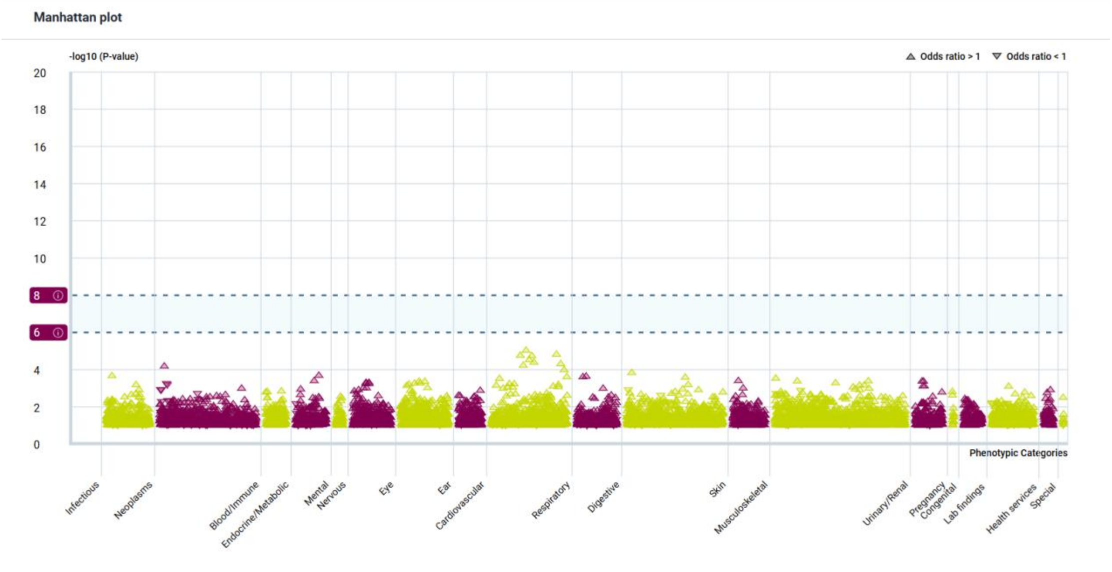

d. Binary traits PheWAS association with HMGCR

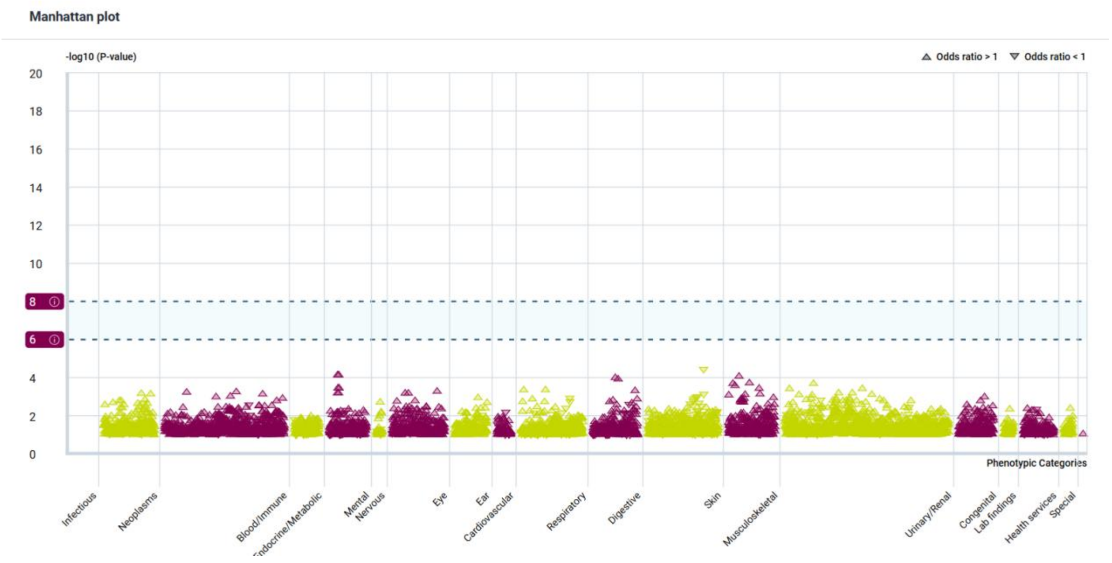

e. Binary traits PheWAS association with CBX6

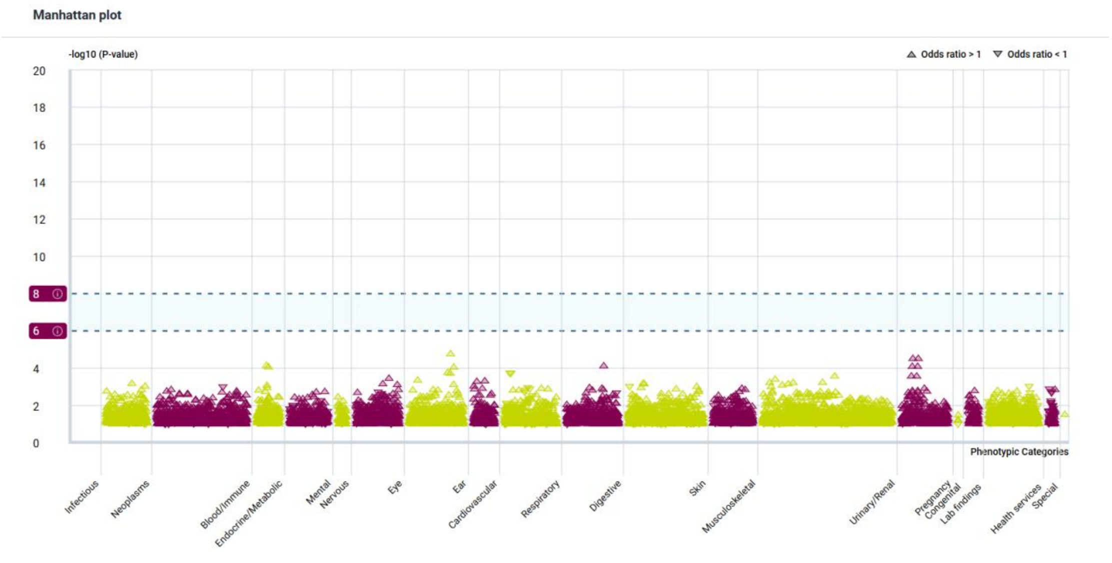

f. Binary traits PheWAS association with TSTA3

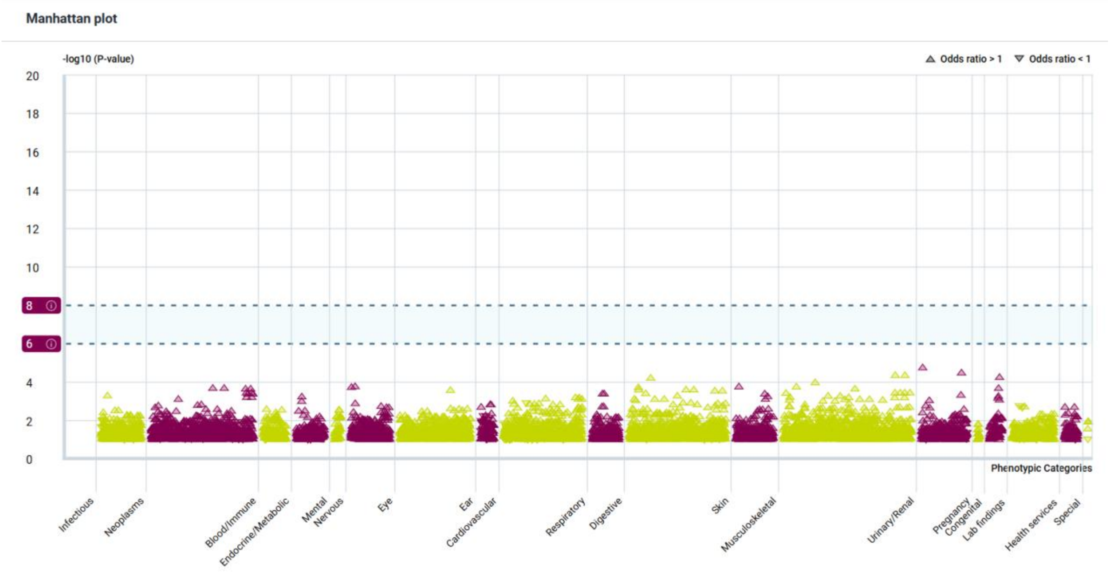

g.Binary traits PheWAS association with BATF2

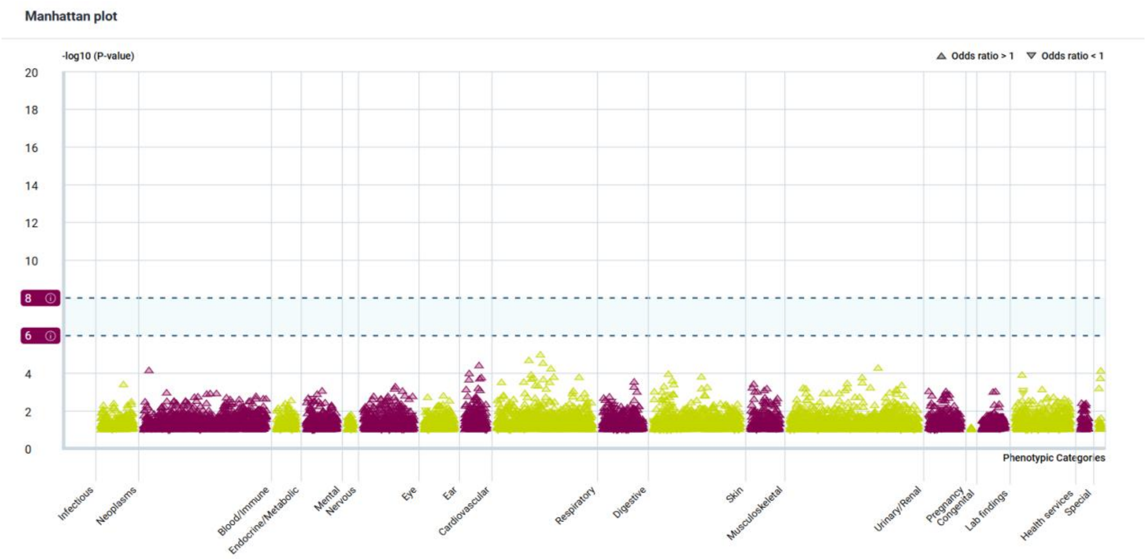

h.Binary traits PheWAS association with ACP2

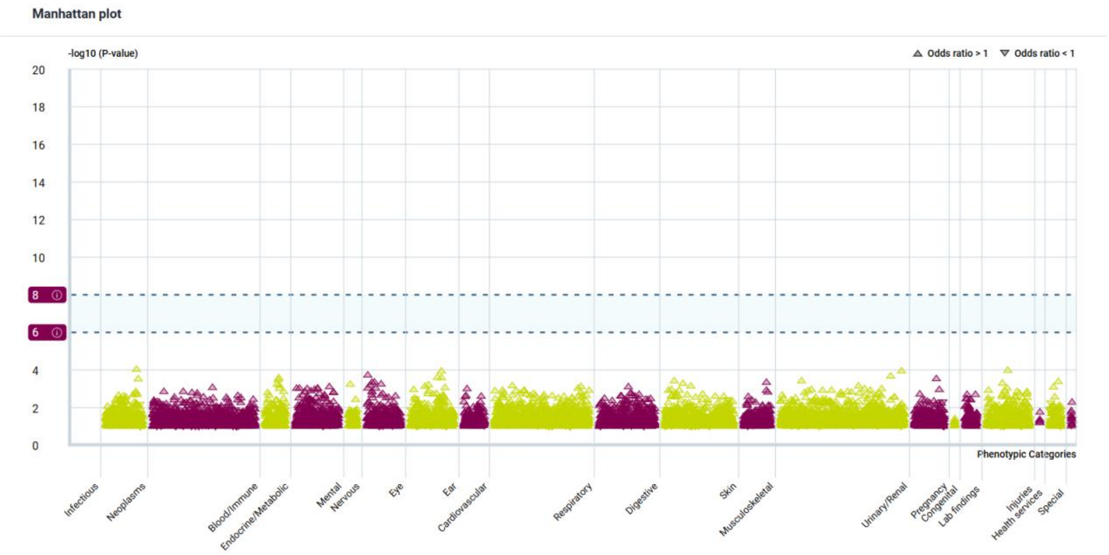

i.Binary traits PheWAS association with GBP1
