# Supplementary material for: Exploring Therapeutic Targets for Preventing Cardiac Arrest by Modulating Dyslipidemia and 25-Hydroxyvitamin D Metabolism: A Mendelian Randomization Study
Source: Hum Mutat. 2025 Jun 19;2025:5536318. doi: 10.1155/humu/5536318 (PMC12202069; doi:10.1155/humu/5536318)
Supplement: Supporting Information 4 — Table S1: STROBE-MR checklist of recommended items to address in reports of Mendelian randomization studies. Table S2: Detailed information on used GWAS studies. Table S3: Genetic variants used as instrumental variables in univariable MR. Table S4: Confounding factors and the SNPs associated with lipid traits and 25(OH)D. Table S5: Cochran Q test result and MR-Egger intercept in univariable MR. Table S6: Sex-related SNPs among lipid traits and 25(OH)D. Table S7: Cochran Q test result and MR-Egger intercept in univariable MR outcome: serum 25(OH)D. Table S8: Cochran Q test result and MR-Egger intercept in univariable MR exposure: serum 25(OH)D. Table S9: Results of Bayesian tests for colocalization. Table S10: The results of MR-radial method of size-defined lipoprotein particle fractions on CA risk. Table S11: Analyses for the causal impact of lipids and 25(OH)D on mediators. Table S12: MVMR analyses for the causal impact of potential mediators on CA. Table S13: Results of Mendelian mediation analysis. Table S14: Baseline characteristics of the study population. Table S15: Baseline characteristics of the study population. Table S16: HRs (95% CIs) for all-cause mortality and CVD mortality according to vitamin D concentrations. Table S17: Sensitivity analysis of the association of the vitamin D concentrations with all-cause mortality and CVD mortality. Table S18: colocalization results of eQTLs for nine genes with apolipoprotein A1, HDL, and serum 25-hydroxyvitamin D associated SNPs. Table S19: The impact of druggable genes on lipid traits, vitamin D, and the risk of cardiac arrest. Table S20: Candidate drug predicted using DSigDB and TCMSP. Table S21: Docking results of available proteins with small molecules. [file 5536318.f4.pdf]

Supplementary Table 1- Supplementary Table 21

TableS1 STROBE-MR checklist of recommended items to address in reports of Mendelian randomization stuies [1,2]

Item No.	Section	Checklist item	Relevant text from manuscript
1	**TITLE and ABSTRACT**	Indicate Mendelian randomization (MR) as the study's design in the title and/or the abstract if that is a main purpose of the study	Exploring Therapeutic Targets for Cardiac Arrest Prevention by Modulating Dyslipidemia and 25-Hydroxy25(OH)D Metabolism: A Mendelian Randomization Study
	**INTRODUCTION**		
2	**Background**	Explain the scientific background and rationale for the reported study. What is theexposure? is a potential causal relationship between exposure and outcomeplausible? Justify why MR is a helpful method to address the study question.	Cardiac arrest has an extremely high mortality rate once it occurs, making the prevention of its occurrence a current focus of attention. Although dyslipidemia and 25-Hydroxy25(OH)D [25(OH)D] levels are established risk factors for cardiovascular disease, their causal relationship with cardiac arrest remains to be elucidated.
3	**Objectives**	State specific objectives clearly, including pre-specified causal hypotheses (if any), State that MR is a method that, under specific assumptions, intends to estimatecausal effects	This research aims to investigate the causal relationships among dyslipidemia, 25(OH)D levels, and cardiac arrest, identifying new potentially therapeutic targets for cardiac arrest prevention.
	**METHODS**		
4	**Study design and data sources**	Present key elements of the study design early in the article. Consider including a table listing sources of data for all phases of the study. For each data source contributing to the analysis, describe the following:	

	a) Setting: Describe the study design and the underlying population, if possible. Describe the setting, locations, and relevant dates, including periods of recruitment, exposure, follow-up, and data collection, when available.	The GWAS summary statistics used in this research were obtained from publicly available data sets from relevant authoritative consortiums. Summary statistics of high-density lipoprotein cholesterol (HDL-C) comprised of 357,810 samples and 10,783,660 SNPs.low-density lipoprotein cholesterol (LDL-C) comprised of 431,167 samples and 16,293,344 SNPs.Triglycerides (TG) comprised of 94,595 samples and 2,410,057 SNPs. Apolipoprotein A1 (APOA1) levels comprised of 398,508 samples and 4,218,115 SNPs. Apolipoprotein B (APOB) levels comprised of 115,082samples and 11,590,399 SNPs, Serum 25(OH)D levels comprised of 496,946 samples and 6,896,093 SNPs, and the relevant data can be accessed openly at https://gwas.mrcieu.ac.uk. Cardiac arrest related datasets were obtained from the FinnGen consortium.
	b) Participants: Give the eligibility criteria, and the sources and methods of selection of participants. Report the sample size, and whether any power or sample size calculations were carried out prior to the main analysis	The UK Biobank (UKB) is a prospective cohort study comprising approximately 500,000 individuals aged 39 to 72 years from across the United Kingdom. Participants attended one of 21 assessment centers, where blood samples were collected for biomarker and genetic analyses, and height and weight were measured to calculate body mass index. LDL-C levels were measured at the baseline visit (2006–2010) in a non-fasting state. In addition, APOA1 (n = 398,508), APOB (n = 115,082), and HDL-C (n = 357,810) levels were also derived from UKB participants.Serum 25(OH)D concentrations were assessed using blood samples collected at two timepoints: the initial visit (2006–2010) and the repeat assessment (2012–2013),and participants with concentrations outside the validated assay range (10–375 nmol/L) were excluded, resulting in a final study sample of 496,946 individuals. Data on TG were obtained from the Global Lipids Genetics Consortium (GLGC), comprising 177,861 individuals, blood lipid levels were typically measured after more than 8 hours of fasting, and articipants known to be taking lipid-lowering medications were excluded whenever possible. Cardiac arrest–related datasets were obtained from the FinnGen consortium, which launched in 2017, aims to collect genotype data from 500,000 Finnish individuals and link it with comprehensive national health register data. Individuals who experienced cardiac arrest or sudden cardiac death were included as cases,and a total of 2,722 cases and 231,952 controls were included in the analysis.
	c) Describe measurement, quality control and selection of genetic variants	To identify SNPs associated with exposure factors and establish the validity and accuracy of the causal relationship between lipid, 25(OH)D levels and Cardiac arrest the following steps were followed to select the most suitable SNPs. Firstly, a significance threshold of P value was less than 5×10^{-8} was established for the

			detection of SNPs that exhibit strong associations with the investigated exposures. Moreover, to eliminate any presence of linkage disequilibrium, an r^2 threshold of 0.001 and a clump window size of 10,000 kb were implemented. In addition, the selected IVs were assessed for the weak Instrumental variables (IVs) bias by calculating the F-statistic.
		d) For each exposure, outcome, and other relevant variables, describe methods of assessment and diagnostic criteria for diseases	Lipid-related biomarkers and 25(OH)D levels were measured using peripheral blood samples collected from participants, with values outside the normal range considered abnormal. In the UK Biobank, lipid measurements were obtained from specific data fields: LDL-C (Field ID 30780), HDL-C (Field ID 30760), and triglycerides (Field ID 30870). Cardiac arrest cases were defined as individuals who experienced cardiac arrest, and sudden cardiac death cases referred specifically to those with a cardiac origin. In the FinnGen database, cardiac arrest was identified using diagnostic codes: ICD-10 code I46, ICD-9 codes 4275A and 4112, and ICD-8 code 4272.
		e) Provide details of ethics committee approval and participant informed consent, if relevant	This study used publicly available genome-wide association study (GWAS) summary statistics. The original GWAS datasets were approved by the respective ethics committees, and informed consent was obtained from all participants in the primary studies. No additional ethics approval or informed consent was required for this secondary analysis of anonymized data.
5	**Assumptions**	Explicitly state the three core IV assumptions for the main analysis (relevance, independence and exclusion restriction) as well assumptions for any additional or sensitivity analysis	This study strictly adhered to the core principles of Mendelian randomization. IVs were required to satisfy three key assumptions: relevance, independence, and exclusion restriction. Specifically, each SNP was strongly associated with the exposure of interest (relevance), not associated with potential confounders (independence), potential confounders were screened using the IEU Open GWAS database to ensure independence, and influenced the outcome solely through the exposure pathway (exclusion restriction). In addition, sensitivity analyses and pleiotropy tests were performed to evaluate the robustness of the findings.
6	**Statistical methods: main analysis**	Describe statistical methods and statistics used	
		a) Describe how quantitative variables were handled in the analyses (i.e., scale, units, model)	The data used in this study were derived from publicly available summary datasets and did not involve any transformations of quantitative variables.

		b) Describe how genetic variants were handled in the analyses and, if applicable, how their weights were selected	SNPs strongly associated with the exposures of interest were selected using a genome-wide significance threshold of $p < 5\times10^{-8}$. To ensure the independence of these instruments, linkage disequilibrium (LD) clumping was performed with an r^2 threshold of < 0.01 and a 10,000 kb window.To minimize potential reverse causality, SNPs associated with the outcome at a significance level of $p < 5\times10^{-6}$ were excluded. Additionally, to address confounding, we utilized the LDtrait and IEU OpenGWAS platforms to identify and remove SNPs associated with known confounders, such as obesity, smoking, alcohol consumption, and cardiovascular comorbidities. For the main MR analyses, we used the beta coefficients of SNP–exposure associations as instrumental weights. Causal estimates were primarily derived using the inverse variance weighted (IVW) method, and the robustness of results was further assessed using the MR-Egger and weighted median approaches.Heterogeneity among SNPs was evaluated using Cochran's Q test. The MR-Egger intercept was used to detect potential horizontal pleiotropy. Causal effects were reported as odds ratios (ORs) with corresponding 95% confidence intervals (CIs).
		c) Describe the MR estimator (e.g. two-stage least squares, Wald ratio) and related statistics. Detail the included covariates and, in case of two-sample MR, whether the same covariate set was used for adjustment in the two samples	The exposure and outcome datasets were obtained from different populations in this two-sample MR study. However, all contributing GWAS adjusted for at least age, sex, and genetic ancestry, ensuring comparability between samples and minimizing potential confounding due to population stratification.The genetic associations for the exposures were sourced from large-scale meta-analyses of genome-wide association studies (GWAS). For each SNP, causal estimates were calculated using the Wald ratio, and these were aggregated using several Mendelian randomization methods, including IVW with a multiplicative random-effects model, MR-Egger regression, and the weighted median approach. IVW served as the principal method, applying weights based on the inverse variance of each estimate. MR-Egger offered an alternative approach that adjusts for potential directional pleiotropy.
		d) Explain how missing data were addressed	In this MR analysis, the issue of missing data was not involved.
		e) If applicable, indicate how multiple testing was addressed	In this MR analysis, multiple exposures or multiple outcomes were not involved, so multiple testing was not performed.
7	**Assessment of**	Describe any methods or prior knowledge used to assess the assumptions or justify their validity	To evaluate the risk of weak instrument bias, we calculated the F-statistic for each selected instrumental variable (IV). The F-statistic was derived using the formula:a F = R2(N − K − 1) / [K (1 −R2)]. where R^2 is the

	**assumptions**		proportion of variance explained, N is the sample size, and K is the number of instruments.To assess potential bias in the causal estimates due to pleiotropy, we conducted sensitivity analyses using methods such as MR-Egger and the weighted median (WM) approach. These analyses were performed using the TwoSampleMR package in R.
8	**Sensitivity analyses and additional analyses**	Describe any sensitivity analyses or additional analyses performed (e.g. comparison of effect estimates from different approaches, independent replication, bias analytic techniques, validation of instruments, simulations)	To ensure the robustness of the Mendelian randomization (MR) results, both MR-Egger and weighted median (WM) methods were employed as complementary approaches. MR-Egger uses a weighted linear regression model to provide estimates that are robust to violations of instrumental variable assumptions, although it tends to have lower statistical power and may be influenced by outlier variants. The WM method, similar to the IVW approach, assigns weights based on the inverse variance of each SNP and remains reliable even if up to 50% of the instruments are invalid. Cochran's Q test was performed to assess heterogeneity among SNPs. Additionally, horizontal pleiotropy was examined using the MR-Egger intercept.
	**Software and pre-registration**		
		a) Name statistical software and package(s), including version and settings used	All analyses were conducted using R version 4.3.1, with the software packages 'Two-SampleMR','MVMR' , and MendelianRandomization' and so on.Tovisualize the MR analysis, forest plots, leave-one-out plots were generated using the data analysisfunction of the Rstudio platform.
		b) State whether the study protocol and details were pre-registered (as well as when and where)	In this study, the MR analysis was conducted using publicly available summary-level GWAS data. The original GWAS projects (such as UK Biobank, FinnGen, and GLGC) had obtained ethical approval and informed consent from participants during data collection. No individually identifiable information was used. This study was not pre-registered with a study protocol or detailed analysis plan.
	**RESULTS**		
**10**	**Descriptive data**		
		a) Report the numbers of individuals at each stage of included studies and reasons for exclusion.	The GWAS summary statistics used in this Mendelian randomization study were obtained from publicly available datasets.The following sample sizes counts were included for each exposure trait: HDL-C: 357,810 individuals, LDL-C:431,167 individuals, TG: 94,595 individuals, APOA1: 398,508 individuals, APOB:115,082 individuals, 25(OH)D: 496,946 individuals. All exposure data were derived from large-scale GWAS

		meta-analyses and the UK Biobank. SNPs were excluded from the analysis if they were palindromic with intermediate allele frequencies, or if they were not available in the outcome dataset. Outcome data for cardiac arrest and sudden cardiac death were obtained from the FinnGen consortium, with a total of 2,722 cases and 231,952 controls.
	b) Report summary statistics for phenotypic exposure(s), outcome(s), and other relevant variables (e.g. means, SDs, proportions)	Summary data on exposure and outcomes are shown in Supplementary Table S2-S5.
	c) If the data sources include meta-analyses of previous studies, provide the assessments of heterogeneity across these studies	This study did not perform or include any meta-analyses of individual-level studies. Therefore, assessments of heterogeneity across studies were not applicable.
	d) For two-sample MR:<br>i. Provide justification of the similarity of the genetic variant-exposure associations between the exposure and outcome samples<br>ii. Provide information on the number of individuals who overlap between the exposure and outcome studies	The data presented in this study were derived exclusively from European population samples. These samples were obtained from independent GWAS databases, ensuring minimal overlap and bias, detailed data on the number of individuals in the exposure and outcome samples are provided in Table S2.
11<br>**Main results**		
	a) Report the associations between genetic variant and exposure, and between genetic variant and outcome, preferably on an interpretable scale	Univariable forward Mendelian randomization (MR) analysis indicated suggestive associations between genetically proxied lipid traits or 25(OH)D levels and the risk of cardiac arrest (CA). Specifically,nverse-variance weighted (IVW) multivariable regression analysis revealed that higher LDL-C (OR: 0.91, 95% CI: 0.83 – 0.93, p < 0.001), TG (OR: 0.88, 95% CI: 0.83 – 0.92, p < 0.001), ApoA1 (OR: 0.95, 95% CI: 0.92 – 0.98, p = 0.012), and ApoB (OR: 0.499, 95% CI: 0.289 – 0.860, p = 0.001) levels were associated with a reduced risk of low serum 25(OH)D levels. This association was further supported by the MR-Egger, weighted median MR, and simple mode methods, with corresponding p-values < 0.05. No causal relationship was found between HDL-C and 25(OH)D levels (OR: 0.95, 95% CI: 0.92 – 0.98) (Figure 3A).

			Furthermore, we conducted a sensitivity analysis to address the horizontal pleiotropy assumption of the MR analysis, and found no evidence of horizontal pleiotropy except for APOA1. A thorough assessment of heterogeneity and horizontal pleiotropy is provided in Supplementary Table S4. Reverse MR analyses demonstrated significant causal effects of 25(OH)D on three lipid-related traits: LDL-C (OR: 0.72, 95% CI: 056 – 0.92; p =0.01, IVW), TG (OR: 0.60, 95% CI: 0.46 – 0.80; p < 0.001, IVW), and APOB (OR: 0.75, 95% CI: 0.63 – 0.91; p = 0.042, IVW).
		b) Report MR estimates of the relationship between exposure and outcome, and the measures of uncertainty from the MR analysis, on an interpretable scale, such as odds ratio or relative risk per SD differenc	Mengelian randomization estimation reports are detailed in odds ratio or relative risk per SD difference Figure 2.
		c) Consider plots to visualize results (e.g. forest plot, scatterplot of associations between genetic variants and outcome versus between genetic variants and exposure)	In this study, forest plots were used to visualize the results of the Mendelian randomization analyses.
12	**Assessment of assumptions**		
		a) Report the assessment of the validity of the assumptions	Firstly, SNPs related to lipid traits as instrumental variables by applying widely accepted threshold criteria, indicating a strong association between these SNPs and the exposures. The large F-statistics further suggested that the analyses were unlikely to be affected by weak instrument bias. Secondly, the selected SNPs were verified to have no associations with potential confounding factors that could influence the exposure-outcome relationship. Lastly, these SNPs were confirmed to affect the outcome solely through their influence on the exposures.
		b) Report any additional statistics (e.g., assessments of heterogeneity across genetic variants, such as *I2* , Q statistic or E-value)	The Cochran's Q test did not detect any heterogeneity of the SNPs, These causal relationships did not show any directional pleiotropy according to the MR-Egger intercept test, as detailed in Supplementary Table S5.
13	**Sensitivity analyses and**		

	**additional analyses**		
		a) Report any sensitivity analyses to assess the robustness of the main results to violations of the assumptions	Sensitivity analyses to assess the robustness of the main results are presented in Table S5.
		b) Report any assessment of direction of causal relationship (e.g., bidirectional MR)	We conducted bidirectional MR analyses to evaluate potential causal relationships between lipid traits, 25(OH)D levels, and cardiac arrest. The forward MR analysis supported a causal effect of lipid traits and 25(OH)D on the risk of cardiac arrest. Additionally, as shown in Figure 3, we also examined the bidirectional relationship between lipid traits and 25(OH)D. In the mediation MR analysis, bidirectional MR was further performed between each mediator and cardiac arrest, revealing no evidence of a reverse causal effect from the mediators to cardiac arrest.
		c) When relevant, report and compare with estimates from non-MR analyses	This study investigated the association between 25(OH)D levels and cardiovascular mortality among 9,988 patients with dyslipidemia in the NHANES database. The results confirmed that patients with lower 25(OH)D levels had a higher risk of cardiovascular mortality.
		d))Consider additional plots to visualize results (e.g., leave-one-out analyses)	In this study, both forest map analyses and a heatmap were used to visualize the MR results, as shown in Figure 2 and Figure 4.
	**DISCUSSION**		
14	**Key results**	Summarize key results with reference to study objectives	This study suggests a causal relationship between dyslipidemia, 25(OH)D deficiency, and sudden cardiac arrest. Both dyslipidemia and 25(OH)D deficiency independently increase the risk of sudden cardiac arrest. Additionally, there is a reciprocal influence between dyslipidemia and 25(OH)D levels, with dyslipidemia contributing to the exacerbation of 25(OH)D deficiency.
15	**Limitations**	Discuss limitations of the study, taking into account the validity of the IV assumptions, other sources of potential bias, and imprecision. Discuss both direction and magnitude of any potential bias and any efforts to address them	The limitations of this study are as follows. First, the database used in this study primarily includes individuals of European descent. To strengthen the evidence, it would be important to expand the database to include individuals from other ethnic backgrounds, such as those of Asian or African descent. Second, this study relied on publicly available summary-level data, which limited the ability to conduct individual-level analyses. As a result, we were unable to perform stratification by factors such as gender and age. Regarding validation using the NHANES database, the lack of specific information on cardiac arrest cases, coupled with the fact that

			cardiovascular disease is the leading cause of cardiac arrest, led us to use individuals who died from cardiovascular diseases for validation. Therefore, the results may not completely reflect the reality,future cohort studies are needed to further validate these findings.
16	**Interpretation**		
		a) Meaning: Give a cautious overall interpretation of results in the context of their limitations and in comparison with other studies	The results suggest a potential causal relationship between lipid traits, 25(OH)D levels, and the occurrence of cardiac arrest.
		b) Mechanism: Discuss underlying biological mechanisms that could drive a potential causal relationship between the investigated exposure and the outcome, and whether the gene-environment equivalence assumption is reasonable. Use causal language carefully, clarifying that IV estimates may provide causal effects only under certain assumptions	The findings suggest that higher levels of HDL-C, ApoA1, and 25(OH)D are associated with a reduced risk of cardiac arrest, whereas elevated levels of LDL-C, ApoB, and triglycerides (TG) are linked to an increased risk. These results offer valuable insights into the mechanisms of cardiac arrest and highlight potential targets for prevention. Additionally, this study provides evidence of an inverse causal relationship between genetically predicted serum 25(OH)D levels and both TG and LDL-C levels, which aligns with previous research. Further colocalization analysis revealed shared genetic susceptibility loci between LDL-C, ApoB, and 25(OH)D, strongly suggesting that these factors may jointly contribute to the onset and progression of cardiac arrest. Dyslipidemia and 25(OH)D deficiency are growing global public health challenges, often associated with comorbidities such as hypertension, diabetes, coronary heart disease, and cerebrovascular disease. Both mediation analysis and validation in the NHANES dataset further support the hypothesis that dyslipidemia and reduced 25(OH)D levels may mediate cardiovascular disease and increase the risk of cardiac arrest.
		c) Clinical relevance: Discuss whether the results have clinical or public policy relevance, and to what extent they inform effect sizes of possible interventions	The prevention of sudden cardiac arrest (SCA) is critically important. This study demonstrates that both lipid levels and serum 25(OH)D concentrations influence the risk of SCA. Additionally, the mutual influence observed between dyslipidemia and 25(OH)D deficiency suggests that these two factors may interact in contributing to disease development. These findings highlight the importance of concurrently addressing lipid abnormalities and 25(OH)D deficiency in clinical practice. Such an approach may offer a new direction for the prevention of SCA and inform both clinical strategies and public health interventions.

17	**Generalizability**	Discuss the generalizability of the study results (a) to other populations, (b) across other exposure periods/timings, and (c) across other levels of exposure	(a) This study was conducted using data from individuals of European ancestry, which may limit the generalizability of the findings to other populations, such as those of Asian or African descent.(b) The study did not explore the impact of different exposure periods or the timing of lipid or 25(OH)D levels, making it difficult to infer whether the observed associations hold true across varying life stages or durations of exposure.(c) In the validation phase using NHANES data, the study demonstrated a dose-dependent association between 25(OH)D levels and cardiac arrest among individuals with dyslipidemia. These findings suggest that maintaining serum 25(OH)D levels within an appropriate range may be beneficial in reducing the risk of cardiac arrest.
	**OTHER INFORMATION**		
18	**Funding**	Describe sources of funding and the role of funders in the present study and, if applicable, sources of funding for the databases and original study or studies on which the present study is based	This research was funded by the project of Henan Province Medical Science and Technology Research Program (LHGJ20240194), Henan Province medical science and technology research plan of the province to build the key Project (SBGJ202102155),Henan Province Key Research and Development Program (251111311700).
19	**Data and data sharing**	Provide the data used to perform all analyses or report where and how the data can be accessed, and reference these sources in the article. Provide the statistical code needed to reproduce the results in the article, or report whether the code is publicly accessible and if so, where	All publicly available databases used in this study have been listed and properly referenced within the article. The access date for the NHANES database has also been clearly indicated. The statistical code used for all analyses in this study is available upon reasonable request from the corresponding author.
20	**Conflicts of Interest**	All authors should declare all potential conflicts of interest	The authors declare that the research was conducted in the absence of any commercial or financial relationships that could be construed as a potential conflict of interest.

This checklist is copyrighted by the Equator Network under the Creative Commons Attribution 3.0 Unported (CC BY 3.0) license.

1. Skrivankova VW, Richmond RC, Woolf BAR, Yarmolinsky J, Davies NM, Swanson SA, et al. Strengthening the Reporting of Observational Studies in Epidemiology using Mendelian Randomization (STROBE-MR) Statement. JAMA. 2021;under review.

2. Skrivankova VW, Richmond RC, Woolf BAR, Davies NM, Swanson SA, VanderWeele TJ, et al. Strengthening the Reporting of Observational Studies in Epidemiology using Mendelian Randomisation (STROBE-MR): Explanation and Elaboration. BMJ. 2021;375:n2233.

Table S2 Detailed information on used GWAS studies

Exposure/Outcome	Data source	Participants	GWAS ID or web sources
Blood lipid and apolipoprotein	UK Biobank/lipid GWASs of 46 studies	357,810 individuals for HDL-C,<br>431,167 individuals for LDL-C,<br>94,595 individuals for TG,<br>398,508 individuals for ApoA-I,<br>115,082 individuals for ApoB of European ancestry	34017140/ebi-a-GCST90002412<br>32493714/ebi-a-GCST90014007<br>24097068/ebi-a-GCST002216<br>34226706/ebi-a-GCST90025955<br>35213538/ebi-a-GCST90092809
Compositions in size-defined lipoproteins	UK Biobank	115,082 individuals of European ancestry	https://www.ukb iobank.ac.uk/
Serum 25-Hydroxyvitamin D levels	UK Biobank	496,946 individuals of European ancestry	32242144/ebi-a-GCST90000618
`Cardiac arrest	FinnGen biobank	234674 individuals of European ancestry	gs://finngen-public-data-r11/sum mary_stats/finngen_R11_I9_CAR DARR.gz

GWAS = genome-wide association studies, HDL-C = high-density lipoprotein cholesterol, LDL-C= low-density lipoprotein cholesterol, TG = triglycerides, ApoA-I = apolipoprotein A-I, ApoB =apolipoprotein B.

Table S3 Genetic variants used as instrumental variables in univariable MR

Traits	SNP	CHR	A1	A2	EAF	BETA	SE	p-value	Outcome
LDL-C	rs10021804	4	G	A	0.380215	-0.011471	0.00197637	6.50E-09	CA, Serum-25(OH)D
LDL-C	rs10025454	4	A	T	0.329038	-0.0121934	0.00204198	2.40E-09	CA, Serum-25(OH)D
LDL-C	rs10076475	5	A	G	0.563358	0.01221	0.00193344	2.70E-10	CA, Serum-25(OH)D
LDL-C	rs10145740	14	T	C	0.244559	-0.0183362	0.00224034	2.70E-16	CA, Serum-25(OH)D
LDL-C	rs1016102	8	C	T	0.748823	0.0132695	0.00228578	6.40E-09	CA, Serum-25(OH)D
LDL-C	rs10184004	2	T	C	0.406121	-0.0183546	0.00192292	1.40E-21	CA, Serum-25(OH)D
LDL-C	rs10272002	7	G	A	0.212476	-0.0210319	0.00234079	2.60E-19	CA, Serum-25(OH)D
LDL-C	rs10438978	18	C	T	0.818988	0.0248331	0.0025037	3.50E-23	CA, Serum-25(OH)D
LDL-C	rs10445374	17	C	T	0.514705	-0.0359766	0.00191809	1.70E-78	CA, Serum-25(OH)D
LDL-C	rs1044573	20	G	A	0.493023	0.0139532	0.00191791	3.50E-13	CA, Serum-25(OH)D
LDL-C	rs10448340	9	G	T	0.320223	-0.0166194	0.00205322	5.80E-16	CA, Serum-25(OH)D
LDL-C	rs10500834	11	T	A	0.739096	0.0241684	0.00217547	1.10E-28	CA, Serum-25(OH)D
LDL-C	rs1065853	19	T	G	0.080024	-0.545565	0.00341604	1.00E-200	CA, Serum-25(OH)D
LDL-C	rs10782922	1	A	G	0.795032	0.0217596	0.00233435	1.10E-20	CA, Serum-25(OH)D
LDL-C	rs10794579	10	C	T	0.57533	0.0201777	0.00193933	2.40E-25	CA, Serum-25(OH)D
LDL-C	rs10795464	10	A	G	0.421268	0.0144189	0.00197467	2.80E-13	CA, Serum-25(OH)D
LDL-C	rs10808709	8	C	G	0.369114	0.0142412	0.00198143	6.60E-13	CA, Serum-25(OH)D

LDL-C	rs1081105	19	C	A	0.027701	0.221489	0.00565339	1.00E-200	CA, Serum-25(OH)D
LDL-C	rs10817718	9	A	G	0.292709	-0.0123176	0.00210538	4.90E-09	CA, Serum-25(OH)D
LDL-C	rs10843391	12	G	A	0.284902	-0.0117868	0.00212478	2.90E-08	CA, Serum-25(OH)D
LDL-C	rs10846740	12	C	T	0.857972	-0.0309308	0.00279237	1.60E-28	CA, Serum-25(OH)D
LDL-C	rs10869598	9	C	T	0.694291	0.016609	0.00209097	2.00E-15	CA, Serum-25(OH)D
LDL-C	rs10877955	12	A	G	0.063489	0.028411	0.00393318	5.10E-13	CA, Serum-25(OH)D
LDL-C	rs10910476	1	T	C	0.555572	0.0156095	0.0019078	2.80E-16	CA, Serum-25(OH)D
LDL-C	rs10910522	1	A	G	0.421212	-0.0117792	0.00191916	8.40E-10	CA, Serum-25(OH)D
LDL-C	rs10930590	2	A	G	0.327715	0.0111478	0.00201374	3.10E-08	CA, Serum-25(OH)D
LDL-C	rs10953260	7	C	T	0.816012	0.0228986	0.00248097	2.70E-20	CA, Serum-25(OH)D
LDL-C	rs10953298	7	T	C	0.236207	-0.0229706	0.00226348	3.40E-24	CA, Serum-25(OH)D
LDL-C	rs11057397	12	T	C	0.336888	-0.0186053	0.00202681	4.30E-20	CA, Serum-25(OH)D
LDL-C	rs11085721	19	C	G	0.161673	-0.016179	0.0025225	1.40E-10	CA, Serum-25(OH)D
LDL-C	rs111278137	19	A	G	0.021522	-0.112997	0.00659977	1.00E-65	CA, Serum-25(OH)D
LDL-C	rs111371088	20	C	T	0.030856	-0.0353733	0.00569135	5.10E-10	CA, Serum-25(OH)D
LDL-C	rs11159071	14	T	C	0.518782	-0.0107128	0.00193087	2.90E-08	CA, Serum-25(OH)D
LDL-C	rs111990272	11	A	G	0.251688	0.0140366	0.00221541	2.40E-10	CA, Serum-25(OH)D
LDL-C	rs112201728	6	T	C	0.069764	0.0671186	0.00375323	1.60E-71	CA, Serum-25(OH)D
LDL-C	rs11226108	11	C	G	0.191458	-0.0194011	0.00243145	1.50E-15	CA, Serum-25(OH)D
LDL-C	rs11231711	11	A	G	0.057345	0.0262637	0.00411844	1.80E-10	CA, Serum-25(OH)D
LDL-C	rs11237488	11	T	C	0.127965	-0.0167239	0.00288663	6.90E-09	CA, Serum-25(OH)D
LDL-C	rs112679104	13	T	C	0.018215	-0.0475502	0.00768498	6.10E-10	CA, Serum-25(OH)D
LDL-C	rs112693563	1	C	T	0.203652	0.0152164	0.00234105	8.00E-11	CA, Serum-25(OH)D
LDL-C	rs112811239	5	C	T	0.216136	-0.0135468	0.00233135	6.20E-09	CA, Serum-25(OH)D
LDL-C	rs1133790	7	C	T	0.324978	-0.0114272	0.00207336	3.60E-08	CA, Serum-25(OH)D
LDL-C	rs1134027	8	A	G	0.369937	0.0243881	0.00199538	2.40E-34	CA, Serum-25(OH)D

LDL-C	rs1141362	13	A	G	0.59916	0.011674	0.00196406	2.80E-09	CA, Serum-25(OH)D
LDL-C	rs114165349	1	C	G	0.023223	0.0914981	0.00630213	9.20E-48	CA, Serum-25(OH)D
LDL-C	rs11429307	5	GT	G	0.190862	0.0283518	0.0024383	3.00E-31	CA, Serum-25(OH)D
LDL-C	rs114756490	4	A	G	0.013145	0.0637655	0.00868399	2.10E-13	CA, Serum-25(OH)D
LDL-C	rs114783000	4	G	A	0.045225	-0.0255399	0.00461203	3.10E-08	CA, Serum-25(OH)D
LDL-C	rs115383270	1	A	G	0.074502	0.0274859	0.00365226	5.20E-14	CA, Serum-25(OH)D
LDL-C	rs11568318	2	A	C	0.06632	0.0336663	0.00379062	6.60E-19	CA, Serum-25(OH)D
LDL-C	rs11591147	1	T	G	0.017455	-0.457849	0.0071963	1.00E-200	CA, Serum-25(OH)D
LDL-C	rs11601507	11	A	C	0.069337	0.0493409	0.00371368	2.80E-40	CA, Serum-25(OH)D
LDL-C	rs11621792	14	T	C	0.452899	0.0267004	0.00194171	5.00E-43	CA, Serum-25(OH)D
LDL-C	rs11641811	16	A	C	0.522685	0.0293316	0.00191805	8.60E-53	CA, Serum-25(OH)D
LDL-C	rs11646091	16	C	T	0.041703	-0.0342311	0.00479134	9.00E-13	CA, Serum-25(OH)D
LDL-C	rs11652501	17	C	G	0.638424	-0.0115693	0.00199198	6.30E-09	CA, Serum-25(OH)D
LDL-C	rs1169288	12	C	A	0.315761	0.0383025	0.00207161	2.50E-76	CA, Serum-25(OH)D
LDL-C	rs117733303	6	G	A	0.018489	0.155275	0.00707981	1.30E-106	CA, Serum-25(OH)D
LDL-C	rs11789603	9	T	C	0.107996	0.0309585	0.00308951	1.20E-23	CA, Serum-25(OH)D
LDL-C	rs11846704	14	T	C	0.26884	-0.0128621	0.00217476	3.30E-09	CA, Serum-25(OH)D
LDL-C	rs11895352	2	T	C	0.477596	-0.0229815	0.00189418	7.10E-34	CA, Serum-25(OH)D
LDL-C	rs11983987	7	G	A	0.16648	0.0217127	0.00256894	2.90E-17	CA, Serum-25(OH)D
LDL-C	rs11997161	8	C	T	0.517654	0.0106387	0.00191608	2.80E-08	CA, Serum-25(OH)D
LDL-C	rs12027388	1	G	C	0.20785	-0.017145	0.00232306	1.60E-13	CA, Serum-25(OH)D
LDL-C	rs12054451	3	G	T	0.259243	0.0191916	0.00219453	2.20E-18	CA, Serum-25(OH)D
LDL-C	rs12179053	6	T	C	0.252046	-0.0263752	0.00219871	3.70E-33	CA, Serum-25(OH)D
LDL-C	rs12202204	6	G	A	0.313736	0.0139897	0.00205564	1.00E-11	CA, Serum-25(OH)D
LDL-C	rs12212146	6	C	T	0.07415	-0.0237755	0.00373812	2.00E-10	CA, Serum-25(OH)D
LDL-C	rs12271333	11	C	A	0.139018	0.01835	0.00276569	3.20E-11	CA, Serum-25(OH)D

LDL-C	rs1229984	4	C	T	0.97343	0.0807339	0.00604443	1.10E-40	CA, Serum-25(OH)D
LDL-C	rs12320328	12	G	A	0.084231	-0.022934	0.00345496	3.20E-11	CA, Serum-25(OH)D
LDL-C	rs12445804	16	A	G	0.074873	0.0363752	0.00367779	4.60E-23	CA, Serum-25(OH)D
LDL-C	rs12453219	17	G	C	0.146378	-0.0191522	0.00271222	1.60E-12	CA, Serum-25(OH)D
LDL-C	rs12469822	2	A	G	0.598483	-0.0112645	0.00193914	6.30E-09	CA, Serum-25(OH)D
LDL-C	rs12471768	2	C	T	0.704276	0.014803	0.00207156	8.90E-13	CA, Serum-25(OH)D
LDL-C	rs1250259	2	A	T	0.736205	0.0157213	0.00214831	2.50E-13	CA, Serum-25(OH)D
LDL-C	rs1260326	2	C	T	0.60459	-0.0529385	0.00193189	2.60E-165	CA, Serum-25(OH)D
LDL-C	rs12614487	2	T	C	0.075512	-0.0276135	0.00357121	1.10E-14	CA, Serum-25(OH)D
LDL-C	rs12638256	3	G	A	0.534555	-0.0155767	0.00192518	5.90E-16	CA, Serum-25(OH)D
LDL-C	rs12655342	5	G	T	0.141916	-0.0163171	0.00274509	2.80E-09	CA, Serum-25(OH)D
LDL-C	rs12665537	6	G	A	0.330225	0.0181891	0.00203422	3.80E-19	CA, Serum-25(OH)D
LDL-C	rs12684235	9	A	G	0.063027	-0.0217118	0.00396064	4.20E-08	CA, Serum-25(OH)D
LDL-C	rs12740374	1	T	G	0.221249	-0.162741	0.00227119	1.00E-200	CA, Serum-25(OH)D
LDL-C	rs12891477	14	T	C	0.368809	0.0139634	0.00200214	3.10E-12	CA, Serum-25(OH)D
LDL-C	rs12916	5	C	T	0.400373	0.0811025	0.00195048	1.00E-200	CA, Serum-25(OH)D
LDL-C	rs12977362	19	T	C	0.408464	0.0213492	0.00189345	1.70E-29	CA, Serum-25(OH)D
LDL-C	rs12985907	19	A	G	0.231381	-0.0158515	0.00220062	5.90E-13	CA, Serum-25(OH)D
LDL-C	rs12986248	19	G	A	0.593991	-0.0138055	0.0018929	3.00E-13	CA, Serum-25(OH)D
LDL-C	rs13076933	3	G	T	0.259454	-0.0322061	0.0021932	8.10E-49	CA, Serum-25(OH)D
LDL-C	rs13107325	4	T	C	0.074856	-0.0323431	0.00364345	6.90E-19	CA, Serum-25(OH)D
LDL-C	rs13108218	4	G	A	0.615429	-0.0275204	0.00198645	1.20E-43	CA, Serum-25(OH)D
LDL-C	rs13255048	8	A	G	0.138453	0.0332894	0.00276766	2.50E-33	CA, Serum-25(OH)D
LDL-C	rs13359927	5	A	T	0.114359	0.0199155	0.00301355	3.90E-11	CA, Serum-25(OH)D
LDL-C	rs1336455	10	A	G	0.358514	-0.0116911	0.00199733	4.80E-09	CA, Serum-25(OH)D
LDL-C	rs1349852	4	C	A	0.475358	0.0165743	0.00193311	1.00E-17	CA, Serum-25(OH)D

LDL-C	rs1362965	12	T	C	0.627819	0.0134411	0.00198053	1.10E-11	CA, Serum-25(OH)D
LDL-C	rs1375131	2	C	T	0.278463	0.0182348	0.00218398	6.90E-17	CA, Serum-25(OH)D
LDL-C	rs13796	1	C	T	0.135064	0.0163122	0.00275844	3.30E-09	CA, Serum-25(OH)D
LDL-C	rs138352	22	G	T	0.658569	-0.0163988	0.00203396	7.50E-16	CA, Serum-25(OH)D
LDL-C	rs141469619	11	G	A	0.009952	0.107803	0.0101854	3.50E-26	CA, Serum-25(OH)D
LDL-C	rs141783576	6	C	G	0.072227	0.0312637	0.00375077	7.70E-17	CA, Serum-25(OH)D
LDL-C	rs1434282	1	T	C	0.72482	0.0128446	0.00212002	1.40E-09	CA, Serum-25(OH)D
LDL-C	rs143455776	5	C	T	0.033035	-0.0552804	0.00536521	6.80E-25	CA, Serum-25(OH)D
LDL-C	rs146433259	5	T	C	0.011692	-0.0577472	0.00952965	1.40E-09	CA, Serum-25(OH)D
LDL-C	rs146947088	7	TC	T	0.186396	0.0224344	0.00246199	8.10E-20	CA, Serum-25(OH)D
LDL-C	rs147510296	7	A	G	0.023031	0.0379851	0.00655185	6.70E-09	CA, Serum-25(OH)D
LDL-C	rs147539187	6	G	C	0.07348	-0.0311891	0.00366627	1.80E-17	CA, Serum-25(OH)D
LDL-C	rs14842	22	T	A	0.110337	0.0197196	0.00307642	1.50E-10	CA, Serum-25(OH)D
LDL-C	rs1489501	11	C	G	0.879895	-0.0173911	0.00294144	3.40E-09	CA, Serum-25(OH)D
LDL-C	rs1497406	1	G	A	0.579087	0.0192839	0.00190754	5.00E-24	CA, Serum-25(OH)D
LDL-C	rs150169808	7	GCTCT	G	0.216685	0.0462388	0.00233114	1.50E-87	CA, Serum-25(OH)D
LDL-C	rs150474434	2	A	G	0.101364	-0.0433941	0.00315131	3.90E-43	CA, Serum-25(OH)D
LDL-C	rs150783681	4	C	G	0.020706	0.0451238	0.00674523	2.20E-11	CA, Serum-25(OH)D
LDL-C	rs151009137	16	A	G	0.00977	-0.0555215	0.00986246	1.80E-08	CA, Serum-25(OH)D
LDL-C	rs15285	8	T	C	0.286145	-0.0284133	0.00211509	3.80E-41	CA, Serum-25(OH)D
LDL-C	rs1532085	15	G	A	0.613593	-0.0264105	0.00197206	6.70E-41	CA, Serum-25(OH)D
LDL-C	rs1544155	6	A	C	0.421645	-0.0208176	0.001932	4.50E-27	CA, Serum-25(OH)D
LDL-C	rs1571790	9	T	A	0.613818	-0.0174563	0.00197637	1.00E-18	CA, Serum-25(OH)D
LDL-C	rs165722	22	T	C	0.515715	0.0114996	0.00193704	2.90E-09	CA, Serum-25(OH)D
LDL-C	rs16988410	22	C	T	0.046227	0.0265452	0.0045791	6.70E-09	CA, Serum-25(OH)D

LDL-C	rs17031776	2	T	C	0.240518	0.0151406	0.00221634	8.40E-12	CA, Serum-25(OH)D
LDL-C	rs17036085	1	G	A	0.012152	-0.0774542	0.00862011	2.60E-19	CA, Serum-25(OH)D
LDL-C	rs17050272	2	A	G	0.409538	-0.0260593	0.00192093	6.40E-42	CA, Serum-25(OH)D
LDL-C	rs17137472	7	T	C	0.500916	0.0110657	0.00191756	7.90E-09	CA, Serum-25(OH)D
LDL-C	rs174564	11	G	A	0.348974	-0.0407562	0.00200581	8.70E-92	CA, Serum-25(OH)D
LDL-C	rs17476364	10	C	T	0.108482	-0.033023	0.00308568	1.00E-26	CA, Serum-25(OH)D
LDL-C	rs17580	14	A	T	0.047695	0.0514106	0.00450828	4.00E-30	CA, Serum-25(OH)D
LDL-C	rs17712208	1	A	T	0.035215	0.0296226	0.00511677	7.10E-09	CA, Serum-25(OH)D
LDL-C	rs17875609	19	T	C	0.024109	-0.0629727	0.00624071	6.10E-24	CA, Serum-25(OH)D
LDL-C	rs17883691	21	G	GATAA	0.044252	0.0325059	0.00467574	3.60E-12	CA, Serum-25(OH)D
LDL-C	rs1800562	6	A	G	0.077103	-0.0645775	0.00358339	1.30E-72	CA, Serum-25(OH)D
LDL-C	rs1800961	20	T	C	0.03097	-0.0755416	0.0055309	1.80E-42	CA, Serum-25(OH)D
LDL-C	rs1801689	17	C	A	0.030367	0.0832757	0.00557678	2.00E-50	CA, Serum-25(OH)D
LDL-C	rs185263492	2	A	T	0.154191	0.0242368	0.0026198	2.20E-20	CA, Serum-25(OH)D
LDL-C	rs1883711	20	C	G	0.031445	0.156174	0.0056001	3.80E-171	CA, Serum-25(OH)D
LDL-C	rs1896995	10	T	C	0.485636	0.0127252	0.00192851	4.20E-11	CA, Serum-25(OH)D
LDL-C	rs1963676	21	T	C	0.564604	0.0189758	0.00194707	1.90E-22	CA, Serum-25(OH)D
LDL-C	rs203273	4	T	C	0.656059	0.0139005	0.00201444	5.20E-12	CA, Serum-25(OH)D
LDL-C	rs2066714	9	C	T	0.12843	0.0292953	0.00285967	1.30E-24	CA, Serum-25(OH)D
LDL-C	rs2068888	10	A	G	0.450637	-0.0275689	0.00192443	1.50E-46	CA, Serum-25(OH)D
LDL-C	rs2073547	7	G	A	0.183957	0.0488167	0.00245287	3.90E-88	CA, Serum-25(OH)D
LDL-C	rs2081048	19	C	T	0.345708	-0.0111302	0.00196305	1.40E-08	CA, Serum-25(OH)D
LDL-C	rs2082511	3	G	T	0.705772	-0.011993	0.0021085	1.30E-08	CA, Serum-25(OH)D
LDL-C	rs2122982	12	A	G	0.239282	-0.0230697	0.00224176	7.70E-25	CA, Serum-25(OH)D
LDL-C	rs213499	1	C	A	0.473689	-0.0112731	0.00189239	2.60E-09	CA, Serum-25(OH)D

LDL-C	rs216140	5	T	C	0.720191	0.0124318	0.00214428	6.70E-09	CA, Serum-25(OH)D
LDL-C	rs2193006	12	G	T	0.490986	0.0123221	0.00191411	1.20E-10	CA, Serum-25(OH)D
LDL-C	rs2220729	12	A	G	0.346096	0.0128201	0.00201258	1.90E-10	CA, Serum-25(OH)D
LDL-C	rs2238162	13	T	C	0.523181	-0.0251239	0.00192262	5.00E-39	CA, Serum-25(OH)D
LDL-C	rs224424	20	G	A	0.211657	-0.0260843	0.00235348	1.50E-28	CA, Serum-25(OH)D
LDL-C	rs2250802	10	A	G	0.724548	-0.0252871	0.00214648	4.90E-32	CA, Serum-25(OH)D
LDL-C	rs2347699	7	A	T	0.279149	-0.0121069	0.00217157	2.50E-08	CA, Serum-25(OH)D
LDL-C	rs2382818	2	T	A	0.416806	0.0115756	0.00192906	2.00E-09	CA, Serum-25(OH)D
LDL-C	rs2384008	2	G	A	0.363764	0.0140236	0.0019676	1.00E-12	CA, Serum-25(OH)D
LDL-C	rs2395617	6	C	A	0.877312	-0.0177128	0.0029086	1.10E-09	CA, Serum-25(OH)D
LDL-C	rs2413338	22	T	C	0.633864	-0.0123633	0.00200533	7.00E-10	CA, Serum-25(OH)D
LDL-C	rs2439222	3	T	C	0.371275	-0.013561	0.00197958	7.40E-12	CA, Serum-25(OH)D
LDL-C	rs246564	5	G	T	0.182681	0.0162867	0.00247382	4.60E-11	CA, Serum-25(OH)D
LDL-C	rs2510344	18	C	T	0.490158	-0.0116266	0.00192603	1.60E-09	CA, Serum-25(OH)D
LDL-C	rs2522062	5	G	A	0.187574	-0.0204169	0.00244858	7.50E-17	CA, Serum-25(OH)D
LDL-C	rs2577611	2	T	G	0.190609	0.0184959	0.00239908	1.30E-14	CA, Serum-25(OH)D
LDL-C	rs2618567	20	T	G	0.65876	-0.036634	0.0020236	3.00E-73	CA, Serum-25(OH)D
LDL-C	rs262680	1	T	C	0.424494	-0.0130691	0.00191099	8.00E-12	CA, Serum-25(OH)D
LDL-C	rs267733	1	G	A	0.160889	-0.0187326	0.00256622	2.90E-13	CA, Serum-25(OH)D
LDL-C	rs2737245	8	T	G	0.279516	-0.0328372	0.00213766	3.00E-53	CA, Serum-25(OH)D
LDL-C	rs2738447	19	C	A	0.592593	0.0591662	0.00188722	1.00E-200	CA, Serum-25(OH)D
LDL-C	rs2740488	9	C	A	0.265267	-0.0374266	0.00217187	1.50E-66	CA, Serum-25(OH)D
LDL-C	rs2773807	9	G	A	0.533523	-0.0117053	0.00192376	1.20E-09	CA, Serum-25(OH)D
LDL-C	rs28406917	7	T	C	0.427878	0.0152282	0.00193928	4.10E-15	CA, Serum-25(OH)D
LDL-C	rs28471687	8	G	A	0.07839	0.020205	0.00355787	1.40E-08	CA, Serum-25(OH)D
LDL-C	rs28478252	3	C	A	0.755639	0.0219291	0.00222734	7.20E-23	CA, Serum-25(OH)D

LDL-C	rs28601761	8	G	C	0.418796	-0.0919577	0.00195753	1.00E-200	CA, Serum-25(OH)D
LDL-C	rs28615248	8	C	T	0.196344	0.0300403	0.00241463	1.60E-35	CA, Serum-25(OH)D
LDL-C	rs28807203	19	C	A	0.048982	-0.13655	0.00430913	1.00E-200	CA, Serum-25(OH)D
LDL-C	rs2911987	8	G	A	0.679945	0.0136165	0.0020516	3.20E-11	CA, Serum-25(OH)D
LDL-C	rs2972146	2	T	G	0.647469	0.0112509	0.00197461	1.20E-08	CA, Serum-25(OH)D
LDL-C	rs308915	2	C	A	0.5092	-0.0119831	0.00194322	7.00E-10	CA, Serum-25(OH)D
LDL-C	rs31226	5	C	T	0.606043	-0.0109438	0.00196562	2.60E-08	CA, Serum-25(OH)D
LDL-C	rs3127580	6	T	C	0.155458	0.0545401	0.00263476	3.40E-95	CA, Serum-25(OH)D
LDL-C	rs34023524	9	C	T	0.078193	0.0350765	0.00356721	8.10E-23	CA, Serum-25(OH)D
LDL-C	rs34042070	16	G	C	0.187636	0.0700152	0.00246375	1.20E-177	CA, Serum-25(OH)D
LDL-C	rs34815285	1	C	T	0.466702	0.0125348	0.00189681	3.90E-11	CA, Serum-25(OH)D
LDL-C	rs34914463	17	C	T	0.131437	-0.0171311	0.00283229	1.50E-09	CA, Serum-25(OH)D
LDL-C	rs35081008	19	T	C	0.147847	-0.0322757	0.00262353	8.80E-35	CA, Serum-25(OH)D
LDL-C	rs35243054	1	T	G	0.44237	-0.0292634	0.00189744	1.20E-53	CA, Serum-25(OH)D
LDL-C	rs35363532	7	CT	C	0.491703	-0.0186209	0.00191751	2.70E-22	CA, Serum-25(OH)D
LDL-C	rs35468353	16	G	A	0.376823	0.0193754	0.00197527	1.00E-22	CA, Serum-25(OH)D
LDL-C	rs35570186	20	A	G	0.040443	0.0299265	0.00510483	4.60E-09	CA, Serum-25(OH)D
LDL-C	rs35814089	6	C	T	0.491442	0.0112247	0.00191091	4.30E-09	CA, Serum-25(OH)D
LDL-C	rs369298568	18	C	T	0.123843	0.020139	0.00291926	5.20E-12	CA, Serum-25(OH)D
LDL-C	rs3732359	3	A	G	0.780531	-0.0160503	0.00231512	4.10E-12	CA, Serum-25(OH)D
LDL-C	rs3775228	4	T	C	0.399891	0.0185041	0.00196215	4.10E-21	CA, Serum-25(OH)D
LDL-C	rs3780181	9	G	A	0.067554	-0.0385591	0.00384155	1.00E-23	CA, Serum-25(OH)D
LDL-C	rs3820897	2	C	T	0.819888	-0.0189654	0.00248214	2.20E-14	CA, Serum-25(OH)D
LDL-C	rs3824667	10	G	A	0.827136	0.0141513	0.00255074	2.90E-08	CA, Serum-25(OH)D
LDL-C	rs41280463	4	A	G	0.165185	-0.0196581	0.00257945	2.50E-14	CA, Serum-25(OH)D
LDL-C	rs4148810	7	T	C	0.181194	-0.0209187	0.00248633	4.00E-17	CA, Serum-25(OH)D

LDL-C	rs41785	7	A	C	0.417318	-0.0111724	0.00194196	8.80E-09	CA, Serum-25(OH)D
LDL-C	rs4299376	2	T	G	0.677001	-0.0779529	0.00202069	1.00E-200	CA, Serum-25(OH)D
LDL-C	rs4307732	11	A	G	0.105934	0.0573325	0.00311171	8.30E-76	CA, Serum-25(OH)D
LDL-C	rs4374942	7	C	T	0.074128	0.0259845	0.00364859	1.10E-12	CA, Serum-25(OH)D
LDL-C	rs4607926	1	G	A	0.216259	-0.0125245	0.002292	4.60E-08	CA, Serum-25(OH)D
LDL-C	rs4616688	3	T	G	0.529015	-0.013898	0.00192044	4.60E-13	CA, Serum-25(OH)D
LDL-C	rs463599	6	T	G	0.141505	0.0531173	0.0027429	1.50E-83	CA, Serum-25(OH)D
LDL-C	rs4689088	4	A	G	0.618766	0.0146061	0.001972	1.30E-13	CA, Serum-25(OH)D
LDL-C	rs472495	1	T	G	0.649347	0.0546333	0.00197866	8.09E-168	CA, Serum-25(OH)D
LDL-C	rs4751996	10	G	A	0.519157	0.0164643	0.00191883	9.50E-18	CA, Serum-25(OH)D
LDL-C	rs4841146	8	A	C	0.209172	0.017184	0.00235883	3.20E-13	CA, Serum-25(OH)D
LDL-C	rs4860987	4	T	A	0.259533	0.0339193	0.00230742	6.40E-49	CA, Serum-25(OH)D
LDL-C	rs4935356	6	A	T	0.244532	0.0363583	0.00234984	5.30E-54	CA, Serum-25(OH)D
LDL-C	rs4955153	3	A	T	0.047345	-0.0310322	0.00453969	8.20E-12	CA, Serum-25(OH)D
LDL-C	rs542049	1	C	T	0.330496	-0.0207965	0.00201843	6.80E-25	CA, Serum-25(OH)D
LDL-C	rs55637835	1	T	C	0.120974	-0.0239567	0.00294683	4.30E-16	CA, Serum-25(OH)D
LDL-C	rs55691818	19	G	C	0.186729	0.0171018	0.00238008	6.70E-13	CA, Serum-25(OH)D
LDL-C	rs55714927	17	T	C	0.190707	-0.0397688	0.00243653	6.90E-60	CA, Serum-25(OH)D
LDL-C	rs557933	1	C	A	0.520059	0.044949	0.00189236	1.00E-124	CA, Serum-25(OH)D
LDL-C	rs56000661	17	C	A	0.311664	-0.0207389	0.002068	1.10E-23	CA, Serum-25(OH)D
LDL-C	rs56265089	16	A	G	0.029154	-0.0487402	0.00569811	1.20E-17	CA, Serum-25(OH)D
LDL-C	rs57159332	14	C	T	0.138356	0.0223575	0.00281666	2.10E-15	CA, Serum-25(OH)D
LDL-C	rs58148580	4	T	C	0.109885	0.0198041	0.00305834	9.50E-11	CA, Serum-25(OH)D
LDL-C	rs58542926	19	T	C	0.074738	-0.132092	0.00352508	1.00E-200	CA, Serum-25(OH)D
LDL-C	rs59328596	8	A	G	0.14816	-0.0234829	0.00268649	2.30E-18	CA, Serum-25(OH)D
LDL-C	rs603424	10	A	G	0.172083	0.021614	0.00254438	2.00E-17	CA, Serum-25(OH)D

LDL-C	rs60847460	10	T	C	0.143391	-0.0159145	0.00274091	6.40E-09	CA, Serum-25(OH)D
LDL-C	rs60852193	3	A	G	0.240829	0.0128346	0.00224731	1.10E-08	CA, Serum-25(OH)D
LDL-C	rs608994	20	A	G	0.665415	0.0116834	0.00205876	1.40E-08	CA, Serum-25(OH)D
LDL-C	rs6090040	20	C	A	0.521087	-0.0170833	0.00192902	8.30E-19	CA, Serum-25(OH)D
LDL-C	rs6093446	20	A	G	0.287664	0.0324432	0.00211743	5.50E-53	CA, Serum-25(OH)D
LDL-C	rs6139114	20	G	C	0.08371	-0.0212079	0.00346407	9.20E-10	CA, Serum-25(OH)D
LDL-C	rs61468422	11	A	G	0.191472	-0.0179139	0.00243724	2.00E-13	CA, Serum-25(OH)D
LDL-C	rs61750929	9	T	C	0.055749	-0.0243212	0.00418258	6.10E-09	CA, Serum-25(OH)D
LDL-C	rs61754230	12	T	C	0.019774	0.0636057	0.006869	2.00E-20	CA, Serum-25(OH)D
LDL-C	rs61871243	11	A	G	0.124702	0.018856	0.00288791	6.60E-11	CA, Serum-25(OH)D
LDL-C	rs62008532	15	C	T	0.436399	0.0112369	0.00193792	6.70E-09	CA, Serum-25(OH)D
LDL-C	rs62072466	17	A	T	0.190259	0.0178362	0.00243944	2.60E-13	CA, Serum-25(OH)D
LDL-C	rs62075819	17	T	C	0.355414	0.0134751	0.00200233	1.70E-11	CA, Serum-25(OH)D
LDL-C	rs62219001	21	T	G	0.017155	-0.0530653	0.00748012	1.30E-12	CA, Serum-25(OH)D
LDL-C	rs62305783	4	T	C	0.095928	0.0218533	0.00332953	5.30E-11	CA, Serum-25(OH)D
LDL-C	rs62509311	8	T	A	0.282035	-0.0159506	0.00212802	6.60E-14	CA, Serum-25(OH)D
LDL-C	rs62544387	9	G	C	0.226775	-0.0148144	0.00228849	9.60E-11	CA, Serum-25(OH)D
LDL-C	rs6602909	13	C	T	0.327024	0.0296194	0.00205245	3.30E-47	CA, Serum-25(OH)D
LDL-C	rs6606731	12	A	T	0.192089	0.0159681	0.00242777	4.80E-11	CA, Serum-25(OH)D
LDL-C	rs666334	2	A	G	0.287282	0.0141091	0.00209008	1.50E-11	CA, Serum-25(OH)D
LDL-C	rs6709904	2	G	A	0.112245	-0.0505577	0.00299811	8.40E-64	CA, Serum-25(OH)D
LDL-C	rs67560206	5	C	G	0.078099	0.0276835	0.00355832	7.30E-15	CA, Serum-25(OH)D
LDL-C	rs6785233	3	G	T	0.079587	0.0277941	0.00353741	3.90E-15	CA, Serum-25(OH)D
LDL-C	rs67854369	13	GT	G	0.696758	-0.0124182	0.00211554	4.40E-09	CA, Serum-25(OH)D
LDL-C	rs68023264	17	A	G	0.142586	-0.021396	0.00273892	5.60E-15	CA, Serum-25(OH)D
LDL-C	rs6882076	5	C	T	0.634646	0.0455741	0.00198294	6.90E-117	CA, Serum-25(OH)D

LDL-C	rs6896005	5	C	T	0.550223	0.0199662	0.00192092	2.60E-25	CA, Serum-25(OH)D
LDL-C	rs7005453	8	A	G	0.210378	0.0135228	0.00235313	9.10E-09	CA, Serum-25(OH)D
LDL-C	rs704	17	A	G	0.475436	0.0192243	0.00191489	1.00E-23	CA, Serum-25(OH)D
LDL-C	rs7108486	11	C	T	0.023659	-0.0439372	0.00634523	4.40E-12	CA, Serum-25(OH)D
LDL-C	rs71311871	3	G	A	0.082975	-0.0400404	0.00347381	9.70E-31	CA, Serum-25(OH)D
LDL-C	rs7136799	12	T	C	0.769766	0.0182634	0.00228401	1.30E-15	CA, Serum-25(OH)D
LDL-C	rs7187512	16	G	A	0.43119	-0.0109739	0.00193603	1.40E-08	CA, Serum-25(OH)D
LDL-C	rs7204	5	C	T	0.209209	-0.0160365	0.00234828	8.50E-12	CA, Serum-25(OH)D
LDL-C	rs72631343	17	G	C	0.128742	-0.04404	0.00285567	1.20E-53	CA, Serum-25(OH)D
LDL-C	rs72784625	2	G	T	0.2611	-0.0125258	0.00215358	6.00E-09	CA, Serum-25(OH)D
LDL-C	rs72818967	2	A	G	0.129312	-0.0161257	0.00282958	1.20E-08	CA, Serum-25(OH)D
LDL-C	rs7300192	12	G	A	0.638124	-0.0174791	0.00198948	1.60E-18	CA, Serum-25(OH)D
LDL-C	rs73015024	19	T	G	0.118663	-0.219509	0.00286668	1.00E-200	CA, Serum-25(OH)D
LDL-C	rs73075609	20	T	C	0.026882	0.0490853	0.0059639	1.90E-16	CA, Serum-25(OH)D
LDL-C	rs73118985	20	C	T	0.042108	-0.0393312	0.00477345	1.70E-16	CA, Serum-25(OH)D
LDL-C	rs7316368	12	C	T	0.730086	0.0157077	0.00216882	4.40E-13	CA, Serum-25(OH)D
LDL-C	rs738170	22	T	C	0.613095	0.0128409	0.00197206	7.40E-11	CA, Serum-25(OH)D
LDL-C	rs739431	3	G	A	0.137845	-0.0152048	0.00277482	4.30E-08	CA, Serum-25(OH)D
LDL-C	rs74035509	16	T	C	0.079132	0.0265066	0.00358126	1.30E-13	CA, Serum-25(OH)D
LDL-C	rs74341202	3	A	G	0.05513	-0.0476176	0.00420924	1.10E-29	CA, Serum-25(OH)D
LDL-C	rs7516453	1	G	C	0.271123	-0.0123612	0.00212883	6.40E-09	CA, Serum-25(OH)D
LDL-C	rs7534572	1	G	C	0.647535	0.0550705	0.00197515	4.50E-171	CA, Serum-25(OH)D
LDL-C	rs7551451	1	G	A	0.200397	-0.0216138	0.00236124	5.50E-20	CA, Serum-25(OH)D
LDL-C	rs75588192	12	A	G	0.137806	0.0197578	0.00282447	2.60E-12	CA, Serum-25(OH)D
LDL-C	rs7569317	2	C	T	0.530802	0.0254243	0.00189143	3.40E-41	CA, Serum-25(OH)D
LDL-C	rs75735512	15	G	A	0.044644	0.0333884	0.00466944	8.70E-13	CA, Serum-25(OH)D

LDL-C	rs7603427	2	T	C	0.533049	0.0138321	0.00189786	3.10E-13	CA, Serum-25(OH)D
LDL-C	rs76147254	1	C	G	0.070869	-0.0204889	0.00375412	4.80E-08	CA, Serum-25(OH)D
LDL-C	rs76769360	4	A	G	0.067546	-0.0235457	0.00382146	7.20E-10	CA, Serum-25(OH)D
LDL-C	rs76775468	8	C	T	0.04021	0.0286419	0.00485427	3.60E-09	CA, Serum-25(OH)D
LDL-C	rs76895963	12	G	T	0.020712	-0.0709513	0.00741143	1.00E-21	CA, Serum-25(OH)D
LDL-C	rs7695536	4	C	T	0.552242	-0.011118	0.00193366	8.90E-09	CA, Serum-25(OH)D
LDL-C	rs76984261	19	T	A	0.056098	0.0408799	0.00413894	5.20E-23	CA, Serum-25(OH)D
LDL-C	rs7707394	5	A	G	0.356529	0.0511164	0.00199404	6.30E-145	CA, Serum-25(OH)D
LDL-C	rs7746081	6	A	G	0.303849	-0.0334797	0.00207864	2.30E-58	CA, Serum-25(OH)D
LDL-C	rs77542162	17	G	A	0.022588	0.17887	0.00645012	2.90E-169	CA, Serum-25(OH)D
LDL-C	rs77960347	18	G	A	0.013347	0.0851286	0.00836788	2.60E-24	CA, Serum-25(OH)D
LDL-C	rs78058190	2	A	G	0.050474	0.0270032	0.00486519	2.90E-08	CA, Serum-25(OH)D
LDL-C	rs78173576	17	G	T	0.036349	-0.0341094	0.00510931	2.50E-11	CA, Serum-25(OH)D
LDL-C	rs78186330	17	A	G	0.208567	0.0182684	0.00235849	9.50E-15	CA, Serum-25(OH)D
LDL-C	rs78677597	6	C	A	0.244328	-0.0210513	0.00222461	3.00E-21	CA, Serum-25(OH)D
LDL-C	rs7902032	10	T	C	0.240136	0.0127474	0.00225205	1.50E-08	CA, Serum-25(OH)D
LDL-C	rs7908745	10	G	A	0.314965	0.0134821	0.00206234	6.30E-11	CA, Serum-25(OH)D
LDL-C	rs7960935	12	C	T	0.436504	-0.0144037	0.00193922	1.10E-13	CA, Serum-25(OH)D
LDL-C	rs799157	7	C	T	0.956415	-0.0468863	0.00468473	1.40E-23	CA, Serum-25(OH)D
LDL-C	rs8005362	14	G	A	0.472304	0.0201149	0.00192716	1.70E-25	CA, Serum-25(OH)D
LDL-C	rs80162990	6	G	A	0.180616	-0.0141107	0.00255063	3.20E-08	CA, Serum-25(OH)D
LDL-C	rs80276949	10	A	G	0.022696	0.0468997	0.00644767	3.50E-13	CA, Serum-25(OH)D
LDL-C	rs8030799	15	C	G	0.848403	-0.0160446	0.0026849	2.30E-09	CA, Serum-25(OH)D
LDL-C	rs8103315	19	A	C	0.134637	0.0630036	0.00279272	1.10E-112	CA, Serum-25(OH)D
LDL-C	rs867772	1	G	A	0.684136	0.0311833	0.0020318	3.70E-53	CA, Serum-25(OH)D
LDL-C	rs9287908	2	T	C	0.603264	-0.0251497	0.00193692	1.50E-38	CA, Serum-25(OH)D

LDL-C	rs9297994	8	A	G	0.664237	-0.0411393	0.0020233	6.60E-92	CA, Serum-25(OH)D
LDL-C	rs934197	2	A	G	0.336027	0.112288	0.00200093	1.00E-200	CA, Serum-25(OH)D
LDL-C	rs9389268	6	G	A	0.260018	-0.0276924	0.00218455	8.00E-37	CA, Serum-25(OH)D
LDL-C	rs9471975	6	C	T	0.582413	-0.0146922	0.00193549	3.20E-14	CA, Serum-25(OH)D
LDL-C	rs9604529	13	G	A	0.190458	-0.020138	0.00244294	1.70E-16	CA, Serum-25(OH)D
LDL-C	rs9616822	22	A	G	0.35155	0.0158614	0.00201459	3.50E-15	CA, Serum-25(OH)D
LDL-C	rs964184	11	C	G	0.866609	-0.106826	0.00280881	1.00E-200	CA, Serum-25(OH)D
LDL-C	rs9673065	15	G	T	0.573986	0.0130176	0.00194665	2.30E-11	CA, Serum-25(OH)D
LDL-C	rs969075	20	C	T	0.664572	0.0132764	0.00204072	7.70E-11	CA, Serum-25(OH)D
LDL-C	rs9834932	3	G	A	0.08894	-0.0379173	0.00335939	1.50E-29	CA, Serum-25(OH)D
LDL-C	rs9871402	3	C	A	0.570079	-0.0116615	0.00193675	1.70E-09	CA, Serum-25(OH)D
LDL-C	rs9894946	17	G	A	0.841519	-0.0235826	0.0026656	9.00E-19	CA, Serum-25(OH)D
LDL-C	rs9915658	17	A	G	0.27864	0.0144735	0.0021492	1.60E-11	CA, Serum-25(OH)D
LDL-C	rs9987289	8	G	A	0.908757	0.0567992	0.00331823	2.20E-44	CA, Serum-25(OH)D
HDL-C	rs10012624	4	A	C	0.380215	-0.0195014	0.00195683	2.15E-23	CA, Serum-25(OH)D
HDL-C	rs10105127	8	C	T	0.329038	0.0130101	0.00207952	3.94E-10	CA, Serum-25(OH)D
HDL-C	rs10233430	7	C	T	0.563358	-0.0198275	0.00197228	8.90E-24	CA, Serum-25(OH)D
HDL-C	rs1037378	11	A	G	0.244559	-0.0136856	0.00196674	3.44E-12	CA, Serum-25(OH)D
HDL-C	rs1045242	5	G	A	0.748823	0.0175732	0.00220242	1.47E-15	CA, Serum-25(OH)D
HDL-C	rs10460585	2	G	T	0.406121	0.0128916	0.00200313	1.23E-10	CA, Serum-25(OH)D
HDL-C	rs1047891	2	A	C	0.212476	-0.0164271	0.00209883	5.01E-15	CA, Serum-25(OH)D
HDL-C	rs10504477	8	C	T	0.818988	-0.0166671	0.00200298	8.71E-17	CA, Serum-25(OH)D
HDL-C	rs1051613	4	A	G	0.514705	0.0172912	0.00196223	1.23E-18	CA, Serum-25(OH)D
HDL-C	rs1052373	11	T	C	0.493023	0.0448586	0.00209381	7.93E-102	CA, Serum-25(OH)D
HDL-C	rs1057517	8	A	G	0.320223	0.0322764	0.00198217	1.30E-59	CA, Serum-25(OH)D
HDL-C	rs10786114	10	T	C	0.739096	0.0262097	0.00294567	5.70E-19	CA, Serum-25(OH)D

HDL-C	rs10876447	12	A	G	0.080024	-0.0184203	0.00270014	8.98E-12	CA, Serum-25(OH)D
HDL-C	rs11009262	10	T	G	0.795032	-0.0247379	0.00418049	3.27E-09	CA, Serum-25(OH)D
HDL-C	rs11021232	11	C	T	0.57533	-0.0195005	0.00256538	2.93E-14	CA, Serum-25(OH)D
HDL-C	rs11045172	12	C	A	0.421268	0.0293385	0.00246733	1.32E-32	CA, Serum-25(OH)D
HDL-C	rs11057692	12	G	A	0.369114	-0.0256661	0.00232515	2.49E-28	CA, Serum-25(OH)D
HDL-C	rs11076174	16	C	T	0.027701	-0.13977	0.00370879	1.00E-200	CA, Serum-25(OH)D
HDL-C	rs112001035	17	A	G	0.292709	-0.0499704	0.00412742	9.71E-34	CA, Serum-25(OH)D
HDL-C	rs11218738	11	A	G	0.284902	0.0236964	0.00226295	1.17E-25	CA, Serum-25(OH)D
HDL-C	rs11254464	10	C	T	0.857972	0.0130657	0.0019782	3.98E-11	CA, Serum-25(OH)D
HDL-C	rs113563886	16	C	T	0.694291	0.059432	0.00313284	2.98E-80	CA, Serum-25(OH)D
HDL-C	rs115447786	6	T	C	0.063489	-0.0510865	0.00470143	1.67E-27	CA, Serum-25(OH)D
HDL-C	rs11614202	12	G	A	0.555572	0.0217765	0.00268794	5.43E-16	CA, Serum-25(OH)D
HDL-C	rs11653260	17	G	A	0.421212	-0.0142414	0.00237893	2.14E-09	CA, Serum-25(OH)D
HDL-C	rs116577546	9	T	G	0.327715	-0.0196661	0.00319974	7.94E-10	CA, Serum-25(OH)D
HDL-C	rs11664369	18	T	C	0.816012	-0.0226943	0.00220953	9.51E-25	CA, Serum-25(OH)D
HDL-C	rs1168114	1	G	A	0.236207	0.0139866	0.00205213	9.39E-12	CA, Serum-25(OH)D
HDL-C	rs116843064	19	A	G	0.336888	0.209801	0.00710304	9.73E-192	CA, Serum-25(OH)D
HDL-C	rs117230571	13	G	A	0.161673	-0.026237	0.00370367	1.40E-12	CA, Serum-25(OH)D
HDL-C	rs117266250	15	A	G	0.021522	0.0635108	0.00476675	1.69E-40	CA, Serum-25(OH)D
HDL-C	rs117269855	12	T	C	0.030856	-0.0302995	0.00520185	5.72E-09	CA, Serum-25(OH)D
HDL-C	rs11738093	5	G	A	0.518782	-0.0144112	0.00225375	1.61E-10	CA, Serum-25(OH)D
HDL-C	rs117488242	10	G	A	0.251688	-0.0165034	0.00300812	4.11E-08	CA, Serum-25(OH)D
HDL-C	rs117597286	15	C	T	0.069764	-0.0841351	0.0109956	1.98E-14	CA, Serum-25(OH)D
HDL-C	rs11789603	9	T	C	0.191458	0.0661776	0.00315248	7.72E-98	CA, Serum-25(OH)D
HDL-C	rs11894589	2	T	C	0.057345	0.0117309	0.00214743	4.69E-08	CA, Serum-25(OH)D
HDL-C	rs11973318	7	C	T	0.127965	-0.0177448	0.00279111	2.05E-10	CA, Serum-25(OH)D

HDL-C	rs12146566	11	C	A	0.018215	-0.0145224	0.00246687	3.93E-09	CA, Serum-25(OH)D
HDL-C	rs1225056	3	A	C	0.203652	-0.012639	0.00221739	1.20E-08	CA, Serum-25(OH)D
HDL-C	rs1240811	6	T	C	0.216136	0.0133753	0.00212589	3.14E-10	CA, Serum-25(OH)D
HDL-C	rs12462109	19	T	C	0.324978	-0.0144195	0.00216482	2.72E-11	CA, Serum-25(OH)D
HDL-C	rs12629376	3	C	A	0.369937	0.0119688	0.00197033	1.24E-09	CA, Serum-25(OH)D
HDL-C	rs12632030	3	C	T	0.59916	-0.0116081	0.00195593	2.94E-09	CA, Serum-25(OH)D
HDL-C	rs12686780	9	T	C	0.023223	-0.0172583	0.00257902	2.20E-11	CA, Serum-25(OH)D
HDL-C	rs12702510	7	T	C	0.190862	-0.0257489	0.00214661	3.77E-33	CA, Serum-25(OH)D
HDL-C	rs12887521	14	A	C	0.013145	0.016567	0.00204982	6.36E-16	CA, Serum-25(OH)D
HDL-C	rs12895424	14	T	G	0.045225	0.0115108	0.00196589	4.76E-09	CA, Serum-25(OH)D
HDL-C	rs12921195	16	A	C	0.074502	-0.0182459	0.00298766	1.01E-09	CA, Serum-25(OH)D
HDL-C	rs12928099	16	A	C	0.06632	0.0217868	0.00219341	3.00E-23	CA, Serum-25(OH)D
HDL-C	rs12986742	2	C	T	0.017455	-0.0121357	0.00196578	6.68E-10	CA, Serum-25(OH)D
HDL-C	rs13066793	3	G	A	0.069337	0.0189099	0.00340817	2.88E-08	CA, Serum-25(OH)D
HDL-C	rs13107325	4	T	C	0.452899	-0.0806844	0.00372003	2.59E-104	CA, Serum-25(OH)D
HDL-C	rs13111599	4	G	A	0.522685	0.0140001	0.00221912	2.81E-10	CA, Serum-25(OH)D
HDL-C	rs13134992	4	T	C	0.041703	0.0130897	0.00198914	4.69E-11	CA, Serum-25(OH)D
HDL-C	rs13179413	5	T	C	0.638424	-0.0227449	0.00219503	3.69E-25	CA, Serum-25(OH)D
HDL-C	rs13360957	5	A	G	0.315761	0.0147052	0.00255044	8.13E-09	CA, Serum-25(OH)D
HDL-C	rs13379043	14	C	T	0.018489	0.0195001	0.00221298	1.23E-18	CA, Serum-25(OH)D
HDL-C	rs13389219	2	T	C	0.107996	0.0277767	0.00199786	6.05E-44	CA, Serum-25(OH)D
HDL-C	rs137878880	17	TA	T	0.26884	0.0125332	0.00209699	2.28E-09	CA, Serum-25(OH)D
HDL-C	rs137991784	19	A	G	0.477596	0.017233	0.00297034	6.56E-09	CA, Serum-25(OH)D
HDL-C	rs140489	22	A	G	0.16648	-0.0322505	0.00246876	5.33E-39	CA, Serum-25(OH)D
HDL-C	rs141062196	17	A	G	0.517654	-0.0174648	0.00247141	1.59E-12	CA, Serum-25(OH)D
HDL-C	rs1412234	9	C	T	0.20785	-0.0127381	0.00208637	1.03E-09	CA, Serum-25(OH)D

HDL-C	rs141440048	1	T	C	0.259243	0.0459719	0.00768655	2.22E-09	CA, Serum-25(OH)D
HDL-C	rs141469619	11	G	A	0.252046	-0.191251	0.0103112	8.47E-77	CA, Serum-25(OH)D
HDL-C	rs141859350	16	A	G	0.313736	-0.0731804	0.00529169	1.70E-43	CA, Serum-25(OH)D
HDL-C	rs142288236	8	T	C	0.07415	-0.0818735	0.00830726	6.48E-23	CA, Serum-25(OH)D
HDL-C	rs1423551	5	T	C	0.139018	-0.0123505	0.00210354	4.32E-09	CA, Serum-25(OH)D
HDL-C	rs1431659	8	G	A	0.97343	0.0131758	0.00222149	3.01E-09	CA, Serum-25(OH)D
HDL-C	rs143771461	15	T	C	0.084231	-0.0659264	0.00951274	4.20E-12	CA, Serum-25(OH)D
HDL-C	rs144014029	8	C	T	0.074873	0.0910321	0.00942758	4.64E-22	CA, Serum-25(OH)D
HDL-C	rs144311893	19	T	C	0.146378	0.0813137	0.00680947	7.21E-33	CA, Serum-25(OH)D
HDL-C	rs1446585	2	G	A	0.598483	0.019988	0.00234189	1.40E-17	CA, Serum-25(OH)D
HDL-C	rs145660863	16	AG	A	0.704276	0.0776597	0.00458341	2.14E-64	CA, Serum-25(OH)D
HDL-C	rs145919630	5	ACT	A	0.736205	-0.0304258	0.00524702	6.68E-09	CA, Serum-25(OH)D
HDL-C	rs1464456	4	A	G	0.60459	0.0125164	0.00203538	7.78E-10	CA, Serum-25(OH)D
HDL-C	rs147011441	8	A	G	0.075512	-0.0437767	0.00648135	1.44E-11	CA, Serum-25(OH)D
HDL-C	rs148024643	6	A	G	0.534555	-0.0266496	0.00221692	2.76E-33	CA, Serum-25(OH)D
HDL-C	rs1497077	14	C	T	0.141916	0.0113371	0.0020554	3.47E-08	CA, Serum-25(OH)D
HDL-C	rs150224153	20	T	C	0.330225	-0.0930854	0.00581923	1.36E-57	CA, Serum-25(OH)D
HDL-C	rs150237291	18	C	T	0.063027	0.0505568	0.00664819	2.86E-14	CA, Serum-25(OH)D
HDL-C	rs150844304	15	C	A	0.221249	-0.0915023	0.00632576	2.02E-47	CA, Serum-25(OH)D
HDL-C	rs1534696	7	A	C	0.368809	0.0175731	0.00195614	2.62E-19	CA, Serum-25(OH)D
HDL-C	rs1544913	2	C	T	0.400373	-0.0208462	0.00213602	1.68E-22	CA, Serum-25(OH)D
HDL-C	rs1618725	18	T	C	0.408464	0.0159266	0.00195531	3.78E-16	CA, Serum-25(OH)D
HDL-C	rs1645788	19	G	A	0.231381	0.0411707	0.00233386	1.20E-69	CA, Serum-25(OH)D
HDL-C	rs16928809	11	A	G	0.593991	-0.0285157	0.0033906	4.09E-17	CA, Serum-25(OH)D
HDL-C	rs17041868	2	C	T	0.259454	-0.0259151	0.0039819	7.61E-11	CA, Serum-25(OH)D
HDL-C	rs17309930	11	A	C	0.074856	-0.0229014	0.0024157	2.54E-21	CA, Serum-25(OH)D

HDL-C	rs17326656	2	T	G	0.615429	-0.0210604	0.00229941	5.23E-20	CA, Serum-25(OH)D
HDL-C	rs174566	11	G	A	0.138453	-0.0582235	0.00205306	6.41E-177	CA, Serum-25(OH)D
HDL-C	rs17663165	3	A	C	0.114359	0.0153533	0.00198009	8.92E-15	CA, Serum-25(OH)D
HDL-C	rs1771582	1	G	T	0.358514	0.0142951	0.00203455	2.12E-12	CA, Serum-25(OH)D
HDL-C	rs1779809	1	T	C	0.475358	-0.0247036	0.00207689	1.26E-32	CA, Serum-25(OH)D
HDL-C	rs183906992	7	C	T	0.627819	0.0309074	0.00484831	1.83E-10	CA, Serum-25(OH)D
HDL-C	rs1968493	16	G	A	0.278463	0.0717175	0.00217584	1.00E-200	CA, Serum-25(OH)D
HDL-C	rs1970811	10	C	T	0.135064	-0.0113083	0.00195971	7.91E-09	CA, Serum-25(OH)D
HDL-C	rs1980552	20	T	C	0.658569	0.0130891	0.00197768	3.63E-11	CA, Serum-25(OH)D
HDL-C	rs198325	1	T	C	0.009952	0.0194958	0.00235745	1.34E-16	CA, Serum-25(OH)D
HDL-C	rs200540247	15	C	A	0.072227	0.0138815	0.00218695	2.19E-10	CA, Serum-25(OH)D
HDL-C	rs2011614	17	A	G	0.72482	-0.0146463	0.00203985	6.97E-13	CA, Serum-25(OH)D
HDL-C	rs2058914	15	A	G	0.033035	0.0146822	0.00215693	9.97E-12	CA, Serum-25(OH)D
HDL-C	rs2066714	9	C	T	0.011692	0.0458185	0.00293471	5.97E-55	CA, Serum-25(OH)D
HDL-C	rs2067819	3	A	G	0.186396	0.0194017	0.00237702	3.29E-16	CA, Serum-25(OH)D
HDL-C	rs2068888	10	A	G	0.023031	0.0180208	0.00195775	3.42E-20	CA, Serum-25(OH)D
HDL-C	rs2098918	4	T	C	0.07348	0.0120292	0.00195951	8.31E-10	CA, Serum-25(OH)D
HDL-C	rs2111216	12	G	A	0.110337	0.0215379	0.0019934	3.27E-27	CA, Serum-25(OH)D
HDL-C	rs2159607	3	T	G	0.879895	-0.0244579	0.0024892	8.73E-23	CA, Serum-25(OH)D
HDL-C	rs2175766	4	C	A	0.579087	0.0114332	0.0019585	5.29E-09	CA, Serum-25(OH)D
HDL-C	rs2236464	21	C	T	0.216685	-0.0171198	0.00239771	9.33E-13	CA, Serum-25(OH)D
HDL-C	rs2247355	8	T	C	0.101364	0.0190194	0.00254923	8.60E-14	CA, Serum-25(OH)D
HDL-C	rs2266130	11	A	G	0.020706	-0.0120562	0.00221142	4.99E-08	CA, Serum-25(OH)D
HDL-C	rs2290866	8	T	C	0.00977	-0.0132754	0.00225919	4.20E-09	CA, Serum-25(OH)D
HDL-C	rs2294915	22	T	C	0.286145	-0.0130119	0.00230786	1.72E-08	CA, Serum-25(OH)D
HDL-C	rs2297402	9	T	C	0.613593	-0.0702982	0.00664935	4.01E-26	CA, Serum-25(OH)D

HDL-C	rs2298624	18	T	C	0.421645	0.0304429	0.00287633	3.54E-26	CA, Serum-25(OH)D
HDL-C	rs2298632	1	T	C	0.613818	0.0126329	0.00199227	2.28E-10	CA, Serum-25(OH)D
HDL-C	rs2302263	11	T	C	0.515715	-0.0377043	0.00346623	1.47E-27	CA, Serum-25(OH)D
HDL-C	rs2306363	11	T	G	0.046227	0.0217351	0.0024242	3.08E-19	CA, Serum-25(OH)D
HDL-C	rs2307111	5	C	T	0.240518	0.0180816	0.0019975	1.40E-19	CA, Serum-25(OH)D
HDL-C	rs235314	21	T	C	0.012152	-0.0176643	0.0019521	1.44E-19	CA, Serum-25(OH)D
HDL-C	rs2417125	9	G	A	0.409538	-0.0126629	0.00217649	5.95E-09	CA, Serum-25(OH)D
HDL-C	rs2419605	10	G	A	0.500916	-0.0297213	0.00276375	5.68E-27	CA, Serum-25(OH)D
HDL-C	rs2494748	14	T	C	0.348974	-0.0252129	0.00200696	3.38E-36	CA, Serum-25(OH)D
HDL-C	rs251465	5	C	A	0.108482	0.0130889	0.00227121	8.27E-09	CA, Serum-25(OH)D
HDL-C	rs254024	5	T	G	0.047695	-0.0113675	0.00196319	7.02E-09	CA, Serum-25(OH)D
HDL-C	rs254559	5	A	C	0.035215	-0.0123831	0.0019851	4.43E-10	CA, Serum-25(OH)D
HDL-C	rs2642438	1	G	A	0.024109	0.028106	0.00213885	1.92E-39	CA, Serum-25(OH)D
HDL-C	rs2643195	17	G	A	0.044252	0.0277715	0.00210876	1.31E-39	CA, Serum-25(OH)D
HDL-C	rs2653363	6	T	C	0.077103	0.0107292	0.00195702	4.20E-08	CA, Serum-25(OH)D
HDL-C	rs2715554	17	G	A	0.03097	-0.0169269	0.00272067	4.92E-10	CA, Serum-25(OH)D
HDL-C	rs2723065	2	G	A	0.030367	0.015493	0.00201876	1.66E-14	CA, Serum-25(OH)D
HDL-C	rs2726114	7	G	A	0.154191	-0.015353	0.00243789	3.02E-10	CA, Serum-25(OH)D
HDL-C	rs2740488	9	C	A	0.031445	-0.0683484	0.00222098	1.00E-200	CA, Serum-25(OH)D
HDL-C	rs2745411	6	A	G	0.485636	-0.0117855	0.00194966	1.49E-09	CA, Serum-25(OH)D
HDL-C	rs2792751	10	C	T	0.564604	-0.0354451	0.00218543	3.71E-59	CA, Serum-25(OH)D
HDL-C	rs2804894	10	A	G	0.656059	0.0167041	0.00223061	6.96E-14	CA, Serum-25(OH)D
HDL-C	rs282146	20	A	G	0.12843	-0.0138926	0.00201112	4.92E-12	CA, Serum-25(OH)D
HDL-C	rs2823021	21	A	G	0.450637	-0.0136109	0.00240405	1.50E-08	CA, Serum-25(OH)D
HDL-C	rs28446899	8	T	C	0.183957	-0.0251871	0.00368914	8.65E-12	CA, Serum-25(OH)D
HDL-C	rs28718554	18	C	T	0.345708	-0.0137422	0.00229908	2.27E-09	CA, Serum-25(OH)D

HDL-C	rs2925979	16	C	T	0.705772	0.0373204	0.00217927	9.63E-66	CA, Serum-25(OH)D
HDL-C	rs2943645	2	T	C	0.239282	-0.04504	0.00204269	9.68E-108	CA, Serum-25(OH)D
HDL-C	rs2963468	5	G	A	0.473689	-0.0195123	0.0023191	3.97E-17	CA, Serum-25(OH)D
HDL-C	rs296886	9	G	A	0.720191	0.0140745	0.00238354	3.53E-09	CA, Serum-25(OH)D
HDL-C	rs2980888	8	C	T	0.490986	0.0390633	0.00214586	4.79E-74	CA, Serum-25(OH)D
HDL-C	rs308	8	G	T	0.346096	0.130848	0.00688141	1.29E-80	CA, Serum-25(OH)D
HDL-C	rs3184504	12	C	T	0.523181	0.028304	0.00195763	2.22E-47	CA, Serum-25(OH)D
HDL-C	rs34237080	1	T	C	0.211657	-0.021515	0.00368761	5.40E-09	CA, Serum-25(OH)D
HDL-C	rs343	8	A	C	0.724548	0.136302	0.00360345	1.00E-200	CA, Serum-25(OH)D
HDL-C	rs34672342	5	TA	T	0.279149	-0.011936	0.00211493	1.66E-08	CA, Serum-25(OH)D
HDL-C	rs34712273	1	A	C	0.416806	-0.0131813	0.00198998	3.50E-11	CA, Serum-25(OH)D
HDL-C	rs34940374	7	A	G	0.363764	-0.0147987	0.00253028	4.96E-09	CA, Serum-25(OH)D
HDL-C	rs351379	1	T	G	0.877312	0.0121155	0.00202122	2.05E-09	CA, Serum-25(OH)D
HDL-C	rs35473170	4	A	G	0.633864	0.015591	0.00207187	5.27E-14	CA, Serum-25(OH)D
HDL-C	rs3732356	3	T	G	0.371275	-0.0313032	0.00397188	3.24E-15	CA, Serum-25(OH)D
HDL-C	rs3747973	1	G	A	0.182681	0.0134167	0.00199173	1.63E-11	CA, Serum-25(OH)D
HDL-C	rs375054	16	C	T	0.490158	-0.0540937	0.00374412	2.59E-47	CA, Serum-25(OH)D
HDL-C	rs3768321	1	T	G	0.187574	-0.0461651	0.00245905	1.24E-78	CA, Serum-25(OH)D
HDL-C	rs3769159	2	A	C	0.190609	0.0145545	0.00242277	1.89E-09	CA, Serum-25(OH)D
HDL-C	rs3803800	17	G	A	0.65876	0.022489	0.00238731	4.50E-21	CA, Serum-25(OH)D
HDL-C	rs3814883	16	T	C	0.424494	-0.0167744	0.00200619	6.20E-17	CA, Serum-25(OH)D
HDL-C	rs3912391	4	A	G	0.160889	0.0129785	0.00195086	2.88E-11	CA, Serum-25(OH)D
HDL-C	rs4016338	12	C	T	0.279516	0.0218557	0.00350765	4.64E-10	CA, Serum-25(OH)D
HDL-C	rs40270	5	C	A	0.592593	-0.0292065	0.00232336	3.05E-36	CA, Serum-25(OH)D
HDL-C	rs429358	19	C	T	0.265267	-0.0779609	0.00269421	4.15E-184	CA, Serum-25(OH)D
HDL-C	rs4309533	2	A	G	0.533523	-0.0156071	0.00269918	7.37E-09	CA, Serum-25(OH)D

HDL-C	rs4323719	1	T	C	0.427878	-0.0172504	0.00197021	2.03E-18	CA, Serum-25(OH)D
HDL-C	rs4327684	6	G	A	0.07839	0.012884	0.00210813	9.87E-10	CA, Serum-25(OH)D
HDL-C	rs4330777	16	A	G	0.755639	-0.0230878	0.00200308	9.74E-31	CA, Serum-25(OH)D
HDL-C	rs4332553	12	C	T	0.418796	-0.0137529	0.00199727	5.74E-12	CA, Serum-25(OH)D
HDL-C	rs4369653	16	T	C	0.196344	0.076252	0.0021823	1.00E-200	CA, Serum-25(OH)D
HDL-C	rs4614	11	G	A	0.048982	-0.0172976	0.00199603	4.48E-18	CA, Serum-25(OH)D
HDL-C	rs4650994	1	A	G	0.679945	-0.0203661	0.00195461	2.02E-25	CA, Serum-25(OH)D
HDL-C	rs467303	9	T	C	0.647469	0.0140837	0.00221665	2.10E-10	CA, Serum-25(OH)D
HDL-C	rs4675812	2	A	G	0.5092	0.0156207	0.00198268	3.31E-15	CA, Serum-25(OH)D
HDL-C	rs4686392	3	G	A	0.606043	-0.0127076	0.00215179	3.51E-09	CA, Serum-25(OH)D
HDL-C	rs468692	22	T	C	0.155458	0.0133262	0.00231286	8.32E-09	CA, Serum-25(OH)D
HDL-C	rs4803748	19	T	C	0.078193	0.012221	0.00202355	1.55E-09	CA, Serum-25(OH)D
HDL-C	rs4804413	19	T	C	0.187636	-0.0144587	0.00197524	2.48E-13	CA, Serum-25(OH)D
HDL-C	rs4805881	19	C	A	0.466702	0.022508	0.00207509	2.07E-27	CA, Serum-25(OH)D
HDL-C	rs4834749	4	A	G	0.131437	0.0149329	0.0025352	3.86E-09	CA, Serum-25(OH)D
HDL-C	rs4843780	16	T	C	0.147847	-0.0156616	0.00203341	1.34E-14	CA, Serum-25(OH)D
HDL-C	rs4875043	8	C	A	0.44237	-0.013509	0.00240478	1.94E-08	CA, Serum-25(OH)D
HDL-C	rs488490	15	A	C	0.491703	-0.106871	0.00280366	1.00E-200	CA, Serum-25(OH)D
HDL-C	rs4899251	14	T	C	0.376823	-0.0256251	0.00423503	1.44E-09	CA, Serum-25(OH)D
HDL-C	rs490937	11	G	T	0.040443	-0.0117072	0.00212814	3.77E-08	CA, Serum-25(OH)D
HDL-C	rs4917675	10	C	T	0.491442	0.0151956	0.00223983	1.17E-11	CA, Serum-25(OH)D
HDL-C	rs4930352	11	T	G	0.123843	0.015897	0.00199616	1.67E-15	CA, Serum-25(OH)D
HDL-C	rs4939883	18	C	T	0.780531	0.0854533	0.00254446	1.00E-200	CA, Serum-25(OH)D
HDL-C	rs4947121	6	C	T	0.399891	-0.0133155	0.00234616	1.38E-08	CA, Serum-25(OH)D
HDL-C	rs4950978	1	T	C	0.067554	-0.0137339	0.00236077	5.97E-09	CA, Serum-25(OH)D
HDL-C	rs4969143	17	C	T	0.915	0.0303833	0.00203186	1.48E-50	CA, Serum-25(OH)D

HDL-C	rs55665473	10	A	G	0.081	0.030516	0.00228323	9.65E-41	CA, Serum-25(OH)D
HDL-C	rs558003	13	A	G	0.593	0.0197377	0.00333843	3.37E-09	CA, Serum-25(OH)D
HDL-C	rs558971	1	G	A	0.612	0.0169648	0.0019632	5.55E-18	CA, Serum-25(OH)D
HDL-C	rs56101431	13	C	T	0.225	-0.0111519	0.00199082	2.12E-08	CA, Serum-25(OH)D
HDL-C	rs56848735	12	T	C	0.394	-0.0210896	0.00259448	4.34E-16	CA, Serum-25(OH)D
HDL-C	rs57384528	3	A	C	0.023	0.0129923	0.00234421	2.99E-08	CA, Serum-25(OH)D
HDL-C	rs5788459	10	TA	T	0.835	-0.0192461	0.00345526	2.55E-08	CA, Serum-25(OH)D
HDL-C	rs58729290	17	A	G	0.184	-0.0298949	0.00403461	1.27E-13	CA, Serum-25(OH)D
HDL-C	rs59360013	18	T	C	0.068	-0.0268348	0.00485534	3.26E-08	CA, Serum-25(OH)D
HDL-C	rs59781045	11	T	C	0.353	0.0738466	0.00391307	1.95E-79	CA, Serum-25(OH)D
HDL-C	rs6018652	20	A	G	0.021	0.024449	0.00239955	2.22E-24	CA, Serum-25(OH)D
HDL-C	rs60572790	11	C	T	0.261	-0.0117912	0.00211596	2.51E-08	CA, Serum-25(OH)D
HDL-C	rs6066149	20	A	G	0.393	0.0145458	0.00216849	1.98E-11	CA, Serum-25(OH)D
HDL-C	rs6073958	20	C	T	0.667	-0.0611812	0.00244466	3.15E-138	CA, Serum-25(OH)D
HDL-C	rs6123685	20	A	G	0.478	0.0151632	0.00224153	1.34E-11	CA, Serum-25(OH)D
HDL-C	rs6142206	20	A	G	0.238	-0.0166433	0.00197438	3.47E-17	CA, Serum-25(OH)D
HDL-C	rs61749613	5	G	A	0.121	0.0310417	0.00489675	2.31E-10	CA, Serum-25(OH)D
HDL-C	rs62117489	19	A	C	0.358	0.0467021	0.00430598	2.09E-27	CA, Serum-25(OH)D
HDL-C	rs62217799	20	T	G	0.49	-0.011648	0.00206867	1.79E-08	CA, Serum-25(OH)D
HDL-C	rs62285077	3	T	C	0.205	0.0154736	0.00264927	5.20E-09	CA, Serum-25(OH)D
HDL-C	rs62526883	8	G	T	0.061	-0.0131225	0.00227124	7.57E-09	CA, Serum-25(OH)D
HDL-C	rs635769	6	C	T	0.255	0.0210318	0.00201789	1.95E-25	CA, Serum-25(OH)D
HDL-C	rs646776	1	T	C	0.097	-0.0286243	0.00235229	4.56E-34	CA, Serum-25(OH)D
HDL-C	rs6542680	2	T	C	0.646	-0.0264511	0.00256426	6.01E-25	CA, Serum-25(OH)D
HDL-C	rs66733826	2	C	T	0.692	0.0131501	0.00231915	1.43E-08	CA, Serum-25(OH)D
HDL-C	rs6705285	2	T	G	0.811	0.0108553	0.00197254	3.73E-08	CA, Serum-25(OH)D

HDL-C	rs6718037	2	G	T	0.431	-0.0116906	0.00197352	3.15E-09	CA, Serum-25(OH)D
HDL-C	rs676210	2	A	G	0.13	0.0599317	0.00242205	3.58E-135	CA, Serum-25(OH)D
HDL-C	rs6762415	3	G	T	0.462	-0.0107927	0.00196007	3.66E-08	CA, Serum-25(OH)D
HDL-C	rs6765484	3	T	C	0.203	0.0233916	0.00195569	5.70E-33	CA, Serum-25(OH)D
HDL-C	rs68062403	15	G	A	0.226	-0.0130705	0.00212106	7.17E-10	CA, Serum-25(OH)D
HDL-C	rs686030	9	A	C	0.193	0.049521	0.002807	1.17E-69	CA, Serum-25(OH)D
HDL-C	rs6877776	5	G	A	0.393	-0.0147944	0.00201942	2.37E-13	CA, Serum-25(OH)D
HDL-C	rs689183	11	T	G	0.246	-0.0147057	0.0022832	1.19E-10	CA, Serum-25(OH)D
HDL-C	rs6934962	6	T	C	0.664	0.0165364	0.00199096	9.92E-17	CA, Serum-25(OH)D
HDL-C	rs7010207	8	T	C	0.4	0.0147135	0.00223864	4.95E-11	CA, Serum-25(OH)D
HDL-C	rs703966	10	A	G	0.697	0.0152056	0.00197604	1.42E-14	CA, Serum-25(OH)D
HDL-C	rs7136506	12	C	T	0.271	-0.0384138	0.00241316	4.72E-57	CA, Serum-25(OH)D
HDL-C	rs7144654	14	G	A	0.394	-0.0147362	0.00195223	4.41E-14	CA, Serum-25(OH)D
HDL-C	rs7149672	14	G	A	0.41	-0.0123293	0.00205517	1.98E-09	CA, Serum-25(OH)D
HDL-C	rs7156516	14	T	G	0.037	-0.0163567	0.00227153	5.99E-13	CA, Serum-25(OH)D
HDL-C	rs71603401	4	G	A	0.603	-0.0157241	0.00287476	4.51E-08	CA, Serum-25(OH)D
HDL-C	rs7170463	15	G	A	0.634	0.0207597	0.0021214	1.29E-22	CA, Serum-25(OH)D
HDL-C	rs7199285	16	T	C	0.174	0.014885	0.00261966	1.33E-08	CA, Serum-25(OH)D
HDL-C	rs7251640	19	C	T	0.146	0.0147618	0.00249071	3.09E-09	CA, Serum-25(OH)D
HDL-C	rs72630915	1	T	C	0.234	-0.0143819	0.00255928	1.92E-08	CA, Serum-25(OH)D
HDL-C	rs72647336	8	A	G	0.113	-0.0457267	0.00455999	1.15E-23	CA, Serum-25(OH)D
HDL-C	rs72801474	5	A	G	0.212	0.0215319	0.00335273	1.34E-10	CA, Serum-25(OH)D
HDL-C	rs72836561	17	T	C	0.591	-0.168228	0.00552841	1.00E-200	CA, Serum-25(OH)D
HDL-C	rs72959041	6	A	G	0.024	-0.052141	0.00452333	9.63E-31	CA, Serum-25(OH)D
HDL-C	rs72964564	3	C	A	0.712	-0.0123546	0.00225131	4.07E-08	CA, Serum-25(OH)D
HDL-C	rs7307711	12	C	T	0.074	-0.0123456	0.00220968	2.31E-08	CA, Serum-25(OH)D

HDL-C	rs73109462	7	G	A	0.176	-0.0176584	0.00290016	1.14E-09	CA, Serum-25(OH)D
HDL-C	rs73243877	4	G	A	0.203	-0.0268967	0.00261193	7.22E-25	CA, Serum-25(OH)D
HDL-C	rs737337	19	C	T	0.783	-0.0545912	0.00367826	7.89E-50	CA, Serum-25(OH)D
HDL-C	rs74456742	17	A	G	0.399	0.0324141	0.0053363	1.25E-09	CA, Serum-25(OH)D
HDL-C	rs74500135	9	C	T	0.614	0.0605072	0.0103936	5.83E-09	CA, Serum-25(OH)D
HDL-C	rs7546242	1	T	C	0.123	0.0131506	0.00202668	8.66E-11	CA, Serum-25(OH)D
HDL-C	rs75479205	6	G	A	0.417	0.0137385	0.00248201	3.11E-08	CA, Serum-25(OH)D
HDL-C	rs75609851	8	A	G	0.316	0.173443	0.00992822	2.44E-68	CA, Serum-25(OH)D
HDL-C	rs75663614	15	C	T	0.151	-0.103704	0.00565354	3.74E-75	CA, Serum-25(OH)D
HDL-C	rs7583067	2	T	C	0.078	0.0157565	0.00230151	7.59E-12	CA, Serum-25(OH)D
HDL-C	rs76186975	14	T	C	0.199	-0.0168461	0.00267194	2.89E-10	CA, Serum-25(OH)D
HDL-C	rs76281393	4	G	T	0.663	-0.0162671	0.00269072	1.49E-09	CA, Serum-25(OH)D
HDL-C	rs76428106	13	C	T	0.256	-0.0514878	0.00896423	9.26E-09	CA, Serum-25(OH)D
HDL-C	rs76534758	7	C	T	0.021	0.0158204	0.00232579	1.03E-11	CA, Serum-25(OH)D
HDL-C	rs76602912	20	C	T	0.083	-0.0372021	0.00640565	6.33E-09	CA, Serum-25(OH)D
HDL-C	rs7665587	4	C	T	0.279	0.0137423	0.00198045	3.95E-12	CA, Serum-25(OH)D
HDL-C	rs7684939	4	A	G	0.471	0.0141757	0.00194872	3.48E-13	CA, Serum-25(OH)D
HDL-C	rs76856822	18	C	T	0.03	0.0450406	0.0075136	2.04E-09	CA, Serum-25(OH)D
HDL-C	rs76880877	3	C	A	0.017	-0.0288303	0.00285041	4.77E-24	CA, Serum-25(OH)D
HDL-C	rs7700617	5	A	C	0.173	-0.0118587	0.00195466	1.30E-09	CA, Serum-25(OH)D
HDL-C	rs77243579	6	G	A	0.308	-0.0201574	0.0020964	6.89E-22	CA, Serum-25(OH)D
HDL-C	rs7725218	5	A	G	0.164	-0.012542	0.00206201	1.18E-09	CA, Serum-25(OH)D
HDL-C	rs7730268	5	G	T	0.121	-0.0202961	0.00366287	3.01E-08	CA, Serum-25(OH)D
HDL-C	rs77320712	3	T	G	0.253	-0.012655	0.00231764	4.75E-08	CA, Serum-25(OH)D
HDL-C	rs7794796	7	T	C	0.589	-0.0172666	0.00208884	1.38E-16	CA, Serum-25(OH)D
HDL-C	rs77960347	18	G	A	0.697	0.285486	0.00846122	1.00E-200	CA, Serum-25(OH)D

HDL-C	rs78058190	2	A	G	0.551	-0.077146	0.00501273	1.91E-53	CA, Serum-25(OH)D
HDL-C	rs7817574	8	C	T	0.723	0.0346457	0.0025252	7.71E-43	CA, Serum-25(OH)D
HDL-C	rs7826177	8	C	T	0.418	0.0120367	0.0020484	4.20E-09	CA, Serum-25(OH)D
HDL-C	rs78506520	11	A	G	0.183	0.0231523	0.00407544	1.34E-08	CA, Serum-25(OH)D
HDL-C	rs79153732	8	T	C	0.161	-0.0928086	0.00765921	8.56E-34	CA, Serum-25(OH)D
HDL-C	rs7924036	10	T	G	0.261	0.013836	0.00195085	1.32E-12	CA, Serum-25(OH)D
HDL-C	rs79354956	18	G	A	0.35	0.0339182	0.00600684	1.64E-08	CA, Serum-25(OH)D
HDL-C	rs7964492	12	C	A	0.189	0.0312532	0.00227271	4.99E-43	CA, Serum-25(OH)D
HDL-C	rs8025665	15	A	G	0.079	-0.0114236	0.00207709	3.80E-08	CA, Serum-25(OH)D
HDL-C	rs8083730	18	A	G	0.658	0.0198134	0.00353251	2.04E-08	CA, Serum-25(OH)D
HDL-C	rs8107967	19	G	A	0.074	0.0152238	0.00197286	1.19E-14	CA, Serum-25(OH)D
HDL-C	rs900802	15	T	C	0.085	0.0156331	0.00214177	2.90E-13	CA, Serum-25(OH)D
HDL-C	rs907866	2	A	G	0.054	-0.0183126	0.00196957	1.43E-20	CA, Serum-25(OH)D
HDL-C	rs921919	12	A	G	0.046	-0.0416808	0.00212036	5.00E-86	CA, Serum-25(OH)D
HDL-C	rs9306	6	C	T	0.395	-0.0139282	0.00201906	5.26E-12	CA, Serum-25(OH)D
HDL-C	rs932589	6	T	C	0.232	-0.0141653	0.00200077	1.44E-12	CA, Serum-25(OH)D
HDL-C	rs9490	7	C	T	0.239	0.0130301	0.00229313	1.33E-08	CA, Serum-25(OH)D
HDL-C	rs9527706	13	G	A	0.313	-0.0132097	0.00218672	1.53E-09	CA, Serum-25(OH)D
HDL-C	rs9604045	13	T	G	0.13	0.0184994	0.00234233	2.84E-15	CA, Serum-25(OH)D
HDL-C	rs9608972	22	C	T	0.184	-0.0154562	0.00227071	9.98E-12	CA, Serum-25(OH)D
HDL-C	rs968050	6	T	C	0.345	0.0142919	0.00195489	2.65E-13	CA, Serum-25(OH)D
HDL-C	rs9877304	3	A	G	0.315	-0.0141294	0.00222277	2.06E-10	CA, Serum-25(OH)D
HDL-C	rs9977554	21	C	T	0.31	-0.0131766	0.00237454	2.87E-08	CA, Serum-25(OH)D
HDL-C	rs998584	6	A	C	0.196	-0.0337103	0.0019577	1.90E-66	CA, Serum-25(OH)D
HDL-C	rs9987289	8	G	A	0.267	0.0913426	0.00339798	3.62E-159	CA, Serum-25(OH)D
TG	rs10401969	15	G	A	0.276	0.013	0.002	2.00E-08	CA, Serum-25(OH)D

TG	rs10440120	15	T	A	0.255	0.016	0.002	2.20E-12	CA, Serum-25(OH)D
TG	rs10501321	15	C	A	0.425	-0.022	0.002	1.10E-27	CA, Serum-25(OH)D
TG	rs10761762	15	C	T	0.612	-0.031	0.002	1.90E-51	CA, Serum-25(OH)D
TG	rs10790162	15	G	T	0.162	-0.017	0.003	2.90E-10	CA, Serum-25(OH)D
TG	rs11057408	16	G	A	0.455	0.013	0.002	2.70E-10	CA, Serum-25(OH)D
TG	rs11613352	16	A	C	0.296	-0.028	0.002	2.90E-38	CA, Serum-25(OH)D
TG	rs11820504	16	G	T	0.069	-0.022	0.004	1.50E-08	CA, Serum-25(OH)D
TG	rs11974409	17	G	C	0.709	-0.014	0.002	1.90E-10	CA, Serum-25(OH)D
TG	rs1260326	17	T	C	0.194	0.014	0.003	3.20E-08	CA, Serum-25(OH)D
TG	rs12676857	17	G	T	0.604	-0.018	0.002	2.80E-18	CA, Serum-25(OH)D
TG	rs12678919	17	G	A	0.063	0.039	0.004	2.30E-21	CA, Serum-25(OH)D
TG	rs12748152	17	A	G	0.728	0.012	0.002	3.60E-08	CA, Serum-25(OH)D
TG	rs1321257	17	T	C	0.774	-0.015	0.002	2.10E-10	CA, Serum-25(OH)D
TG	rs13389219	18	T	C	0.073	-0.023	0.004	2.50E-09	CA, Serum-25(OH)D
TG	rs16948098	18	C	T	0.49	-0.016	0.002	6.80E-16	CA, Serum-25(OH)D
TG	rs174535	18	A	G	0.576	-0.014	0.002	1.80E-11	CA, Serum-25(OH)D
TG	rs17513135	19	A	G	0.042	0.028	0.005	2.10E-08	CA, Serum-25(OH)D
TG	rs1832007	19	G	A	0.933	0.028	0.004	1.20E-12	CA, Serum-25(OH)D
TG	rs2043085	19	T	C	0.075	-0.103	0.004	5.30E-164	CA, Serum-25(OH)D
TG	rs2068888	19	T	A	0.286	0.02	0.002	3.90E-20	CA, Serum-25(OH)D
TG	rs2239520	19	T	C	0.183	-0.016	0.003	7.40E-10	CA, Serum-25(OH)D
TG	rs2247056	20	T	C	0.481	0.014	0.002	6.80E-12	CA, Serum-25(OH)D
TG	rs2250802	20	G	C	0.282	-0.018	0.002	7.10E-16	CA, Serum-25(OH)D
TG	rs247616	20	A	G	0.258	-0.013	0.002	2.00E-08	CA, Serum-25(OH)D
TG	rs2665357	20	C	T	0.199	0.056	0.002	1.60E-110	CA, Serum-25(OH)D
TG	rs287621	20	A	G	0.599	0.016	0.002	3.80E-15	CA, Serum-25(OH)D

TG	rs2954022	21	C	T	0.498	-0.013	0.002	9.40E-11	CA, Serum-25(OH)D
TG	rs2972146	22	T	C	0.335	-0.012	0.002	3.40E-08	CA, Serum-25(OH)D
TG	rs3198697	22	A	T	0.345	0.016	0.002	7.30E-15	CA, Serum-25(OH)D
TG	rs3760627	22	T	C	0.581	0.022	0.002	1.40E-26	CA, Serum-25(OH)D
TG	rs3761445	22	C	T	0.214	0.018	0.002	3.20E-13	CA, Serum-25(OH)D
TG	rs38855	7	T	C	0.119	-0.017	0.003	1.70E-08	CA, Serum-25(OH)D
TG	rs439401	8	G	A	0.03	-0.034	0.006	5.40E-09	CA, Serum-25(OH)D
TG	rs442177	15	G	A	0.22	0.047	0.002	2.20E-86	CA, Serum-25(OH)D
TG	rs4587594	12	A	C	0.431	-0.017	0.002	1.20E-17	CA, Serum-25(OH)D
TG	rs4719841	5	G	C	0.222	0.015	0.002	3.90E-10	CA, Serum-25(OH)D
TG	rs4810479	19	C	T	-0.121		0.0065	9.70E-70	CA, Serum-25(OH)D
TG	rs588136	3	A	C	-0.0306		0.0044	5.34E-11	CA, Serum-25(OH)D
TG	rs6029143	11	C	T	-0.0216		0.0035	1.41E-08	CA, Serum-25(OH)D
TG	rs634869	10	C	T	-0.027		0.0033	1.06E-17	CA, Serum-25(OH)D
TG	rs645040	11	G	A	-0.2305		0.0065	1.00E-200	CA, Serum-25(OH)D
TG	rs676210	12	T	G	-0.0258		0.0035	2.05E-12	CA, Serum-25(OH)D
TG	rs6831256	12	T	C	-0.028		0.0039	9.40E-14	CA, Serum-25(OH)D
TG	rs6882076	11	C	T	0.0604		0.0044	1.09E-39	CA, Serum-25(OH)D
TG	rs6995541	7	G	A	-0.0899		0.0042	1.36E-100	CA, Serum-25(OH)D
TG	rs719726	2	C	T	-0.1148		0.0034	1.00E-200	CA, Serum-25(OH)D
TG	rs7248104	8	C	T	0.0332		0.0046	7.29E-12	CA, Serum-25(OH)D
TG	rs731839	8	G	A	-0.1702		0.0056	1.82E-199	CA, Serum-25(OH)D
TG	rs749671	1	T	C	0.0372		0.0059	1.10E-09	CA, Serum-25(OH)D
TG	rs8077889	1	A	G	-0.0402		0.0034	5.99E-31	CA, Serum-25(OH)D
TG	rs9686661	2	T	C	-0.0271		0.0034	2.60E-15	CA, Serum-25(OH)D
TG	rs998584	15	A	G	0.08		0.0089	4.84E-17	CA, Serum-25(OH)D

APO-A1	rs10111219	8	C	T	0.280964	0.0204584	0.00226727	3.19963e-20	CA, Serum-25(OH)D
APO-A1	rs102275	11	C	T	0.351875	-0.043149	0.00213484	1.10002e-97	CA, Serum-25(OH)D
APO-A1	rs10229964	7	G	A	0.427732	-0.0236128	0.00207214	4.60045e-33	CA, Serum-25(OH)D
APO-A1	rs10282707	7	T	C	0.396699	-0.0295705	0.0020951	1.59993e-48	CA, Serum-25(OH)D
APO-A1	rs103294	19	T	C	0.223821	0.0472954	0.00245568	2.19989e-90	CA, Serum-25(OH)D
APO-A1	rs1039968	2	C	T	0.297191	-0.0177334	0.00222829	7.10068e-20	CA, Serum-25(OH)D
APO-A1	rs10454087	17	T	C	0.283041	-0.0132697	0.00228541	1.59993e-11	CA, Serum-25(OH)D
APO-A1	rs1047210	3	C	A	0.476424	-0.0155012	0.00206773	1.99986e-14	CA, Serum-25(OH)D
APO-A1	rs1047891	2	A	C	0.313303	-0.0251166	0.00224589	4.30031e-31	CA, Serum-25(OH)D
APO-A1	rs1060582	4	G	A	0.171192	-0.0146214	0.00274904	1.89998e-08	CA, Serum-25(OH)D
APO-A1	rs1062687	22	C	T	0.294526	-0.0126469	0.00226341	7.90005e-09	CA, Serum-25(OH)D
APO-A1	rs10740118	10	C	G	0.417825	0.0139087	0.0020811	9.49948e-11	CA, Serum-25(OH)D
APO-A1	rs10758189	9	C	T	0.315099	-0.0126389	0.00220239	2.80001e-09	CA, Serum-25(OH)D
APO-A1	rs10773112	12	T	C	0.721304	0.0390846	0.00228287	4.00037e-65	CA, Serum-25(OH)D
APO-A1	rs10840631	12	T	C	0.491847	0.0142094	0.00204378	7.19946e-12	CA, Serum-25(OH)D
APO-A1	rs10876171	12	A	G	0.637904	-0.010313	0.0021253	1.7e-08	CA, Serum-25(OH)D
APO-A1	rs10876447	12	A	G	0.153934	-0.01783	0.00283181	1e-11	CA, Serum-25(OH)D
APO-A1	rs10896018	11	A	G	0.286989	0.0145467	0.00229267	3.10027e-11	CA, Serum-25(OH)D
APO-A1	rs10936575	3	T	C	0.620773	0.0112842	0.00213186	4.20001e-08	CA, Serum-25(OH)D
APO-A1	rs10992836	9	C	T	0.662078	-0.0111967	0.0021826	3.2e-08	CA, Serum-25(OH)D
APO-A1	rs11023265	11	T	C	0.342752	-0.0145561	0.00214718	6.20012e-14	CA, Serum-25(OH)D
APO-A1	rs11065987	12	G	A	0.416423	-0.0203506	0.00207517	2.70023e-25	CA, Serum-25(OH)D
APO-A1	rs11072530	15	A	G	0.635672	0.0174667	0.00211084	4.40048e-17	CA, Serum-25(OH)D
APO-A1	rs11080054	17	A	G	0.592074	-0.0204555	0.00208344	8.80035e-25	CA, Serum-25(OH)D
APO-A1	rs11135037	5	A	G	0.308311	0.0148604	0.00221851	3.40017e-12	CA, Serum-25(OH)D
APO-A1	rs11161243	15	G	A	0.583364	0.0110213	0.00206491	5.19996e-09	CA, Serum-25(OH)D

APO-A1	rs11216060	11	A	G	0.137733	0.0184207	0.00293287	2.69998e-09	CA, Serum-25(OH)D
APO-A1	rs11226108	11	C	G	0.188699	-0.0161667	0.00263559	1.40001e-10	CA, Serum-25(OH)D
APO-A1	rs11248955	16	A	C	0.271989	0.0154791	0.00227141	8.49963e-11	CA, Serum-25(OH)D
APO-A1	rs113017476	2	A	G	0.039359	0.0292431	0.00524599	2.39999e-08	CA, Serum-25(OH)D
APO-A1	rs1132274	20	A	C	0.15344	-0.0342373	0.00285553	1.50003e-33	CA, Serum-25(OH)D
APO-A1	rs11553746	2	T	C	0.346916	0.0121081	0.0021582	1.7e-09	CA, Serum-25(OH)D
APO-A1	rs11626364	14	C	T	0.153695	0.0239527	0.00285247	4.70002e-19	CA, Serum-25(OH)D
APO-A1	rs11630209	15	T	C	0.282665	0.0154932	0.00225594	3.29989e-11	CA, Serum-25(OH)D
APO-A1	rs11632618	15	A	G	0.0698753	0.127925	0.00398805	1e-200	CA, Serum-25(OH)D
APO-A1	rs11639845	16	T	C	0.397538	-0.0115391	0.00206522	7.19996e-10	CA, Serum-25(OH)D
APO-A1	rs11668585	19	T	C	0.104675	-0.022422	0.00334276	5.50047e-12	CA, Serum-25(OH)D
APO-A1	rs1168013	1	G	C	0.64756	0.0517044	0.00213035	4.10204e-135	CA, Serum-25(OH)D
APO-A1	rs11688492	2	C	T	0.455688	0.0114404	0.00205035	3.69999e-08	CA, Serum-25(OH)D
APO-A1	rs11706429	3	A	G	0.453401	0.0118628	0.00205414	1.5e-09	CA, Serum-25(OH)D
APO-A1	rs11717619	3	C	A	0.100839	0.0312407	0.00339899	1.39991e-20	CA, Serum-25(OH)D
APO-A1	rs117291242	11	T	C	0.0366081	-0.0401932	0.00566221	4.79954e-12	CA, Serum-25(OH)D
APO-A1	rs117310695	16	A	G	0.0592036	0.025405	0.00428488	2.69998e-10	CA, Serum-25(OH)D
APO-A1	rs117762989	11	T	C	0.0431587	-0.0270841	0.00501549	1.40001e-08	CA, Serum-25(OH)D
APO-A1	rs117853493	8	A	G	0.0150273	0.0620597	0.0083896	1.9002e-14	CA, Serum-25(OH)D
APO-A1	rs11858548	15	C	T	0.0439375	-0.0375541	0.00500043	2.90001e-14	CA, Serum-25(OH)D
APO-A1	rs1198423	1	T	G	0.86668	0.0207136	0.00303515	1.10002e-11	CA, Serum-25(OH)D
APO-A1	rs11992444	8	T	G	0.509236	0.0104595	0.00204384	3.59998e-08	CA, Serum-25(OH)D
APO-A1	rs12133576	1	G	A	0.63151	-0.0242518	0.00210611	8.60003e-34	CA, Serum-25(OH)D
APO-A1	rs12225230	11	C	G	0.172997	0.107043	0.00270817	1e-200	CA, Serum-25(OH)D
APO-A1	rs1229984	4	C	T	0.970238	-0.0760602	0.00658526	2.19989e-35	CA, Serum-25(OH)D
APO-A1	rs1260326	2	C	T	0.598524	-0.0389373	0.00210774	4.10015e-83	CA, Serum-25(OH)D

APO-A1	rs12619647	2	G	T	0.321626	0.0160065	0.00221665	2.19989e-14	CA, Serum-25(OH)D
APO-A1	rs12729444	1	G	T	0.0814647	-0.048616	0.00372002	1.59993e-44	CA, Serum-25(OH)D
APO-A1	rs12740374	1	T	G	0.221017	0.0529442	0.00245205	2.30144e-111	CA, Serum-25(OH)D
APO-A1	rs1281958	6	A	C	0.419766	0.0119644	0.00207835	2.19999e-09	CA, Serum-25(OH)D
APO-A1	rs12951098	17	G	A	0.17717	-0.0153094	0.00269791	4.39997e-08	CA, Serum-25(OH)D
APO-A1	rs13056506	22	T	G	0.598448	-0.0251268	0.00209878	2.70023e-35	CA, Serum-25(OH)D
APO-A1	rs13083133	3	C	T	0.0919466	0.0255408	0.00353976	4.49987e-13	CA, Serum-25(OH)D
APO-A1	rs13107325	4	T	C	0.0749578	-0.0666067	0.00395031	1.50003e-70	CA, Serum-25(OH)D
APO-A1	rs13273592	8	C	T	0.28628	-0.0140742	0.00225784	4.60045e-11	CA, Serum-25(OH)D
APO-A1	rs1432603	3	T	C	0.764412	0.0121255	0.00241112	2.19999e-08	CA, Serum-25(OH)D
APO-A1	rs14347	16	G	T	0.293193	-0.0183478	0.00223134	1e-16	CA, Serum-25(OH)D
APO-A1	rs1446585	2	G	A	0.235986	0.0180024	0.00253898	1.69981e-15	CA, Serum-25(OH)D
APO-A1	rs145594822	14	AT	A	0.55066	-0.0165162	0.00207934	2.09991e-14	CA, Serum-25(OH)D
APO-A1	rs15285	8	T	C	0.285872	0.104055	0.00225893	1e-200	CA, Serum-25(OH)D
APO-A1	rs1531517	19	A	G	0.0724193	0.0746751	0.00393576	1.80011e-86	CA, Serum-25(OH)D
APO-A1	rs1534696	7	A	C	0.541243	0.0134022	0.00205659	1.20005e-11	CA, Serum-25(OH)D
APO-A1	rs1672867	16	G	A	0.979461	0.117568	0.00797273	6.79986e-53	CA, Serum-25(OH)D
APO-A1	rs1689800	1	G	A	0.359528	-0.0157951	0.00211851	3.19963e-14	CA, Serum-25(OH)D
APO-A1	rs16942887	16	A	G	0.114807	0.0687787	0.00316501	2.69774e-115	CA, Serum-25(OH)D
APO-A1	rs16999141	14	A	G	0.476794	0.0129545	0.00206849	1.10002e-12	CA, Serum-25(OH)D
APO-A1	rs17050272	2	A	G	0.409839	0.0117628	0.00207543	4.79999e-09	CA, Serum-25(OH)D
APO-A1	rs17106304	14	G	C	0.653339	-0.016324	0.00217631	2.90001e-14	CA, Serum-25(OH)D
APO-A1	rs17129638	10	C	T	0.148127	-0.0342985	0.00288443	4.30031e-35	CA, Serum-25(OH)D
APO-A1	rs17504495	1	C	T	0.235587	-0.0152656	0.00240033	4.19952e-12	CA, Serum-25(OH)D
APO-A1	rs17630640	6	G	A	0.131287	0.0173574	0.00303568	1.09999e-09	CA, Serum-25(OH)D
APO-A1	rs17713557	2	T	G	0.065163	-0.0250131	0.00413364	4.79999e-09	CA, Serum-25(OH)D

APO-A1	rs1800961	20	T	C	0.0308439	-0.176871	0.00604067	1e-200	CA, Serum-25(OH)D
APO-A1	rs1892534	1	T	C	0.374341	0.0149626	0.00210207	2.39994e-13	CA, Serum-25(OH)D
APO-A1	rs1902023	4	C	A	0.47369	0.0218509	0.00207123	1.69981e-28	CA, Serum-25(OH)D
APO-A1	rs2048508	4	C	T	0.175748	-0.0227435	0.00269268	2.60016e-19	CA, Serum-25(OH)D
APO-A1	rs204906	19	T	C	0.0329554	0.0741014	0.00572476	3.29989e-41	CA, Serum-25(OH)D
APO-A1	rs2066716	9	T	C	0.0820637	0.0587018	0.00375212	1.10002e-60	CA, Serum-25(OH)D
APO-A1	rs2066718	9	T	C	0.0287119	0.142253	0.0061542	3.1989e-131	CA, Serum-25(OH)D
APO-A1	rs2068888	10	A	G	0.450587	0.0169827	0.00205994	3.59998e-16	CA, Serum-25(OH)D
APO-A1	rs2071410	15	G	C	0.312673	0.0127398	0.00223528	6.59994e-09	CA, Serum-25(OH)D
APO-A1	rs2081687	8	C	T	0.662995	-0.0129781	0.00215783	2.5e-09	CA, Serum-25(OH)D
APO-A1	rs2112161	5	A	G	0.719957	0.0142085	0.00228144	6.29941e-12	CA, Serum-25(OH)D
APO-A1	rs2153271	9	T	C	0.611475	0.012004	0.00209499	2.1e-08	CA, Serum-25(OH)D
APO-A1	rs2224933	1	G	A	0.570082	0.0111999	0.00205241	2.90001e-08	CA, Serum-25(OH)D
APO-A1	rs2229357	12	A	G	0.240178	0.0328327	0.00240585	2.99985e-45	CA, Serum-25(OH)D
APO-A1	rs2255141	10	G	A	0.72454	-0.042881	0.00229496	3.80014e-84	CA, Serum-25(OH)D
APO-A1	rs2270788	12	C	T	0.0979653	0.0463226	0.00348726	1.69981e-42	CA, Serum-25(OH)D
APO-A1	rs2274892	17	G	T	0.362934	-0.0158013	0.00214126	8.10028e-14	CA, Serum-25(OH)D
APO-A1	rs2275086	1	G	C	0.698249	0.0149114	0.00222855	3.50026e-12	CA, Serum-25(OH)D
APO-A1	rs2278426	19	T	C	0.0347101	-0.128331	0.00565683	4.39542e-124	CA, Serum-25(OH)D
APO-A1	rs2279574	12	A	C	0.537796	-0.0149794	0.00207627	8.10028e-14	CA, Serum-25(OH)D
APO-A1	rs2286660	11	G	T	0.643836	0.0123428	0.00215081	1.40001e-08	CA, Serum-25(OH)D
APO-A1	rs2291428	10	C	G	0.238803	0.0405493	0.00241105	1.39991e-66	CA, Serum-25(OH)D
APO-A1	rs2292137	12	G	C	0.355063	-0.0184147	0.00220294	1.59993e-17	CA, Serum-25(OH)D
APO-A1	rs2293476	1	C	G	0.22767	-0.0404332	0.00242979	1.9002e-66	CA, Serum-25(OH)D
APO-A1	rs2298214	1	A	C	0.567547	-0.0136997	0.00210443	8.80035e-12	CA, Serum-25(OH)D
APO-A1	rs2298624	18	T	C	0.132207	0.0376002	0.00302984	2.70023e-35	CA, Serum-25(OH)D

APO-A1	rs2301179	10	G	A	0.493011	-0.011714	0.00207982	1.29999e-08	CA, Serum-25(OH)D
APO-A1	rs2303361	7	C	T	0.238111	0.0363883	0.00241474	5.00035e-58	CA, Serum-25(OH)D
APO-A1	rs2315129	6	A	T	0.161391	-0.0285364	0.0028214	7.19946e-26	CA, Serum-25(OH)D
APO-A1	rs235314	21	T	C	0.530902	-0.0234187	0.00207094	8.4004e-32	CA, Serum-25(OH)D
APO-A1	rs2395045	6	T	C	0.75603	-0.0221767	0.00368027	4.90004e-10	CA, Serum-25(OH)D
APO-A1	rs2395943	6	G	A	0.587674	0.0325075	0.00208046	1.29987e-57	CA, Serum-25(OH)D
APO-A1	rs2424993	20	C	G	0.696866	-0.0180032	0.00224283	3.50026e-15	CA, Serum-25(OH)D
APO-A1	rs2494748	14	T	C	0.610463	-0.0390385	0.00213443	5.10035e-80	CA, Serum-25(OH)D
APO-A1	rs2575876	9	A	G	0.25232	-0.0945542	0.00235229	1e-200	CA, Serum-25(OH)D
APO-A1	rs2636897	9	C	A	0.548234	0.0148865	0.00205425	9.3994e-14	CA, Serum-25(OH)D
APO-A1	rs2642438	1	G	A	0.700859	0.0368828	0.0022383	3.90032e-65	CA, Serum-25(OH)D
APO-A1	rs267738	1	G	T	0.218359	0.0418301	0.00247258	2.70023e-66	CA, Serum-25(OH)D
APO-A1	rs2768541	6	G	A	0.794504	-0.019461	0.00253273	2.60016e-15	CA, Serum-25(OH)D
APO-A1	rs2777938	20	T	C	0.481939	-0.0135735	0.00205341	1.69981e-11	CA, Serum-25(OH)D
APO-A1	rs2810489	9	A	G	0.258298	0.0136343	0.0023348	6.29999e-09	CA, Serum-25(OH)D
APO-A1	rs2811929	9	A	G	0.617897	0.0117589	0.00211038	1.2e-08	CA, Serum-25(OH)D
APO-A1	rs2814982	6	T	C	0.102332	-0.027485	0.00338884	5.70033e-18	CA, Serum-25(OH)D
APO-A1	rs2820441	1	C	A	0.316518	0.0157872	0.0021856	1.99986e-14	CA, Serum-25(OH)D
APO-A1	rs2862954	10	C	T	0.495555	0.0280807	0.00205525	6.20012e-43	CA, Serum-25(OH)D
APO-A1	rs2923110	11	G	A	0.701704	0.0232276	0.00222502	4.40048e-26	CA, Serum-25(OH)D
APO-A1	rs2925979	16	C	T	0.699812	0.030974	0.00220373	1e-49	CA, Serum-25(OH)D
APO-A1	rs2928619	8	T	C	0.65386	0.0121076	0.00214331	1.09999e-09	CA, Serum-25(OH)D
APO-A1	rs2943641	2	C	T	0.648184	-0.0388056	0.00213332	6.59933e-80	CA, Serum-25(OH)D
APO-A1	rs2954020	8	G	A	0.684469	0.0183877	0.0021953	2.60016e-18	CA, Serum-25(OH)D
APO-A1	rs30380	5	C	A	0.641523	0.0142974	0.00218024	1.50003e-12	CA, Serum-25(OH)D
APO-A1	rs330089	8	C	T	0.0980118	0.0236867	0.00343227	7.19946e-13	CA, Serum-25(OH)D

APO-A1	rs34108083	8	G	T	0.0992972	-0.0208858	0.00340574	1e-10	CA, Serum-25(OH)D
APO-A1	rs34145453	5	G	A	0.367581	0.0208847	0.00212639	2.80027e-23	CA, Serum-25(OH)D
APO-A1	rs34176120	7	C	T	0.228239	-0.0205937	0.00244007	1.69981e-18	CA, Serum-25(OH)D
APO-A1	rs34293391	19	C	T	0.0283601	0.0407005	0.00631396	2.69998e-09	CA, Serum-25(OH)D
APO-A1	rs34810344	3	A	G	0.189622	0.0166975	0.00262017	1.89998e-10	CA, Serum-25(OH)D
APO-A1	rs34931250	17	T	C	0.057907	-0.0575215	0.00455302	2.49977e-37	CA, Serum-25(OH)D
APO-A1	rs35078364	2	G	A	0.436757	0.0105136	0.00205349	5.49997e-09	CA, Serum-25(OH)D
APO-A1	rs355770	3	A	G	0.84523	-0.0169197	0.00283329	9.70063e-11	CA, Serum-25(OH)D
APO-A1	rs35616673	7	CT	C	0.168928	-0.0175902	0.00275722	5.79963e-11	CA, Serum-25(OH)D
APO-A1	rs35853021	15	T	G	0.357654	0.141599	0.00212165	1e-200	CA, Serum-25(OH)D
APO-A1	rs372883	21	C	T	0.486631	0.0117128	0.00206259	1.5e-09	CA, Serum-25(OH)D
APO-A1	rs3732356	3	T	G	0.930801	-0.0400475	0.00415046	5.90065e-23	CA, Serum-25(OH)D
APO-A1	rs374033872	5	GT	G	0.353847	0.0120234	0.0021445	4e-08	CA, Serum-25(OH)D
APO-A1	rs3750733	10	A	G	0.0917742	-0.0237735	0.00358172	5.10035e-11	CA, Serum-25(OH)D
APO-A1	rs3752440	4	A	G	0.083505	0.0269207	0.00377784	2.70023e-13	CA, Serum-25(OH)D
APO-A1	rs3756772	6	T	C	0.400143	0.0207563	0.00209707	1.10002e-24	CA, Serum-25(OH)D
APO-A1	rs3758938	11	G	T	0.309061	0.0139047	0.0022242	1.7e-09	CA, Serum-25(OH)D
APO-A1	rs3761706	2	A	G	0.0629055	-0.0226389	0.00420847	2e-08	CA, Serum-25(OH)D
APO-A1	rs3764261	16	A	C	0.323472	0.208561	0.00216011	1e-200	CA, Serum-25(OH)D
APO-A1	rs3770589	2	T	C	0.550053	-0.00994626	0.00205218	1.7e-08	CA, Serum-25(OH)D
APO-A1	rs3779234	7	T	C	0.373159	-0.014913	0.00213206	8.30042e-14	CA, Serum-25(OH)D
APO-A1	rs3779881	8	T	C	0.561699	0.0351782	0.00205741	1.69981e-70	CA, Serum-25(OH)D
APO-A1	rs3790414	1	A	T	0.229507	0.0138213	0.00241851	8.79995e-09	CA, Serum-25(OH)D
APO-A1	rs3815148	7	C	A	0.226725	0.0233916	0.00244807	7.10068e-22	CA, Serum-25(OH)D
APO-A1	rs3822072	4	A	G	0.450819	-0.0181096	0.00205801	9.79941e-20	CA, Serum-25(OH)D
APO-A1	rs3825061	11	T	C	0.386051	-0.0168278	0.00217881	1.50003e-14	CA, Serum-25(OH)D

APO-A1	rs3827385	22	C	T	0.179643	-0.0175239	0.00267966	3.59998e-12	CA, Serum-25(OH)D
APO-A1	rs3829125	10	G	C	0.153635	-0.0269987	0.00285331	2.29985e-26	CA, Serum-25(OH)D
APO-A1	rs3912391	4	A	G	0.507128	0.0183422	0.00204959	2.49977e-20	CA, Serum-25(OH)D
APO-A1	rs3995817	16	T	C	0.104931	0.0402521	0.00331265	3.69999e-34	CA, Serum-25(OH)D
APO-A1	rs4129767	17	A	G	0.489536	0.0331847	0.00205006	2.09991e-64	CA, Serum-25(OH)D
APO-A1	rs41298373	10	A	G	0.0994952	-0.023315	0.00361643	1.5e-10	CA, Serum-25(OH)D
APO-A1	rs413458	15	G	C	0.728359	0.0338596	0.00228472	1.9002e-55	CA, Serum-25(OH)D
APO-A1	rs4146466	8	G	A	0.900796	0.021761	0.00340795	1e-10	CA, Serum-25(OH)D
APO-A1	rs4239651	20	C	T	0.794031	0.0292668	0.00254228	8.19974e-32	CA, Serum-25(OH)D
APO-A1	rs4240199	2	G	A	0.586978	0.0161662	0.00208626	4.90004e-17	CA, Serum-25(OH)D
APO-A1	rs429358	19	C	T	0.153855	-0.132441	0.0028456	1e-200	CA, Serum-25(OH)D
APO-A1	rs4409785	11	C	T	0.172467	-0.019252	0.00269926	2.49977e-13	CA, Serum-25(OH)D
APO-A1	rs4563251	2	C	T	0.391948	0.0138339	0.00209025	5.40008e-11	CA, Serum-25(OH)D
APO-A1	rs459193	5	G	A	0.747045	-0.0240949	0.00235311	3.90032e-27	CA, Serum-25(OH)D
APO-A1	rs4715210	6	T	C	0.169586	0.0177133	0.00273153	1e-12	CA, Serum-25(OH)D
APO-A1	rs471760	1	A	G	0.333099	-0.0225889	0.00215757	6.09958e-26	CA, Serum-25(OH)D
APO-A1	rs4729655	7	C	T	0.512031	0.0126882	0.00205199	3.2e-09	CA, Serum-25(OH)D
APO-A1	rs4731702	7	T	C	0.493124	0.0287215	0.00204949	1.39991e-48	CA, Serum-25(OH)D
APO-A1	rs4795554	17	C	T	0.507876	0.0157985	0.00205335	3.29989e-16	CA, Serum-25(OH)D
APO-A1	rs4804573	19	A	G	0.468738	0.0228039	0.00206768	7.00003e-30	CA, Serum-25(OH)D
APO-A1	rs4821124	22	C	T	0.188871	-0.0408051	0.00262788	4.49987e-58	CA, Serum-25(OH)D
APO-A1	rs4841132	8	G	A	0.908754	0.107681	0.00376507	3.09742e-189	CA, Serum-25(OH)D
APO-A1	rs4846914	1	A	G	0.605978	0.0605646	0.00208361	1.99986e-199	CA, Serum-25(OH)D
APO-A1	rs4850047	2	C	T	0.868332	-0.026909	0.00301352	3.40017e-20	CA, Serum-25(OH)D
APO-A1	rs4855697	3	G	A	0.430621	0.0191385	0.00206793	1e-21	CA, Serum-25(OH)D
APO-A1	rs4883201	12	G	A	0.102258	-0.0319227	0.00337906	3.90032e-21	CA, Serum-25(OH)D

APO-A1	rs4917014	7	G	T	0.318694	0.0200572	0.00220203	1.20005e-20	CA, Serum-25(OH)D
APO-A1	rs4939883	18	C	T	0.820096	0.115687	0.00266788	1e-200	CA, Serum-25(OH)D
APO-A1	rs4951261	1	C	A	0.393329	-0.0105399	0.00208094	1.40001e-09	CA, Serum-25(OH)D
APO-A1	rs4953618	2	G	A	0.267467	0.0131633	0.0023064	2.59998e-10	CA, Serum-25(OH)D
APO-A1	rs499974	11	A	C	0.156824	-0.0525085	0.0028016	6.70039e-81	CA, Serum-25(OH)D
APO-A1	rs5093	11	A	G	0.0186991	0.0961502	0.00757644	9.3994e-40	CA, Serum-25(OH)D
APO-A1	rs526936	1	A	G	0.518133	0.0222341	0.00203803	1.39991e-31	CA, Serum-25(OH)D
APO-A1	rs554202	11	G	A	0.46074	0.0125388	0.00204803	1.89998e-10	CA, Serum-25(OH)D
APO-A1	rs55707100	15	T	C	0.0256911	-0.0933246	0.00644681	7.59976e-48	CA, Serum-25(OH)D
APO-A1	rs56398830	13	A	G	0.0111238	0.0767204	0.010024	7.10068e-16	CA, Serum-25(OH)D
APO-A1	rs5763301	22	A	G	0.226374	-0.0141519	0.00245651	3.29997e-09	CA, Serum-25(OH)D
APO-A1	rs581465	6	C	T	0.406275	0.0130406	0.00208551	6.09958e-11	CA, Serum-25(OH)D
APO-A1	rs5888	12	G	A	0.510347	-0.0348608	0.00206489	2.29985e-69	CA, Serum-25(OH)D
APO-A1	rs6076	15	A	G	0.168564	0.0295162	0.00278879	1.29987e-26	CA, Serum-25(OH)D
APO-A1	rs6089070	20	A	G	0.212435	0.0157918	0.00250996	1.29999e-09	CA, Serum-25(OH)D
APO-A1	rs60960031	2	A	G	0.401871	0.0114717	0.00208324	8.70001e-10	CA, Serum-25(OH)D
APO-A1	rs6123685	20	A	G	0.254778	0.0192972	0.00236164	5.19996e-15	CA, Serum-25(OH)D
APO-A1	rs6151483	15	G	A	0.361876	0.0120002	0.00214779	1.09999e-09	CA, Serum-25(OH)D
APO-A1	rs61587156	3	G	T	0.185395	-0.0302461	0.00263401	2.09991e-33	CA, Serum-25(OH)D
APO-A1	rs61747585	14	A	C	0.0415696	-0.0300739	0.00516687	2.39999e-09	CA, Serum-25(OH)D
APO-A1	rs632057	6	G	T	0.627673	0.0185927	0.00211917	2.29985e-21	CA, Serum-25(OH)D
APO-A1	rs643531	9	A	C	0.864461	0.0618326	0.00298341	2.39883e-102	CA, Serum-25(OH)D
APO-A1	rs645040	3	T	G	0.773057	-0.0319973	0.00244298	4.90004e-41	CA, Serum-25(OH)D
APO-A1	rs660716	18	A	G	0.608333	-0.0174429	0.00209935	1.59993e-17	CA, Serum-25(OH)D
APO-A1	rs676210	2	A	G	0.2043	0.0732926	0.00253663	3.09742e-200	CA, Serum-25(OH)D
APO-A1	rs6773682	3	A	G	0.346017	-0.0119135	0.00215063	1.40001e-08	CA, Serum-25(OH)D

APO-A1	rs679995	17	T	A	0.239054	-0.0300207	0.00244467	3.19963e-35	CA, Serum-25(OH)D
APO-A1	rs6811376	4	A	G	0.777186	0.0157582	0.00246339	6.69993e-10	CA, Serum-25(OH)D
APO-A1	rs6847544	4	G	T	0.260907	-0.0149692	0.00233131	9.09913e-11	CA, Serum-25(OH)D
APO-A1	rs6866471	5	A	G	0.627471	-0.0150012	0.00212505	1.39991e-14	CA, Serum-25(OH)D
APO-A1	rs6867836	5	G	A	0.364095	-0.0118228	0.00213012	5.19996e-09	CA, Serum-25(OH)D
APO-A1	rs6887101	5	G	A	0.558973	0.0121566	0.00206315	8.10009e-10	CA, Serum-25(OH)D
APO-A1	rs6926219	6	A	G	0.553734	0.0109127	0.00209244	1.40001e-08	CA, Serum-25(OH)D
APO-A1	rs703974	10	G	A	0.413983	0.0152433	0.00207997	4.40048e-13	CA, Serum-25(OH)D
APO-A1	rs7113826	11	G	T	0.432111	-0.0119987	0.00205384	5.60003e-10	CA, Serum-25(OH)D
APO-A1	rs7133378	12	A	G	0.316233	0.0406513	0.00220974	4.90004e-77	CA, Serum-25(OH)D
APO-A1	rs7134375	12	A	C	0.431348	0.0197414	0.00206266	7.89951e-24	CA, Serum-25(OH)D
APO-A1	rs7134972	12	C	T	0.9468	-0.0317848	0.00455287	2.99985e-12	CA, Serum-25(OH)D
APO-A1	rs7166701	15	G	A	0.127183	-0.0189307	0.00304907	5e-09	CA, Serum-25(OH)D
APO-A1	rs7197475	16	T	C	0.363228	0.0121241	0.00209688	5.19996e-09	CA, Serum-25(OH)D
APO-A1	rs723177	1	C	T	0.698559	0.0131473	0.00222023	1.6e-09	CA, Serum-25(OH)D
APO-A1	rs72729610	4	G	A	0.165558	-0.0166616	0.00275822	7.00003e-12	CA, Serum-25(OH)D
APO-A1	rs72733748	14	G	T	0.349394	-0.0119986	0.00215623	5.1e-09	CA, Serum-25(OH)D
APO-A1	rs72801474	5	A	G	0.0922392	0.0189859	0.00354994	2.5e-08	CA, Serum-25(OH)D
APO-A1	rs72836561	17	T	C	0.0313646	-0.175936	0.00594141	1e-200	CA, Serum-25(OH)D
APO-A1	rs72961013	6	A	G	0.0657901	-0.035519	0.00413683	3.19963e-17	CA, Serum-25(OH)D
APO-A1	rs7298565	12	A	G	0.521202	0.0313874	0.00205478	4.49987e-57	CA, Serum-25(OH)D
APO-A1	rs731839	19	A	G	0.665206	0.0292931	0.00216536	7.39946e-46	CA, Serum-25(OH)D
APO-A1	rs7337877	13	C	T	0.402173	0.015599	0.00209418	9.09913e-15	CA, Serum-25(OH)D
APO-A1	rs735144	16	G	A	0.430916	0.0444626	0.0020404	2.79898e-115	CA, Serum-25(OH)D
APO-A1	rs73667430	8	T	C	0.0160587	-0.0808669	0.00813011	3.40017e-24	CA, Serum-25(OH)D
APO-A1	rs74780677	3	G	A	0.0150886	-0.0970314	0.00859217	2.80027e-31	CA, Serum-25(OH)D

APO-A1	rs7483	1	T	C	0.300041	0.0146802	0.00224533	3.50026e-11	CA, Serum-25(OH)D
APO-A1	rs75257814	16	C	T	0.057861	-0.0524035	0.00433227	3.50026e-34	CA, Serum-25(OH)D
APO-A1	rs75297654	2	T	C	0.120018	0.0381106	0.00313882	1.59993e-38	CA, Serum-25(OH)D
APO-A1	rs75429795	5	T	G	0.139728	-0.0140983	0.00296132	4.90004e-08	CA, Serum-25(OH)D
APO-A1	rs754671	19	T	C	0.699403	0.0244118	0.00223066	1.99986e-27	CA, Serum-25(OH)D
APO-A1	rs75688370	2	G	C	0.0124679	-0.0540189	0.00921628	6.29999e-09	CA, Serum-25(OH)D
APO-A1	rs76026733	5	C	T	0.0527638	-0.0362546	0.00458371	3.50026e-15	CA, Serum-25(OH)D
APO-A1	rs76035382	10	A	G	0.134903	-0.0195462	0.00299772	6.20012e-12	CA, Serum-25(OH)D
APO-A1	rs762707	3	T	C	0.0180364	0.0442072	0.00785432	3.09999e-08	CA, Serum-25(OH)D
APO-A1	rs76856822	18	C	T	0.0173191	0.0573163	0.00785086	2.90001e-14	CA, Serum-25(OH)D
APO-A1	rs7697204	4	T	C	0.742011	-0.0132457	0.0023438	6.59994e-10	CA, Serum-25(OH)D
APO-A1	rs77169818	18	T	A	0.0418289	-0.0271418	0.00512925	2.19999e-08	CA, Serum-25(OH)D
APO-A1	rs77960347	18	G	A	0.0131072	0.405131	0.00906045	1e-200	CA, Serum-25(OH)D
APO-A1	rs7817574	8	C	T	0.184957	0.0419044	0.00262364	2.70023e-57	CA, Serum-25(OH)D
APO-A1	rs78710178	4	A	C	0.0111232	-0.0655647	0.00978681	4.49987e-12	CA, Serum-25(OH)D
APO-A1	rs7874056	9	G	T	0.106877	-0.0222951	0.00330842	1.2e-10	CA, Serum-25(OH)D
APO-A1	rs80255063	16	C	T	0.0412354	0.033442	0.00508007	1e-10	CA, Serum-25(OH)D
APO-A1	rs8029952	15	C	T	0.320793	0.0159859	0.00217441	1.99986e-14	CA, Serum-25(OH)D
APO-A1	rs8064100	16	G	A	0.419964	-0.0209091	0.00204561	1.50003e-24	CA, Serum-25(OH)D
APO-A1	rs870526	2	T	C	0.523289	-0.0254633	0.00204236	6.20012e-37	CA, Serum-25(OH)D
APO-A1	rs871841	17	C	T	0.492229	0.012785	0.00210789	6.29941e-11	CA, Serum-25(OH)D
APO-A1	rs900400	3	C	T	0.399545	0.0200215	0.00209368	6.59933e-24	CA, Serum-25(OH)D
APO-A1	rs901746	11	G	A	0.301093	0.0382211	0.00222733	2.09991e-72	CA, Serum-25(OH)D
APO-A1	rs910798	22	G	A	0.888789	0.0182492	0.0034085	2.69998e-08	CA, Serum-25(OH)D
APO-A1	rs9315781	13	C	T	0.295174	-0.0128852	0.00225008	2.30001e-10	CA, Serum-25(OH)D
APO-A1	rs9321572	6	A	G	0.589934	0.0131055	0.00208425	5.40008e-11	CA, Serum-25(OH)D

APO-A1	rs9427104	1	T	C	0.478509	0.0177987	0.00203597	1.20005e-20	CA, Serum-25(OH)D
APO-A1	rs9508721	13	T	C	0.949087	0.0295983	0.00468134	3.2e-09	CA, Serum-25(OH)D
APO-A1	rs954090	12	G	A	0.324844	0.0129242	0.0021848	4.20001e-09	CA, Serum-25(OH)D
APO-A1	rs976002	4	G	A	0.243633	0.0260174	0.00241007	2.39994e-28	CA, Serum-25(OH)D
APO-A1	rs9834	5	C	T	0.881041	-0.0210917	0.00328021	5.70033e-11	CA, Serum-25(OH)D
APO-A1	rs9837573	3	C	T	0.664463	0.0173386	0.00216677	3.80014e-16	CA, Serum-25(OH)D
APO-A1	rs987044	12	G	A	0.425707	-0.0122615	0.00206778	4.30002e-10	CA, Serum-25(OH)D
APO-A1	rs9901671	17	C	T	0.601674	-0.01577	0.00209306	1.29987e-15	CA, Serum-25(OH)D
APO-A1	rs9923575	16	T	C	0.194123	0.0177888	0.00254905	1.69981e-13	CA, Serum-25(OH)D
APO-A1	rs9953528	18	A	G	0.246307	-0.0204754	0.00237248	4.60045e-19	CA, Serum-25(OH)D
APO-A1	rs9977268	21	T	C	0.197169	-0.0207572	0.00260211	6.20012e-18	CA, Serum-25(OH)D
APO-B	rs10096633	8	T	C	0.123907	-0.0406003	0.00624844	8.19974e-11	CA, Serum-25(OH)D
APO-B	rs102275	11	C	T	0.34979	-0.0393688	0.00431065	6.70039e-20	CA, Serum-25(OH)D
APO-B	rs10260606	7	C	G	0.182652	0.0327546	0.00532405	7.59994e-10	CA, Serum-25(OH)D
APO-B	rs10504255	8	A	G	0.662958	-0.0267141	0.00437679	1e-09	CA, Serum-25(OH)D
APO-B	rs1065853	19	T	G	0.080581	-0.45074	0.00758806	1e-200	CA, Serum-25(OH)D
APO-B	rs1081105	19	C	A	0.027621	0.166668	0.0125821	4.70002e-40	CA, Serum-25(OH)D
APO-B	rs111278137	19	A	G	0.021614	-0.113131	0.0146428	1.10002e-14	CA, Serum-25(OH)D
APO-B	rs11206517	1	G	T	0.033012	0.0952718	0.0115367	1.50003e-16	CA, Serum-25(OH)D
APO-B	rs112875651	8	A	G	0.392379	-0.0668373	0.00428097	6.00067e-55	CA, Serum-25(OH)D
APO-B	rs115478735	9	T	A	0.183304	0.0567209	0.00533262	1.99986e-26	CA, Serum-25(OH)D
APO-B	rs115740542	6	C	T	0.073601	-0.0494022	0.00793082	4.70002e-10	CA, Serum-25(OH)D
APO-B	rs11591147	1	T	G	0.017457	-0.323815	0.015704	1.80011e-94	CA, Serum-25(OH)D
APO-B	rs12151108	19	A	G	0.119677	-0.158333	0.00633223	5.49541e-138	CA, Serum-25(OH)D
APO-B	rs12208357	6	T	C	0.07	0.0677003	0.0081062	6.70039e-17	CA, Serum-25(OH)D
APO-B	rs12620918	2	C	T	0.173985	0.0299356	0.00544745	3.89996e-08	CA, Serum-25(OH)D

APO-B	rs12740374	1	T	G	0.221064	-0.115903	0.0049586	7.7983e-121	CA, Serum-25(OH)D
APO-B	rs12804228	11	G	A	0.08472	0.0561202	0.00746086	5.40008e-14	CA, Serum-25(OH)D
APO-B	rs12916	5	C	T	0.400341	0.0590342	0.00420817	1e-44	CA, Serum-25(OH)D
APO-B	rs1499279	5	G	T	0.041623	-0.0596608	0.0103591	8.40001e-09	CA, Serum-25(OH)D
APO-B	rs1883711	20	C	G	0.031207	0.105018	0.0121099	4.19952e-18	CA, Serum-25(OH)D
APO-B	rs247616	16	T	C	0.324699	-0.0561441	0.00440689	3.50026e-37	CA, Serum-25(OH)D
APO-B	rs261290	15	C	T	0.654645	-0.0536278	0.00434549	5.40008e-35	CA, Serum-25(OH)D
APO-B	rs2618566	20	T	G	0.660294	-0.0240134	0.00435304	3.50002e-08	CA, Serum-25(OH)D
APO-B	rs2737245	8	T	G	0.278602	-0.0273596	0.00460812	2.90001e-09	CA, Serum-25(OH)D
APO-B	rs2738447	19	C	A	0.59318	0.0363862	0.00419589	4.30031e-18	CA, Serum-25(OH)D
APO-B	rs28807203	19	C	A	0.049207	-0.119257	0.00955395	9.30037e-36	CA, Serum-25(OH)D
APO-B	rs34042070	16	G	C	0.186978	0.0454725	0.00530804	1.10002e-17	CA, Serum-25(OH)D
APO-B	rs4299376	2	T	G	0.676277	-0.0478895	0.00441406	1.99986e-27	CA, Serum-25(OH)D
APO-B	rs4665972	2	C	T	0.604609	-0.0378495	0.00423902	4.30031e-19	CA, Serum-25(OH)D
APO-B	rs4704834	5	G	A	0.644028	0.0449162	0.00429985	1.50003e-25	CA, Serum-25(OH)D
APO-B	rs4935356	6	A	T	0.244158	0.0313287	0.00507999	7.00003e-10	CA, Serum-25(OH)D
APO-B	rs553427	1	T	C	0.517037	0.0309179	0.00412208	6.4003e-14	CA, Serum-25(OH)D
APO-B	rs553741	1	C	G	0.645268	0.0322686	0.00431766	7.8001e-14	CA, Serum-25(OH)D
APO-B	rs55714927	17	T	C	0.190994	-0.0295577	0.00524256	1.7e-08	CA, Serum-25(OH)D
APO-B	rs56325564	17	A	G	0.482691	0.0332508	0.00415357	1.20005e-15	CA, Serum-25(OH)D
APO-B	rs58542926	19	T	C	0.074399	-0.114534	0.00784275	2.70023e-48	CA, Serum-25(OH)D
APO-B	rs633695	15	G	A	0.292354	0.0406651	0.00454959	4.00037e-19	CA, Serum-25(OH)D
APO-B	rs6601299	8	C	T	0.899281	0.0387853	0.00685675	1.5e-08	CA, Serum-25(OH)D
APO-B	rs6602909	13	C	T	0.328942	0.0272472	0.00439206	5.49997e-10	CA, Serum-25(OH)D
APO-B	rs6920309	6	T	C	0.456849	-0.0243583	0.00415082	4.39997e-09	CA, Serum-25(OH)D
APO-B	rs693	2	A	G	0.520889	0.0877224	0.00412312	1.9002e-100	CA, Serum-25(OH)D

APO-B	rs72631343	17	G	C	0.129021	-0.0436946	0.00615144	1.20005e-12	CA, Serum-25(OH)D
APO-B	rs72694391	14	C	T	0.482108	0.0249671	0.00413713	1.6e-09	CA, Serum-25(OH)D
APO-B	rs7534572	1	G	C	0.64747	0.0426133	0.00430754	4.49987e-23	CA, Serum-25(OH)D
APO-B	rs7610507	3	G	C	0.786528	0.0283443	0.00504011	1.89998e-08	CA, Serum-25(OH)D
APO-B	rs7704651	5	A	G	0.331143	0.0248561	0.00438511	1.40001e-08	CA, Serum-25(OH)D
APO-B	rs7707394	5	A	G	0.357075	0.0380553	0.00429472	7.89951e-19	CA, Serum-25(OH)D
APO-B	rs77542162	17	G	A	0.022666	0.144912	0.0138643	1.39991e-25	CA, Serum-25(OH)D
APO-B	rs8103315	19	A	C	0.135788	0.0485477	0.00618103	4.00037e-15	CA, Serum-25(OH)D
APO-B	rs9295128	6	T	G	0.016821	-0.123375	0.0163146	4.00037e-14	CA, Serum-25(OH)D
APO-B	rs9616847	22	T	A	0.388272	0.0243827	0.00424955	9.59997e-09	CA, Serum-25(OH)D
APO-B	rs964184	11	C	G	0.86726	-0.0756427	0.00607626	1.39991e-35	CA, Serum-25(OH)D
Serum-25(OH)D	rs10277163	7	G	A	0.254715	-0.0143336	0.0023507	1.07676e-09	CA,LDL-C,HDL-C,TG,APO-A1,APO-B
Serum-25(OH)D	rs1038165	12	T	C	0.579451	0.0115149	0.00205629	2.14541e-08	CA,LDL-C,HDL-C,TG,APO-A1,APO-B
Serum-25(OH)D	rs1042034	2	T	C	0.792138	-0.0151254	0.00250017	1.45071e-09	CA,LDL-C,HDL-C,TG,APO-A1,APO-B
Serum-25(OH)D	rs10438978	18	C	T	0.820277	-0.0172243	0.00264437	7.33838e-11	CA,LDL-C,HDL-C,TG,APO-A1,APO-B
Serum-25(OH)D	rs1047891	2	A	C	0.317078	-0.0133984	0.00218016	7.96453e-10	CA,LDL-C,HDL-C,TG,APO-A1,APO-B
Serum-25(OH)D	rs1048328	19	A	G	0.0797967	0.0313497	0.00374376	5.57571e-17	CA,LDL-C,HDL-C,TG,APO-A1,APO-B
Serum-25(OH)D	rs10859995	12	C	T	0.579841	-0.0436264	0.00205452	4.60045e-100	CA,LDL-C,HDL-C,TG,APO-A1,APO-B
Serum-25(	rs11023159	11	C	T	0.0325143	0.0482117	0.00572507	3.72735e-17	CA,LDL-C,HDL-C,TG,AP

OH)D									O-A1,APO-B
Serum-25(OH)D	rs11076175	16	G	A	0.175666	0.0229033	0.00266997	9.64273e-18	CA,LDL-C,HDL-C,TG,APO-A1,APO-B
Serum-25(OH)D	rs111515741	11	A	G	0.0173081	-0.0487364	0.00779044	3.95185e-10	CA,LDL-C,HDL-C,TG,APO-A1,APO-B
Serum-25(OH)D	rs11207969	1	G	A	0.351365	0.0209396	0.00212672	7.1351e-23	CA,LDL-C,HDL-C,TG,APO-A1,APO-B
Serum-25(OH)D	rs11264361	1	G	T	0.251408	0.0174875	0.00234081	7.97444e-14	CA,LDL-C,HDL-C,TG,APO-A1,APO-B
Serum-25(OH)D	rs1128535	3	T	C	0.500049	0.0164131	0.0020293	6.06457e-16	CA,LDL-C,HDL-C,TG,APO-A1,APO-B
Serum-25(OH)D	rs115288876	1	A	G	0.0433211	0.0788065	0.00498229	2.3632e-56	CA,LDL-C,HDL-C,TG,APO-A1,APO-B
Serum-25(OH)D	rs11542462	16	A	G	0.133511	-0.0247803	0.00298265	9.72076e-17	CA,LDL-C,HDL-C,TG,APO-A1,APO-B
Serum-25(OH)D	rs11600054	11	A	G	0.0100965	0.0681747	0.0101478	1.83992e-11	CA,LDL-C,HDL-C,TG,APO-A1,APO-B
Serum-25(OH)D	rs11726886	4	A	C	0.290783	-0.0536726	0.00225483	3.08319e-125	CA,LDL-C,HDL-C,TG,APO-A1,APO-B
Serum-25(OH)D	rs117300835	11	A	G	0.0133061	-0.334985	0.00886005	1e-200	CA,LDL-C,HDL-C,TG,APO-A1,APO-B
Serum-25(OH)D	rs11791258	9	A	G	0.191072	0.0140808	0.00258052	4.85434e-08	CA,LDL-C,HDL-C,TG,APO-A1,APO-B
Serum-25(OH)D	rs11867297	17	T	C	0.38533	0.0135432	0.00209454	1.00649e-10	CA,LDL-C,HDL-C,TG,APO-A1,APO-B
Serum-25(	rs12056768	8	G	T	0.584044	-0.0231961	0.00206406	2.65094e-29	CA,LDL-C,HDL-C,TG,AP

OH)D									O-A1,APO-B
Serum-25(OH)D	rs12283049	11	G	A	0.234433	-0.0564566	0.00240614	9.61612e-122	CA,LDL-C,HDL-C,TG,APO-A1,APO-B
Serum-25(OH)D	rs12324720	15	A	G	0.174629	-0.0149159	0.00267451	2.44625e-08	CA,LDL-C,HDL-C,TG,APO-A1,APO-B
Serum-25(OH)D	rs12462826	19	A	G	0.369566	-0.0132119	0.0021149	4.18312e-10	CA,LDL-C,HDL-C,TG,APO-A1,APO-B
Serum-25(OH)D	rs12501515	4	A	G	0.58966	-0.078957	0.00206932	1e-200	CA,LDL-C,HDL-C,TG,APO-A1,APO-B
Serum-25(OH)D	rs1260326	2	C	T	0.603925	0.0197194	0.00207421	1.96336e-21	CA,LDL-C,HDL-C,TG,APO-A1,APO-B
Serum-25(OH)D	rs12775091	10	T	C	0.213441	0.0155618	0.00247693	3.32767e-10	CA,LDL-C,HDL-C,TG,APO-A1,APO-B
Serum-25(OH)D	rs13076508	3	C	T	0.0535034	0.0250544	0.00451036	2.77856e-08	CA,LDL-C,HDL-C,TG,APO-A1,APO-B
Serum-25(OH)D	rs13108245	4	G	A	0.386601	-0.0122246	0.00208616	4.63234e-09	CA,LDL-C,HDL-C,TG,APO-A1,APO-B
Serum-25(OH)D	rs1321247	6	T	A	0.101578	-0.0221847	0.00336578	4.36214e-11	CA,LDL-C,HDL-C,TG,APO-A1,APO-B
Serum-25(OH)D	rs13294734	9	T	C	0.466128	0.012568	0.00205837	1.02277e-09	CA,LDL-C,HDL-C,TG,APO-A1,APO-B
Serum-25(OH)D	rs1343776	1	A	G	0.22133	0.0180762	0.00245028	1.61659e-13	CA,LDL-C,HDL-C,TG,APO-A1,APO-B
Serum-25(OH)D	rs138335	22	G	C	0.658587	-0.0137663	0.00215115	1.55887e-10	CA,LDL-C,HDL-C,TG,APO-A1,APO-B
Serum-25(	rs1384687	8	A	G	0.132271	-0.0168656	0.00299675	1.82331e-08	CA,LDL-C,HDL-C,TG,AP

OH)D									O-A1,APO-B
Serum-25(OH)D	rs142004400	14	C	A	0.0342066	-0.0310034	0.00559561	3.01328e-08	CA,LDL-C,HDL-C,TG,APO-A1,APO-B
Serum-25(OH)D	rs142158911	19	A	G	0.111826	0.026284	0.00323446	4.42792e-16	CA,LDL-C,HDL-C,TG,APO-A1,APO-B
Serum-25(OH)D	rs144965707	11	A	G	0.0618296	-0.0348143	0.00421754	1.52335e-16	CA,LDL-C,HDL-C,TG,APO-A1,APO-B
Serum-25(OH)D	rs1532085	15	G	A	0.616672	0.0252805	0.00208639	8.59805e-34	CA,LDL-C,HDL-C,TG,APO-A1,APO-B
Serum-25(OH)D	rs1627043	11	C	G	0.0332414	-0.0486441	0.00566106	8.4918e-18	CA,LDL-C,HDL-C,TG,APO-A1,APO-B
Serum-25(OH)D	rs1684600	16	T	C	0.298695	-0.0125301	0.00221719	1.59188e-08	CA,LDL-C,HDL-C,TG,APO-A1,APO-B
Serum-25(OH)D	rs17207784	6	C	T	0.324195	-0.0134939	0.0021713	5.14375e-10	CA,LDL-C,HDL-C,TG,APO-A1,APO-B
Serum-25(OH)D	rs17473257	11	A	G	0.0172466	-0.0611372	0.00780044	4.59092e-15	CA,LDL-C,HDL-C,TG,APO-A1,APO-B
Serum-25(OH)D	rs1800588	15	T	C	0.214964	-0.0305021	0.00246932	4.72607e-35	CA,LDL-C,HDL-C,TG,APO-A1,APO-B
Serum-25(OH)D	rs1858889	7	C	A	0.502532	0.0134514	0.00203065	3.4914e-11	CA,LDL-C,HDL-C,TG,APO-A1,APO-B
Serum-25(OH)D	rs1871395	12	G	A	0.152679	-0.0203733	0.00282689	5.72137e-13	CA,LDL-C,HDL-C,TG,APO-A1,APO-B
Serum-25(OH)D	rs1949633	3	C	T	0.605759	0.0114162	0.00208637	4.45492e-08	CA,LDL-C,HDL-C,TG,APO-A1,APO-B
Serum-25(	rs2037511	18	A	G	0.165924	0.0176624	0.00272732	9.41239e-11	CA,LDL-C,HDL-C,TG,AP

OH)D									O-A1,APO-B
Serum-25(OH)D	rs2074735	22	C	G	0.0648365	0.0292667	0.00412	1.21563e-12	CA,LDL-C,HDL-C,TG,APO-A1,APO-B
Serum-25(OH)D	rs2171427	12	A	G	0.156491	-0.0165489	0.00281738	4.25706e-09	CA,LDL-C,HDL-C,TG,APO-A1,APO-B
Serum-25(OH)D	rs2229742	21	C	G	0.104659	-0.0249837	0.00331409	4.75007e-14	CA,LDL-C,HDL-C,TG,APO-A1,APO-B
Serum-25(OH)D	rs2245133	6	C	T	0.164339	-0.0212917	0.00273999	7.80369e-15	CA,LDL-C,HDL-C,TG,APO-A1,APO-B
Serum-25(OH)D	rs2297991	10	C	T	0.718478	0.0127547	0.00225578	1.5656e-08	CA,LDL-C,HDL-C,TG,APO-A1,APO-B
Serum-25(OH)D	rs2494429	1	G	A	0.822999	-0.0148459	0.00267333	2.80292e-08	CA,LDL-C,HDL-C,TG,APO-A1,APO-B
Serum-25(OH)D	rs2595644	7	T	G	0.384889	-0.0122625	0.00209685	4.9737e-09	CA,LDL-C,HDL-C,TG,APO-A1,APO-B
Serum-25(OH)D	rs2710651	2	A	G	0.526101	-0.0115892	0.00203474	1.2288e-08	CA,LDL-C,HDL-C,TG,APO-A1,APO-B
Serum-25(OH)D	rs2756119	14	A	G	0.38143	0.0121434	0.0021104	8.71224e-09	CA,LDL-C,HDL-C,TG,APO-A1,APO-B
Serum-25(OH)D	rs2807834	1	G	T	0.68514	-0.0150625	0.00218678	5.65979e-12	CA,LDL-C,HDL-C,TG,APO-A1,APO-B
Serum-25(OH)D	rs28435470	12	A	G	0.663204	-0.0118696	0.00214831	3.29367e-08	CA,LDL-C,HDL-C,TG,APO-A1,APO-B
Serum-25(OH)D	rs2847500	11	A	G	0.123204	-0.022548	0.00308665	2.77268e-13	CA,LDL-C,HDL-C,TG,APO-A1,APO-B
Serum-25(	rs290400	20	A	G	0.665472	-0.0130967	0.00216338	1.41465e-09	CA,LDL-C,HDL-C,TG,AP

OH)D									O-A1,APO-B
Serum-25(OH)D	rs3114045	4	C	T	0.866137	-0.0221737	0.00298008	1.00207e-13	CA,LDL-C,HDL-C,TG,APO-A1,APO-B
Serum-25(OH)D	rs325393	15	T	G	0.278218	-0.0136497	0.00227676	2.03226e-09	CA,LDL-C,HDL-C,TG,APO-A1,APO-B
Serum-25(OH)D	rs34186890	3	G	A	0.259637	-0.0156853	0.00231839	1.3277e-11	CA,LDL-C,HDL-C,TG,APO-A1,APO-B
Serum-25(OH)D	rs34726834	8	T	C	0.252209	0.014013	0.00234858	2.42237e-09	CA,LDL-C,HDL-C,TG,APO-A1,APO-B
Serum-25(OH)D	rs35270497	2	T	C	0.176236	0.0156723	0.0026815	5.07715e-09	CA,LDL-C,HDL-C,TG,APO-A1,APO-B
Serum-25(OH)D	rs35823191	1	C	T	0.342049	-0.0232636	0.00214072	1.65272e-27	CA,LDL-C,HDL-C,TG,APO-A1,APO-B
Serum-25(OH)D	rs3732220	2	A	G	0.0852646	-0.0478406	0.00363266	1.31341e-39	CA,LDL-C,HDL-C,TG,APO-A1,APO-B
Serum-25(OH)D	rs3829251	11	A	G	0.133277	-0.114453	0.00298056	1e-200	CA,LDL-C,HDL-C,TG,APO-A1,APO-B
Serum-25(OH)D	rs4147536	4	C	A	0.788613	-0.0148038	0.00249007	2.76179e-09	CA,LDL-C,HDL-C,TG,APO-A1,APO-B
Serum-25(OH)D	rs4348160	4	G	T	0.326968	-0.0258401	0.00216263	6.61607e-33	CA,LDL-C,HDL-C,TG,APO-A1,APO-B
Serum-25(OH)D	rs4364259	4	A	G	0.198665	0.0172408	0.00256676	1.85524e-11	CA,LDL-C,HDL-C,TG,APO-A1,APO-B
Serum-25(OH)D	rs4420638	19	G	A	0.176831	-0.0192973	0.00265905	3.95094e-13	CA,LDL-C,HDL-C,TG,APO-A1,APO-B
Serum-25(	rs4580037	13	C	A	0.285578	-0.0135627	0.00225079	1.68306e-09	CA,LDL-C,HDL-C,TG,AP

OH)D									O-A1,APO-B
Serum-25(OH)D	rs512083	1	C	T	0.462488	0.0122172	0.00204286	2.22526e-09	CA,LDL-C,HDL-C,TG,APO-A1,APO-B
Serum-25(OH)D	rs5770794	22	T	C	0.3143	-0.0133141	0.00221143	1.738e-09	CA,LDL-C,HDL-C,TG,APO-A1,APO-B
Serum-25(OH)D	rs6129648	20	G	A	0.379837	0.0140631	0.00210628	2.44287e-11	CA,LDL-C,HDL-C,TG,APO-A1,APO-B
Serum-25(OH)D	rs61698755	17	C	T	0.560041	-0.011465	0.0020505	2.25372e-08	CA,LDL-C,HDL-C,TG,APO-A1,APO-B
Serum-25(OH)D	rs61747728	1	T	C	0.0385611	0.0303061	0.00526894	8.82755e-09	CA,LDL-C,HDL-C,TG,APO-A1,APO-B
Serum-25(OH)D	rs61813875	1	G	C	0.0248209	0.0821291	0.00658876	1.15824e-35	CA,LDL-C,HDL-C,TG,APO-A1,APO-B
Serum-25(OH)D	rs61887421	11	C	T	0.0300871	-0.036726	0.00597754	8.04674e-10	CA,LDL-C,HDL-C,TG,APO-A1,APO-B
Serum-25(OH)D	rs62007299	15	A	G	0.712958	-0.0124205	0.00224413	3.11853e-08	CA,LDL-C,HDL-C,TG,APO-A1,APO-B
Serum-25(OH)D	rs62129966	19	A	C	0.160854	0.0611636	0.00276373	1.59588e-108	CA,LDL-C,HDL-C,TG,APO-A1,APO-B
Serum-25(OH)D	rs635634	9	T	C	0.186573	-0.0150476	0.0026042	7.55092e-09	CA,LDL-C,HDL-C,TG,APO-A1,APO-B
Serum-25(OH)D	rs6438900	3	G	C	0.256034	0.0150486	0.00234031	1.27447e-10	CA,LDL-C,HDL-C,TG,APO-A1,APO-B
Serum-25(OH)D	rs6672758	1	T	C	0.80023	0.0162478	0.0025554	2.04094e-10	CA,LDL-C,HDL-C,TG,APO-A1,APO-B
Serum-25(	rs6834488	4	T	C	0.422833	-0.01445	0.00205911	2.25684e-12	CA,LDL-C,HDL-C,TG,AP

OH)D									O-A1,APO-B
Serum-25(OH)D	rs71599974	4	G	A	0.148048	0.0257378	0.00286182	2.39497e-19	CA,LDL-C,HDL-C,TG,APO-A1,APO-B
Serum-25(OH)D	rs727857	2	A	G	0.61181	-0.0120548	0.00209882	9.26659e-09	CA,LDL-C,HDL-C,TG,APO-A1,APO-B
Serum-25(OH)D	rs733454	11	T	C	0.0991663	0.0188545	0.00340001	2.93265e-08	CA,LDL-C,HDL-C,TG,APO-A1,APO-B
Serum-25(OH)D	rs73413596	12	C	T	0.0740317	0.0223468	0.00388928	9.15103e-09	CA,LDL-C,HDL-C,TG,APO-A1,APO-B
Serum-25(OH)D	rs742493	6	C	T	0.112871	0.0183528	0.00320607	1.03805e-08	CA,LDL-C,HDL-C,TG,APO-A1,APO-B
Serum-25(OH)D	rs7528419	1	G	A	0.22441	0.0215389	0.00243165	8.16582e-19	CA,LDL-C,HDL-C,TG,APO-A1,APO-B
Serum-25(OH)D	rs7569755	2	A	G	0.288618	0.0136395	0.00225626	1.49242e-09	CA,LDL-C,HDL-C,TG,APO-A1,APO-B
Serum-25(OH)D	rs7580771	2	T	G	0.175983	-0.0165625	0.0026652	5.15383e-10	CA,LDL-C,HDL-C,TG,APO-A1,APO-B
Serum-25(OH)D	rs7652808	3	G	T	0.649492	-0.0212858	0.00212614	1.35738e-23	CA,LDL-C,HDL-C,TG,APO-A1,APO-B
Serum-25(OH)D	rs7712001	5	G	T	0.440176	0.011939	0.00206215	7.05472e-09	CA,LDL-C,HDL-C,TG,APO-A1,APO-B
Serum-25(OH)D	rs77532868	10	T	C	0.0521528	0.0259557	0.00456301	1.28316e-08	CA,LDL-C,HDL-C,TG,APO-A1,APO-B
Serum-25(OH)D	rs7784802	7	T	A	0.358913	0.0133202	0.0021157	3.05675e-10	CA,LDL-C,HDL-C,TG,APO-A1,APO-B
Serum-25(	rs77924615	16	A	G	0.194374	-0.0152475	0.00259019	3.94176e-09	CA,LDL-C,HDL-C,TG,AP

OH)D									O-A1,APO-B
Serum-25(OH)D	rs77960347	18	G	A	0.0127013	-0.0525688	0.00905963	6.53221e-09	CA,LDL-C,HDL-C,TG,APO-A1,APO-B
Serum-25(OH)D	rs78649910	4	A	T	0.105728	-0.0191377	0.00332162	8.33355e-09	CA,LDL-C,HDL-C,TG,APO-A1,APO-B
Serum-25(OH)D	rs8018720	14	C	G	0.823508	-0.0344962	0.00266074	1.93508e-38	CA,LDL-C,HDL-C,TG,APO-A1,APO-B
Serum-25(OH)D	rs804281	8	G	A	0.583498	0.015895	0.00205997	1.19922e-14	CA,LDL-C,HDL-C,TG,APO-A1,APO-B
Serum-25(OH)D	rs8107974	19	T	A	0.0763013	0.0355672	0.00382316	1.36427e-20	CA,LDL-C,HDL-C,TG,APO-A1,APO-B
Serum-25(OH)D	rs8121940	20	G	C	0.19762	-0.0435612	0.00254898	1.77052e-65	CA,LDL-C,HDL-C,TG,APO-A1,APO-B
Serum-25(OH)D	rs9375037	6	C	A	0.443168	0.0117059	0.00205423	1.20895e-08	CA,LDL-C,HDL-C,TG,APO-A1,APO-B
Serum-25(OH)D	rs9409266	9	A	G	0.862222	-0.0167771	0.00294656	1.2426e-08	CA,LDL-C,HDL-C,TG,APO-A1,APO-B
Serum-25(OH)D	rs964184	11	C	G	0.867234	0.0406845	0.00298936	3.50429e-42	CA,LDL-C,HDL-C,TG,APO-A1,APO-B
Serum-25(OH)D	rs9847248	3	A	G	0.712969	-0.0123077	0.00224484	4.19035e-08	CA,LDL-C,HDL-C,TG,APO-A1,APO-B
Serum-25(OH)D	rs986649	5	G	A	0.321628	0.0128637	0.00217811	3.50728e-09	CA,LDL-C,HDL-C,TG,APO-A1,APO-B
Serum-25(OH)D	rs9946771	18	T	C	0.0663426	-0.0233992	0.00407655	9.47022e-09	CA,LDL-C,HDL-C,TG,APO-A1,APO-B

Table S4 Confounding factors and the SNPs associated with lipid traits and 25(OH)D

Confounders - ischemic cardiomyopathy

Exposure	CHR	BETA	SE	p-value	id	SNP	A1	A2	EAF	traits
25(OH)D	19	-0.1243	0.0183	1.0639e-11	finn-b-I9_ISCHHEART	rs142158911	A	G	0.1024	Ischemic heart diseases
25(OH)D	1	-0.0813	0.0135	1.844e-09	finn-b-I9_ISCHHEART	rs7528419	G	A	0.2147	Ischemic heart diseases
25(OH)D	19	0.0728	0.0126	6.72001e-09	finn-b-I9_ISCHHEART	rs4420638	G	A	0.2717	Ischemic heart diseases
HDL-C	12	-0.0632	0.0112	1.88101e-08	finn-b-I9_ISCHHEART	rs3184504	C	T	0.5912	Ischemic heart diseases
HDL-C	1	0.0822	0.0135	1.17101e-09	finn-b-I9_ISCHHEART	rs646776	T	C	0.7854	Ischemic heart diseases
HDL-C	20	0.0531	0.0111	1.67001e-06	finn-b-I9_ISCHHEART	rs6142206	A	G	0.4711	Ischemic heart diseases
LDL-C	6	0.4853	0.0534	9.60506e-20	finn-b-I9_ISCHHEART	rs117733303	G	A	0.0113	Ischemic heart diseases
LDL-C	1	-0.2103	0.0299	2.16721e-12	finn-b-I9_ISCHHEART	rs11591147	T	G	0.03609	Ischemic heart diseases
LDL-C	1	-0.0822	0.0135	1.19201e-09	finn-b-I9_ISCHHEART	rs12740374	T	G	0.2145	Ischemic heart diseases
LDL-C	6	160710851	NA	3.11903e-06	finn-b-I9_ISCHHEART	rs3127580	T	C	0.1243	Ischemic heart diseases

LDL-C	1	0.0566	0.0112	4.33701e-07	finn-b-I9_ISCHHEART	rs472495	T	G	0.5766	Ischemic heart diseases
LDL-C	19	-0.1261	0.0183	5.83042e-12	finn-b-I9_ISCHHEART	rs73015024	T	G	0.102	Ischemic heart diseases
ApoA1	12	0.0613	0.0114	6.77595e-08	finn-b-I9_ISCHHEART	rs11065987	G	A	0.3828	Ischemic heart diseases
ApoA1	1	-0.0822	0.0135	1.19201e-09	finn-b-I9_ISCHHEART	rs12740374	T	G	0.2145	Ischemic heart diseases
ApoA1	19	-0.1194	0.0231	2.27499e-07	finn-b-I9_ISCHHEART	rs1531517	A	G	0.06287	Ischemic heart diseases
ApoA1	6	0.0683	1.95398e-06	NA	finn-b-I9_ISCHHEART	rs2395045	T	C	0.8121	Ischemic heart diseases
ApoA1	19	0.0707	0.0146	1.181e-06	finn-b-I9_ISCHHEART	rs429358	C	T	0.1826	Ischemic heart diseases
ApoB	6	0.3846	0.0526	2.73212e-13	finn-b-I9_ISCHHEART	rs9295128	T	G	0.01146	Ischemic heart diseases
ApoB	1	-0.2103	0.0299	2.16721e-12	finn-b-I9_ISCHHEART	rs11591147	T	G	0.03609	Ischemic heart diseases
ApoB	19	-0.1261	0.0183	5.87895e-12	finn-b-I9_ISCHHEART	rs12151108	A	G	0.102	Ischemic heart diseases
ApoB	1	-0.0822	0.0135	1.19201e-09	finn-b-I9_ISCHHEART	rs12740374	T	G	0.2145	Ischemic heart diseases
ApoB	1	0.0583	0.0116	4.51305e-07	finn-b-I9_ISCHHEART	rs553741	C	G	0.639	Ischemic heart diseases
ApoB	11	-0.0948	0.0156	1.199e-09	finn-b-I9_ISCHHEART	rs964184	C	G	0.8542	Ischemic heart diseases

Confounders - schizophrenia-hypertrophic cardiomyopathy

None

Confounders - schizophrenia-hypertrophic cardiomyopathy-arrhythmias

None

Confounders - schizophrenia

25(OH)D	16	0.0437011	0.0081	7.46105e-08	ieu-b-5099	rs1684600	T	C	0.318	Schizophrenia
HDL-C	4	0.151897	0.0215	1.53603e-12	ieu-b-42	rs13107325	T	C	0.078	schizophrenia
HDL-C	6	-0.047103	0.0094	5.94593e-07	ieu-b-5099	rs4947121	C	T	0.799	Schizophrenia
HDL-C	12	0.242	0.0512	2.29599e-06	finn-b-F5_SCHZPHR	rs4016338	C	T	0.1008	Schizophrenia
HDL-C	3	0.0380965	0.0081	2.39999e-06	ieu-b-5100	rs6765484	T	C	0.553	Schizophrenia
HDL-C	16	-0.0572967	0.0077	8.92483e-14	ieu-b-5099	rs3814883	T	C	0.451	Schizophrenia
HDL-C	8	-0.0832008	0.0188	9.22104e-06	ieu-a-22	rs9987289	G	A	NA	Schizophrenia
HDL-C	3	0.0380965	0.0077	8.39499e-07	ieu-b-5099	rs6765484	T	C	0.555	Schizophrenia
HDL-C	14	-0.0384031	0.008	1.692e-06	ieu-b-5100	rs7149672	G	A	0.621	Schizophrenia

HDL-C	5	-0.038201	0.0084	4.783e-06	ieu-b-5100	rs6877776	G	A	0.674	Schizophrenia
LDL-C	12	0.0411971	0.0081	3.92799e-07	ieu-b-5099	rs11057397	T	C	0.302	Schizophrenia
LDL-C	1	0.051904	0.0107	1.29799e-06	ieu-b-5102	rs10782922	A	G	0.793	Schizophrenia
LDL-C	4	0.156701	0.0165	1.91911e-21	ieu-b-5099	rs13107325	T	C	0.064	Schizophrenia
LDL-C	14	0.0557982	0.0082	9.33254e-12	ieu-b-5100	rs12891477	T	C	0.357	Schizophrenia
LDL-C	3	136121600	175799	5.14399e-06	ieu-b-5099	rs28478252	C	A	0.777	Schizophrenia
ApoB	2	0.011	0.0024	7.10003e-06	ebi-a-GCST90016621	rs4665972	C	T	NA	Schizophrenia vs bipolar disorder (ordinary least squares (OLS))
TG	6	0.0666955	0.0101	4.36616e-11	ieu-b-5102	rs2247056	C	T	0.738	Schizophrenia
TG	12	0.0501992	0.009	2.81099e-08	ieu-b-5102	rs11057408	T	G	0.338	Schizophrenia
TG	3	-0.0495994	0.0091	5.09003e-08	ieu-b-5099	rs645040	T	G	0.794	Schizophrenia
ApoA1	3	-0.0600974	0.0129	3.17001e-06	ieu-b-5099	rs11717619	C	A	0.092	Schizophrenia
ApoA1	4	0.1567	0.0165	1.91911e	ieu-b-5099	rs13107325	T	C	0.064	Schizophrenia

		01		-21						
ApoA1	15	-0.0708964	0.0094	5.82908e-14	ieu-b-5102	rs2071410	G	C	0.328	Schizophrenia
ApoA1	12	0.104805	0.0207	4.05901e-07	ieu-b-5101	rs2270788	C	T	0.216	Schizophrenia
ApoA1	3	-0.0495994	0.0091	5.09003e-08	ieu-b-5099	rs645040	T	G	0.794	Schizophrenia
ApoA1	12	0.0444954	0.0092	1.21801e-06	ieu-b-5102	rs7133378	A	G	0.326	Schizophrenia
ApoA1	5	-0.0374018	0.008	3.11997e-06	ieu-b-5099	rs6866471	A	G	0.676	Schizophrenia
ApoA1	12	0.036498	0.0082	7.69095e-06	ieu-b-5099	rs7133378	A	G	0.299	Schizophrenia
**Confounders - alcohol consumption**										
25(OH)D	2	0.02839	0.002973	1.34091e-21	ieu-a-1283	rs1260326	C	T	0.6066	Alcohol consumption
25(OH)D	4	0.02036	0.002976	7.98362e-12	ieu-a-1283	rs3114045	C	T	0.8692	Alcohol consumption
25(OH)D	3	-0.01477	0.002973	6.80205e-07	ieu-a-1283	rs7652808	G	T	0.6496	Alcohol consumption
ApoA1	2	0.02839	0.002973	1.34091e-21	ieu-a-1283	rs1260326	C	T	0.6066	Alcohol consumption
ApoA1	4	0.3878	0.0834	3.35097e-06	ieu-b-4834	rs1229984	C	T	NA	Alcohol consumption
ApoB	2	0.0270	0.003023	3.408e-1	ieu-a-1283	rs4665972	C	T	0.605	Alcohol

		8		9						consumption
TG	2	0.02839	0.002973	1.34091e-21	ieu-a-1283	rs1260326	C	T	0.6066	Alcohol consumption
LDL-C	4	0.3878	0.0834	3.35097e-06	ieu-b-4834	rs1229984	C	T	NA	Alcohol consumption
LDL-C	2	0.02112	0.00467995	6.4e-06	ukb-b-5359	rs1260326	C	T	0.603772	Alcohol
**Confounders - smoking status**										
25(OH)D	3	0.00631613	0.00140709	9.09997e-06	ebi-a-GCST90029014	rs1949633	C	T	0.605387	Smoking status
25(OH)D	3	-0.0122094	0.0014362	3.50026e-17	ebi-a-GCST90029014	rs7652808	G	T	0.64902	Smoking status
HDL-C	11	0.0196374	0.00438013	7.45006e-06	ieu-b-4877	rs17309930	A	C	0.185	smoking initiation
HDL-C	10	-0.00559773	0.00113835	8.79995e-07	ukb-b-223	rs7924036	T	G	0.503294	Current tobacco smoking
HDL-C	18	-0.0155055	0.00329051	2.45143e-06	ukb-a-16	rs59360013	T	C	0.0421057	Current tobacco smoking
HDL-C	6	0.0210198	0.00426409	8.16e-07	ieu-b-4877	rs4947121	C	T	0.766	smoking initiation
	19	-0.0622556	0.00647302	6.75927e-22	ieu-b-142	rs12985907	A	G	0.248	Cigarettes smoked per day
	14	0.00712783	0.00143307	9.09997e-07	ebi-a-GCST90029014	rs12891477	T	C	0.368936	Smoking status
	8	-0.012	0.002703	5.1e-06	ukb-b-2134	rs11997161	C	T	0.51751	Past tobacco

		3243	03						4	smoking
LDL-C	21	0.0137696	0.00272962	4.49997e-07	ebi-a-GCST009971	rs4860987	T	A	0.271013	Nicotine metabolite ratio in current smokers
LDL-C	18	21113285	0.00347698	3.59998e-06	ukb-b-10831	rs2510344	C	T	0.49054	Pack years of smoking
LDL-C	3	136121600	0.00706864	3.76999e-06	ieu-b-142	rs28478252	C	A	0.754	Cigarettes smoked per day
ApoA1	4	-0.153955	0.03135	9.22996e-07	ebi-a-GCST90029014	rs9901671	C	T	0.601457	Smoking status
ApoA1	15	-0.00677667	0.00147221	3.89996e-06	ebi-a-GCST90029014	rs2071410	G	C	0.325327	Smoking status
ApoA1	18	-0.0167895	0.00342372	1.09999e-06	ebi-a-GCST90029014	rs77169818	T	A	0.042004	Smoking status
ApoA1	17	0.00730894	0.00140345	2.39999e-07	ebi-a-GCST90029014	rs9901671	C	T	0.601457	Smoking status
**Confounders -anxiety**										
25(OH)D	3	0.0101191	0.00126335	1.15345e-15	ukb-a-51	rs7652808	G	T	0.649997	Worrier / anxious feelings
25(OH)D	10	0.0113967	0.00227439	5.39995e-07	ukb-b-6519	rs77532868	T	C	0.05367	Worrier / anxious feelings
HDL-C	18	-0.00791906	0.001362	6.09523e-09	ukb-a-51	rs11664369	T	C	0.26831	Worrier / anxious feelings
HDL-C	11	-0.006	0.001268	1.40001e	ukb-b-6519	rs17309930	A	C	0.20545	Worrier /

		11679	5	-06					7	anxious feelings
HDL-C	5	0.0064 3887	0.000982 765	5.70033e -11	ukb-b-6991	rs254024	T	G	0.43787 8	Seen doctor (GP) for nerves, anxiety, tension or depression
HDL-C	12	0.0054 9363	0.001046 6	1.5e-07	ukb-b-6519	rs2111216	G	A	0.59365 4	Worrier / anxious feelings
HDL-C	14	0.0061 546	0.000978 018	3.09999e -10	ukb-b-6991	rs7144654	G	A	0.54010 1	Seen doctor (GP) for nerves, anxiety, tension or depression
ApoA1	11	3.2999 7e-07	0.001547 89		ukb-b-6519	rs11237488	T	C	0.12813 1	Worrier / anxious feelings
ApoA1	16	31056 433	450765		ukb-b-6519	rs35468353	G	A	0.37678 5	Worrier / anxious feelings
ApoA1	6	0.0057 7887	0.001262 36	5.89997e -08	ukb-b-6519	rs4935356	A	T	0.24384 4	Worrier / anxious feelings
ApoA1	6	-0.006 43708	0.001194 65	7.10003e -08	ukb-b-6519	rs78677597	C	A	0.24434 5	Worrier / anxious feelings
ApoB	6	0.0057 7887	0.001262 36	4.70002e -06	ukb-b-6519	rs4935356	A	T	0.24384 4	Worrier / anxious feelings
**Confounders - obesity**										
HDL-C	18	0.1007	0.0208	1.23001e -06	finn-b-E4_OB ESITY	rs11664369	T	C	0.1998	Obesity
HDL-C	2	0.0763	0.0168	5.64495e -06	finn-b-E4_OB ESITY	rs12986742	C	T	0.4177	Obesity

ApoA1	6	0.17	9.60064e-12	0.025	ieu-a-91	rs4715210	T	C	0.074	Obesity
**Confounders - asthma**										
	17	-0.1137	0.0195	5.25702e-09	finn-b-ASTHMA_ACUTE_RESPIRATORY_INFECTIONS	rs2643195	G	A	0.706	Asthma-related acute respiratory infections
**Confounders - abdominal aortic aneurysm**										
HDL-C	11	-0.503407	0.0117896	3.2734099999998e-310	eqtl-a-ENSG00000025434	rs1052373	T	C	0.344118	ENSG00000025434*
HDL-C	12	-0.057242	0.0119361	1.62032e-06	eqtl-a-ENSG00000025434	rs3184504	C	T	0.542056	ENSG00000025434
ApoA1	12	0.0663553	0.0122449	5.99998e-08	eqtl-a-ENSG00000025434	rs11065987	G	A	0.380678	ENSG00000025434
TG	11	-0.512481	0.0118282	3.2734099999998e-310	eqtl-a-ENSG00000025434	rs10501321	C	T	0.337123	ENSG00000025434
ApoA1	11	-0.52799	3.27340999999998e-310	0.0118163	eqtl-a-ENSG00000025434	rs901746	G	A	0.333459	ENSG00000025434
**Confounders - bipolar disorder and major depressive disorder**										
HDL-C	6	0.013	0.0027	9.09997e-07	ebi-a-GCST90016607	rs968050	T	C	NA	Bipolar disorder vs major

										depressive disorder (ordinary least squares (OLS))
ApoA1	2	-0.012	7.00003e-06	0.0027	ebi-a-GCST90016607	rs60960031	A	G	NA	Bipolar disorder vs major depressive disorder (ordinary least squares (OLS))
ApoB	2	-0.013	0.0027	2.80001e-06	ebi-a-GCST90016607	rs4665972	C	T	NA	Bipolar disorder vs major depressive disorder (ordinary least squares (OLS))

*LXRα Promotes Abdominal Aortic Aneurysm Formation Through UHRF1 Epigenetic Modification of miR-26b-3p. Circulation. 2024 Jul 2;150(1):30-46. doi: 10.1161/CIRCULATIONAHA.123.065202.

Table S5 Cochran Q test result and MR-Egger intercept in univariable MR

**Outcome**	**Exposure**	**Q test**		**MR-Egger**		
		Q-statistic	Q_p	Intercept	SE	P
Cardiac arrest	HDL-C	246.069476969209	'0.868722830464989	-0.005553505	0.00340529179748145	0.104080249141281
	LDL-C	361.722101573803	0.00263517954340075	-0.001660814	0.00332122616387529	0.617411798414442
	TG	47.3055840687793	0.419073769843244	-0.008405885	0.00792723457848745	0.29450758529731
	ApoA1	262.237754892918	0.284913669514364	-0.006222755	0.0031979513170311	0.052792593327759

ApoB	52.0579004067664	'0.137462423891581	0.00797280999840704	0.00920101467855704	0.391131656917186
Serum-25(OH)D	113.775251659834	0.357994410112086	-0.006872003	0.00554966926227284	0.218514097649137

Table S6 Sex-Related SNPs Among lipid traits and 25(OH)D

Sex hormone binding globulin (SHBG)

Exposure	CHR	BETA	SE	p-value	id	SNP	A1	A2	EAF	trait
HDL-C	10	-0.0422	0.0046	2.99985e-20	ieu-b-4870	rs1564406558	G	A	0.131148	Sex hormone binding globulin (SHBG)
HDL-C	2	0.0256	0.0032	5.90065e-16	ieu-b-4870	rs1047891	A	C	0.315465	Sex hormone binding globulin (SHBG)
HDL-C	11	-0.0283	0.0039	2.49977e-13	ieu-b-4870	rs11021232	C	T	0.17993	Sex hormone binding globulin (SHBG)
HDL-C	5	-0.0246	0.0034	6.89922e-13	ieu-b-4870	rs11738093	G	A	0.25159	Sex hormone binding globulin (SHBG)
HDL-C	16	0.0228	0.0035	6.09958e-11	ieu-b-4871	rs12928099	A	C	0.29544	Sex hormone binding globulin (SHBG)
HDL-C	5	-0.0207	0.0037	1.7e-08	ieu-b-4871	rs11738093	G	A	0.251179	Sex hormone binding globulin (SHBG)
HDL-C	6	-0.0375	0.0071	1.5e-07	ieu-b-4870	rs115447786	T	C	0.044919	Sex hormone binding globulin (SHBG)
HDL-C	12	-0.0214	0.0041	1.7e-07	ieu-b-4870	rs10876447	A	G	0.154008	Sex hormone binding globulin (SHBG)
HDL-C	4	-0.0153	0.0030	2.30001e-07	ieu-b-4870	rs10012624	A	C	0.463229	Sex hormone binding globulin (SHBG)
HDL-C	18	-0.0167	0.0034	5.80003e-07	ieu-b-4870	rs11664369	T	C	0.266867	Sex hormone binding globulin (SHBG)
HDL-C	14	-0.0149	0.0030	6.4e-07	ieu-b-4870	rs12895424	T	G	0.56801	Sex hormone binding globulin (SHBG)
HDL-C	11	-0.0193	0.0041	3.09999e-06	ieu-b-4871	rs11021232	C	T	0.182273	Sex hormone binding globulin (SHBG)
HDL-C	16	-0.0220	0.0047	3.2e-06	ieu-b-4871	rs12921195	A	C	0.139992	Sex hormone binding globulin (SHBG)
HDL-C	10	0.0207	0.0045	3.79997e-06	ieu-b-4870	rs10786114	T	C	0.875159	Sex hormone binding globulin (SHBG)
HDL-C	2	0.0326	0.0030	3.10027e-27	ieu-b-4870	rs13389219	T	C	0.392214	Sex hormone binding globulin (SHBG)

HDL-C	15	-0.0826	0.0093	5.60015e-19	ieu-b-4870	rs150844304	C	A	0.026004	Sex hormone binding globulin (SHBG)
HDL-C	11	-0.0269	0.0031	4.60045e-18	ieu-b-4870	rs174566	G	A	0.34952	Sex hormone binding globulin (SHBG)
HDL-C	2	0.0280	0.0032	5.50047e-18	ieu-b-4871	rs13389219	T	C	0.393151	Sex hormone binding globulin (SHBG)
HDL-C	5	-0.0228	0.0033	8.60003e-12	ieu-b-4870	rs13179413	T	C	0.283791	Sex hormone binding globulin (SHBG)
HDL-C	22	0.0245	0.0038	7.39946e-11	ieu-b-4870	rs140489	A	G	0.192703	Sex hormone binding globulin (SHBG)
HDL-C	14	0.0201	0.0034	2.1e-09	ieu-b-4870	rs13379043	C	T	0.279423	Sex hormone binding globulin (SHBG)
HDL-C	2	-0.0338	0.0060	2.1e-08	ieu-b-4870	rs17041868	C	T	0.063971	Sex hormone binding globulin (SHBG)
HDL-C	1	-0.0195	0.0036	4.09996e-08	ieu-b-4870	rs198325	T	C	0.219688	Sex hormone binding globulin (SHBG)
HDL-C	4	0.0161	0.0031	1.7e-07	ieu-b-4870	rs1464456	A	G	0.368277	Sex hormone binding globulin (SHBG)
HDL-C	5	-0.0185	0.0036	2.19999e-07	ieu-b-4871	rs13179413	T	C	0.285914	Sex hormone binding globulin (SHBG)
HDL-C	11	-0.0167	0.0033	5.1e-07	ieu-b-4871	rs174566	G	A	0.35081	Sex hormone binding globulin (SHBG)
HDL-C	5	0.0183	0.0039	2.19999e-06	ieu-b-4870	rs13360957	A	G	0.820363	Sex hormone binding globulin (SHBG)
HDL-C	7	0.0134	0.0030	6.4e-06	ieu-b-4870	rs1534696	A	C	0.540358	Sex hormone binding globulin (SHBG)
HDL-C	22	0.0675	0.0038	5.50047e-72	ieu-b-4871	rs2294915	T	C	0.2319	Sex hormone binding globulin (SHBG)
HDL-C	9	0.0478	0.0036	5.10035e-40	ieu-b-4870	rs296886	G	A	0.213992	Sex hormone binding globulin (SHBG)
HDL-C	1	0.0355	0.0035	8.30042e-25	ieu-b-4871	rs2642438	G	A	0.701824	Sex hormone binding globulin (SHBG)
HDL-C	22	0.0338	0.0035	6.4998e-22	ieu-b-4870	rs2294915	T	C	0.231508	Sex hormone binding globulin (SHBG)
HDL-C	2	-0.0311	0.0033	6.4998e-21	ieu-b-4871	rs2943645	T	C	0.647089	Sex hormone binding globulin (SHBG)
HDL-C	2	-0.0278	0.0031	1.80011e-19	ieu-b-4870	rs2943645	T	C	0.64627	Sex hormone binding globulin (SHBG)
HDL-C	10	0.0237	0.0030	1.29987e-15	ieu-b-4870	rs2068888	A	G	0.4516	Sex hormone binding globulin (SHBG)
HDL-C	16	0.0254	0.0032	4.00037e-15	ieu-b-4870	rs2925979	C	T	0.70015	Sex hormone binding globulin (SHBG)
HDL-C	14	-0.0243	0.0033	1.10002e-13	ieu-b-4871	rs766668229	T	C	0.616769	Sex hormone binding globulin (SHBG)
HDL-C	5	0.0212	0.0030	2.49977e-12	ieu-b-4870	rs2307111	C	T	0.394964	Sex hormone binding globulin (SHBG)
HDL-C	3	0.0248	0.0036	6.20012e-12	ieu-b-4870	rs2067819	A	G	0.213711	Sex hormone binding globulin (SHBG)
HDL-C	10	-0.0232	0.0035	5.79963e-11	ieu-b-4871	rs2792751	C	T	0.725652	Sex hormone binding globulin (SHBG)
HDL-C	9	-0.0230	0.0035	7.39946e-11	ieu-b-4871	rs2417125	G	A	0.283508	Sex hormone binding globulin (SHBG)

HDL-C	5	-0.0225	0.0035	1.6e-10	ieu-b-4870	rs2963468	G	A	0.234691	Sex hormone binding globulin (SHBG)
HDL-C	3	0.0245	0.0039	2.30001e-10	ieu-b-4871	rs2067819	A	G	0.214266	Sex hormone binding globulin (SHBG)
HDL-C	15	0.0205	0.0032	2.39999e-10	ieu-b-4870	rs2058914	A	G	0.703146	Sex hormone binding globulin (SHBG)
HDL-C	8	-0.0348	0.0056	4.20001e-10	ieu-b-4870	rs28446899	T	C	0.076977	Sex hormone binding globulin (SHBG)
HDL-C	9	-0.0170	0.0033	2.30001e-07	ieu-b-4870	rs2417125	G	A	0.284405	Sex hormone binding globulin (SHBG)
HDL-C	2	0.0160	0.0033	8.9e-07	ieu-b-4871	rs2723065	G	A	0.377012	Sex hormone binding globulin (SHBG)
HDL-C	1	0.0157	0.0032	1.2e-06	ieu-b-4870	rs2642438	G	A	0.703589	Sex hormone binding globulin (SHBG)
HDL-C	10	0.0154	0.0032	1.2e-06	ieu-b-4871	rs2068888	A	G	0.449215	Sex hormone binding globulin (SHBG)
HDL-C	15	0.0168	0.0035	1.40001e-06	ieu-b-4871	rs2058914	A	G	0.705193	Sex hormone binding globulin (SHBG)
HDL-C	5	-0.0180	0.0038	1.7e-06	ieu-b-4871	rs2963468	G	A	0.235901	Sex hormone binding globulin (SHBG)
HDL-C	11	0.0167	0.0037	5.1e-06	ieu-b-4870	rs1327872995	T	G	0.206607	Sex hormone binding globulin (SHBG)
HDL-C	10	-0.0147	0.0033	8.09991e-06	ieu-b-4870	rs2792751	C	T	0.724614	Sex hormone binding globulin (SHBG)
HDL-C	17	0.1423	0.0036	1.39991e-33	ieu-b-4870	rs3803800	G	A	0.788445	Sex hormone binding globulin (SHBG)
HDL-C	19	-0.0361	0.0030	2.80027e-28	ieu-b-4870	rs4804413	T	C	0.426882	Sex hormone binding globulin (SHBG)
HDL-C	5	-0.0389	0.0035	1.69981e-26	ieu-b-4870	rs40270	C	A	0.772585	Sex hormone binding globulin (SHBG)
HDL-C	4	0.0357	0.0033	6.79986e-22	ieu-b-4871	rs35473170	A	G	0.654631	Sex hormone binding globulin (SHBG)
HDL-C	1	-0.0384	0.0040	1.9002e-20	ieu-b-4871	rs3768321	T	G	0.196746	Sex hormone binding globulin (SHBG)
HDL-C	19	-0.0297	0.0032	1.59993e-18	ieu-b-4871	rs4804413	T	C	0.428205	Sex hormone binding globulin (SHBG)
HDL-C	5	-0.0332	0.0038	6.29941e-17	ieu-b-4871	rs40270	C	A	0.771541	Sex hormone binding globulin (SHBG)
HDL-C	16	-0.0271	0.0032	3.40017e-13	ieu-b-4871	rs4843780	T	C	0.416933	Sex hormone binding globulin (SHBG)
HDL-C	16	-0.0219	0.0030	1.29987e-12	ieu-b-4870	rs4843780	T	C	0.414248	Sex hormone binding globulin (SHBG)
HDL-C	1	-0.0264	0.0037	5.60015e-12	ieu-b-4870	rs3768321	T	G	0.196599	Sex hormone binding globulin (SHBG)
HDL-C	19	0.0282	0.0041	6.1e-09	ieu-b-4870	rs429358	C	T	0.154275	Sex hormone binding globulin (SHBG)
HDL-C	4	-0.0172	0.0030	5.69994e-08	ieu-b-4870	rs3912391	A	G	0.506942	Sex hormone binding globulin (SHBG)
HDL-C	11	0.0164	0.0030	5.69994e-08	ieu-b-4870	rs4930352	T	G	0.4941	Sex hormone binding globulin (SHBG)
HDL-C	9	-0.0195	0.0036	2.1e-07	ieu-b-4871	rs467303	T	C	0.266306	Sex hormone binding globulin (SHBG)

HDL-C	1	-0.0155	0.0030	2.19999e-07	ieu-b-4870	rs4323719	T	C	0.56416	Sex hormone binding globulin (SHBG)
HDL-C	19	0.0162	0.0031	2.30001e-07	ieu-b-4870	rs4805881	C	A	0.665721	Sex hormone binding globulin (SHBG)
HDL-C	13	0.0263	0.0051	6.80002e-07	ieu-b-4870	rs558003	A	G	0.093629	Sex hormone binding globulin (SHBG)
HDL-C	19	0.0167	0.0034	4.30002e-06	ieu-b-4871	rs4805881	C	A	0.664179	Sex hormone binding globulin (SHBG)
HDL-C	12	-0.0181	0.0039	NA	ieu-b-4870	rs56848735	T	C	0.172569	Sex hormone binding globulin (SHBG)
HDL-C	3	0.0200	0.0030	1.50003e-11	ieu-b-4870	rs6765484	T	C	0.474099	Sex hormone binding globulin (SHBG)
HDL-C	10	0.0345	0.0052	4.40048e-11	ieu-b-4870	rs1168111585	T	A	0.911941	Sex hormone binding globulin (SHBG)
HDL-C	20	-0.0196	0.0032	1.2e-09	ieu-b-4871	rs6142206	A	G	0.419774	Sex hormone binding globulin (SHBG)
HDL-C	6	0.0180	0.0031	3.89996e-09	ieu-b-4870	rs635769	C	T	0.628507	Sex hormone binding globulin (SHBG)
HDL-C	20	-0.0177	0.0030	4e-09	ieu-b-4870	rs6142206	A	G	0.42148	Sex hormone binding globulin (SHBG)
HDL-C	20	-0.0173	0.0031	3.59998e-08	ieu-b-4870	rs35733523	T	G	0.659844	Sex hormone binding globulin (SHBG)
HDL-C	6	0.0171	0.0032	1.09999e-07	ieu-b-4871	rs6934962	T	C	0.400747	Sex hormone binding globulin (SHBG)
HDL-C	3	0.0161	0.0032	4.09996e-07	ieu-b-4871	rs6765484	T	C	0.471281	Sex hormone binding globulin (SHBG)
HDL-C	5	-0.0165	0.0033	5e-07	ieu-b-4871	rs6877776	G	A	0.6313	Sex hormone binding globulin (SHBG)
HDL-C	2	-0.0183	0.0039	2.30001e-06	ieu-b-4870	rs1341629290	T	C	0.819564	Sex hormone binding globulin (SHBG)
HDL-C	20	-0.0158	0.0034	2.69998e-06	ieu-b-4871	rs35733523	T	G	0.657745	Sex hormone binding globulin (SHBG)
HDL-C	10	0.0140	0.0030	2.99999e-06	ieu-b-4870	rs703966	A	G	0.419079	Sex hormone binding globulin (SHBG)
HDL-C	15	-0.0147	0.0032	4.20001e-06	ieu-b-4870	rs200780188	G	A	0.670655	Sex hormone binding globulin (SHBG)
HDL-C	17	-0.0270	0.0060	7.19996e-06	ieu-b-4870	rs58729290	A	G	0.063584	Sex hormone binding globulin (SHBG)
HDL-C	10	0.0032	0.1494	0.0001	ieu-b-4871	rs7924036	T	G	0.502796	Sex hormone binding globulin (SHBG)
HDL-C	10	0.0030	0.0895	3.49945e-201	ieu-b-4870	rs7924036	T	G	0.503784	Sex hormone binding globulin (SHBG)
HDL-C	12	0.0037	0.0431	3.29989e-31	ieu-b-4871	rs538979053	C	A	0.239789	Sex hormone binding globulin (SHBG)
HDL-C	8	0.0055	0.0540	1.50003e-22	ieu-b-4871	rs9987289	G	A	0.909157	Sex hormone binding globulin (SHBG)
HDL-C	4	0.0032	0.0280	2.80027e-18	ieu-b-4871	rs7665587	C	T	0.422628	Sex hormone binding globulin (SHBG)
HDL-C	12	0.0034	0.0292	2.39994e-17	ieu-b-4870	rs538979053	C	A	0.240754	Sex hormone binding globulin (SHBG)
HDL-C	8	0.0051	0.0409	1.29987e-15	ieu-b-4870	rs9987289	G	A	0.9084	Sex hormone binding globulin (SHBG)

HDL-C	6	0.0030	-0.0211	1.10002e-12	ieu-b-4870	rs998584	A	C	0.483837	Sex hormone binding globulin (SHBG)
HDL-C	6	0.0038	0.0254	1.69981e-11	ieu-b-4870	rs75479205	G	A	0.189884	Sex hormone binding globulin (SHBG)
HDL-C	2	0.0076	-0.0483	2e-10	ieu-b-4870	rs78058190	A	G	0.05061	Sex hormone binding globulin (SHBG)
HDL-C	2	0.0032	-0.0174	5.19996e-08	ieu-b-4871	rs907866	A	G	0.444199	Sex hormone binding globulin (SHBG)
HDL-C	19	0.0030	0.0162	5.69994e-08	ieu-b-4870	rs8107967	G	A	0.565127	Sex hormone binding globulin (SHBG)
HDL-C	6	0.0032	0.0150	2.39999e-06	ieu-b-4871	rs968050	T	C	0.482166	Sex hormone binding globulin (SHBG)
HDL-C	6	0.0041	0.0185	5.39995e-06	ieu-b-4871	rs75479205	G	A	0.189003	Sex hormone binding globulin (SHBG)
LDL-C	1	-0.2160	0.0106	1.69981e-91	ieu-b-4871	rs114165349	C	G	0.022866	Sex hormone binding globulin (SHBG)
LDL-C	7	0.0739	0.0038	1.50003e-82	ieu-b-4870	rs10953260	C	T	0.81612	Sex hormone binding globulin (SHBG)
LDL-C	7	0.0764	0.0041	1.39991e-77	ieu-b-4871	rs10953260	C	T	0.81544	Sex hormone binding globulin (SHBG)
LDL-C	1	-0.1465	0.0098	4.10015e-50	ieu-b-4870	rs114165349	C	G	0.023317	Sex hormone binding globulin (SHBG)
LDL-C	17	0.0408	0.0029	5.50047e-44	ieu-b-4870	rs10445374	C	T	0.515095	Sex hormone binding globulin (SHBG)
LDL-C	2	0.0330	0.0030	4.00037e-28	ieu-b-4870	rs10184004	T	C	0.405686	Sex hormone binding globulin (SHBG)
LDL-C	17	0.0293	0.0031	9.20026e-21	ieu-b-4871	rs10445374	C	T	0.514725	Sex hormone binding globulin (SHBG)
LDL-C	2	0.0263	0.0032	2.90001e-16	ieu-b-4871	rs10184004	T	C	0.406541	Sex hormone binding globulin (SHBG)
LDL-C	1	0.0283	0.0039	5.19996e-13	ieu-b-4871	rs10782922	A	G	0.795457	Sex hormone binding globulin (SHBG)
LDL-C	5	0.0270	0.0038	9.8992e-13	ieu-b-4870	rs11429307	G	GT	0.809667	Sex hormone binding globulin (SHBG)
LDL-C	12	0.0205	0.0031	5.70033e-11	ieu-b-4870	rs11057397	T	C	0.335951	Sex hormone binding globulin (SHBG)
LDL-C	5	0.0232	0.0040	9.09997e-09	ieu-b-4871	rs11429307	G	GT	0.808113	Sex hormone binding globulin (SHBG)
LDL-C	14	0.0194	0.0035	2e-08	ieu-b-4870	rs10145740	T	C	0.243804	Sex hormone binding globulin (SHBG)
LDL-C	1	0.0193	0.0037	1.29999e-07	ieu-b-4870	rs10782922	A	G	0.7955	Sex hormone binding globulin (SHBG)
LDL-C	11	-0.0292	0.0063	4.09996e-06	ieu-b-4870	rs11231711	A	G	0.057978	Sex hormone binding globulin (SHBG)
LDL-C	19	0.0265	0.0058	5.69994e-06	ieu-b-4871	rs1065853	T	G	0.080505	Sex hormone binding globulin (SHBG)
LDL-C	19	0.0406	0.0090	6.59994e-06	ieu-b-4870	rs1081105	C	A	0.027886	Sex hormone binding globulin (SHBG)

LDL-C	8	0.0137	0.0031	9.09997e-06	ieu-b-4870	rs1134027	A	G	0.370559	Sex hormone binding globulin (SHBG)
LDL-C	2	0.0913	0.0032	9.7051e-176	ieu-b-4871	rs1260326	C	T	0.603649	Sex hormone binding globulin (SHBG)
LDL-C	2	0.0638	0.0030	3.10027e-99	ieu-b-4870	rs1260326	C	T	0.605329	Sex hormone binding globulin (SHBG)
LDL-C	4	-0.0566	0.0033	1.10002e-66	ieu-b-4871	rs13108218	G	A	0.614877	Sex hormone binding globulin (SHBG)
LDL-C	14	-0.0481	0.0030	6.4003e-58	ieu-b-4870	rs11621792	T	C	0.452552	Sex hormone binding globulin (SHBG)
LDL-C	4	0.0504	0.0032	5.10035e-56	ieu-b-4871	rs1349852	C	A	0.475383	Sex hormone binding globulin (SHBG)
LDL-C	2	-0.0506	0.0032	4.00037e-55	ieu-b-4870	rs12471768	C	T	0.704457	Sex hormone binding globulin (SHBG)
LDL-C	4	-0.0468	0.0031	7.50067e-53	ieu-b-4870	rs13108218	G	A	0.614831	Sex hormone binding globulin (SHBG)
LDL-C	12	-0.0594	0.0057	2.80027e-25	ieu-b-4871	rs12320328	G	A	0.084393	Sex hormone binding globulin (SHBG)
LDL-C	12	-0.0350	0.0034	1.69981e-24	ieu-b-4871	rs1169288	C	A	0.315876	Sex hormone binding globulin (SHBG)
LDL-C	14	-0.0286	0.0032	4.40048e-19	ieu-b-4871	rs11621792	T	C	0.453741	Sex hormone binding globulin (SHBG)
LDL-C	2	-0.0303	0.0035	2.29985e-18	ieu-b-4871	rs12471768	C	T	0.703876	Sex hormone binding globulin (SHBG)
LDL-C	12	-0.0430	0.0053	6.59933e-16	ieu-b-4870	rs12320328	G	A	0.084214	Sex hormone binding globulin (SHBG)
LDL-C	1	-0.0276	0.0039	1.59993e-12	ieu-b-4871	rs12027388	G	C	0.207683	Sex hormone binding globulin (SHBG)
LDL-C	11	0.0387	0.0061	2.99999e-10	ieu-b-4871	rs11601507	A	C	0.069695	Sex hormone binding globulin (SHBG)
LDL-C	5	0.0184	0.0030	1.2e-09	ieu-b-4870	rs12916	C	T	0.400234	Sex hormone binding globulin (SHBG)
LDL-C	12	-0.0190	0.0032	2.59998e-09	ieu-b-4870	rs1169288	C	A	0.316671	Sex hormone binding globulin (SHBG)
LDL-C	6	0.0216	0.0037	3.2e-09	ieu-b-4871	rs12179053	T	C	0.252181	Sex hormone binding globulin (SHBG)
LDL-C	10	0.0180	0.0031	5.39995e-09	ieu-b-4870	rs1336455	A	G	0.359053	Sex hormone binding globulin (SHBG)
LDL-C	12	0.0175	0.0031	9.20005e-09	ieu-b-4870	rs1362965	T	C	0.628327	Sex hormone binding globulin (SHBG)
LDL-C	12	0.0183	0.0033	2.69998e-08	ieu-b-4871	rs1362965	T	C	0.628538	Sex hormone binding globulin (SHBG)
LDL-C	14	0.0173	0.0033	1.79999e-07	ieu-b-4871	rs12891477	T	C	0.367704	Sex hormone binding globulin (SHBG)
LDL-C	11	0.0291	0.0057	3.89996e-07	ieu-b-4870	rs11601507	A	C	0.069501	Sex hormone binding globulin (SHBG)
LDL-C	4	-0.0443	0.0091	9.90011e-07	ieu-b-4870	rs1229984	C	T	0.97335	Sex hormone binding globulin (SHBG)
LDL-C	2	-0.0154	0.0032	1.2e-06	ieu-b-4871	rs11895352	T	C	0.476485	Sex hormone binding globulin (SHBG)
LDL-C	4	0.0138	0.0030	3.50002e-06	ieu-b-4870	rs1349852	C	A	0.475076	Sex hormone binding globulin (SHBG)

LDL-C	11	0.0212	0.0046	3.69999e-06	ieu-b-4871	rs12271333	C	A	0.13893	Sex hormone binding globulin (SHBG)
LDL-C	10	0.1248	0.0032	0.0001	ieu-b-4871	rs1463994339	T	C	0.485387	Sex hormone binding globulin (SHBG)
LDL-C	10	0.0770	0.0030	6.5013e-148	ieu-b-4870	rs1463994339	T	C	0.485831	Sex hormone binding globulin (SHBG)
LDL-C	1	0.0402	0.0032	1.99986e-36	ieu-b-4871	rs1497406	G	A	0.577235	Sex hormone binding globulin (SHBG)
LDL-C	12	0.0436	0.0037	7.70016e-32	ieu-b-4871	rs2122982	A	G	0.238251	Sex hormone binding globulin (SHBG)
LDL-C	14	0.0725	0.0074	1.69981e-22	ieu-b-4871	rs17580	A	T	0.047832	Sex hormone binding globulin (SHBG)
LDL-C	17	-0.0811	0.0085	2.09991e-21	ieu-b-4870	rs1801689	C	A	0.030385	Sex hormone binding globulin (SHBG)
LDL-C	17	-0.0845	0.0091	1.59993e-20	ieu-b-4871	rs1801689	C	A	0.030469	Sex hormone binding globulin (SHBG)
LDL-C	11	-0.0271	0.0031	2.49977e-18	ieu-b-4870	rs174564	G	A	0.348407	Sex hormone binding globulin (SHBG)
LDL-C	6	0.0516	0.0059	3.80014e-18	ieu-b-4871	rs1800562	A	G	0.076991	Sex hormone binding globulin (SHBG)
LDL-C	12	0.0291	0.0035	3.59998e-17	ieu-b-4870	rs2122982	A	G	0.239334	Sex hormone binding globulin (SHBG)
LDL-C	10	0.0237	0.0030	1.29987e-15	ieu-b-4870	rs2068888	A	G	0.4516	Sex hormone binding globulin (SHBG)
LDL-C	14	0.0459	0.0070	4.10015e-11	ieu-b-4870	rs17580	A	T	0.047472	Sex hormone binding globulin (SHBG)
LDL-C	22	-0.0173	0.0030	7.10003e-09	ieu-b-4870	rs165722	T	C	0.515456	Sex hormone binding globulin (SHBG)
LDL-C	11	-0.0168	0.0033	4.09996e-07	ieu-b-4871	rs174564	G	A	0.349606	Sex hormone binding globulin (SHBG)
LDL-C	10	0.0154	0.0032	1.2e-06	ieu-b-4871	rs2068888	A	G	0.449215	Sex hormone binding globulin (SHBG)
LDL-C	2	0.0154	0.0032	1.6e-06	ieu-b-4871	rs17050272	A	G	0.409011	Sex hormone binding globulin (SHBG)
LDL-C	17	0.0885	0.0043	8.69961e-93	ieu-b-4870	rs34914463	C	T	0.13137	Sex hormone binding globulin (SHBG)
LDL-C	3	-0.0564	0.0034	3.19963e-60	ieu-b-4870	rs28478252	C	A	0.756439	Sex hormone binding globulin (SHBG)
LDL-C	17	0.0721	0.0047	4.00037e-54	ieu-b-4871	rs34914463	C	T	0.130312	Sex hormone binding globulin (SHBG)
LDL-C	3	-0.0551	0.0037	1.50003e-50	ieu-b-4871	rs28478252	C	A	0.754427	Sex hormone binding globulin (SHBG)
LDL-C	4	-0.0414	0.0032	2.49977e-37	ieu-b-4871	rs3775228	T	C	0.399457	Sex hormone binding globulin (SHBG)
LDL-C	4	-0.0300	0.0030	3.50026e-23	ieu-b-4870	rs3775228	T	C	0.399549	Sex hormone binding globulin (SHBG)
LDL-C	2	-0.0313	0.0033	3.10027e-21	ieu-b-4871	rs2972146	T	G	0.647864	Sex hormone binding globulin (SHBG)
LDL-C	2	-0.0278	0.0031	1.9002e-19	ieu-b-4870	rs2972146	T	G	0.64708	Sex hormone binding globulin (SHBG)
LDL-C	1	-0.0269	0.0032	2.60016e-17	ieu-b-4871	rs939177387	T	G	0.443178	Sex hormone binding globulin (SHBG)

LDL-C	1	-0.0273	0.0040	9.8992e-12	ieu-b-4870	rs267733	G	A	0.160766	Sex hormone binding globulin (SHBG)
LDL-C	8	-0.0357	0.0055	8.80035e-11	ieu-b-4870	rs796851304	G	A	0.078415	Sex hormone binding globulin (SHBG)
LDL-C	10	-0.0229	0.0035	9.60064e-11	ieu-b-4871	rs2250802	A	G	0.7252	Sex hormone binding globulin (SHBG)
LDL-C	20	-0.0194	0.0031	5.60003e-10	ieu-b-4870	rs2618567	T	G	0.657848	Sex hormone binding globulin (SHBG)
LDL-C	1	-0.0256	0.0043	2.80001e-09	ieu-b-4871	rs267733	G	A	0.161078	Sex hormone binding globulin (SHBG)
LDL-C	6	-0.0240	0.0041	4.09996e-09	ieu-b-4870	rs3127580	T	C	0.15503	Sex hormone binding globulin (SHBG)
LDL-C	4	-0.0228	0.0043	9.80009e-08	ieu-b-4871	rs41280463	A	G	0.164254	Sex hormone binding globulin (SHBG)
LDL-C	7	-0.0149	0.0030	5.1e-07	ieu-b-4870	rs35363532	C	CT	0.509799	Sex hormone binding globulin (SHBG)
LDL-C	7	-0.0155	0.0032	1.5e-06	ieu-b-4871	rs41785	A	C	0.416734	Sex hormone binding globulin (SHBG)
LDL-C	2	-0.0186	0.0039	1.7e-06	ieu-b-4870	rs3820897	C	T	0.819702	Sex hormone binding globulin (SHBG)
LDL-C	5	0.0189	0.0041	3.29997e-06	ieu-b-4871	rs2522062	G	A	0.187569	Sex hormone binding globulin (SHBG)
LDL-C	5	0.0171	0.0038	5.80003e-06	ieu-b-4870	rs2522062	G	A	0.188477	Sex hormone binding globulin (SHBG)
LDL-C	18	0.0202	0.0045	7.10003e-06	ieu-b-4870	rs66785255	C	T	0.123385	Sex hormone binding globulin (SHBG)
LDL-C	4	0.0730	0.0038	1.99986e-81	ieu-b-4871	rs4860987	T	A	0.259115	Sex hormone binding globulin (SHBG)
LDL-C	17	0.0573	0.0040	5.10035e-47	ieu-b-4871	rs55714927	T	C	0.191042	Sex hormone binding globulin (SHBG)
LDL-C	17	0.0413	0.0037	2.09991e-28	ieu-b-4870	rs55714927	T	C	0.189524	Sex hormone binding globulin (SHBG)
LDL-C	17	0.0209	0.0034	6.29999e-10	ieu-b-4871	rs56000661	C	A	0.313671	Sex hormone binding globulin (SHBG)
LDL-C	5	0.0189	0.0032	3.09999e-09	ieu-b-4871	rs6896005	C	T	0.549391	Sex hormone binding globulin (SHBG)
LDL-C	20	0.0188	0.0033	8.10009e-09	ieu-b-4870	rs6093446	A	G	0.288069	Sex hormone binding globulin (SHBG)
LDL-C	17	-0.0246	0.0045	4.20001e-08	ieu-b-4871	rs68023264	A	G	0.141774	Sex hormone binding globulin (SHBG)
LDL-C	6	-0.0178	0.0033	6.80002e-08	ieu-b-4871	rs4935356	G	T	0.516454	Sex hormone binding globulin (SHBG)
LDL-C	20	0.0182	0.0035	2.1e-07	ieu-b-4871	rs6093446	A	G	0.287266	Sex hormone binding globulin (SHBG)
LDL-C	3	-0.0165	0.0035	1.89998e-06	ieu-b-4870	rs60852193	A	G	0.241503	Sex hormone binding globulin (SHBG)
LDL-C	17	-0.0177	0.0037	2.30001e-06	ieu-b-4870	rs1435836890	A	T	0.190146	Sex hormone binding globulin (SHBG)
LDL-C	19	0.0190	0.0041	2.90001e-06	ieu-b-4871	rs201505713	G	C	0.187483	Sex hormone binding globulin (SHBG)

LDL-C	17	-0.0178	0.0040	7.19996e-06	ieu-b-4871	rs1435836890	A	T	0.190509	Sex hormone binding globulin (SHBG)
LDL-C	12	0.1382	0.0114	1.39991e-33	ieu-b-4870	rs1256427846	G	T	0.020684	Sex hormone binding globulin (SHBG)
LDL-C	12	0.1370	0.0123	5.19996e-29	ieu-b-4871	rs1256427846	G	T	0.020727	Sex hormone binding globulin (SHBG)
LDL-C	1	0.0326	0.0034	9.79941e-22	ieu-b-4871	rs867772	G	A	0.683073	Sex hormone binding globulin (SHBG)
LDL-C	12	-0.0235	0.0030	4.79954e-15	ieu-b-4870	rs7960935	C	T	0.43565	Sex hormone binding globulin (SHBG)
LDL-C	2	-0.0483	0.0076	2e-10	ieu-b-4870	rs78058190	A	G	0.05061	Sex hormone binding globulin (SHBG)
LDL-C	17	0.0479	0.0078	8.79995e-10	ieu-b-4870	rs78173576	G	T	0.036439	Sex hormone binding globulin (SHBG)
LDL-C	17	-0.0283	0.0047	1.29999e-09	ieu-b-4871	rs72631343	G	C	0.129086	Sex hormone binding globulin (SHBG)
LDL-C	17	0.0540	0.0106	3.29997e-07	ieu-b-4871	rs77542162	G	A	0.022462	Sex hormone binding globulin (SHBG)
LDL-C	7	-0.0361	0.0072	5.89997e-07	ieu-b-4870	rs799157	C	T	0.956293	Sex hormone binding globulin (SHBG)
LDL-C	17	0.0405	0.0083	1.2e-06	ieu-b-4871	rs78173576	G	T	0.036462	Sex hormone binding globulin (SHBG)
Apo-A1	10	0.1431	0.0032	0.0001	ieu-b-4871	rs10740118	C	G	0.417301	Sex hormone binding globulin (SHBG)
Apo-A1	2	-0.0396	0.0030	1.59993e-40	ieu-b-4870	rs11688492	C	T	0.455937	Sex hormone binding globulin (SHBG)
Apo-A1	11	-0.0268	0.0031	4.79954e-18	ieu-b-4870	rs102275	C	T	0.35119	Sex hormone binding globulin (SHBG)
Apo-A1	2	0.0256	0.0032	5.90065e-16	ieu-b-4870	rs1047891	A	C	0.315465	Sex hormone binding globulin (SHBG)
Apo-A1	16	-0.0240	0.0032	1.50003e-13	ieu-b-4871	rs11639845	T	C	0.398168	Sex hormone binding globulin (SHBG)
Apo-A1	17	-0.0236	0.0035	1e-11	ieu-b-4871	rs10454087	T	C	0.284438	Sex hormone binding globulin (SHBG)
Apo-A1	2	0.0529	0.0083	1.7e-10	ieu-b-4871	rs113017476	A	G	0.038733	Sex hormone binding globulin (SHBG)
Apo-A1	12	-0.0230	0.0044	1.5e-07	ieu-b-4871	rs10876447	A	G	0.154262	Sex hormone binding globulin (SHBG)
Apo-A1	14	0.0210	0.0041	3.09999e-07	ieu-b-4870	rs1458814917	C	T	0.152868	Sex hormone binding globulin (SHBG)
Apo-A1	1	0.0220	0.0044	6.29999e-07	ieu-b-4870	rs1198423	T	G	0.869611	Sex hormone binding globulin (SHBG)
Apo-A1	11	-0.0163	0.0033	8.60003e-07	ieu-b-4871	rs102275	C	T	0.352471	Sex hormone binding globulin (SHBG)
Apo-A1	1	-0.0157	0.0033	1.6e-06	ieu-b-4871	rs12133576	G	A	0.630684	Sex hormone binding globulin (SHBG)
Apo-A1	2	0.0638	0.0030	3.10027e-99	ieu-b-4870	rs1260326	C	T	0.605329	Sex hormone binding globulin (SHBG)
Apo-A1	4	0.0498	0.0032	1.10002e-55	ieu-b-4871	rs1902023	C	A	0.475486	Sex hormone binding globulin (SHBG)
Apo-A1	1	-0.0702	0.0058	9.8992e-34	ieu-b-4871	rs12729444	G	T	0.080863	Sex hormone binding globulin (SHBG)

Apo-A1	8	0.0286	0.0033	1.20005e-17	ieu-b-4871	rs2081687	C	T	0.662177	Sex hormone binding globulin (SHBG)
Apo-A1	12	0.0287	0.0034	7.89951e-17	ieu-b-4870	rs2229357	A	G	0.241517	Sex hormone binding globulin (SHBG)
Apo-A1	10	0.0237	0.0030	1.29987e-15	ieu-b-4870	rs2068888	A	G	0.4516	Sex hormone binding globulin (SHBG)
Apo-A1	10	-0.0230	0.0035	8.19974e-11	ieu-b-4871	rs2255141	G	A	0.724823	Sex hormone binding globulin (SHBG)
Apo-A1	8	0.0185	0.0031	3.50002e-09	ieu-b-4870	rs2081687	C	T	0.663699	Sex hormone binding globulin (SHBG)
Apo-A1	5	0.0204	0.0035	8e-09	ieu-b-4871	rs2112161	A	G	0.71981	Sex hormone binding globulin (SHBG)
Apo-A1	4	-0.0443	0.0091	9.90011e-07	ieu-b-4870	rs1229984	C	T	0.97335	Sex hormone binding globulin (SHBG)
Apo-A1	2	0.0154	0.0032	1.6e-06	ieu-b-4871	rs17050272	A	G	0.409011	Sex hormone binding globulin (SHBG)
Apo-A1	4	0.0136	0.0030	4e-06	ieu-b-4870	rs1902023	C	A	0.475143	Sex hormone binding globulin (SHBG)
Apo-A1	7	0.0134	0.0030	6.4e-06	ieu-b-4870	rs1534696	A	C	0.540358	Sex hormone binding globulin (SHBG)
Apo-A1	6	0.0193	0.0044	9.69996e-06	ieu-b-4870	rs17630640	G	A	0.131358	Sex hormone binding globulin (SHBG)
Apo-A1	4	0.0734	0.0057	1.29987e-37	ieu-b-4871	rs3752440	A	G	0.084755	Sex hormone binding globulin (SHBG)
Apo-A1	1	0.0355	0.0035	8.30042e-25	ieu-b-4871	rs2642438	G	A	0.701824	Sex hormone binding globulin (SHBG)
Apo-A1	2	-0.0312	0.0033	4.60045e-21	ieu-b-4871	rs2943641	C	T	0.648401	Sex hormone binding globulin (SHBG)
Apo-A1	1	-0.0354	0.0038	6.90081e-21	ieu-b-4871	rs2293476	C	G	0.227709	Sex hormone binding globulin (SHBG)
Apo-A1	10	0.0293	0.0031	1.59993e-20	ieu-b-4871	rs1564794181	C	T	0.497101	Sex hormone binding globulin (SHBG)
Apo-A1	2	-0.0279	0.0031	1.59993e-19	ieu-b-4870	rs2943641	C	T	0.647626	Sex hormone binding globulin (SHBG)
Apo-A1	4	0.0484	0.0054	1.9002e-19	ieu-b-4870	rs3752440	A	G	0.084285	Sex hormone binding globulin (SHBG)
Apo-A1	9	0.0258	0.0032	5.90065e-16	ieu-b-4871	rs2636897	C	A	0.548893	Sex hormone binding globulin (SHBG)
Apo-A1	16	0.0254	0.0032	4.00037e-15	ieu-b-4870	rs2925979	C	T	0.70015	Sex hormone binding globulin (SHBG)
Apo-A1	14	-0.0243	0.0033	1.10002e-13	ieu-b-4871	rs766668229	T	C	0.616769	Sex hormone binding globulin (SHBG)
Apo-A1	1	-0.0261	0.0035	1.10002e-13	ieu-b-4870	rs2293476	C	G	0.227588	Sex hormone binding globulin (SHBG)
Apo-A1	5	0.0221	0.0031	5.00035e-13	ieu-b-4870	rs34145453	G	A	0.367958	Sex hormone binding globulin (SHBG)
Apo-A1	1	0.0207	0.0032	6.59933e-11	ieu-b-4870	rs2820441	C	A	0.316153	Sex hormone binding globulin (SHBG)
Apo-A1	20	-0.0206	0.0032	1.6e-10	ieu-b-4870	rs2424993	C	G	0.69871	Sex hormone binding globulin (SHBG)
Apo-A1	6	-0.0232	0.0037	3.29997e-10	ieu-b-4871	rs2395045	T	C	0.756459	Sex hormone binding globulin (SHBG)

Apo-A1	10	0.0176	0.0030	2.69998e-09	ieu-b-4870	rs1564794181	C	T	0.495551	Sex hormone binding globulin (SHBG)
Apo-A1	2	-0.0348	0.0061	8.99995e-09	ieu-b-4870	rs3761706	A	G	0.063485	Sex hormone binding globulin (SHBG)
Apo-A1	6	-0.0192	0.0034	2.30001e-08	ieu-b-4870	rs2395045	T	C	0.756553	Sex hormone binding globulin (SHBG)
Apo-A1	1	-0.0197	0.0036	3.2e-08	ieu-b-4870	rs267738	G	T	0.219141	Sex hormone binding globulin (SHBG)
Apo-A1	9	0.0178	0.0033	5e-08	ieu-b-4871	rs2811929	A	G	0.62062	Sex hormone binding globulin (SHBG)
Apo-A1	9	-0.0194	0.0036	8.60003e-08	ieu-b-4871	rs2810489	A	G	0.257827	Sex hormone binding globulin (SHBG)
Apo-A1	6	0.0172	0.0032	1e-07	ieu-b-4871	rs3756772	T	C	0.400932	Sex hormone binding globulin (SHBG)
Apo-A1	3	-0.0200	0.0038	1.2e-07	ieu-b-4870	rs34810344	A	G	0.188391	Sex hormone binding globulin (SHBG)
Apo-A1	1	0.0157	0.0032	1.2e-06	ieu-b-4870	rs2642438	G	A	0.703589	Sex hormone binding globulin (SHBG)
Apo-A1	5	0.0156	0.0033	2.19999e-06	ieu-b-4871	rs34145453	G	A	0.367947	Sex hormone binding globulin (SHBG)
Apo-A1	1	0.0160	0.0034	2.39999e-06	ieu-b-4871	rs2820441	C	A	0.316325	Sex hormone binding globulin (SHBG)
Apo-A1	10	-0.0575	0.0044	2.70023e-39	ieu-b-4871	rs3829125	G	C	0.154043	Sex hormone binding globulin (SHBG)
Apo-A1	22	0.0490	0.0041	1.29987e-32	ieu-b-4871	rs3827385	C	T	0.179845	Sex hormone binding globulin (SHBG)
Apo-A1	10	-0.0414	0.0041	5.50047e-24	ieu-b-4870	rs3829125	G	C	0.153813	Sex hormone binding globulin (SHBG)
Apo-A1	5	-0.0342	0.0034	7.00003e-24	ieu-b-4870	rs459193	G	A	0.74755	Sex hormone binding globulin (SHBG)
Apo-A1	8	0.0539	0.0055	2.39994e-22	ieu-b-4871	rs4841132	G	A	0.909214	Sex hormone binding globulin (SHBG)
Apo-A1	15	-0.0789	0.0092	1.29987e-17	ieu-b-4870	rs55707100	T	C	0.026243	Sex hormone binding globulin (SHBG)
Apo-A1	5	-0.0298	0.0036	2.70023e-16	ieu-b-4871	rs459193	G	A	0.746141	Sex hormone binding globulin (SHBG)
Apo-A1	8	0.0402	0.0051	4.79954e-15	ieu-b-4870	rs4841132	G	A	0.908377	Sex hormone binding globulin (SHBG)
Apo-A1	11	-0.0288	0.0039	1.99986e-13	ieu-b-4870	rs4409785	C	T	0.171655	Sex hormone binding globulin (SHBG)
Apo-A1	1	-0.0246	0.0035	2.09991e-12	ieu-b-4870	rs3790414	A	T	0.229584	Sex hormone binding globulin (SHBG)
Apo-A1	22	0.0268	0.0038	3.50026e-12	ieu-b-4870	rs3827385	C	T	0.180119	Sex hormone binding globulin (SHBG)
Apo-A1	19	0.0282	0.0041	5.60015e-12	ieu-b-4870	rs429358	C	T	0.154275	Sex hormone binding globulin (SHBG)
Apo-A1	22	0.0246	0.0038	8.00018e-11	ieu-b-4870	rs4821124	C	T	0.189354	Sex hormone binding globulin (SHBG)
Apo-A1	1	-0.0229	0.0038	1.09999e-09	ieu-b-4871	rs3790414	A	T	0.229545	Sex hormone binding globulin (SHBG)
Apo-A1	4	-0.0172	0.0030	6.1e-09	ieu-b-4870	rs3912391	A	G	0.506942	Sex hormone binding globulin (SHBG)

Apo-A1	2	-0.0172	0.0032	1.09999e-07	ieu-b-4871	rs4563251	C	T	0.391827	Sex hormone binding globulin (SHBG)
Apo-A1	4	-0.0155	0.0030	1.79999e-07	ieu-b-4870	rs3822072	A	G	0.451186	Sex hormone binding globulin (SHBG)
Apo-A1	7	0.0148	0.0030	5.39995e-07	ieu-b-4870	rs4731702	T	C	0.491495	Sex hormone binding globulin (SHBG)
Apo-A1	2	-0.0148	0.0030	9.59997e-07	ieu-b-4870	rs4563251	C	T	0.391825	Sex hormone binding globulin (SHBG)
Apo-A1	17	0.0133	0.0029	5.80003e-06	ieu-b-4870	rs4129767	A	G	0.48926	Sex hormone binding globulin (SHBG)
Apo-A1	11	-0.0188	0.0042	7.39997e-06	ieu-b-4871	rs4409785	C	T	0.173458	Sex hormone binding globulin (SHBG)
Apo-A1	3	-0.0676	0.0035	9.49948e-82	ieu-b-4870	rs645040	T	G	0.773679	Sex hormone binding globulin (SHBG)
Apo-A1	2	0.0408	0.0046	3.69999e-19	ieu-b-4870	rs75297654	T	C	0.119262	Sex hormone binding globulin (SHBG)
Apo-A1	4	-0.0220	0.0034	7.10068e-11	ieu-b-4870	rs1215217296	T	C	0.741626	Sex hormone binding globulin (SHBG)
Apo-A1	12	0.0202	0.0032	1.89998e-10	ieu-b-4870	rs7133378	A	G	0.317897	Sex hormone binding globulin (SHBG)
Apo-A1	6	0.0178	0.0031	6.4e-09	ieu-b-4870	rs632057	G	T	0.628155	Sex hormone binding globulin (SHBG)
Apo-A1	4	-0.0227	0.0043	1e-07	ieu-b-4871	rs72729610	G	A	0.164649	Sex hormone binding globulin (SHBG)
Apo-A1	5	-0.0346	0.0066	1.6e-07	ieu-b-4870	rs76026733	C	T	0.052786	Sex hormone binding globulin (SHBG)
Apo-A1	19	0.0168	0.0034	5.39995e-07	ieu-b-4871	rs731839	A	G	0.66429	Sex hormone binding globulin (SHBG)
Apo-A1	5	-0.0164	0.0033	5.49997e-07	ieu-b-4871	rs6866471	A	G	0.631493	Sex hormone binding globulin (SHBG)
Apo-A1	3	-0.0151	0.0031	1.2e-06	ieu-b-4870	rs6773682	A	G	0.345513	Sex hormone binding globulin (SHBG)
Apo-A1	10	0.0140	0.0030	2.80001e-06	ieu-b-4870	rs703974	G	A	0.41391	Sex hormone binding globulin (SHBG)
Apo-A1	1	0.0262	0.0032	1.29987e-16	ieu-b-4871	rs9427104	T	C	0.479978	Sex hormone binding globulin (SHBG)
Apo-A1	4	0.0198	0.0034	7.90005e-09	ieu-b-4870	rs976002	G	A	0.245001	Sex hormone binding globulin (SHBG)
Apo-B	2	0.0883	0.0032	1.09901e-162	ieu-b-4871	rs4665972	C	T	0.603941	Sex hormone binding globulin (SHBG)
Apo-B	2	0.0630	0.0030	1.20005e-95	ieu-b-4870	rs4665972	C	T	0.605376	Sex hormone binding globulin (SHBG)
Apo-B	14	-0.0439	0.0030	3.10027e-49	ieu-b-4870	rs72694391	C	T	0.480748	Sex hormone binding globulin (SHBG)
Apo-B	17	0.0573	0.0040	5.10035e-47	ieu-b-4871	rs55714927	T	C	0.191042	Sex hormone binding globulin (SHBG)
Apo-B	17	-0.0403	0.0029	1.59993e-42	ieu-b-4870	rs56325564	A	G	0.482067	Sex hormone binding globulin (SHBG)
Apo-B	3	-0.0480	0.0039	2.29985e-35	ieu-b-4871	rs7610507	G	C	0.786526	Sex hormone binding globulin (SHBG)
Apo-B	3	-0.0448	0.0036	3.19963e-35	ieu-b-4870	rs7610507	G	C	0.787723	Sex hormone binding globulin (SHBG)

Apo-B	17	0.0413	0.0037	2.09991e-28	ieu-b-4870	rs55714927	T	C	0.189524	Sex hormone binding globulin (SHBG)
Apo-B	8	0.0519	0.0053	7.50067e-23	ieu-b-4871	rs6601299	C	T	0.899061	Sex hormone binding globulin (SHBG)
Apo-B	8	0.0294	0.0034	1.69981e-18	ieu-b-4871	rs10504255	A	G	0.661887	Sex hormone binding globulin (SHBG)
Apo-B	17	-0.0274	0.0032	3.50026e-18	ieu-b-4871	rs56325564	A	G	0.482407	Sex hormone binding globulin (SHBG)
Apo-B	11	-0.0268	0.0031	4.79954e-18	ieu-b-4870	rs102275	C	T	0.35119	Sex hormone binding globulin (SHBG)
Apo-B	14	-0.0265	0.0032	8.80035e-17	ieu-b-4871	rs72694391	C	T	0.481016	Sex hormone binding globulin (SHBG)
Apo-B	6	0.0493	0.0061	5.90065e-16	ieu-b-4871	rs115740542	C	T	0.073791	Sex hormone binding globulin (SHBG)
Apo-B	8	0.0350	0.0049	7.8001e-13	ieu-b-4870	rs6601299	C	T	0.898346	Sex hormone binding globulin (SHBG)
Apo-B	8	0.0195	0.0031	4.70002e-10	ieu-b-4870	rs10504255	A	G	0.663223	Sex hormone binding globulin (SHBG)
Apo-B	20	-0.0193	0.0031	6.69993e-10	ieu-b-4870	rs2618566	T	G	0.65939	Sex hormone binding globulin (SHBG)
Apo-B	5	0.0184	0.0030	1.2e-09	ieu-b-4870	rs12916	C	T	0.400234	Sex hormone binding globulin (SHBG)
Apo-B	17	-0.0283	0.0047	1.29999e-09	ieu-b-4871	rs72631343	G	C	0.129086	Sex hormone binding globulin (SHBG)
Apo-B	6	-0.0178	0.0033	6.80002e-08	ieu-b-4871	rs4935356	G	T	0.516454	Sex hormone binding globulin (SHBG)
Apo-B	17	0.0540	0.0106	3.29997e-07	ieu-b-4871	rs77542162	G	A	0.022462	Sex hormone binding globulin (SHBG)
Apo-B	11	-0.0163	0.0033	8.60003e-07	ieu-b-4871	rs102275	C	T	0.352471	Sex hormone binding globulin (SHBG)
Apo-B	19	0.0265	0.0058	5.69994e-06	ieu-b-4871	rs1065853	T	G	0.080505	Sex hormone binding globulin (SHBG)
Apo-B	19	0.0406	0.0090	6.59994e-06	ieu-b-4870	rs1081105	C	A	0.027886	Sex hormone binding globulin (SHBG)
25(OH)D	2	0.0913	0.0032	9.7051e-176	ieu-b-4871	rs1260326	C	T	0.603649	Sex hormone binding globulin (SHBG)
25(OH)D	2	0.0638	0.0030	3.10027e-99	ieu-b-4870	rs1260326	C	T	0.605329	Sex hormone binding globulin (SHBG)
25(OH)D	12	-0.0769	0.0044	4.60045e-68	ieu-b-4871	rs1871395	G	A	0.152194	Sex hormone binding globulin (SHBG)
25(OH)D	12	-0.0533	0.0041	2.60016e-38	ieu-b-4870	rs1871395	G	A	0.152399	Sex hormone binding globulin (SHBG)
25(OH)D	2	0.0256	0.0032	5.90065e-16	ieu-b-4870	rs1047891	A	C	0.315465	Sex hormone binding globulin (SHBG)
25(OH)D	8	-0.0206	0.0030	6.59933e-12	ieu-b-4870	rs12056768	G	T	0.583263	Sex hormone binding globulin (SHBG)
25(OH)D	3	-0.0194	0.0030	5.10035e-11	ieu-b-4870	rs1128535	T	C	0.499287	Sex hormone binding globulin (SHBG)
25(OH)D	14	0.0494	0.0080	7.90005e-10	ieu-b-4870	rs142004400	C	A	0.035845	Sex hormone binding globulin (SHBG)
25(OH)D	14	0.0525	0.0087	1.5e-09	ieu-b-4871	rs142004400	C	A	0.035449	Sex hormone binding globulin (SHBG)

25(OH)D	3	-0.0180	0.0032	1.40001e-08	ieu-b-4871	rs1128535	T	C	0.503032	Sex hormone binding globulin (SHBG)
25(OH)D	6	0.0290	0.0052	2.90001e-08	ieu-b-4871	rs1321247	T	A	0.103205	Sex hormone binding globulin (SHBG)
25(OH)D	16	-0.0190	0.0035	3.59998e-08	ieu-b-4871	rs1684600	T	C	0.300168	Sex hormone binding globulin (SHBG)
25(OH)D	15	-0.0224	0.0042	9.29994e-08	ieu-b-4871	rs12324720	A	G	0.172801	Sex hormone binding globulin (SHBG)
25(OH)D	8	-0.0154	0.0032	1.79999e-06	ieu-b-4871	rs12056768	G	T	0.584587	Sex hormone binding globulin (SHBG)
25(OH)D	15	-0.0183	0.0039	2.99999e-06	ieu-b-4870	rs12324720	A	G	0.173171	Sex hormone binding globulin (SHBG)
25(OH)D	4	-0.0453	0.0032	2.99985e-45	ieu-b-4871	rs6834488	T	C	0.424233	Sex hormone binding globulin (SHBG)
25(OH)D	4	-0.0303	0.0030	4.79954e-24	ieu-b-4870	rs6834488	T	C	0.424118	Sex hormone binding globulin (SHBG)
25(OH)D	12	0.0565	0.0056	1e-23	ieu-b-4870	rs73413596	C	T	0.075628	Sex hormone binding globulin (SHBG)
25(OH)D	1	0.0326	0.0034	9.30037e-22	ieu-b-4871	rs2807834	G	T	0.684287	Sex hormone binding globulin (SHBG)
25(OH)D	12	0.0571	0.0060	2.39994e-21	ieu-b-4871	rs73413596	C	T	0.076269	Sex hormone binding globulin (SHBG)
25(OH)D	19	0.0248	0.0038	5.30029e-11	ieu-b-4870	rs4420638	G	A	0.189163	Sex hormone binding globulin (SHBG)
25(OH)D	10	-0.0227	0.0035	9.3994e-11	ieu-b-4871	rs2297991	C	T	0.719673	Sex hormone binding globulin (SHBG)
25(OH)D	2	-0.0136	0.0030	8.40001e-06	ieu-b-4870	rs727857	A	G	0.610284	Sex hormone binding globulin (SHBG)
**Estradiol levels**										
LDL-C	2	0.0056	0.0009	9.60064e-11	ebi-a-GCST 90012105	rs1260326	C	T	0.605566	Estradiol levels
LDL-C	2	0.0716	0.0133	6.72249e-08	ebi-a-GCST 90020091	rs1260326	C	T	0.607103	Estradiol levels
LDL-C	17	0.0037	0.0008	8.9e-06	ebi-a-GCST 90012105	rs704	A	G	0.474924	Estradiol levels
APOA	2	0.0247	0.0022	1.80011e-29	ebi-a-GCST 90012105	rs113017476	A	G	0.039146	Estradiol levels
APOA	2	0.2992	0.0301	2.73464e-23	ebi-a-GCST 90020091	rs113017476	A	G	0.039044	Estradiol levels
APOA	2	0.0056	0.0009	9.60064e-11	ebi-a-GCST	rs1260326	C	T	0.605566	Estradiol levels

					90012105					
APOA	2	0.0716	0.0133	6.72249e-08	ebi-a-GCST 90020091	rs1260326	C	T	0.607103	Estradiol levels
25(OH)D	2	0.0056	0.0009	9.60064e-11	ebi-a-GCST 90012105	rs1260326	C	T	0.605566	Estradiol levels
25(OH)D	2	0.0716	0.0133	6.72249e-08	ebi-a-GCST 90020091	rs1260326	C	T	0.607103	Estradiol levels
25(OH)D	19	0.0075	0.0011	7.19946e-11	ebi-a-GCST 90012105	rs62129966	A	C	0.164239	Estradiol levels
25(OH)D	19	0.1070	0.0169	2.18389e-10	ebi-a-GCST 90020091	rs62129966	A	C	0.164338	Estradiol levels
25(OH)D	4	-0.0053	0.0009	5.60003e-09	ebi-a-GCST 90012105	rs4348160	G	T	0.324254	Estradiol levels
25(OH)D	4	-0.0787	0.0139	1.4238e-08	ebi-a-GCST 90020091	rs4348160	G	T	0.323529	Estradiol levels
**Testosterone levels**										
LDL-C	2	0.0100	0.0400	8.99829e-06	ebi-a-GCST 005074	rs1260326	C	T	NA	Testosterone levels
APOA	2	0.0400	0.0100	8.99829e-06	ebi-a-GCST 005074	rs1260326	C	T	NA	Testosterone levels
Apo-B	2	0.0239	0.0015	1.03657e-57	ebi-a-GCST 90014013	rs4665972	C	T	NA	Testosterone levels
Apo-B	17	0.0154	0.0018	7.14661e-17	ebi-a-GCST 90014013	rs55714927	T	C	NA	Testosterone levels
Apo-B	11	-0.0075	0.0015	6.26874e-07	ebi-a-GCST 90014013	rs102275	C	T	NA	Testosterone levels

Apo-B	1	-0.0069	0.0015	5.15965e-06	ebi-a-GCST90014013	rs7534572	G	C	NA	Testosterone levels
25(OH)D	2	0.0400	0.0100	8.99829e-06	ebi-a-GCST005074	rs1260326	C	T	NA	Testosterone levels
**Estrogen sulfotransferase**										
LDL-C	17	0.1414	0.0246	8.70964e-09	prot-a-2892	rs704	A	G	0.46665	Estrogen sulfotransferase
25(OH)D	19	-0.2767	0.0313	8.51138e-19	prot-a-2892	rs4420638	G	A	0.18912	Estrogen sulfotransferase
**Female correlation traits**										
APOA	4	0.0227	0.0516	6.56901e-06	finn-b-N14_FITUB	rs1892534	T	C	0.4815	Female infertility, tubal origin
APOA	4	0.0203	0.0181	7.09202e-06	finn-b-N14_FEMGENPROL	rs13056506	T	G	0.6082	Female genital prolapse

Table S7 Cochran Q test result and MR-Egger intercept in univariable MR-Outcome:Serum-25(OH)D

Outcome	Exposure	Q test		MR-Egger		
		Q-statistic	Q_p	Intercept	SE	P
Serum-25(OH)D	HDL-C	4054.198	0	0.001837046	0.00085142	0.03175324
	LDL-C	1862.208	3.1787E-223	0.0003496763	0.000519383	0.5013062
	TG	725.8228	1.1527E-120	0.000136768	0.00197082	0.944951
	APO-A1	2660.75	0	0.001971369	0.000703289	0.005460296
	APOB	911.6721	6.2439E-160	0.00311025	0.002877105	0.2851977

Table S8 Cochran Q test result and MR-Egger intercept in univariable MR-Exposure:Serum-25(OH)D

Outcome	Exposure	Q test		MR-Egger		
		Q-statistic	Q_p	Intercept	SE	P
HDL-C	Serum-25(OH)D	7385.84866320079	0	-0.010840242	0.00508600892645852	0.0351864609808817
LDL-C	Serum-25(OH)D	24606.0921118778	0	0.00100100153062304	0.00289673679857738	0.730320272987847
TG	Serum-25(OH)D	3150.05065601478	0	-0.008678921	0.00586614242080619	0.142620367008375
APO-A1	Serum-25(OH)D	24316.7980486233	0	-0.009407879	0.0108613101932457	0.389481234162102
APOB	Serum-25(OH)D	3118.70085908551	0	-0.008480572	0.00391288871857424	0.0322705216383633

Table S9 Results of Bayesian tests for co-localization

	snp	V.df1	z.df1	r.df1	lABF.df1	V.df2	z.df2	r.df2	lABF.df2	internal.sum.lABF	SNP.PP.H4
LDL-C and Serum-25(OH)D	rs11591147	5.178673369e-05	-63.62283396	0.997023891990047	2015.00048915762	6.301978225e-05	5.44	0.9972	11.81	2026.81	1
ApoB and and Serum-25(OH)D	rs7412	5.75209997476e-05	-59.14987092	0.997397073002249	1741.82461563952	1.38357217296e-05	8.36	0.9994	31.28	1773.11	1

Table S10 The results of MR-radial method of Size-Defined Lipoprotein Particle Fractions on CA Risk

Exposure	Method	p.value	b
L-VLDL-C%	MR Egger	2.04204E-07	0.851601974
L-VLDL-C%	Weighted median	0.000884599	0.450661652
L-VLDL-C%	Inverse variance weighted (fixed effects)	1.62048E-07	0.487477379
L-VLDL-CE%	MR Egger	5.70359E-07	0.855323953
L-VLDL-CE%	Weighted median	0.024744626	0.288430279
L-VLDL-CE%	Inverse variance weighted (fixed effects)	3.37054E-05	0.41545616
L-VLDL-FC%	MR Egger	0.006129751	0.870478472
L-VLDL-FC%	Weighted median	0.02356329	0.40886523
L-VLDL-FC%	Inverse variance weighted (fixed effects)	0.000357491	0.537984954
L-VLDL-TG%	MR Egger	0.009520302	-0.49021788
L-VLDL-TG%	Weighted median	0.013650999	-0.32172901
L-VLDL-TG%	Inverse variance weighted (fixed effects)	0.000109978	-0.431912904
M-VLDL-C	MR Egger	0.001409782	0.491537024
M-VLDL-C	Weighted median	0.000242839	0.471981955
M-VLDL-C	Inverse variance weighted (fixed effects)	1.19262E-07	0.49603423
M-VLDL-C%	MR Egger	5.61972E-05	0.612214863
M-VLDL-C%	Weighted median	0.006445884	0.324214654
M-VLDL-C%	Inverse variance weighted (fixed effects)	0.007340261	0.264509417
M-VLDL-CE	MR Egger	0.009962065	0.383038672
M-VLDL-CE	Weighted median	0.000430175	0.433709921

M-VLDL-CE	Inverse variance weighted (fixed effects)	5.72786E-07	0.483427736
M-VLDL-CE	MR Egger	0.000109989	0.57841548
M-VLDL-CE	Weighted median	0.019151987	0.291717386
M-VLDL-CE	Inverse variance weighted (fixed effects)	0.020623498	0.227649553
M-VLDL-FC	MR Egger	0.007409758	0.488632488
M-VLDL-FC	Weighted median	2.63068E-05	0.572712242
M-VLDL-FC	Inverse variance weighted (fixed effects)	0.000180647	0.394853682
M-VLDL-FC%	MR Egger	1.1636E-05	0.72467273
M-VLDL-FC%	Weighted median	0.000392507	0.427456177
M-VLDL-FC%	Inverse variance weighted (fixed effects)	0.001758713	0.321264674
M-VLDL-TL	MR Egger	0.098530041	0.33438252
M-VLDL-TL	Weighted median	0.339961842	0.14061376
M-VLDL-TL	Inverse variance weighted (fixed effects)	0.018023882	0.270023502
M-VLDL-Con	MR Egger	0.029345638	0.438077033
M-VLDL-Con	Weighted median	0.017971275	0.338483032
M-VLDL-Con	Inverse variance weighted (fixed effects)	0.001642165	0.347038255
M-VLDL-PL	MR Egger	0.015150697	0.467506512
M-VLDL-PL	Weighted median	2.37279E-05	0.558094339
M-VLDL-PL	Inverse variance weighted (fixed effects)	0.000567246	0.377382934
M-VLDL-PL%	MR Egger	6.36458E-05	0.624364163
M-VLDL-PL%	Weighted median	0.000222192	0.435666134
M-VLDL-PL%	Inverse variance weighted (fixed effects)	3.6666E-05	0.395548515
M-VLDL-TG%	MR Egger	8.10282E-06	-0.670461666
M-VLDL-TG%	Weighted median	0.002240488	-0.359918636
M-VLDL-TG%	Inverse variance weighted (fixed effects)	0.003476386	-0.285603082
S-VLDL-C	MR Egger	0.020654911	0.433610031

S-VLDL-C	Weighted median	0.000147618	0.504085968
S-VLDL-C	Inverse variance weighted (fixed effects)	0.000172637	0.392105255
S-VLDL-C%	MR Egger	0.000179624	0.555420366
S-VLDL-C%	Weighted median	0.000736199	0.412715384
S-VLDL-C%	Inverse variance weighted (fixed effects)	0.000403569	0.345937049
S-VLDL-CE	MR Egger	0.013032495	0.470279255
S-VLDL-CE	Weighted median	0.091329234	0.222749934
S-VLDL-CE	Inverse variance weighted (fixed effects)	0.000376631	0.363872063
S-VLDL-CE	MR Egger	0.000266521	0.554174101
S-VLDL-CE	Weighted median	0.00025709	0.404302215
S-VLDL-CE	Inverse variance weighted (fixed effects)	4.77706E-06	0.43925769
S-VLDL-FC	MR Egger	0.012171729	0.427648912
S-VLDL-FC	Weighted median	1.18854E-05	0.548661357
S-VLDL-FC	Inverse variance weighted (fixed effects)	8.27109E-06	0.439549131
S-VLDL-FC%	MR Egger	0.000215198	0.489657951
S-VLDL-FC%	Weighted median	0.006005467	0.303914965
S-VLDL-FC%	Inverse variance weighted (fixed effects)	0.018596056	0.212031691
S-VLDL-TL	MR Egger	0.574256306	0.103606694
S-VLDL-TL	Weighted median	0.334154772	0.119279321
S-VLDL-TL	Inverse variance weighted (fixed effects)	0.036972618	0.213259888
S-VLDL-PL	MR Egger	0.014863415	0.454443482
S-VLDL-PL	Weighted median	0.00763363	0.350198486
S-VLDL-PL	Inverse variance weighted (fixed effects)	0.000248123	0.373642858
S-VLDL-PL%	MR Egger	0.000181572	0.497476781
S-VLDL-PL%	Weighted median	0.002969561	0.32498768
S-VLDL-PL%	Inverse variance weighted (fixed effects)	0.008713918	0.238831426

S-VLDL-TG%	MR Egger	0.00012523	-0.552119385
S-VLDL-TG%	Weighted median	0.001700858	-0.370657811
S-VLDL-TG%	Inverse variance weighted (fixed effects)	0.001307071	-0.309035529
VLDL-C	MR Egger	0.06539863	0.397187836
VLDL-C	Weighted median	0.209098699	0.18701499
VLDL-C	Inverse variance weighted (fixed effects)	0.005580907	0.312272394
VLDL-CE	MR Egger	0.006510388	0.507475201
VLDL-CE	Weighted median	1.95681E-05	0.568244643
VLDL-CE	Inverse variance weighted (fixed effects)	6.68287E-05	0.419690244
VLDL-Con	MR Egger	0.166775339	0.28088886
VLDL-Con	Weighted median	0.318722644	0.14788992
VLDL-Con	Inverse variance weighted (fixed effects)	0.007755988	0.286874231
VL-VLDL-C%	MR Egger	4.17758E-05	0.771202351
VL-VLDL-C%	Weighted median	0.003807795	0.406308515
VL-VLDL-C%	Inverse variance weighted (fixed effects)	0.000437597	0.384496608
VL-VLDL-CE%	MR Egger	3.3908E-05	0.716496383
VL-VLDL-CE%	Weighted median	0.007238674	0.369896403
VL-VLDL-CE%	Inverse variance weighted (fixed effects)	0.000361319	0.371965344
VL-VLDL-FC%	MR Egger	5.28474E-05	0.919066157
VL-VLDL-FC%	Weighted median	0.030656218	0.32220031
VL-VLDL-FC%	Inverse variance weighted (fixed effects)	0.000533432	0.418527774
VL-VLDL-PL%	MR Egger	0.055329655	0.518160808
VL-VLDL-PL%	Weighted median	0.006027862	0.453081195
VL-VLDL-PL%	Inverse variance weighted (fixed effects)	3.63509E-06	0.62612748
VL-VLDL-TG%	MR Egger	1.80368E-07	-1.019352229
VL-VLDL-TG%	Weighted median	3.87727E-05	-0.553122415

VL-VLDL-TG%	Inverse variance weighted (fixed effects)	0.000226968	-0.422938311
VS-VLDL-C	MR Egger	0.00271951	0.435463666
VS-VLDL-C	Weighted median	0.000338133	0.410485007
VS-VLDL-C	Inverse variance weighted (fixed effects)	3.57023E-05	0.373082774
VS-VLDL-CE	MR Egger	0.006441819	0.438967912
VS-VLDL-CE	Weighted median	0.000126766	0.443888412
VS-VLDL-CE	Inverse variance weighted (fixed effects)	0.000121963	0.38675991
VS-VLDL-FC	MR Egger	0.012605323	0.371024477
VS-VLDL-FC	Weighted median	0.010622065	0.331006712
VS-VLDL-FC	Inverse variance weighted (fixed effects)	5.05955E-05	0.362426501
VS-VLDL-FC	MR Egger	0.043191875	0.399289345
VS-VLDL-FC	Weighted median	0.011525534	0.33603958
VS-VLDL-FC	Inverse variance weighted (fixed effects)	0.010507256	0.307549334
VS-VLDL-TL	MR Egger	0.038667342	0.308212106
VS-VLDL-TL	Weighted median	0.047851977	0.246805801
VS-VLDL-TL	Inverse variance weighted (fixed effects)	0.00012037	0.347149195
VS-VLDL-Con	MR Egger	0.017430877	0.355105188
VS-VLDL-Con	Weighted median	0.013220237	0.324012084
VS-VLDL-Con	Inverse variance weighted (fixed effects)	4.90242E-05	0.369826097
VS-VLDL-PL	MR Egger	0.043786565	0.289432356
VS-VLDL-PL	Weighted median	0.074445161	0.209340498
VS-VLDL-PL	Inverse variance weighted (fixed effects)	0.000127598	0.338901722
CHY-EL-VLDL-C	MR Egger	0.00037311	1.064297787
CHY-EL-VLDL-C	Weighted median	0.00776165	0.523073934
CHY-EL-VLDL-C	Inverse variance weighted (fixed effects)	0.000290745	0.61708228
CHY-EL-VLDL-CE%	MR Egger	3.31224E-05	1.489120508

CHY-EL-VLDL-CE%	Weighted median	7.22197E-06	0.880359019
CHY-EL-VLDL-CE%	Inverse variance weighted (fixed effects)	2.99589E-05	0.759781664
CHY-EL-VLDL-FC	MR Egger	0.005899832	0.887703671
CHY-EL-VLDL-FC	Weighted median	0.049769819	0.373997995
CHY-EL-VLDL-FC	Inverse variance weighted (fixed effects)	0.005911837	0.506401209
CHY-EL-VLDL-TL	MR Egger	0.008271872	-0.428235186
CHY-EL-VLDL-TL	Weighted median	0.765948276	-0.039169831
CHY-EL-VLDL-TL	Inverse variance weighted (fixed effects)	0.038140055	-0.214643426
CHY-EL-VLDL-PL	MR Egger	0.013927817	-0.36884592
CHY-EL-VLDL-PL	Weighted median	0.796613951	-0.034507073
CHY-EL-VLDL-PL	Inverse variance weighted (fixed effects)	0.027508469	-0.212797541
CHY-EL-VLDL-TG	MR Egger	0.000545251	-0.513289052
CHY-EL-VLDL-TG	Weighted median	0.448963306	-0.105416929
CHY-EL-VLDL-TG	Inverse variance weighted (fixed effects)	0.004784365	-0.240731803
CHY-EL-VLDL-TG%	MR Egger	0.003958798	-0.880850691
CHY-EL-VLDL-TG%	Weighted median	0.069518348	-0.307570927
CHY-EL-VLDL-TG%	Inverse variance weighted (fixed effects)	0.001757752	-0.511834202

**Exposure**	**Method**	**p.value**	**b**
S-VLDL-C	MR Egger	0.020654911	0.433610031
S-VLDL-C	Weighted median	0.000147618	0.504085968
S-VLDL-C	Inverse variance weighted (fixed effects)	0.000172637	0.392105255
S-VLDL-C%	MR Egger	0.000179624	0.555420366
S-VLDL-C%	Weighted median	0.000736199	0.412715384
S-VLDL-C%	Inverse variance weighted (fixed effects)	0.000403569	0.345937049
S-VLDL-CE	MR Egger	0.013032495	0.470279255
S-VLDL-CE	Weighted median	0.091329234	0.222749934

S-VLDL-CE	Inverse variance weighted (fixed effects)	0.000376631	0.363872063
S-VLDL-CE	MR Egger	0.000266521	0.554174101
S-VLDL-CE	Weighted median	0.00025709	0.404302215
S-VLDL-CE	Inverse variance weighted (fixed effects)	4.77706E-06	0.43925769
S-VLDL-FC	MR Egger	0.012171729	0.427648912
S-VLDL-FC	Weighted median	1.18854E-05	0.548661357
S-VLDL-FC	Inverse variance weighted (fixed effects)	8.27109E-06	0.439549131
S-VLDL-FC%	MR Egger	0.000215198	0.489657951
S-VLDL-FC%	Weighted median	0.006005467	0.303914965
S-VLDL-FC%	Inverse variance weighted (fixed effects)	0.018596056	0.212031691
S-VLDL-TL	MR Egger	0.574256306	0.103606694
S-VLDL-TL	Weighted median	0.334154772	0.119279321
S-VLDL-TL	Inverse variance weighted (fixed effects)	0.036972618	0.213259888
S-VLDL-PL	MR Egger	0.014863415	0.454443482
S-VLDL-PL	Weighted median	0.00763363	0.350198486
S-VLDL-PL	Inverse variance weighted (fixed effects)	0.000248123	0.373642858
S-VLDL-PL%	MR Egger	0.000181572	0.497476781
S-VLDL-PL%	Weighted median	0.002969561	0.32498768
S-VLDL-PL%	Inverse variance weighted (fixed effects)	0.008713918	0.238831426
S-VLDL-TG%	MR Egger	0.00012523	-0.552119385
S-VLDL-TG%	Weighted median	0.001700858	-0.370657811
S-VLDL-TG%	Inverse variance weighted (fixed effects)	0.001307071	-0.309035529
VLDL-C	MR Egger	0.06539863	0.397187836
VLDL-C	Weighted median	0.209098699	0.18701499
VLDL-C	Inverse variance weighted (fixed effects)	0.005580907	0.312272394
VLDL-CE	MR Egger	0.006510388	0.507475201

VLDL-CE	Weighted median	1.95681E-05	0.568244643
VLDL-CE	Inverse variance weighted (fixed effects)	6.68287E-05	0.419690244
VLDL-Con	MR Egger	0.166775339	0.28088886
VLDL-Con	Weighted median	0.318722644	0.14788992
VLDL-Con	Inverse variance weighted (fixed effects)	0.007755988	0.286874231
VL-VLDL-C%	MR Egger	4.17758E-05	0.771202351
VL-VLDL-C%	Weighted median	0.003807795	0.406308515
VL-VLDL-C%	Inverse variance weighted (fixed effects)	0.000437597	0.384496608
VL-VLDL-CE%	MR Egger	3.3908E-05	0.716496383
VL-VLDL-CE%	Weighted median	0.007238674	0.369896403
VL-VLDL-CE%	Inverse variance weighted (fixed effects)	0.000361319	0.371965344
VL-VLDL-FC%	MR Egger	5.28474E-05	0.919066157
VL-VLDL-FC%	Weighted median	0.030656218	0.32220031
VL-VLDL-FC%	Inverse variance weighted (fixed effects)	0.000533432	0.418527774
VL-VLDL-PL%	MR Egger	0.055329655	0.518160808
VL-VLDL-PL%	Weighted median	0.006027862	0.453081195
VL-VLDL-PL%	Inverse variance weighted (fixed effects)	3.63509E-06	0.62612748
VL-VLDL-TG%	MR Egger	1.80368E-07	-1.019352229
VL-VLDL-TG%	Weighted median	3.87727E-05	-0.553122415
VL-VLDL-TG%	Inverse variance weighted (fixed effects)	0.000226968	-0.422938311
**Exposure**	**Method**	**p.value**	**b**
VS-VLDL-C	MR Egger	0.00271951	0.435463666
VS-VLDL-C	Weighted median	0.000338133	0.410485007
VS-VLDL-C	Inverse variance weighted (fixed effects)	3.57023E-05	0.373082774
VS-VLDL-CE	MR Egger	0.006441819	0.438967912
VS-VLDL-CE	Weighted median	0.000126766	0.443888412

VS-VLDL-CE	Inverse variance weighted (fixed effects)	0.000121963	0.38675991
VS-VLDL-FC	MR Egger	0.012605323	0.371024477
VS-VLDL-FC	Weighted median	0.010622065	0.331006712
VS-VLDL-FC	Inverse variance weighted (fixed effects)	5.05955E-05	0.362426501
VS-VLDL-FC	MR Egger	0.043191875	0.399289345
VS-VLDL-FC	Weighted median	0.011525534	0.33603958
VS-VLDL-FC	Inverse variance weighted (fixed effects)	0.010507256	0.307549334
VS-VLDL-TL	MR Egger	0.038667342	0.308212106
VS-VLDL-TL	Weighted median	0.047851977	0.246805801
VS-VLDL-TL	Inverse variance weighted (fixed effects)	0.00012037	0.347149195
VS-VLDL-Con	MR Egger	0.017430877	0.355105188
VS-VLDL-Con	Weighted median	0.013220237	0.324012084
VS-VLDL-Con	Inverse variance weighted (fixed effects)	4.90242E-05	0.369826097
VS-VLDL-PL	MR Egger	0.043786565	0.289432356
VS-VLDL-PL	Weighted median	0.074445161	0.209340498
VS-VLDL-PL	Inverse variance weighted (fixed effects)	0.000127598	0.338901722
CHY-EL-VLDL-C	MR Egger	0.00037311	1.064297787
CHY-EL-VLDL-C	Weighted median	0.00776165	0.523073934
CHY-EL-VLDL-C	Inverse variance weighted (fixed effects)	0.000290745	0.61708228
CHY-EL-VLDL-CE%	MR Egger	3.31224E-05	1.489120508
CHY-EL-VLDL-CE%	Weighted median	7.22197E-06	0.880359019
CHY-EL-VLDL-CE%	Inverse variance weighted (fixed effects)	2.99589E-05	0.759781664
CHY-EL-VLDL-FC	MR Egger	0.005899832	0.887703671
CHY-EL-VLDL-FC	Weighted median	0.049769819	0.373997995
CHY-EL-VLDL-FC	Inverse variance weighted (fixed effects)	0.005911837	0.506401209
CHY-EL-VLDL-TL	MR Egger	0.008271872	-0.428235186

CHY-EL-VLDL-TL	Weighted median	0.765948276	-0.039169831
CHY-EL-VLDL-TL	Inverse variance weighted (fixed effects)	0.038140055	-0.214643426
CHY-EL-VLDL-PL	MR Egger	0.013927817	-0.36884592
CHY-EL-VLDL-PL	Weighted median	0.796613951	-0.034507073
CHY-EL-VLDL-PL	Inverse variance weighted (fixed effects)	0.027508469	-0.212797541
CHY-EL-VLDL-TG	MR Egger	0.000545251	-0.513289052
CHY-EL-VLDL-TG	Weighted median	0.448963306	-0.105416929
CHY-EL-VLDL-TG	Inverse variance weighted (fixed effects)	0.004784365	-0.240731803
CHY-EL-VLDL-TG%	MR Egger	0.003958798	-0.880850691
CHY-EL-VLDL-TG%	Weighted median	0.069518348	-0.307570927
CHY-EL-VLDL-TG%	Inverse variance weighted (fixed effects)	0.001757752	-0.511834202

**Exposure**	**Method**	**p.value**	**b**
HDL-C	MR Egger	0.416769427	-0.075871858
HDL-C	Weighted median	0.009503314	-0.271345365
HDL-C	Inverse variance weighted (fixed effects)	0.001785621	-0.197408512
L-HDL-FC	MR Egger	0.001065199	0.392173753
L-HDL-FC	Weighted median	0.109380829	0.16911547
L-HDL-FC	Inverse variance weighted (fixed effects)	0.013992431	0.203652506
L-HDL-FC	MR Egger	0.041473265	-0.335976048
L-HDL-FC	Weighted median	0.423932787	-0.116507651
L-HDL-FC	Inverse variance weighted (fixed effects)	0.022975193	-0.192958846
L-HDL-PL%	MR Egger	0.026538333	-0.32759591
L-HDL-PL%	Weighted median	0.068676174	-0.227611326
L-HDL-PL%	Inverse variance weighted (fixed effects)	0.021771994	-0.184062854
M-HDL-Con	MR Egger	0.246681176	-0.1975168
M-HDL-Con	Weighted median	0.197565881	-0.167289287

M-HDL-Con	Inverse variance weighted (fixed effects)	0.045508646	-0.186389167
M-HDL-EC	MR Egger	0.11450412	-0.241331507
M-HDL-EC	Weighted median	0.244022189	-0.150226439
M-HDL-EC	Inverse variance weighted (fixed effects)	0.049733852	-0.157285852
M-HDL-PL	MR Egger	0.38814077	-0.145397498
M-HDL-PL	Weighted median	0.181612537	-0.174832872
M-HDL-PL	Inverse variance weighted (fixed effects)	0.020554114	-0.189277403
M-HDL-TC	MR Egger	0.020455909	0.358573199
M-HDL-TC	Weighted median	0.000723966	0.406290247
M-HDL-TC	Inverse variance weighted (fixed effects)	2.71558E-06	0.468769919
M-HDL-TL	MR Egger	0.001908803	0.46550349
M-HDL-TL	Weighted median	7.40826E-05	0.490151014
M-HDL-TL	Inverse variance weighted (fixed effects)	1.22835E-10	0.509821791
S-HDL-FC	MR Egger	0.415466806	-0.129821705
S-HDL-FC	Weighted median	0.060273668	-0.245979306
S-HDL-FC	Inverse variance weighted (fixed effects)	0.049723858	-0.205452241
S-HDL-PL	MR Egger	0.005052553	0.344100915
S-HDL-PL	Weighted median	0.262499967	0.123602067
S-HDL-PL	Inverse variance weighted (fixed effects)	0.027395882	0.157778339

**Exposure**	**Method**	**p.value**	**b**
IDL-C	MR Egger	0.006758345	0.377809006
IDL-C	Weighted median	0.001790425	0.350583703
IDL-C	Inverse variance weighted (fixed effects)	5.54063E-05	0.375937035
IDL-CE	MR Egger	0.010504297	0.371823284
IDL-CE	Weighted median	0.001458012	0.358548579
IDL-CE	Inverse variance weighted (fixed effects)	9.67167E-05	0.381964476

IDL-FCE	MR Egger	0.004329732	0.40793546
IDL-FCE	Weighted median	0.001301294	0.375340016
IDL-FCE	Inverse variance weighted (fixed effects)	0.000123926	0.35618288
IDL-TL	MR Egger	0.001991162	0.427311344
IDL-TL	Weighted median	0.001180682	0.376412838
IDL-TL	Inverse variance weighted (fixed effects)	7.80795E-05	0.354427919
IDL-Con	MR Egger	0.004718919	0.413265026
IDL-Con	Weighted median	0.001110973	0.357160329
IDL-Con	Inverse variance weighted (fixed effects)	8.30978E-06	0.435155509
IDL-PL	MR Egger	0.0019942	0.413424891
IDL-PL	Weighted median	0.000478113	0.402708413
IDL-PL	Inverse variance weighted (fixed effects)	3.12328E-05	0.363062355
**Exposure**	**Method**	**p.value**	**b**
Omega-6 fatty acid	MR Egger	0.049580135	0.470641285
Omega-6 fatty acid	Weighted median	0.228603079	0.178930126
Omega-6 fatty acid	Inverse variance weighted (fixed effects)	0.026443788	0.268976537
Phenylalanine	MR Egger	0.030979451	1.206691268
Phenylalanine	Weighted median	0.004892872	0.689343595
Phenylalanine	Inverse variance weighted (fixed effects)	0.003042167	0.632015211
Polyunsaturated fatty acid	MR Egger	0.062706241	0.374092885
Polyunsaturated fatty acid	Weighted median	0.255257916	0.146504475
Polyunsaturated fatty acid	Inverse variance weighted (fixed effects)	0.012788247	0.252502314
Pyruvate	MR Egger	0.238592265	0.581196054
Pyruvate	Weighted median	0.002545322	0.705743955
Pyruvate	Inverse variance weighted (fixed effects)	0.047623839	0.461940664
Remnant C	MR Egger	0.001908803	0.46550349

Remnant C	Weighted median	7.40826E-05	0.490151014
Remnant C	Inverse variance weighted (fixed effects)	1.22835E-10	0.509821791
Sphingomyelin	MR Egger	0.048978939	0.356974945
Sphingomyelin	Weighted median	0.000457236	0.455220932
Sphingomyelin	Inverse variance weighted (fixed effects)	0.021351557	0.249012646
Total C	MR Egger	0.005629465	0.476334353
Total C	Weighted median	0.000478503	0.452694613
Total C	Inverse variance weighted (fixed effects)	0.000103712	0.400160673
Total EC	MR Egger	0.005906896	0.470533984
Total EC	Weighted median	0.000934452	0.448483456
Total EC	Inverse variance weighted (fixed effects)	0.000261661	0.3774306
Total FC	MR Egger	0.003067301	0.527323237
Total FC	Weighted median	0.000232529	0.475476284
Total FC	Inverse variance weighted (fixed effects)	0.000300755	0.389470342
Tyrosine	MR Egger	0.005776078	0.594382536
Tyrosine	Weighted median	0.010755151	0.44287223
Tyrosine	Inverse variance weighted (fixed effects)	0.003774785	0.3517141

Table S11 Analyses for the Causal Impact of Lipids and 25(OH)D on Mediators

Potential Mediators / IDs	Exposure	b	se	pval	Q test		MR-Egger		
					Q-statistic	Q_p	Intercept	SE	pval
**Hypertension data**									

Hypertension（ukb-b-14057）	LDL-C	0.0170	0.0041	0.0001	1708.9293	0.0001	0.0005	0.0002	0.0199
Systolic blood pressure（ieu-b-4818）	LDL-C	0.0079	0.2246	0.9719	594.7715	0.0001	0.0121	0.0111	0.2764
Diastolic blood pressure （ukb-b-7992）	LDL-C	-0.0152	0.0104	0.1439	2086.6503	0.0001	0.0016	0.0005	0.0027
**Insulin and glycemic traits**									
Type 2 diabetes （ieu-a-25）	LDL-C	-0.5982	0.6402	0.3502	233.5934	0.0001	0.0877	0.0327	0.0314
HbA1c(ieu-b-4842)	LDL-C	0.0689	0.0278	0.0133	1903.1624	0.0001	0.0045	0.0014	0.0012
**Cardiometabolic diseases**									
Myocardial infarction（finn-b-I9_MI）	LDL-C	0.5709	0.0438	0.0001	661.3749	0.0001	-0.0032	0.0021	0.1414
Angina pectoris（ebi-a-GCST90018793）	LDL-C	0.4369	0.0269	0.0001	1058.5060	0.0001	0.0004	0.0013	0.7469
Atrial fibrillation（ukb-b-964）	LDL-C	-0.0115	0.0343	0.7375	647.3037	0.0111	-0.0054	0.0017	0.0012
**Respiratory diseases traits**									
Asthma（ieu-a-44）	LDL-C	0.0872	0.0616	0.1565	130.7297	0.8282	-0.0004	0.0028	0.8935
Chronic obstructive airways disease （ukb-b-13447）	LDL-C	-0.0004	0.0004	0.2598	199.4066	0.0825	0.0001	0.0001	0.5904
**Metabolic indices**									
Waist circumference （ukb-a-382）	LDL-C	-0.0148	0.0092	0.1072	1469.4987	0.0001	0.0021	0.0004	0.0001
Hip circumference （ukb-a-388）	LDL-C	-0.0452	0.0124	0.0003	2131.4034	0.0001	0.0005	0.0006	0.4083
Basal metabolic rate （ukb-b-16446）	LDL-C	-0.0378	0.0086	0.0001	3529.7178	0.0001	-0.0001	0.0004	0.9025
Body mass index（ukb-b-19953）	LDL-C	-0.0167	0.0108	0.1221	2354.113	0.0001	0.0019	0.0005	0.0005
Weight (ukb-b-12039)	LDL-C	-0.0413	0.0104	0.0001	2910.7794	0.0001	0.0007	0.0005	0.1915
**Lifestyle habit**									
Alcoholic drinking（ieu-b-73）	LDL-C	-0.0065	0.0098	0.5094	1940.0380	0.0001	-0.0006	0.0005	0.2479
Smoking data（ieu-b-4877）	LDL-C	-0.0050	0.0101	0.6201	598.3321	0.0001	-0.0001	0.0005	0.8535
**Psychological states**									
Anxiety（ukb-b-11311）	LDL-C	0.0002	0.0004	0.6334	136.7984	0.9671	0.0001	0.0001	0.7513
Depression（ukb-b-12064）	LDL-C	-0.0021	0.0011	0.0514	406.6890	0.0017	0.0001	0.0001	0.2569

**Hypertension data**									
Hypertension（ukb-b-14057）	HDL-C	0.0309	0.0086	0.0003	1943.0372	0.0001	-0.0012	0.0002	0.0001
Diastolic blood pressure （ukb-b-7992）	HDL-C	-0.0412	0.0118	0.0005	1958.6712	0.0001	-0.0019	0.0005	0.0004
**Insulin and glycemic traits**									
Type 2 diabetes （ieu-a-25）	HDL-C	-1.8430	0.8859	0.0375	164.3496	0.0001	0.0109	0.0703	0.8822
HbA1c(ieu-b-4842)	HDL-C	-0.0501	0.0224	0.0257	761.6859	0.0001	-0.0032	0.0010	0.0017
**Cardiometabolic diseases**									
Myocardial infarction（finn-b-I9_MI）	HDL-C	-0.3296	0.0487	0.0001	628.6006	0.0001	-0.0030	0.0023	0.1801
Angina pectoris（ebi-a-GCST90018793）	HDL-C	-0.0111	0.0014	0.0001	1058.5060	0.0001	0.0004	0.0013	0.7469
Atrial fibrillation（ukb-b-964）	HDL-C	-0.0012	0.0007	0.4359	381.6980	0.0003	0.0001	0.0001	0.2099
**Respiratory diseases traits**									
Asthma （ieu-a-44）	HDL-C	-0.0264	0.0763	0.7298	178.9343	0.0375	0.0033	0.0036	0.3650
Chronic obstructive airways disease（ukb-b-13447）	HDL-C	0.0004	0.0004	0.3298	191.4507	0.5179	0.0001	0.0001	0.2765
**Metabolic indices**									
Waist circumference （ukb-a-382）	HDL-C	0.0044	0.0193	0.8220	3072.9838	0.0001	-0.0053	0.0006	0.0001
Hip circumference （ukb-a-388）	HDL-C	-0.0771	0.0308	0.0123	4008.4477	0.0001	-0.0036	0.0009	0.0001
Basal metabolic rate （ukb-b-16446）	HDL-C	-0.0385	0.0186	0.0390	5392.2867	0.0001	-0.0029	0.0005	0.0001
Body mass index（ukb-b-19953）	HDL-C	-0.0235	0.0219	0.2829	4917.5858	0.0001	-0.0050	0.0008	0.0001
Weight (ukb-b-12039)	HDL-C	-0.0552	0.0223	0.0133	5425.3444	0.0001	-0.0043	0.0007	0.0001
**Lifestyle habit**									
Alcoholic drinking（ieu-b-73）	HDL-C	0.0414	0.0072	0.0001	825.1740	0.0001	0.0016	0.0003	0.0001
Smoking data（ieu-b-4877）	HDL-C	-0.0007	0.0006	0.2277	711.9220	0.0001	0.0001	0.0001	0.0001
**Psychological states**									
Anxiety（ukb-b-11311）	HDL-C	-0.0004	0.0005	0.3942	220.02545	0.0494	0.0001	0.0001	0.6953
Depression（ukb-b-12064）	HDL-C	0.0002	0.0014	0.7514	415.34751	0.0004	0.0001	0.0001	0.4241

**Hypertension data**									
Hypertension（ukb-b-14057）	TG	0.0309	0.0086	0.0003	368.9144	0.0001	0.0023	0.0006	0.0003
Systolic blood pressure（ieu-b-4818）	TG	0.9198	0.3805	0.0156	119.3547	0.0001	0.0619	0.0289	0.0373
Diastolic blood pressure （ukb-b-7992）	TG	0.0349	0.0191	0.0673	435.5390	0.0001	0.0031	0.0015	0.0380
**Insulin and glycemic traits**									
Type 2 diabetes （ieu-a-25）	TG	-0.4472	0.6144	0.4668	175.7381	0.0001	0.1256	0.0341	0.0346
HbA1c(ieu-b-4842)	TG	-0.0448	0.0411	0.2761	213.1498	0.0001	0.0110	0.0029	0.0004
**Cardiometabolic diseases**									
Myocardial infarction（finn-b-I9_MI）	TG	0.3306	0.0686	0.0001	97.7974	0.0002	0.0077	0.0053	0.1529
Angina pectoris（ebi-a-GCST90018793）	TG	0.2677	0.0425	0.0001	163.8534	0.0001	0.0081	0.0033	0.0173
Atrial fibrillation（ukb-b-964）	TG	-0.0003	0.0009	0.7038	79.4463	0.0085	0.0001	0.0001	0.7692
**Respiratory diseases traits**									
Asthma （ieu-a-44）	TG	-0.0264	0.0763	0.7298	178.9343	0.0375	0.0033	0.0036	0.3650
Chronic obstructive airways disease （ukb-b-13447）	TG	-0.0004	0.0006	0.5522	55.0127	0.0573	0.0001	0.0001	0.5909
**Metabolic indices**									
Waist circumference （ukb-a-382）	TG	0.0044	0.0193	0.8220	412.7333	0.0001	0.0029	0.0015	0.0534
Hip circumference （ukb-a-388）	TG	-0.0771	0.0308	0.0123	837.6423	0.0001	-0.0034	0.0024	0.1668
Basal metabolic rate （ukb-b-16446）	TG	-0.0385	0.0186	0.0390	1044.7683	0.0001	0.0003	0.0015	0.8459
Body mass index（ukb-b-19953）	TG	-0.0235	0.0219	0.2829	628.9756	0.0001	0.0010	0.0017	0.5590
Weight (ukb-b-12039)	TG	-0.0552	0.0223	0.0133	833.2077	0.0001	-0.0011	0.0018	0.5528
**Lifestyle habit**									
Alcoholic drinking（ieu-b-73）	TG	-0.0626	0.0121	0.0001	201.5602	0.0001	-0.0001	0.0010	0.9113
Smoking data（ieu-b-4877）	TG	-0.0114	0.0139	0.4118	77.2110	0.0166	0.0005	0.0011	0.6204
**Psychological states**									
Anxiety（ukb-b-11311）	TG	0.0003	0.0005	0.5935	33.9130	0.61455	0.0001	0.0001	0.1480

Depression（ukb-b-12064）	TG	0.0011	0.0016	0.5036	57.3389	0.31745	0.0001	0.0001	0.3101
**Hypertension data**									
Hypertension（ukb-b-14057）	25(OH)D	-0.0005	0.0092	0.9588	651.8412	0.0001	0.0004	0.4203	0.0001
Systolic blood pressure（ieu-b-4818）	25(OH)D	0.1294	0.5112	0.8001	226.3408	0.0001	0.0082	0.0220	0.7112
Diastolic blood pressure （ukb-b-7992）	25(OH)D	0.0116	0.0220	0.5981	709.4525	0.0001	0.0012	0.0009	0.2049
**Insulin and glycemic traits**									
Type 2 diabetes （ieu-a-25）	25(OH)D	4.1497	0.4627	0.0001	0.2774	0.5984	-	-	-
HbA1c(ieu-b-4842)	25(OH)D	0.0643	0.0423	0.1278	263.0617	0.0001	0.0001	0.0018	0.9505
**Cardiometabolic diseases**									
Myocardial infarction（finn-b-I9_MI）	25(OH)D	-0.1926	0.0893	0.0305	220.5339	0.0001	-0.0027	0.0038	0.4786
Angina pectoris（ebi-a-GCST90018793）	25(OH)D	-0.1606	0.0671	0.0166	453.6941	0.0001	-0.0032	0.0029	0.2665
Atrial fibrillation（ukb-b-964）	25(OH)D	0.0004	0.0013	0.7760	151.8086	0.0010	0.0001	0.0001	0.0738
**Respiratory diseases traits**									
Asthma （ieu-a-44）	25(OH)D	0.0922	0.1132	0.4155	52.2537	0.7800	0.0011	0.0049	0.8199
Chronic obstructive airways disease （ukb-b-13447）	25(OH)D	0.0001	0.0009	0.9881	82.7654	0.01807	0.0001	0.0001	0.7135
**Metabolic indices**									
Waist circumference （ukb-a-382）	25(OH)D	0.0098	0.0209	0.6393	593.3310	0.0001	-0.0002	0.0009	0.8014
Hip circumference （ukb-a-388）	25(OH)D	0.0244	0.0234	0.2970	590.0279	0.0001	0.0005	0.0010	0.6487
Basal metabolic rate （ukb-b-16446）	25(OH)D	0.0304	0.0180	0.0909	1187.8696	0.0001	0.0014	0.0008	0.0615
Body mass index（ukb-b-19953）	25(OH)D	0.0248	0.0250	0.3217	1000.2618	0.0001	0.0007	0.0011	0.4851
Weight (ukb-b-12039)	25(OH)D	0.0351	0.0217	0.1056	965.5068	0.0001	0.001	0.0009	0.263
**Lifestyle habit**									
Alcoholic drinking（ieu-b-73）	25(OH)D	0.0338	0.0179	0.0585	523.5345	0.0001	-0.0006	0.0005	0.2479
Smoking data（ieu-b-4877）	25(OH)D	0.0162	0.0246	0.5097	295.35359	0.0001	0.0028	0.0010	0.0074
**Psychological states**									

Anxiety（ukb-b-11311）	25(OH)D	0.0009	0.0008	0.2558	48.5852	0.7487	0.0001	0.0001	0.7563
Depression（ukb-b-12064）	25(OH)D	-0.0024	0.0027	0.3700	186.8453	0.0001	-0.0001	0.0001	0.2801
**Hypertension data**									
Hypertension（ukb-b-14057）	ApoA1	-0.0142	0.0045	0.0016	1640.8292	0.0001	-0.0003	0.0002	0.1944
Systolic blood pressure（ieu-b-4818）	ApoA1	-0.3240	0.2379	0.1732	571.8790	0.0001	0.0164	0.0121	0.1766
Diastolic blood pressure （ukb-b-7992）	ApoA1	-0.0042	0.0107	0.6922	1747.7311	0.0001	-0.0006	0.0005	0.3026
**Insulin and glycemic traits**	ApoA1								
Type 2 diabetes （ieu-a-25）	ApoA1	-1.4816	0.4381	0.0007	53.0177	0.0001	-0.0218	0.0340	0.5556
HbA1c(ieu-b-4842)	ApoA1	-0.0285	0.0180	0.1119	526.0060	0.0001	0.0001	0.0009	0.9942
**Cardiometabolic diseases**									
Myocardial infarction（finn-b-I9_MI）	ApoA1	-0.1508	0.0437	0.0006	503.4999	0.0001	−0. 0048	0. 0022	0. 0298
Angina pectoris（ebi-a-GCST90018793）	ApoA1	-0.1626	0.0312	0.0001	1009.0732	0.0001	-0.0035	0.0016	0.0270
Atrial fibrillation（ukb-b-964）	ApoA1	-0.0010	0.0006	0.0901	326.3765	0.0002	0.0001	0.0001	0.4474
**Respiratory diseases traits**									
Asthma （ieu-a-44）	ApoA1	-0.0508	0.0559	0.3627	186.5392	0.0668	0.0018	0.0029	0.5486
Chronic obstructive airways disease（ukb-b-13447）	ApoA1	0.0003	0.0004	0.3625	237.2868	0.0006	0.0001	0.0001	0.9644
**Metabolic indices**	ApoA1								
Waist circumference （ukb-a-382）	ApoA1	-0.0108	0.0106	0.3067	1576.0733	0.0001	-0.0005	0.0005	0.3866
Hip circumference （ukb-a-388）	ApoA1	0.0151	0.0143	0.2920	2298.0003	0.0001	0.0014	0.0007	0.0607
Basal metabolic rate （ukb-b-16446）	ApoA1	-0.0141	0.0091	0.1201	3168.8336	0.0001	-0.0002	0.0005	0.7334
Body mass index（ukb-b-19953）	ApoA1	-0.0014	0.0121	0.9086	2445.5041	0.0001	0.0003	0.0006	0.5977
Weight (ukb-b-12039)	ApoA1	-0.0039	0.0115	0.7337	2841.4078	0.0001	0.0002	0.0006	0.7082
**Hypertension data**									
Hypertension（ukb-b-14057）	ApoB	0.0048	0.0082	0.5561	473.4163	0.0001	-0.0004	0.0008	0.6054
Systolic blood pressure（ieu-b-4818）	ApoB	-0.1952	0.3711	0.5988	110.0849	0.0001	-0.0219	0.0387	0.5738

Diastolic blood pressure （ukb-b-7992）	ApoB	-0.0531	0.0213	0.0127	609.5161	0.0001	-0.0013	0.0022	0.5642
**Insulin and glycemic traits**	ApoB								
Type 2 diabetes （ieu-a-25）	ApoB	4.1497	0.4627	0.0000	2.5113	0.1130	-	-	-
HbA1c(ieu-b-4842)	ApoB	0.0250	0.0430	0.5615	342.3683	0.0001	0.0039	0.0042	0.3610
**Cardiometabolic diseases**									
Myocardial infarction（finn-b-I9_MI）	ApoB	0.6252	0.0937	0.0001	194.5324	0.0001	-0.0112	0.0093	0.2305
Angina pectoris（ebi-a-GCST90018793）	ApoB	0.4272	0.0622	0.0001	393.2943	0.0001	0.0007	0.0061	0.9091
Atrial fibrillation（ukb-b-964）	ApoB	0.0015	0.0009	0.0976	326.3765	0.0002	0.0001	0.0001	0.4474
**Respiratory diseases traits**									
Asthma （ieu-a-44）	ApoB	0.0878	0.0979	0.3698	26.6234	0.2259	0.0138	0.0097	0.1691
Chronic obstructive airways disease （ukb-b-13447）	ApoB	-0.0008	0.0005	0.1270	25.6271	0.6453	0.0001	0.0001	0.3777
**Metabolic indices**									
Waist circumference （ukb-a-382）	ApoB	-0.0387	0.0153	0.0112	281.6419	0.0001	0.0027	0.0015	0.0757
Hip circumference （ukb-a-388）	ApoB	-0.0468	0.0222	0.0352	476.9344	0.0001	0.0015	0.0023	0.5196
Basal metabolic rate （ukb-b-16446）	ApoB	-0.0334	0.0154	0.0295	793.9359	0.0001	0.0009	0.0016	0.5452
Body mass index（ukb-b-19953）	ApoB	-0.0363	0.0190	0.0568	531.6160	0.0001	0.0024	0.0019	0.2063
Weight (ukb-b-12039)	ApoB	-0.0467	0.0189	0.0136	672.3350	0.0001	0.0017	0.0019	0.3916
**Lifestyle habit**									
Alcoholic drinking（ieu-b-73）	ApoB	-0.0099	0.0128	0.4414	222.5969	0.0001	-0.0020	0.0013	0.1173
Smoking data（ieu-b-4877）	ApoB	-0.0030	0.0014	0.0286	75.4172	0.01474	-0.0030	0.0014	0.0286
**Psychological states**									
Anxiety（ukb-b-11311）	ApoB	0.0003	0.0006	0.5596	21.9491	0.7836	0.0001	0.0001	0.9111
Depression（ukb-b-12064）	ApoB	-0.0032	0.0016	0.0455	62.2232	0.23464	0.0001	0.0002	0.4920

Table S12 MVMR Analyses for the Causal Impact of Potential Mediators on CA

	Exposure	nsnp	b	se	pval	lo_ci	up_ci	or	or_lci95	or_uci95
HDL-C	High density lipoprotein cholesterol levels	7	0.62	0.68	0.36	-0.71	1.95	1.86	0.49	7.04
HDL-C	Myocardial infarction	1	0.72	0.17	< 0.01	0.39	1.05	2.06	1.48	2.87
HDL-C	Type 2 diabetes	13	0.10	0.09	0.27	-0.08	0.29	1.11	0.92	1.33
ApoA1	Myocardial infarction	1	0.80	0.15	0.00	0.51	1.08	2.22	1.66	2.96
ApoA1	Apolipoprotein A1 levels	6	0.11	0.34	0.74	-0.55	0.78	1.12	0.58	2.18
ApoA1	Type 2 diabetes	8	0.05	0.09	0.55	-0.12	0.22	1.05	0.89	1.25
25(OH)D	Serum 25-Hydroxyvitamin D levels	2	0.42	0.80	0.60	-1.15	1.99	1.52	0.32	7.35
25(OH)D	Angina pectoris	1	0.94	0.39	0.02	0.18	1.70	2.55	1.19	5.46
25(OH)D	Myocardial infarction	1	0.09	0.31	0.76	-0.51	0.69	1.10	0.60	2.00
25(OH)D	Type 2 diabetes	16	0.00	0.06	1.00	-0.11	0.11	1.00	0.90	1.12
ApoB	Angina pectoris	1	-1.00	0.30	< 0.01	-1.58	-0.41	0.37	0.21	0.66
ApoB	Apolipoprotein B levels	1	-0.60	0.11	< 0.01	-0.82	-0.38	0.55	0.44	0.68
ApoB	Myocardial infarction	1	1.36	0.22	< 0.01	0.93	1.78	3.89	2.55	5.94
ApoB	Type 2 diabetes	6	-0.24	0.06	< 0.01	-0.36	-0.12	0.79	0.70	0.89
ApoB	Waist circumference	3	7.94	2.37	0.00	3.30	12.59	28.12	26.98	29.31
ApoB	Hip circumference	5	-2.49	1.52	< 0.01	-5.47	0.50	0.08	0.00	1.64
ApoB	Basal metabolic rate	7	-0.63	0.58	0.28	-1.76	0.50	0.53	0.17	1.66
ApoB	Body mass index (BMI)	4	-3.10	0.98	< 0.01	-5.03	-1.17	0.05	0.01	0.31

ApoB	Diastolic blood pressure, automated reading	0	-4.86	0.72	< 0.01	-6.28	-3.45	0.01	< 0.01	0.03
TG	Triglycerides	22	-0.03	0.10	0.73	-0.23	0.16	0.97	0.79	1.18
TG	Angina pectoris	12	0.47	0.11	< 0.01	0.25	0.69	1.60	1.29	2.00
TG	Myocardial infarction	3	0.29	0.09	< 0.01	0.12	0.45	1.33	1.13	1.58
TG	Hip circumference	132	0.30	0.45	0.50	-0.57	1.17	1.35	0.56	3.24
TG	Weight	272	-1.21	0.82	0.14	-2.82	0.40	0.30	0.06	1.49
TG	Basal metabolic rate	311	1.34	0.65	0.04	0.07	2.60	3.80	1.07	13.52
LDL-C	Low density lipoprotein cholesterol levels	200	-0.07	0.08	0.35	-0.23	0.08	0.93	0.80	1.08
LDL-C	Angina pectoris	14	0.48	0.11	< 0.01	0.27	0.70	1.62	1.31	2.01
LDL-C	Myocardial infarction	5	0.27	0.08	< 0.01	0.12	0.43	1.32	1.13	1.54
LDL-C	Hip circumference	151	-0.64	0.39	0.11	-1.40	0.13	0.53	0.25	1.14
LDL-C	Weight	278	0.69	0.41	0.09	-0.11	1.49	1.99	0.89	4.43

Table S13 Results of Mendelian mediation analysis

Exposure	Mediator	B2	se	pval	or	B1	Mediating effect	Proportion of mediating effect
HDL-C	Myocardial infarction	0.72	0.17	< 0.01	2.06	-0.15	-0.11	0.72
Serum-25(OH)D	Angina pectoris	0.94	0.39	0.02	2.55	-0.16	-0.15	0.49
ApoB	Type 2 diabetes	-0.24	0.06	< 0.01	0.79	-1.55	0.37	0.83
ApoB	Waist circumference	7.94	2.37	< 0.01	2812.25	0.01	0.09	0.20

ApoB	Body mass index (BMI)	-3.10	0.98	< 0.01	0.05	-0.04	0.11	0.25
ApoB	Diastolic blood pressure, automated reading	-4.86	0.72	< 0.01	0.01	-0.05	0.26	0.58
TG	Angina pectoris	0.47	0.11	< 0.01	1.60	0.33	0.16	0.81
TG	Myocardial infarction	0.29	0.09	< 0.01	1.33	0.27	0.08	0.40
LDL-C	Angina pectoris	0.48	0.11	< 0.01	1.62	0.57	0.28	0.82
LDL-C	Myocardial infarction	0.27	0.08	< 0.01	1.32	0.4	0.12	0.36

Table S14 Baseline Characteristics of the Study Population

Characteristic	Overall N = 9988	All-cause mortality No N = 8434	All-cause mortality Yes N = 1554	P-value	CVD mortality No N = 9448	CVD mortality Yes N = 540	P-value
Sex, n (%)				0.058			0.040
Female	5107 (50.84%)	4413 (51.17%)	694 (48.28%)		4878 (51.05%)	229 (45.27%)	
Male	4881 (49.16%)	4021 (48.83%)	860 (51.72%)		4570 (48.95%)	311 (54.73%)	
Age, mean (SE)	50.67 (0.26)	48.58 (0.26)	66.81 (0.45)	< 0.001	49.97 (0.26)	68.97 (0.63)	< 0.001
Age strata, n (%)				< 0.001			< 0.001
<60	5889 (68.67%)	5600 (74.05%)	289 (27.15%)		5810 (70.48%)	79 (21.91%)	
≥60	4099 (31.33%)	2834 (25.95%)	1265 (72.85%)		3638 (29.52%)	461 (78.09%)	
Race, n (%)				< 0.001			< 0.001

Non-Hispanic White	4895 (72.13%)	3869 (70.97%)	1026 (81.11%)		4537 (71.80%)	358 (80.74%)	
Non-Hispanic Black	1721 (9.34%)	1475 (9.31%)	246 (9.52%)		1624 (9.26%)	97 (11.31%)	
Mexican American	1704 (7.80%)	1535 (8.36%)	169 (3.46%)		1649 (7.97%)	55 (3.22%)	
Other Hispanic	849 (4.65%)	778 (4.89%)	71 (2.81%)		831 (4.74%)	18 (2.43%)	
Other Race - Including Multi-Racial	819 (6.08%)	777 (6.47%)	42 (3.10%)		807 (6.23%)	12 (2.31%)	
Education, n (%)				< 0.001			< 0.001
College or higher	4963 (57.54%)	4387 (59.53%)	576 (42.17%)		4767 (58.24%)	196 (39.46%)	
Less than college	5025 (42.46%)	4047 (40.47%)	978 (57.83%)		4681 (41.76%)	344 (60.54%)	
Marital, n (%)				< 0.001			< 0.001
Divorced/separated/widowed	2418 (20.42%)	1791 (18.29%)	627 (36.85%)		2179 (19.58%)	239 (42.11%)	
Married/living with a partner	6348 (67.04%)	5514 (68.34%)	834 (57.00%)		6080 (67.63%)	268 (51.80%)	
Never married	1222 (12.54%)	1129 (13.37%)	93 (6.15%)		1189 (12.79%)	33 (6.09%)	
Poverty-income ratio, n (%)				< 0.001			< 0.001
<1.3	2965 (20.39%)	2447 (19.63%)	518 (26.30%)		2795 (20.21%)	170 (25.18%)	
1.3-3.5	3924 (37.16%)	3217 (35.98%)	707 (46.21%)		3656 (36.68%)	268 (49.50%)	
≥3.5	3099 (42.45%)	2770 (44.39%)	329 (27.49%)		2997 (43.11%)	102 (25.33%)	
Smoke, n (%)				< 0.001			< 0.001
Current smoker	2061 (21.41%)	1745 (21.09%)	316 (23.92%)		1970 (21.47%)	91 (20.00%)	
Former smoker	2856 (28.05%)	2223 (26.60%)	633 (39.25%)		2642 (27.68%)	214 (37.74%)	
Never smoker	5071 (50.54%)	4466 (52.31%)	605 (36.84%)		4836 (50.85%)	235 (42.27%)	

Alcohol, n (%)	7237 (76.95%)	6211 (78.18%)	1026 (67.40%)	< 0.001	6902 (77.44%)	335 (64.14%)	< 0.001
BMI, mean (SE)	30.20 (0.11)	30.31 (0.12)	29.35 (0.22)	< 0.001	30.21 (0.11)	29.93 (0.33)	0.6
BMI group, n (%)				< 0.001			0.4
Low	77 (0.82%)	57 (0.74%)	20 (1.48%)		73 (0.83%)	4 (0.67%)	
Normal	1989 (20.46%)	1597 (19.80%)	392 (25.76%)		1874 (20.38%)	115 (22.65%)	
Obese	4304 (43.74%)	3756 (44.31%)	548 (39.23%)		4098 (43.68%)	206 (45.23%)	
Overweight	3494 (34.98%)	2963 (35.16%)	531 (33.53%)		3306 (35.11%)	188 (31.45%)	
Physical activity				< 0.001			< 0.001
Low	3728 (31.57%)	2853 (28.89%)	875 (52.23%)		3407 (30.67%)	321 (55.02%)	
Moderate	3328 (34.91%)	2813 (34.83%)	515 (35.55%)		3152 (34.89%)	176 (35.40%)	
Vigorous	2932 (33.52%)	2768 (36.28%)	164 (12.22%)		2889 (34.44%)	43 (9.58%)	
Diabetes, n (%)	2365 (18.49%)	1790 (16.45%)	575 (34.27%)	< 0.001	2157 (17.69%)	208 (39.30%)	< 0.001
Hypertension, n (%)	5214 (48.00%)	4035 (44.68%)	1179 (73.59%)	< 0.001	4782 (46.76%)	432 (80.17%)	< 0.001
CVD, n (%)	1485 (12.31%)	914 (9.30%)	571 (35.52%)	< 0.001	1231 (10.92%)	254 (48.37%)	< 0.001
TC, mean (SE)	203.19 (0.71)	203.63 (0.77)	199.78 (1.28)	0.004	203.43 (0.71)	197.08 (2.38)	0.006
TG, mean (SE)	162.26 (1.96)	161.50 (2.18)	168.10 (3.10)	< 0.001	161.90 (2)	171.48 (5.33)	< 0.001
HDL-C, mean (SE)	49.40 (0.25)	49.19 (0.26)	51.02 (0.67)	0.048	49.37 (0.26)	50.17 (0.83)	0.6
LDL-C, mean (SE)	122.60 (0.58)	123.44 (0.63)	116.06 (1.05)	< 0.001	122.96 (0.59)	113.21 (2.04)	< 0.001
vitamin D, mean (SE)	68.89 (0.57)	69.37 (0.61)	65.12 (0.81)	< 0.001	69.07 (0.58)	64.21 (1.22)	0.002
vitamin D deficiency, n (%)	2989 (23.83%)	2487 (23.17%)	502 (28.94%)	< 0.001	2817 (23.59%)	172 (30.00%)	0.003
vitamin D group, n (%)				< 0.001			0.017
<25	347 (2.41%)	287 (2.29%)	60 (3.27%)		322 (2.34%)	25 (4.01%)	

25-50	2642 (21.42%)	2200 (20.87%)	442 (25.67%)		2495 (21.25%)	147 (25.99%)
50-75	3870 (39.88%)	3271 (40.03%)	599 (38.69%)		3665 (39.96%)	205 (37.77%)
≥75	3129 (36.29%)	2676 (36.80%)	453 (32.37%)		2966 (36.45%)	163 (32.24%)

Data are presented as weighted mean (SE) or unweighted frequencies (weighted percentages);

Abbreviation: BMI, body mass index; CVD, coronary heart disease; TC, total cholesterol; TG, triglycerides; HDL-C, high-density lipoprotein cholesterol; LDL-C, low-density lipoprotein cholesterol .

Table S15 Baseline Characteristics of the Study Population

**Characteristic**	**Overall N = 9988**	**Vitamin D concentrations (nmol/L)**				**P-value**
		**<25 N = 347**	**25-49.99 N = 2642**	**50-74.99 N = 3870**	**≥75 N = 3129**	
Sex, n (%)						< 0.001
Female	5107 (50.84%)	216 (65.55%)	1387 (53.13%)	1774 (44.48%)	1730 (55.48%)	
Male	4881 (49.16%)	131 (34.45%)	1255 (46.87%)	2096 (55.52%)	1399 (44.52%)	
Age, mean (SE)	50.67 (0.26)	45.93 (1.01)	47.37 (0.41)	48.99 (0.36)	54.79 (0.35)	< 0.001
Age strata, n (%)						< 0.001
<60	5889 (68.67%)	248 (80.40%)	1740 (75.58%)	2405 (73.23%)	1496 (58.80%)	
≥60	4099 (31.33%)	99 (19.60%)	902 (24.42%)	1465 (26.77%)	1633 (41.20%)	
Race, n (%)						< 0.001
Non-Hispanic White	4895 (72.13%)	48 (26.08%)	741 (50.11%)	1990 (74.45%)	2116 (85.63%)	
Non-Hispanic Black	1721 (9.34%)	193 (47.51%)	822 (21.99%)	438 (5.64%)	268 (3.40%)	
Mexican American	1704 (7.80%)	63 (15.85%)	608 (13.46%)	757 (8.67%)	276 (2.95%)	

Other Hispanic	849 (4.65%)	15 (2.83%)	237 (6.29%)	386 (5.42%)	211 (2.96%)	
Other Race - Including Multi-Racial	819 (6.08%)	28 (7.74%)	234 (8.15%)	299 (5.81%)	258 (5.05%)	
Education, n (%)						< 0.001
College or higher	4963 (57.54%)	138 (40.20%)	1195 (50.85%)	1885 (57.32%)	1745 (62.88%)	
Less than college	5025 (42.46%)	209 (59.80%)	1447 (49.15%)	1985 (42.68%)	1384 (37.12%)	
Marital, n (%)						< 0.001
Divorced/separated/widowed	2418 (20.42%)	107 (25.60%)	657 (22.30%)	874 (18.52%)	780 (21.06%)	
Married/living with a partner	6348 (67.04%)	153 (46.84%)	1536 (59.59%)	2565 (68.71%)	2094 (70.94%)	
Never married	1222 (12.54%)	87 (27.56%)	449 (18.11%)	431 (12.77%)	255 (8.00%)	
Poverty-income ratio, n (%)						< 0.001
<1.3	2965 (20.39%)	138 (37.18%)	908 (27.57%)	1164 (19.99%)	755 (15.48%)	
1.3-3.5	3924 (37.16%)	153 (45.54%)	1091 (40.31%)	1478 (36.78%)	1202 (35.16%)	
≥3.5	3099 (42.45%)	56 (17.28%)	643 (32.12%)	1228 (43.23%)	1172 (49.36%)	
Smoke, n (%)						< 0.001
Current smoker	2061 (21.41%)	115 (36.20%)	631 (25.83%)	795 (21.28%)	520 (17.97%)	
Former smoker	2856 (28.05%)	63 (17.72%)	588 (20.24%)	1154 (29.34%)	1051 (31.94%)	
Never smoker	5071 (50.54%)	169 (46.08%)	1423 (53.93%)	1921 (49.38%)	1558 (50.10%)	
Alcohol, n (%)	7237 (76.95%)	248 (73.04%)	1817 (71.86%)	2823 (77.18%)	2349 (79.95%)	< 0.001
BMI, mean (SE)	30.20 (0.11)	33.69 (0.66)	32.23 (0.22)	30.24 (0.16)	28.75 (0.16)	< 0.001
BMI group, n (%)						< 0.001
Low	77 (0.82%)	1 (0.13%)	14 (0.77%)	31 (0.74%)	31 (0.99%)	
Normal	1989 (20.46%)	43 (17.44%)	390 (13.80%)	727 (18.50%)	829 (26.72%)	
Obese	4304 (43.74%)	212 (59.84%)	1391 (56.61%)	1631 (44.89%)	1070 (33.90%)	
Overweight	3494 (34.98%)	80 (22.58%)	810 (28.83%)	1432 (35.88%)	1172 (38.39%)	
Physical activity						< 0.001

Low	3728 (31.57%)	173 (49.70%)	1158 (40.14%)	1335 (29.25%)	1062 (27.86%)	
Moderate	3328 (34.91%)	97 (27.09%)	781 (32.28%)	1316 (35.43%)	1134 (36.42%)	
Vigorous	2932 (33.52%)	77 (23.20%)	703 (27.59%)	1219 (35.31%)	933 (35.72%)	
Diabetes, n (%)	2365 (18.49%)	99 (23.85%)	716 (22.80%)	859 (17.61%)	691 (16.57%)	< 0.001
Hypertension, n (%)	5214 (48.00%)	192 (51.55%)	1392 (49.08%)	1857 (44.86%)	1773 (50.57%)	< 0.001
CVD, n (%)	1485 (12.31%)	54 (15.56%)	379 (12.03%)	529 (11.04%)	523 (13.66%)	0.008
TC, mean (SE)	203.19 (0.71)	197.12 (4.27)	202.27 (1.42)	202.21 (0.83)	205.21 (1.18)	0.011
TG, mean (SE)	162.26 (1.96)	162.48 (22.27)	174.67 (4.93)	168.81 (2.58)	147.73 (2.49)	< 0.001
HDL-C, mean (SE)	49.40 (0.25)	47.77 (1.58)	46.50 (0.36)	47.11 (0.32)	53.74 (0.48)	< 0.001
LDL-C, mean (SE)	122.60 (0.58)	117.28 (3.08)	122.98 (1.23)	122.72 (0.75)	122.59 (1)	0.3
All-cause mortality, n (%)	1554 (11.47%)	60 (15.59%)	442 (13.75%)	599 (11.13%)	453 (10.23%)	< 0.001
CVD mortality, n (%)	540 (3.72%)	25 (6.20%)	147 (4.51%)	205 (3.52%)	163 (3.31%)	0.017

Data are presented as weighted mean (SE) or unweighted frequencies (weighted percentages);

Abbreviation: BMI, body mass index; CVD, coronary heart disease; TC, total cholesterol; TG, triglycerides; HDL-C, high-density lipoprotein cholesterol; LDL-C, low-density lipoprotein cholesterol .

`Table S16 HRs (95% CIs) for All-cause mortality and CVD mortality according to vitamin D concentrations

		Model 1		Model 2		Model 3	
**Characteristic**	**Cases/ participants**	**HR（95% CI）**	**P value**	**HR（95% CI）**	**P value**	**HR（95% CI）**	**P value**
All-cause mortality							
vitamin D Per 1-SD increase	1554/9988	0.921(0.855-0.992)	0.031	0.746(0.688-0.810)	< 0.001	0.851(0.787-0.920)	< 0.001
vitamin D deficiency							
No (<50)	1052/6999	1.000 (Reference)		1.000 (Reference)		1.000 (Reference)	
Yes (≥50)	502/2989	1.271(1.124-1.438)	< 0.001	1.740(1.525-1.984)	< 0.001	1.393(1.206-1.609)	< 0.001
vitamin D group							
<25	60/347	1.000 (Reference)		1.000 (Reference)		1.000 (Reference)	
25-49.99	442/2642	0.699(0.487-1.004)	0.052	0.549(0.389-0.774)	< 0.001	0.596(0.408-0.870)	0.007
50-74.99	599/3870	0.507(0.341-0.754)	< 0.001	0.336(0.228-0.495)	< 0.001	0.280(0.185-0.425)	< 0.001
≥75	453/3129	0.652(0.442-0.962)	0.031	0.505(0.350-0.729)	< 0.001	0.453(0.303-0.676)	< 0.001
CVD mortality							
vitamin D Per 1-SD increase	540/9988	0.880(0.781-0.992)	0.036	0.691(0.601-0.794)	< 0.001	0.815(0.712-0.933)	0.003

vitamin D deficiency							
No (<50)	368/6999	1.000 (Reference)		1.000 (Reference)		1.000 (Reference)	
Yes (≥50)	172/2989	1.338(1.085-1.651)	0.006	1.933(1.540-2.426)	< 0.001	1.439(1.130-1.831)	0.003
vitamin D group							
<25	25/347	1.000 (Reference)		1.000 (Reference)		1.000 (Reference)	
25-49.99	147/2642	0.573(0.316-1.039)	0.067	0.434(0.249-0.757)	0.003	0.481(0.265-0.872)	0.016
50-74.99	205/3870	0.380(0.213-0.677)	0.001	0.232(0.134-0.403)	< 0.001	0.195(0.108-0.352)	< 0.001
≥75	163/3129	0.420(0.235-0.753)	0.004	SS	< 0.001	0.298(0.167-0.530)	< 0.001

Model 1: unadjusted; Model 2: adjusted for age and sex; Model 3: further adjusted for race, education, marital status, poverty-income ratio, alcohol, smoke, physical activity, BMI, hypertension, diabetes, and CVD.

Abbreviation: BMI, body mass index; CVD, coronary heart disease; HR, hazard ratio; CI, confidence interval.

Table S17 Sensitivity Analysis of the Association of the vitamin D concentrations With All-cause mortality and CVD mortality

Characteristic	Cases/participants	OR（95% CI）	p value
Excluding participants with less than 1 years of follow-up			
All-cause mortality			

vitamin D Per 1-SD increase	1449/9858	0.857(0.789-0.931)	< 0.001
vitamin D deficiency			
No (<50)	983/6912	1.000 (Reference)	
Yes (≥50)	466/2946	1.401(1.209-1.623)	< 0.001
vitamin D group			
<25	51/337	1.000 (Reference)	
25-49.99	415/2609	0.739(0.503-1.086)	0.12
50-74.99	556/3820	0.558(0.386-0.807)	0.002
≥75	427/3092	0.511(0.341-0.766)	0.001
CVD mortality			
vitamin D Per 1-SD increase	506/9858	0.823(0.719-0.942)	0.005
vitamin D deficiency			
No (<50)	346/6912	1.000 (Reference)	
Yes (≥50)	160/2946	1.464(1.149-1.865)	0.002
vitamin D group			
<25	22/337	1.000 (Reference)	
25-49.99	138/2609	0.540(0.309-0.942)	0.030
50-74.99	192/3820	0.402(0.243-0.665)	< 0.001
≥75	154/3092	0.363(0.214-0.616)	< 0.001

Each model was adjusted for age, sex, race, education, marital status, poverty-income ratio, alcohol, smoke, physical activity, BMI, hypertension, diabetes, and CVD.

Abbreviation: BMI, body mass index; CVD, coronary heart disease; HR, hazard ratio; CI, confidence interval.

Table S18 Colocalization results of eQTLs for 9 genes with Apolipoprotein A1, HDL and Serum

25-Hydroxyvitamin D associated SNPs

Outcome	Gene	PP.H0	PP.H1	PP.H2	PP.H3	PP.H4
Apolipoprotein A1	BATF2	0	0	0	0.0004	0.9996
Apolipoprotein A1	CA8	0	0.168	0	0.0005	0.8315
Apolipoprotein A1	ACP2	0	0	0	0.0017	0.9983
Apolipoprotein A1	GBP1	0	0	0	0	1
HDL	CEACA,6	0	0.0021	0	0	0.9979
HDL	HMGCR	0	0	0	0.0085	0.9915
HDL	GFUS	0	0.0737	0	0.0005	0.9258
HDL	CBX6	0	0.1482	0	0.0028	0.849
Serum 25(OH)-D	NMRAL1	0	0.002	0	0.0116	0.9863

PP.H0–PP.H4 represent the posterior probabilities of diferent hypotheses, PP.H4>0.8 represents a strong colocalization between gene expression and disease risk

Table S19 The impact of druggable genes on lipid traits, vitamin D and the risk of cardiac arrest

Exposure	Outcome	Method	or	or_lci95	or_uci95	Estimate	P-value
**GBP1**							
	Apolipoprotein A1	Inverse variance weighted	0.917726397	0.879676215	0.95742243	0.92(0.88-0.96)	0.001
	Cardiac arrest	Inverse variance	1.302875873	1.038125344	1.635145072	1.30(1.04-1.64)	0.022

		weighted					
**ACP2**							
	Apolipoprotein A1	Inverse variance weighted	0.972544799	0.947451408654417	0.998302791	0.97(0.95-0.99)	0.037
	Cardiac arrest	Inverse variance weighted	0.896208633	0.818493078671264	0.981303244271175	0.90(0.82-0.98)	0.018
**BATF2**							
	Apolipoprotein A1	Inverse variance weighted	0.838951847118279	0.813729179	0.864956327	0.84(0.81-0.86)	0.001
	Cardiac arrest	Inverse variance weighted	1.639077224	1.151744791	2.332612369	1.64(1.15-2.33)	0.006
**CEACA,6**							
	HDL	Inverse variance weighted	0.962401251125328	0.9379751757026	0.987463413	0.96(0.94-0.99)	0.003
	Cardiac arrest	Inverse variance weighted	0.783287376915899	0.645738861222525	0.950135034	0.78(0.65-0.95)	0.013
**CA8**							
	HDL	Inverse variance weighted	0.966864624948191	0.954894	0.978985	0.97(0.95-0.98)	0.001
	Cardiac arrest	Inverse variance weighted	0.819545344	0.692548479	0.969830404	0.82(0.69-0.97)	0.021
**HMGCR**							
	HDL	Inverse variance weighted	1.056251928	1.014521891	1.099698434	1.06(1.01-1.10)	0.008
	Cardiac arrest	Inverse variance weighted	0.790407422	0.625218838	0.999240353	0.79(0.62-0.99)	0.049

**GFUS**							
	HDL	Inverse variance weighted	1.044255017	1.007079641	1.082802686	1.04(1.01-1.08)	0.019
	Cardiac arrest	Inverse variance weighted	0.823190144	0.696060428	0.973539058	0.82(0.69-0.97)	0.023
**NMRAL1**							
	25-Hydroxyvitamin D	Inverse variance weighted	0.986023864	0.9776539	0.9944655	0.99(0.98-0.99)	0.001
	Cardiac arrest	Inverse variance weighted	1.1398001	1.10553741	1.2309798	1.13(1.11-1.23)	0.001
**CBX6**							
	HDL	Inverse variance weighted	1.014791095	1.002905858	1.026817181	1.01(1.00-1.03)	0.015
	Cardiac arrest	Inverse variance weighted	0.879525988	0.783962639	0.986738302	0.87(0.78-0.99)	0.029

Table S20 Candidate drug predicted using DSigDB and TCMSP

Term	P-value	Adjusted P-value	Odds Ratio	Combined Score	Genes
Sanguinarine	0.0014	0.027	91.2261	601.4396	CBX6;HMGCR
Tetraprenol	0.0022	0.027	713.6786	4351.6858	HMGCR
Tetrandrine	0.003	0.027	525.7368	3054.5885	HMGCR

Lycorine	0.0054	0.0286	44.5047	232.3172	CBX6;HMGCR
Apocarotenal	0.0082	0.0902	245.8889	1181.6582	GBP1
Chenodeoxycholic	0.0084	0.0318	181.2909	866.985	HMGCR
Papaverine	0.0097	0.0337	155.7266	721.5902	HMGCR
Beta-carotene	0.0104	0.0902	193.1553	882.4375	GBP1
Harmine	0.0108	0.0347	140.3239	635.916	CBX6
Thapsigargin	0.0204	0.0477	73.0184	284.1664	CBX6
Ellipticine	0.024	0.0518	61.9906	231.3173	HMGCR
Arachidonic	0.025	0.0523	59.3713	219.0372	HMGCR
Arbutin	0.0417	0.069	35.0819	111.457	CBX6
Pregnenolone	0.048	0.0765	30.3596	92.2111	HMGCR

Table S21 Docking results of available proteins with small molecules

Target gene	UniProtKB ID	Drug	PubChem ID	Binding energy （kcal/mol）
HMGCR	P04035	sanguinarine	MOL001474	-7.4
HMGCR	P04035	lycorine	MOL004136	-7.2
HMGCR	P04035	chenodeoxycholic	MOL008842	-7.2
HMGCR	P04035	papaverine	MOL006980	-6.6

HMGCR	P04035	Tetraprenol (7614-21-3)	MOL005703	-5.2
CBX6	O95503	sanguinarine	MOL001474	-6.3
CBX6	O95503	lycorine	MOL004136	-5.7
